# A dinickel-catalyzed three-component cycloaddition of vinylidenes†

**DOI:** 10.1039/d2sc02696a

**Published:** 2022-09-08

**Authors:** Annah E. Kalb, Mingxin Liu, Megan I. Bosso, Christopher Uyeda

**Affiliations:** Department of Chemistry, Purdue University West Lafayette Indiana 47907 USA cuyeda@purdue.edu

## Abstract

A dinickel catalyst promotes the [2 + 2 + 1]-cycloaddition of two aldehyde equivalents and a vinylidene. The resulting methylenedioxolane products can be deprotected in one pot under acidic conditions to reveal α-hydroxy ketones. This method provides convenient access to unsymmetrical alkyl-substituted α-hydroxy ketones, which are challenging to synthesize selectively using cross-benzoin reactions. Mechanistic studies are consistent with an initial migratory insertion of the aldehyde into a dinickel bridging vinylidene. Insertion of the second aldehyde followed by C–O reductive elimination furnishes the cycloadduct. Under dilute conditions, an enone side product is generated due to a competing β-hydride elimination from the proposed metallacyclic intermediate. A DFT model consistent with the concentration-dependent formation of the methylenedioxolane and enone is presented.

## Abstract 2

A dinickel catalyst promotes reductive [2 + 2 + 1]-cycloaddition reactions using readily available 1,1-dichloroalkenes as vinylidene precursors.

## Graphical abstract

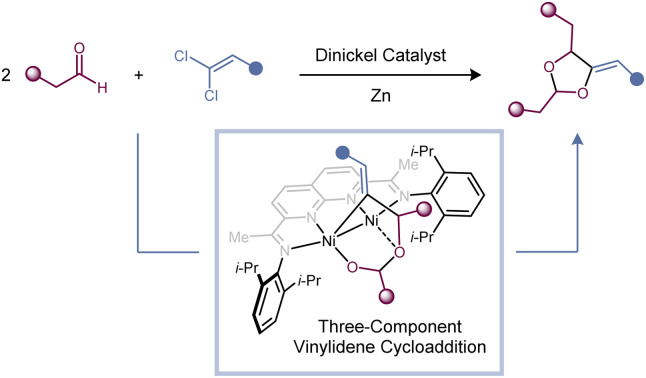

## Introduction

**Fig. 1:**
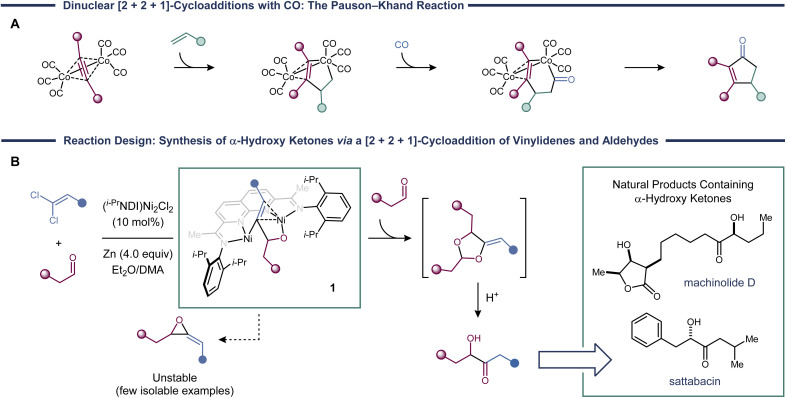
(a) A mechanism of the Pauson–Khand reaction initiated by a dinuclear oxidative coupling of the alkene and the alkyne. (b) A proposed vinylidene [2 + 2 + 1]-cycloaddition process involving a metallacycle derived from the migratory insertion of a 2π-component into a Ni_2_(vinylidene).

[2 + 2 + 1]-Cycloadditions provide some of the most direct routes to five-membered rings and feature in numerous total syntheses of polycyclic natural products.^1^ The Pauson–Khand reaction is the prototypical example in this class of cycloadditions and uses Co_2_(CO)_8_ to mediate the coupling of an alkyne, an alkene, and CO (Fig. 1a).^2^ Since its initial discovery, other transition metal catalysts have been found to promote Pauson–Khand reactions,^3^ and variants where the alkene is replaced with a hetero-π-system, such as an imine^4^ or an aldehyde,^5^ have been developed. In most cases, CO is required to serve as the one-atom partner, though in some cases isonitriles can also be used.^6,7^

It would be synthetically valuable to expand the scope of Pauson–Khand-type reactions to include more reactive classes of carbenes, which have rarely been observed to participate in [2 + 2 + 1]-cycloadditions.^8,9^ The principal challenge is avoiding direct [2 + 1]-additions of the metal carbene, which would lead to the formation of three-membered ring products. Indeed, many of the most common carbene transfer catalysts, such as Rh_2_(CO_2_R)_4_ (ref. 10) and Cu(i) complexes,^11^ carry out cyclopropanation reactions by this concerted pathway, making them unsuitable for the development of three-component cycloadditions.

Dinickel catalysts promote methylenecyclopropanation reactions using vinylidenes derived from 1,1-dichloroalkenes.^12^ Mechanistic studies suggest that, rather than a concerted [2 + 1]-cycloaddition, these reactions proceed in a stepwise manner. The primary evidence came from experiments probing stereospecificity. When a geometrically pure alkene was used, the cyclopropane was formed as a cis/trans mixture. This observation led us to propose a mechanism in which the alkene first undergoes migratory insertion into a Ni_2_(vinylidene) to form an intermediate with the general structure 1 (Fig. 1b). Then, C–C reductive elimination generates the cyclopropane product. We were recently successful in arresting this process and obtaining direct experimental characterization of metallacycle 1.^13^

Based on this stepwise mechanism, we wondered whether metallacycles such as 1 could be intercepted with a third reaction partner to form rings larger than cyclopropanes. Here, we provide an example of such a transformation in the context of a [2 + 2 + 1]-cycloaddition of two aldehyde equivalents and a vinylidene. Key to the realization of this reaction is the fact that competing [2 + 1]-cycloadditions would produce methylene epoxides, which are sufficiently unstable to disfavor reductive elimination.^14^ The [2 + 2 + 1]-cycloaddition products are methylenedioxolanes that can be deprotected under acidic conditions to reveal α-hydroxy ketones.

## Results and discussion

### Reaction development

Reaction optimization studies were carried out using aldehyde 2 (2.0 equiv.) and 1,1-dichloroalkene 3 (1.0 equiv.) as model substrates. Under conditions similar to those previously employed in [2 + 1]-cycloaddition reactions, dinickel catalyst 7 affords methylenedioxolane 4 in 89% yield as an 8 : 1 mixture of diastereomers and with exclusively *Z* stereochemistry at the exocyclic alkene (Table 1, entry 1). Mn provided similar yields to those obtained with Zn (entry 2). Interestingly, the homogeneous reductant Cp_2_Co also produced 4, albeit in significantly lower yield (entry 3). The observation that Zn can be replaced with Cp_2_Co indicates that the reaction does not require the formation of an organozinc intermediate.

**Table 1:** Effect of reaction parameters^a^

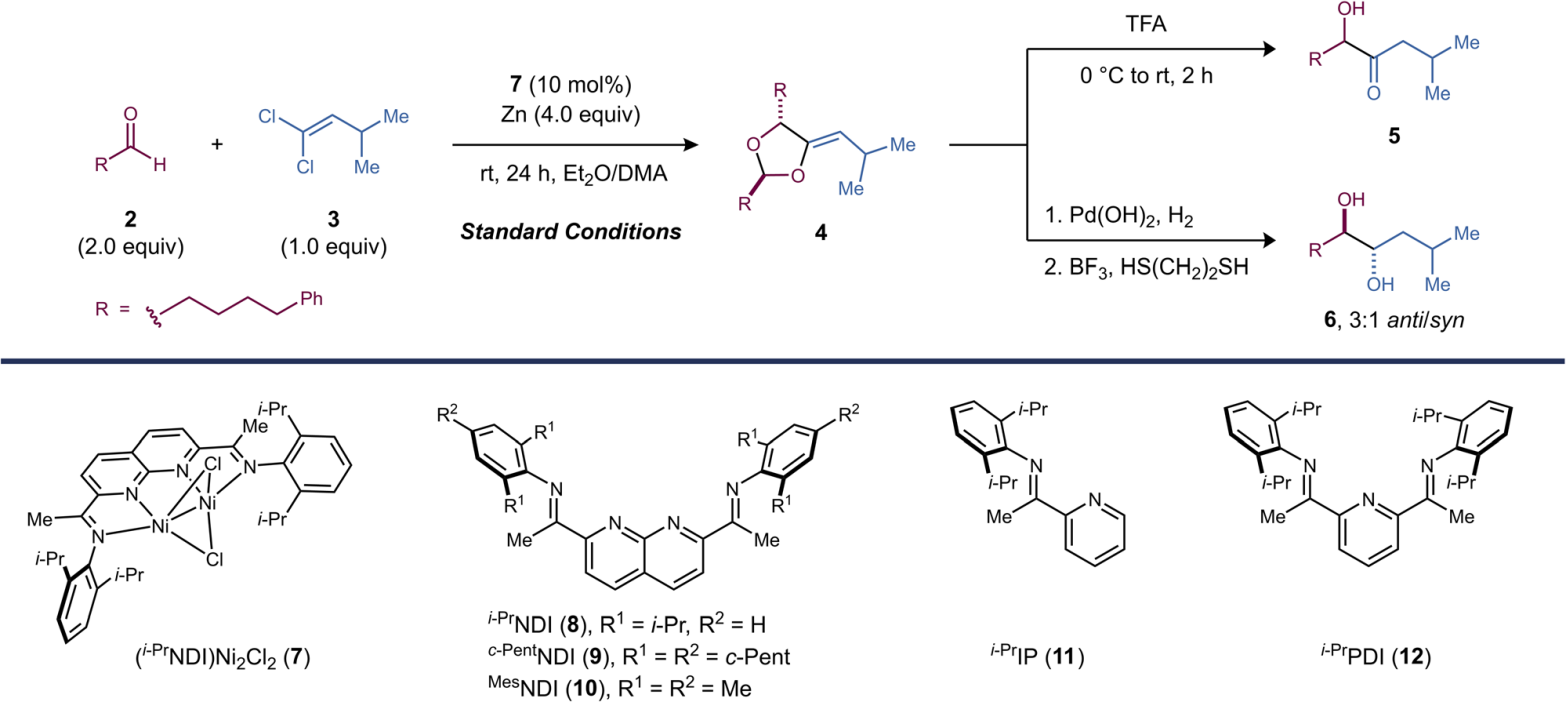				
Entry	Deviations from standard conditions	Yield (%) (4)	Anti : syn (4)	*Z*/*E* (4)
1	None	89	8 : 1	>20 : 1
2	Mn instead of Zn	81	14 : 1	>20 : 1
3	Cp_2_Co instead of Zn	58	1 : 4	>20 : 1
4	No Et_2_O	47	4 : 1	>20 : 1
5	^*i*-Pr^NDI (8) (10 mol%) + Ni(dme)Cl_2_ (20 mol%) instead of 7	85	6 : 1	>20 : 1
6	^*c*-Pent^NDI (9) (10 mol%) + Ni(dme)Cl_2_ (20 mol%) instead of 7	46	3 : 1	>20 : 1
7	^Mes^NDI (10) (10 mol%) + Ni(dme)Cl_2_ (20 mol%) instead of 7	<5	—	—
8	^*i*-Pr^IP (11) (10 mol%) + Ni(dme)Cl_2_ (10 mol%) instead of 7	0	—	—
9	^*i*-Pr^PDI (12) (10 mol%) + Ni(dme)Cl_2_ (10 mol%) instead of 7	0	—	—
10	Ni(dme)Cl_2_ (10 mol%) instead of 7	0	—	—

a Standard reaction conditions: 2 (2.0 equiv.), 3 (0.1 mmol, 1.0 equiv.), (^*i*-Pr^NDI)Ni_2_Cl_2_ (7) (10 mol%), Zn (4.0 equiv.), DMA (0.1 mL), Et_2_O (0.4 mL), 24 h, rt. All yields and selectivities were determined by ^1^H NMR integration using mesitylene as an internal standard.

During reaction development, we found that the inclusion of a nonpolar cosolvent such as Et_2_O was critical for reaction efficiency. With DMA alone, the yield of 4 decreased to 47% (entry 4). Both the identity of the reductant and the solvent appeared to have a significant effect on d.r. but did not impact the high *Z* selectivity at the exocyclic alkene. Under standard conditions, a premetallated dinickel catalyst was used (7). However, the active catalyst could also be assembled *in situ* by stirring free ^*i*-Pr^NDI (8) and Ni(dme)Cl_2_ over Zn, and there was no significant change in reaction outcome (entry 5). Increasing (entry 6) or decreasing (entry 7) the steric profile of the NDI ligand decreased the yield of 4. Finally, Ni(dme)Cl_2_ alone or other mononickel catalysts bearing imine and/or pyridine donors analogous to those found in 7 proved to be ineffective in the reaction (entries 8, 9, and 10).

Following the [2 + 2 + 1]-cycloaddition, methylenedioxolane 4 was deprotected using TFA to form α-hydroxy ketone 5. This two-step sequence can be carried out in a single pot without the need to isolate the intermediate dioxolane (62% yield over the two steps). Alternatively, hydrogenation of methylenedioxolane 4 followed by Lewis acid-catalyzed deprotection yields diol 6 as a 3 : 1 ratio of *anti*/*syn* diastereomers.

α-Hydroxy ketones are found in many biologically active natural products (Fig. 1b).^15^ The benzoin reaction, which involves the Umpolung coupling of two aldehydes *via* an acyl anion intermediate, is one of the most common C–C coupling strategies used to form α-hydroxy ketones. In certain cases, it is possible to carry out selective cross-benzoin reactions by exploiting differences in the steric or electronic properties of the two partners.^16^ However, in cases where the two aldehydes are relatively unbiased, catalytic cross-coupling is generally not feasible. In these cases, a pregenerated acyl anion equivalent, such as a dithiane^17^ or a cyanohydrin,^18^ is required. The drawbacks of this approach are the need for additional synthetic steps and the use of a strong base (for example, an organolithium or metal amide) to generate the requisite anion.

### Substrate scope

**Fig. 2:**
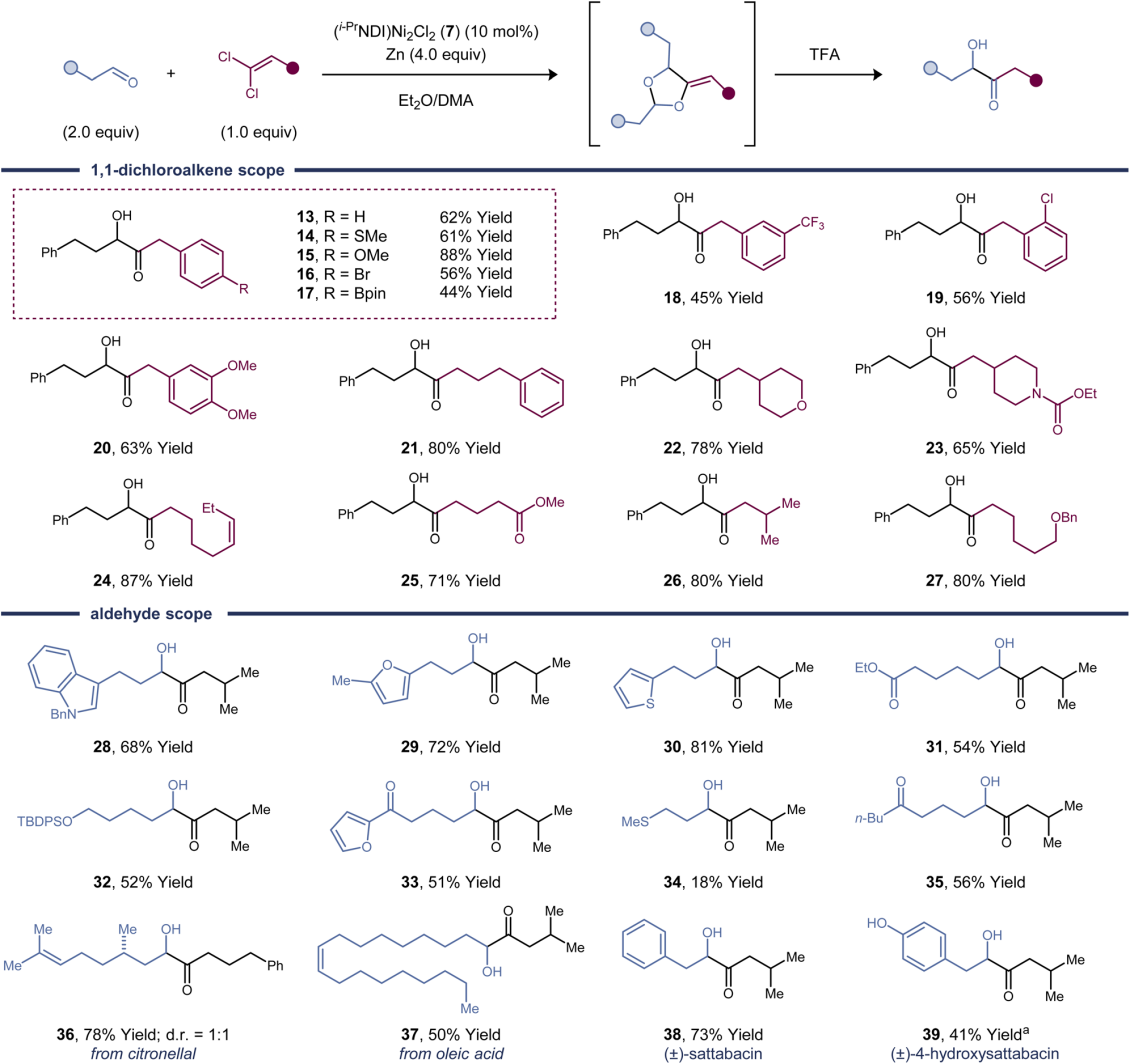
Substrate scope studies. Isolated yields were determined following purification and were averaged over two runs. Standard reaction conditions: dichloroalkene (0.2 mmol, 1.0 equiv.), aldehyde (2.0 equiv.), (^*i*-Pr^NDI)Ni_2_Cl_2_ (7) (10 mol%), Zn (4.0 equiv.), DMA (0.2 mL), Et_2_O (0.8 mL), rt, 24 h; then TFA, 0 °C to rt, 2 h. ^a^Synthesized using 2-(4-((trimethylsilyl)oxy)phenyl)acetaldehyde.

To demonstrate the synthetic utility of this α-hydroxy ketone synthesis, we explored the substrate scope of the one-pot [2 + 2 + 1]-cycloaddition and deprotection sequence (Fig. 2). The reaction proceeds in high yield with a variety of alkyl and aryl substituted 1,1-dichloroalkenes. Aliphatic aldehydes are effective reaction partners, whereas aromatic aldehydes and hindered aliphatic aldehydes containing α-branching are unreactive. A variety of common functional groups and electron-rich heterocycles are compatible with the reaction conditions. Aryl bromides are tolerated (product 16), demonstrating that 1,1-dichloroalkenes undergo activation by the dinickel catalyst at faster rates than C(Ar)–Br bonds. Both a dichloroalkene and an aldehyde containing a pendant alkene proved to be efficient coupling partners (products 24 and 37), and no competing cyclopropanation was observed. Other carbonyl functional groups, such as esters (products 25 and 31), ketones (product 35), and carbamates (product 23), are left untouched in the cycloaddition.

As a synthetic application of this method, two acyloin-containing natural products were prepared.^19^ Phenylacetaldehyde is an effective substrate, forming (±)-sattabacin (38) in 73% yield. Although free phenols are not tolerated, (±)-4-hydroxysattabacin (39) could also be synthesized in 41% yield by utilizing a TMS protecting group. The TMS group is conveniently removed under the same TFA conditions used to deprotect the dioxolane.

### Mechanistic studies

**Fig. 3:**
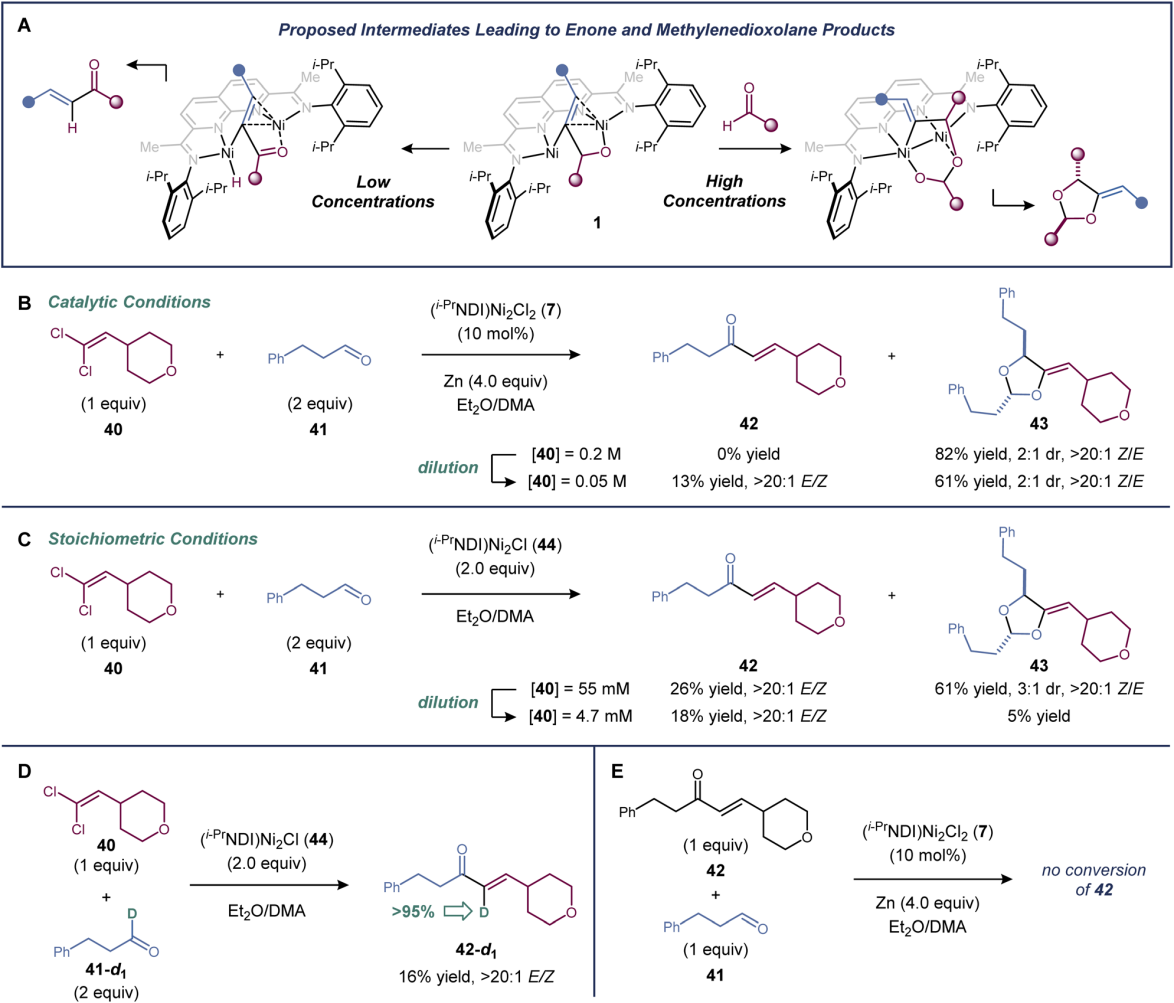
Mechanistic studies. (a) Competing β-hydride elimination and aldehyde migratory pathways. Concentration dependence of β-hydride elimination *vs.* aldehyde migratory insertion under (b) catalytic and (c) stoichiometric conditions. (d) Deuterium labelling experiment tracking the hydrogen undergoing β-hydride elimination. (e) Experiment assessing the intermediacy of enone 42 in the cycloaddition.

There is evidence supporting a metallacyclic intermediate in the [2 + 2 + 1]-cycloaddition similar to that proposed in the dinickel-catalyzed vinylidene–alkene cyclization reaction (Fig. 3a). When the cycloaddition between 1,1-dichloroalkene 40 and aldehyde 41 was carried out at four-fold dilution relative to the standard conditions, the [2 + 2 + 1]-cycloaddition product (43) was formed in a decreased 61% yield, and a minor enone byproduct (42) was generated in 13% yield (Fig. 3b). Presumably, enone formation is due to a competing β-hydride elimination from an intermediate of the general structure 1. The same product profile was observed in experiments carried out under single turnover conditions (Fig. 3c). Reactions between 1,1-dichloroalkene 40, aldehyde 41, and the isolable low-valent form of the catalyst, (^*i*-Pr^NDI)Ni_2_Cl (44), were carried out at two different concentrations. A mixture of 42 and 43 was formed at higher concentrations, but dioxolane formation was suppressed at lower concentrations.

When deuterium-labelled aldehyde 41-*d*_1_ was used, the enone product was labelled exclusively at the α-carbon (>95% deuterium incorporation), confirming the fate of the hydrogen undergoing β-hydride elimination (Fig. 3d). Finally, when enone 42 was subjected to the standard catalytic conditions with an additional equivalent of aldehyde 41, it was not converted to dioxolane 43, indicating that enone 42 is not an intermediate in the [2 + 2 + 1]-cycloaddition (Fig. 3e).

### DFT modeling studies

**Fig. 4:**
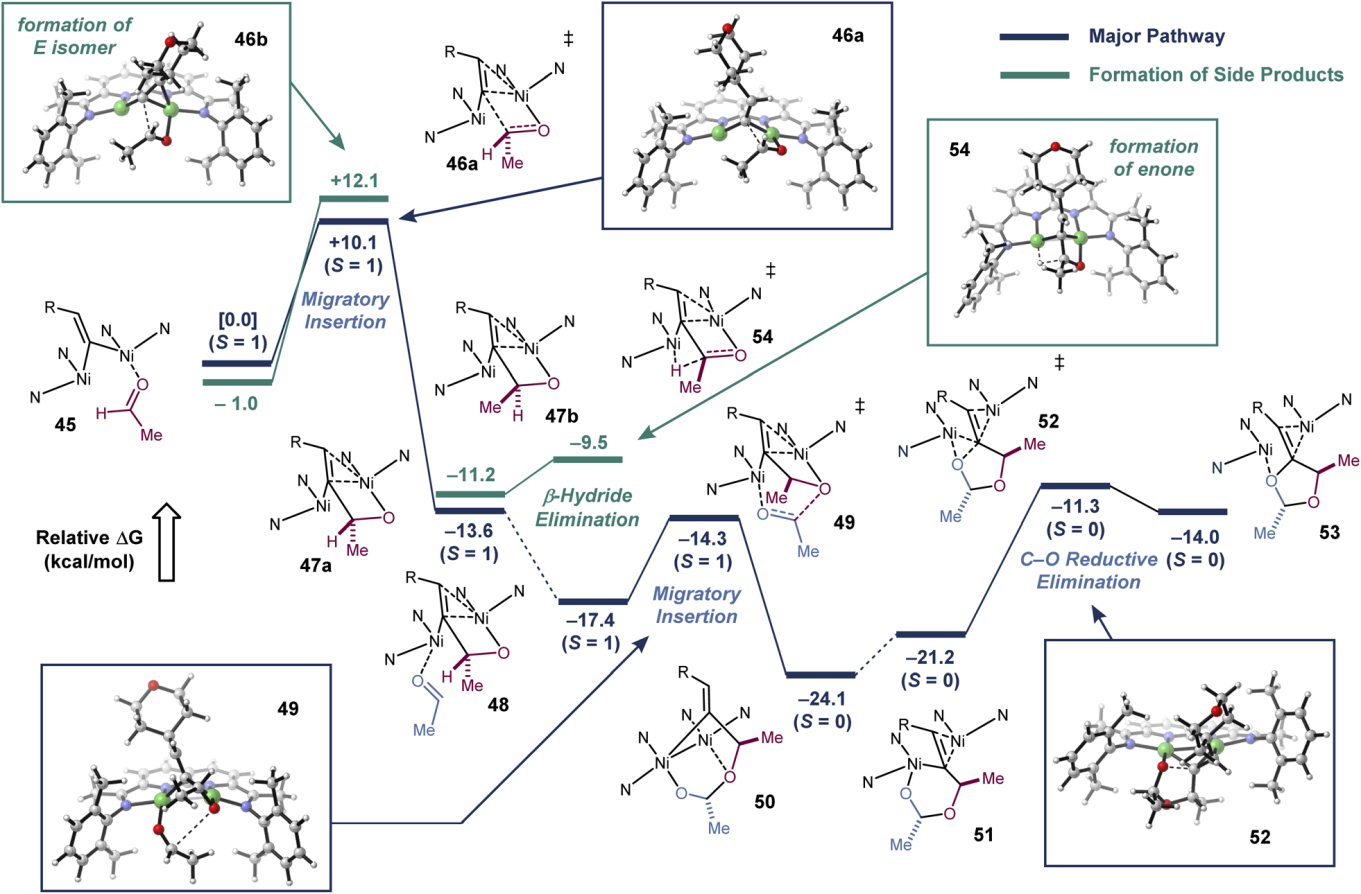
DFT modeling studies. All stationary points are fully optimized at the BP86/6-311g(d,p) level of theory and verified by frequency analysis. *i*-Pr groups on the catalyst were truncated to Me groups. Relative Δ*G* values at 298 K are shown in kcal mol^−1^ and include a dispersion correction (GD3BJ) and an SMD solvent model. The major pathway leading to the [2 + 2 + 1]-cycloaddition product is shown in blue. Competing pathways to form the *E*-isomer of the product and the enone product are shown in green.

With these mechanistic insights in hand, we carried out a series of DFT calculations to further examine the [2 + 2 + 1]-cycloaddition pathway (Fig. 4). As part of these studies, we sought to calculate the competing β-hydride elimination process and explain the origin of the high *Z* selectivity at the exocyclic alkene, which was observed universally across all reaction conditions and substrates that were tested. Stationary points were optimized at the BP86/6-311g(d,p) level of theory, which had previously produced stationary points that corresponded closely to X-ray structures. Single point energy calculations were carried out with an empirical dispersion correction and an SMD model.^20^ In order to compare DFT methods, single point energy calculations were also carried out at the M06L/def2-TZVP level of theory (see ESI†), and no significant changes were observed in the major reaction pathway.

Our model commences with complex 45 (*S* = 1), which is the aldehyde adduct of the putative Ni_2_(μ-vinylidene) intermediate. There are four possible migratory insertion transition states, differing in the orientation of the vinylidene and aldehyde substituents. The most favorable pathway has a barrier of 10.1 kcal mol^−1^ and generates Ni_2_ metallacycle 47a (see ESI† for a description of the higher energy pathways). The calculated structure of 47a is analogous to the metallacycle that we experimentally characterized from the intramolecular addition of a vinylidene to an alkene.^13^

Binding of the second aldehyde to form 48 is exothermic by 3.8 kcal mol^−1^, and the second migratory insertion to form 50 has a low activation barrier of 3.1 kcal mol^−1^. Ogoshi described a mechanistically related head-to-tail coupling of aldehydes in a Tischenko-type reaction.^21^ Metallacycle 50 has a relatively short Ni–Ni distance of 2.5 Å, and NBO analysis suggests that there may be weak Ni–Ni covalent bonding (Wiberg bond index = 0.05). Reductive elimination transition states from metallacycle 50 are prohibitively high in energy. However, dissociation of the ether oxygen and association of the C=C π-bond to one of the Ni atoms would form to an isomeric metallacycle (51) that is similar in energy (+2.9 kcal mol^−1^). Metallacycle 51 has an elongated Ni–Ni distance of 2.9 Å but a notably shortened distance between the C and O undergoing reductive elimination (2.5 Å *vs.* 2.7 Å in 50). Accordingly, C–O reductive elimination from 51 has a barrier of only 9.9 kcal mol^−1^, and this step yields the product adduct 53. Key to this overall process is the ability of the dinuclear active site to form and break weak Ni–Ni interactions in order to traverse different intermediates in the catalytic cycle.

According to the calculated mechanism, the migratory insertion of the first aldehyde is rate-limiting and irreversible. Thus, the *E*/*Z* selectivity of the reaction should be determined in this step. Consistent with the high *Z* selectivity observed experimentally, the migratory insertion transition state that would lead to the *E* product is 2.0 kcal mol^−1^ higher in energy. When this transition state was modeled with *i*-Pr groups on the catalyst instead of the Me truncation, the difference in energy increased to 3.1 kcal mol^−1^ (see ESI† for details).

To model the enone formation under dilute reaction conditions, we searched for β-hydride elimination transition states from intermediate 47. Isomer 47a cannot undergo β–hydride elimination, because the β–H is positioned away from Ni. However, isomer 47b, where the Me and H substituents of the aldehyde are swapped, is poised to undergo a nearly barrierless β-hydride elimination. Finally, we also examined the C–O reductive elimination from 47a, which would form a hypothetical methylene epoxide product. This step has a calculated barrier of 21.4 kcal mol^−1^, making it significantly less favorable than the pathways leading to the methylenedioxolane or to the enone.

## Conclusions

In summary, a dinickel catalyst promotes [2 + 2 + 1]-cycloadditions of two aldehyde equivalents and a vinylidene to form methylenedioxolane products. Unlike the Pauson–Khand reaction, these cycloadditions involve an initial coupling of a vinylidene, which is the C1 component, and an aldehyde to form a metallacyclic intermediate. By avoiding competing reductive elimination and β-hydride elimination processes, it is possible to intercept this intermediate with a second aldehyde and achieve a net three-component cycloaddition. In principle, this mechanism should be compatible with other classes of 2π-systems, and future studies will focus on expanding the scope of partners that can be used.

## Author contributions

Conceptualization, C. U. and A. E. K.; methodology, C. U. and A. E. K.; investigation, A. E. K., M. L., and M. I. B.; writing – original draft, C. U. and A. E. K.; writing – reviewing and editing, C. U., A. E. K., and M. L.; supervision, C. U.; project administration, C. U. and A. E. K.; funding acquisition, C. U.

## Conflicts of interest

There are no conflicts to declare.

## Supplementary Material

SC-013-D2SC02696A-s001
