# Supplementary material for: A dinickel-catalyzed three-component cycloaddition of vinylidenes
Source: Chem Sci. 2022 Sep 8;13(37):11190–6. doi: 10.1039/d2sc02696a (PMC9516891; doi:10.1039/d2sc02696a)
Supplement: SC-013-D2SC02696A-s001 [file SC-013-D2SC02696A-s001.pdf]

Electronic Supplementary Material (ESI) for Chemical Science.
This journal is © The Royal Society of Chemistry 2022

A Dinickel-Catalyzed Three-Component Cycloaddition of Vinylidenes

Annah E. Kalb, Mingxin Liu, Megan I. Bosso, and Christopher Uyeda

Department of Chemistry, Purdue University, West Lafayette, IN 47907, United States

Correspondence: cuyeda@purdue.edu

Supporting Information

1. General Information S2

2. Reaction Optimization Studies S3

3. Synthesis and Characterization of Novel Dichloroalkenes and Aldehydes S9

4. One Pot [2 + 2 + 1]-Cycloadditions/Deprotection and Characterization S11

5. Mechanistic Studies S23

6. NMR Spectral Data S27

7. FT-IR Spectral Data S73

8. DFT Calculations S91

9. References S101

1. General Information

General considerations. All manipulations were carried out using standard Schlenk or glovebox techniques under an atmosphere of N_2. Solvents were dried and degassed by passage through a column of activated alumina and sparging with Ar gas. Deuterated solvents were purchased from Cambridge Isotope Laboratories, Inc. C_6D_6 was degassed using the freeze-pump-thaw method and stored over activated 3 Å molecular sieves prior to use in the glovebox. $CDCl_3$ was stored over activated 3 Å molecular sieves prior to use. Zn powder (325 mesh, 99.9%), Ni(dme)Cl_2 and Ni(COD)$_2$ were purchased from Strem Chemicals, stored under inert atmosphere and used without further purification. Dimethylacetamide (DMA) was stored over 3 Å molecular sieves prior to use. Commercial aldehydes were distilled prior to use. All aldehydes were stored without molecular sieves in the glovebox freezer (–30 °C). All catalytic reactions were run in the glovebox unless otherwise noted. The $^{i\text{-Pr}}$NDI ligand (**8**),[1] $^{c\text{-Pent}}$NDI (**9**),[2] ^{Mes}NDI (**10**),[3] ($^{i\text{-Pr}}$NDI)Ni_2Cl_2 (**7**)[4] and ($^{i\text{-Pr}}$NDI)Ni_2Cl (**44**)[3] were prepared according to previously reported procedures. All other reagents and starting materials were purchased from commercial vendors and used without further purification unless otherwise noted.

Physical methods. ^{1}H NMR, ^{13}C{^{1}H} NMR, ^{19}F NMR, COSY NMR and NOESY NMR spectra were collected at room temperature on a Varian Inova300 with a 5mm 4-nucleus/BB Z-gradient probe, Varian Mercury300 with a 5mm 4-nucleus/BB probe, Bruker AV400 with a 5mm BBFO Z-gradient SmartProbe, a Bruker DRX500 with a 5 mm BBFO Z-gradient ATM probe, or AV800 with a QCI cryoprobe. ^{1}H NMR and ^{13}C{^{1}H} NMR spectra were reported in parts per million relative to tetramethylsilane, using the referenced residual solvent resonances as an internal standard (^{1}H NMR: $CDCl_3$ = 7.26 ppm, C_6D_6 = 7.16 ppm and ^{13}C{^{1}H} NMR: $CDCl_3$ = 77.16 ppm). High-resolution mass data were obtained using a Thermo Scientific LTQ Orbitrap XL mass spectrometer or a Thermo Electron Corporation MAT 95XP-Trap mass spectrometer. IR data were obtained on a Thermo Nicolet Nexus FT-IR spectrometer with an MCT* detector and a KBr beam splitter with a range of 800 – 4500 cm^{-1}.

Computational Methods. DFT calculations were performed with the Gaussian 16 software package. All geometries were fully optimized at the BP86/6-311G(d,p) level of DFT. All stationary points were verified by frequency analysis.

2. Reaction Optimization Studies

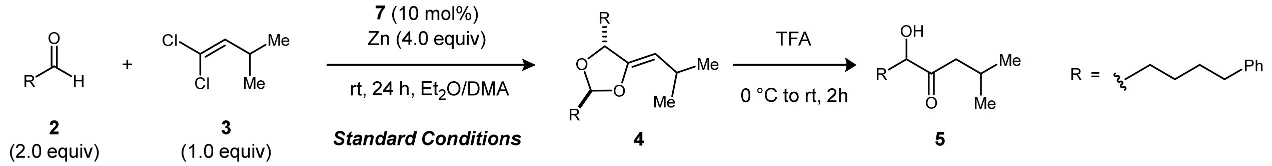

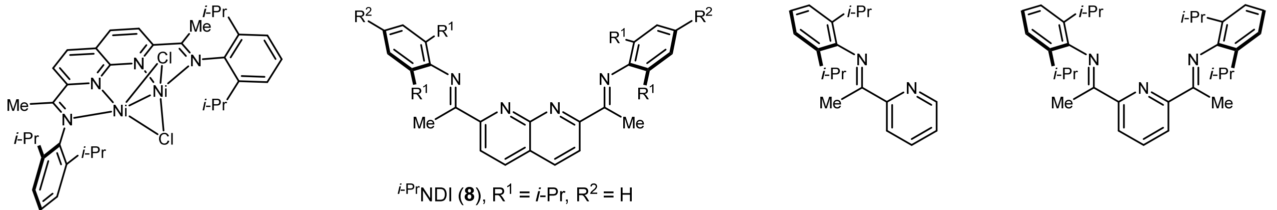

($^{i\text{-Pr}}$NDI)Ni_2Cl_2 (**7**)

$^{i\text{-Pr}}$NDI (**8**), R^1 = *i*-Pr, R^2 = H
$^{c\text{-Pent}}$NDI (**9**), R^1 = R^2 = *c*-Pent
^{Mes}NDI (**10**), R^1 = R^2 = Me

$^{i\text{-Pr}}$IP (**11**)

$^{i\text{-Pr}}$PDI (**12**)

Table S1: Effect of Reaction Parameters

entry	deviations from Standard Conditions	yield (%) (**4**)	anti:syn (**4**)	*Z*/*E* (**4**)
1	none	89	8:1	>20:1
2	Mn instead of Zn	81	14:1	>20:1
3	Cp_2Co instead of Zn	58	1:4	>20:1
4	no Et_2O	47	4:1	>20:1
5	$^{i\text{-Pr}}$NDI (**8**) (10 mol%) + Ni(dme)Cl_2 (20 mol%) instead of **7**	85	6:1	>20:1
6	$^{c\text{-Pent}}$NDI (**9**) (10 mol%) + Ni(dme)Cl_2 (20 mol%) instead of **7**	46	3:1	>20:1
7	^{Mes}NDI (**10**) (10 mol%) + Ni(dme)Cl_2 (20 mol%) instead of **7**	<5	–	–
8	$^{i\text{-Pr}}$IP (**11**) (10 mol%) + Ni(dme)Cl_2 (10 mol%) instead of **7**	0	–	–
9	$^{i\text{-Pr}}$PDI (**12**) (10 mol%) + Ni(dme)Cl_2 (10 mol%) instead of **7**	0	–	–
10	Ni(dme)Cl_2 (10 mol%) instead of **7**	0	–	–

General procedure for entries 1-4. In an N_2-filled glovebox, a 2-dram vial was charged with a magnetic stir bar, ($^{i\text{-Pr}}$NDI)Ni_2Cl_2 (7.2 mg, 0.010 mmol, 10 mol%) and reductant (0.4 mmol, 4.0 equiv). A stock solution of 1,1-dichloro-3-methylbut-1-ene (32363-91-0) (0.1 mmol, 1.0 equiv), 5-phenylpentanal (36884-28-3) (0.2 mmol, 2.0 equiv), mesitylene (0.1 mmol, 1.0 equiv) and DMA (0.1 mL) was added to the catalyst. Et_2O or DMA (entry 5) (0.4 mL) was added, the vial was sealed, and the reaction mixture was stirred (600 rpm) at room temperature. After 24 h, the reaction vial was removed from the glovebox and exposed to air. An aliquot was filtered through a pad of silica (approx. 1 cm) with C_6D_6 and analyzed by ^1H NMR spectroscopy. The yield of the product was determined by ^1H NMR integration against mesitylene.

General procedure for entries 5-10. In an N_2-filled glovebox, a 2-dram vial was charged with a magnetic stir bar, ligand (0.010 mmol, 10 mol%), Ni(dme)Cl_2 (2.2 mg or 4.4 mg, 0.020 mmol or 0.020 mmol, 10 or 20 mol%) and Zn powder (26.2 mg, 0.4 mmol, 4.0 equiv). A stock solution of 1,1-dichloro-3-methylbut-1-ene (32363-91-0) (0.1 mmol, 1.0 equiv), 5-phenylpentanal (36884-28-3) (0.2 mmol, 2.0 equiv), mesitylene (0.1 mmol, 1.0 equiv) and DMA (0.1 mL) was added to the catalyst. Et_2O (0.4 mL) was added, the vial was sealed, and the reaction mixture was stirred (600 rpm) at room temperature. After 24 h, the reaction vial was removed from the glovebox and exposed to air. An aliquot was filtered through a pad of silica (approx. 1 cm) with C_6D_6 and analyzed by ^1H NMR spectroscopy. The yield of the product was determined by ^1H NMR integration against mesitylene.

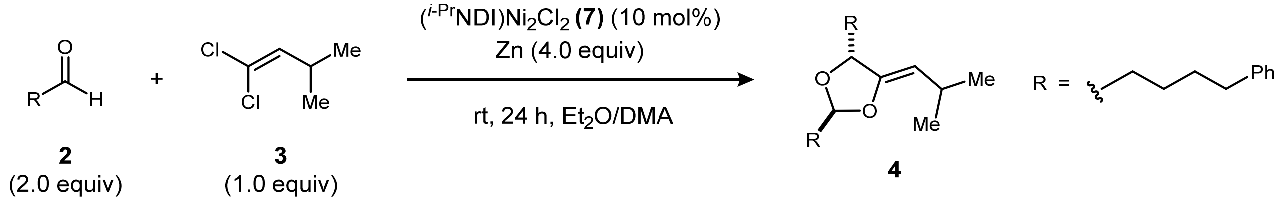

Isolation and Stereochemical Analysis of 4. In an N_2-filled glovebox, a 2-dram vial was charged with a magnetic stir bar, ($^{i\text{-Pr}}$NDI)Ni_2Cl_2 (14.4 mg, 0.02 mmol, 10 mol%), and Zn powder (52.3 mg, 0.8 mmol, 4.0 equiv). DMA (0.2 mL) was added. A solution of 1,1-dichloro-3-methylbut-1-ene (32363-91-0) (0.3 mmol, 1.5 equiv), 5-phenylpentanal (36884-28-3) (0.4 mmol, 2.0 equiv) and Et_2O (0.8 mL) was added. The vial was sealed, and the reaction mixture was stirred (600 rpm) at room temperature. After 24 h, the reaction vial was opened to air and concentrated under a stream of N_2. The remaining residue was loaded directly onto SiO_2 column for purification. Bond connectivity and stereochemistry were determined through analysis of COSY and NOESY NMR.

Purification: SiO_2 column; 1% Et_2O in pentane

TLC: R_f = 0.17 (1% Et_2O in pentane)

^1H NMR (500 MHz, $CDCl_3$) δ 7.33 – 7.26 (m, 4H), 7.23 – 7.16 (m, 6H), **4-*anti*** – 5.30 (t, *J* = 4.8 Hz, 1H), **4-*syn*** – 5.15 (t, *J* = 4.6 Hz, 1H), 4.49 **4-*anti*** – (ddd, *J* = 8.3, 4.1, 1.4 Hz, 1H), **4-*syn*** – 4.41 – 4.34 (m, 1H), **4-*anti*** – 4.02 (dd, *J* = 8.7, 1.4 Hz, 1H), **4-*syn*** – 3.98 (dd, *J* = 8.7, 1.7 Hz, 1H), 2.72 – 2.61 (m, 5H), 1.81 – 1.41 (m, 12H), 1.03 – 0.97 (m, 6H).

$^{13}C\{^1H\}$ NMR (126 MHz, $CDCl_3$) δ 150.7, 142.7, 128.5, 128.4, 125.8, 104.9, 102.7, 77.3, 36.0, 34.3, 31.4, 25.4, 25.2, 23.7, 23.5, 23.4.

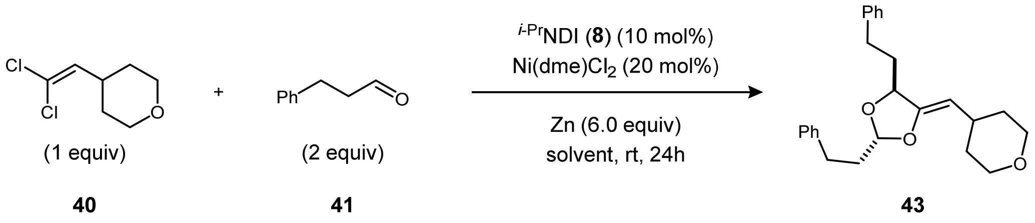

Table S2: Effect of Solvent

entry	Solvent	yield (%) (**43**)	d.r. (anti:syn)	Z/E
1	Et_2O/DMA (4:1)	94	5:1	>99 : 1
2	C_6H_6/DMA (4:1)	74	5:1	>99 : 1
3	THF/DMA (4:1)	84	5:1	>99 : 1
4	MeCN/DMA (4:1)	n.d.	--	--
5	NMP	56	1:3	>99 : 1
6	DMA	43	1:1	>99 : 1
7	Et_2O/DMA (2:3)	60	3:1	>99 : 1

Effect of Solvent. In an N_2-filled glovebox, a 2-dram vial was charged with a magnetic stir bar, $^{i\text{-}Pr}$NDI (**8**) (5.3 mg, 0.010 mmol, 10 mol%), Ni(dme)Cl_2 (4.4 mg, 0.020 mmol, 20mol%), and Zn (39 mg, 0.6 mmol, 6.0 equiv). A stock solution of dichloroalkene **40**[5] (0.1 mmol, 1.0 equiv), aldehyde **41** (104-53-0) (0.2 mmol, 2.0 equiv), mesitylene (0.1 mmol, 1.0 equiv) and DMA (0.1 mL) was added to the same vial. Additional solvent was added (total of 0.5 mL including DMA stock solution), the vial was sealed, and the reaction mixture was stirred (600 rpm) at room temperature. After 24 h, the reaction vial was removed from the glovebox and exposed to air. An aliquot was filtered through a pad of silica (approx. 1 cm) with $CDCl_3$ and analyzed by ^1H NMR spectroscopy. The yield and d.r. of the product was determined by ^1H NMR integration against mesitylene.

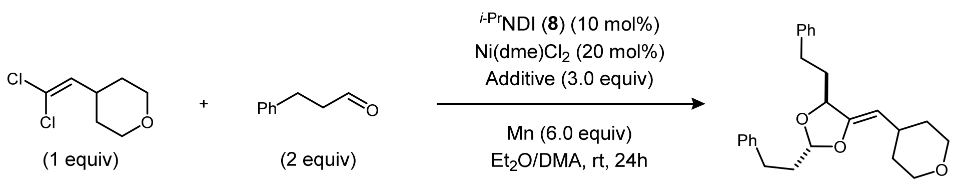

40 **41** **43**

Table S3: Effect of Additives

entry	additive	yield (%) (**43**)	d.r. (anti:syn)	Z/E
1	none	85	6:1	>99 : 1
2	nBu$_4$I	70	1:1	>99 : 1
3	nBu$_4$Br	95	1:3	>99 : 1
4	nBu$_4$Cl	8	--	>99 : 1
5	LiBr	33	1:4	>99 : 1
6	LiCl	48	1:3	>99 : 1
7	NaBr	99	6:1	>99 : 1
8	NaCl	72	9:1	>99 : 1
9	KBr	100	6:1	>99 : 1

Effect of Additives. In an N_2-filled glovebox, a 2-dram vial was charged with a magnetic stir bar, $^{i\text{-Pr}}$NDI (**9**) (5.3 mg, 0.010 mmol, 10 mol%), Ni(dme)Cl_2 (4.4 mg, 0.020 mmol, 20 mol%), additive (0.3 mmol, 3.0 equiv) and Mn (33 mg, 0.6 mmol, 6.0 equiv). A stock solution of dichloroalkene **40**[5] (0.1 mmol, 1.0 equiv), aldehyde **41** (104-53-0) (0.2 mmol, 2.0 equiv), mesitylene (0.1 mmol, 1.0 equiv) and DMA (0.1 mL) was added to the same vial. Et_2O was added (0.4 mL), the vial was sealed, and the reaction mixture was stirred (600 rpm) at room temperature. After 24 h, the reaction vial was removed from the glovebox and exposed to air. An aliquot was filtered through a pad of silica (approx. 1 cm) with $CDCl_3$ and analyzed by ^{1}H NMR spectroscopy. The yield and d.r. of the product was determined by ^{1}H NMR integration against mesitylene.

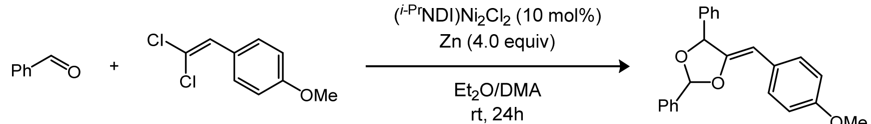

(2 equiv) (1 equiv)

12% conv. 44% conv.

not detected

Reactivity of aryl aldehydes. In an N_2-filled glovebox, a 2-dram vial was charged with a magnetic stir bar, ($^{i\text{-Pr}}$NDI)Ni_2Cl_2 (**7**) (7.2 mg, 0.01 mmol, 10 mol%), and Zn powder (26.1 mg, 0.4 mmol, 4.0 equiv). DMA (0.1 mL) was added. A stock solution of 1,1-dichloroalkene (20.3 mg, 0.1 mmol, 1.0 equiv), benzaldehyde (21.2 mg, 0.2 mmol, 2.0 equiv), 1,3,5-trimethoxybenzene (16.8 mg, 0.10 mmol, 1.0 equiv) and Et_2O (0.4 mL) was added. The vial was sealed, and the reaction mixture was stirred (600 rpm) at room temperature for 24 h. The reaction was removed from the glovebox and exposed to air. An aliquot was removed, filtered through a small pad of silica (approx. 1 cm) eluting with $CDCl_3$, and analyzed by ^{1}H NMR spectroscopy. The 1,3-dioxolane was not detected, and substrate conversions were determined by ^{1}H NMR integration against 1,3,5-trimethoxybenzene.

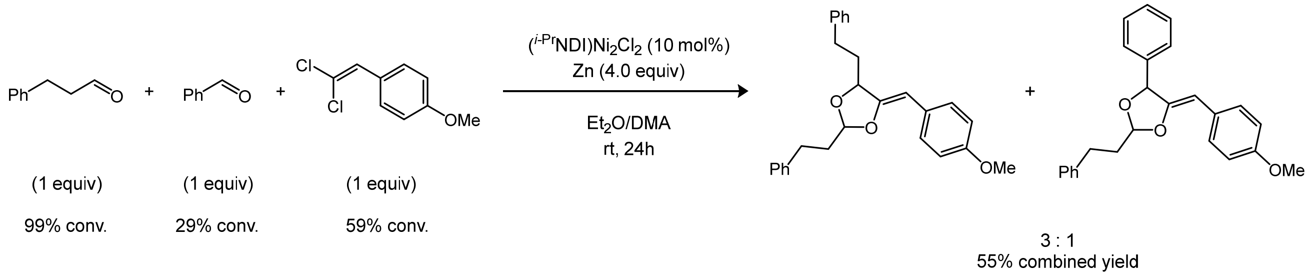

Attempts at forming mixed aldehyde products. In an N_2-filled glovebox, a 2-dram vial was charged with a magnetic stir bar, ($^{i\text{-Pr}}$NDI)Ni_2Cl_2 (**7**) (7.2 mg, 0.01 mmol, 10 mol%), and Zn powder (26.1 mg, 0.4 mmol, 4.0 equiv). DMA (0.1 mL) was added. A stock solution of 1,1-dichloroalkene (20.3 mg, 0.1 mmol, 1.0 equiv), hydrocinnamaldehyde (6.7 mg, 0.1 mmol, 1.0 equiv), benzaldehyde (10.6 mg, 0.1 mmol, 1.0 equiv), mesitylene (12 mg, 0.10 mmol, 1.0 equiv) and Et_2O (0.4 mL) was added. The vial was sealed, and the reaction mixture was stirred (600 rpm) at room temperature for 24 h. The reaction was removed from the glovebox and exposed to air. An aliquot was removed, filtered through a small pad of silica (approx. 1 cm) eluting with $CDCl_3$, and analyzed by ^1H NMR spectroscopy. The product yields and conversion were determined by ^1H NMR integration against mesitylene.

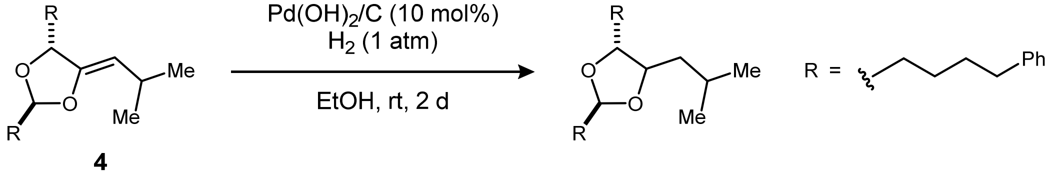

Hydrogenation of compound 4. This reaction was conducted according to a literature procedure with minor modifications[6]. A 25-mL round-bottom flask charged with a stir bar was evacuated and backfilled with a H_2 balloon three times, then 20 wt% $Pd(OH)_2$ on carbon (9.1 mg, 0.013 mmol, 10 mol%) was added to the flask. Compound **4** (51.0 mg, 0.13 mmol, 1.0 equiv) was dissolved in EtOH (2.2 mL, 0.06 M), and the solution was added to the reaction flask at room temperature. The reaction mixture was stirred at room temperature for 2 days. The mixture was filtered through a glass fiber pad, and the filtrate was concentrated. The crude mixture was purified by column chromatography (2% Et_2O in hexanes) to give the hydrogenated product 4-isobutyl-2,5-bis(4-phenylbutyl)-1,3-dioxolane (42.4 mg, 83% yield) as mixture of diastereomers. The mixture was used directly in the next step.

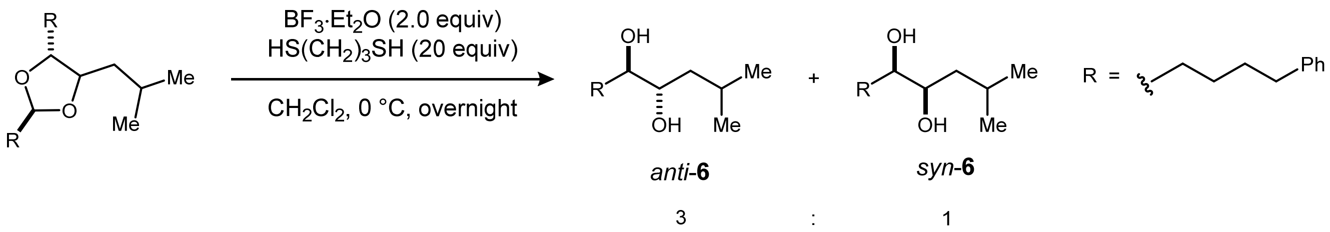

Hydrolysis of 4-isobutyl-2,5-bis(4-phenylbutyl)-1,3-dioxolane. This reaction was conducted according to a literature procedure with minor modifications.[7] A flame-dried 10-mL round-bottom flask charged with a stir bar was evacuated and backfilled with N_2 three times. 4-Isobutyl-2,5-bis(4-phenylbutyl)-1,3-dioxolane (21.2 mg, 0.054 mmol, 1.0 equiv) was dissolved in anhydrous CH_2Cl_2 (0.39

mL, 0.14 M), and the solution was added to the reaction flask at 0 °C. 1,3-Propanedithiol (116.3 mg, 0.11 mL, 1.1 mmol, 20 equiv) and a CH_2Cl_2 solution (1.1 M) of BF_3·Et_2O (0.12 mL, 18.2 mg, 0.13 mmol, 2.4 equiv) were added sequentially at 0 °C. The reaction mixture was stirred at 0 °C overnight. The reaction mixture was then directly loaded onto a silica column for purification (20% EtOAc in hexanes) to give 2-methyl-9-phenylnonane-4,5-diol (**6**) (8.6 mg, 64% yield) as a yellow oil. The diastereomeric ratio and relative configuration were determined by ^{1}H NMR chemical shifts by analogy to compounds reported literature[8–11] (see Figure S85). Further purification on preparative HPLC (Agilent 1260 Infinity II) gave the major *anti* diastereomer as a white solid.

anti-**6**: TLC: R_f = 0.11 (20% EtOAc in hexanes)

^{1}H NMR (400 MHz, $CDCl_3$) δ 7.29 – 7.25 (m, 2H), 7.19 – 7.15 (m, 3H), 3.71 – 3.66 (m, 1H), 3.59 (dt, *J* = 8.3, 4.3 Hz, 1 H), 2.63 (t, *J* = 7.6 Hz, 2 H), 1.81 – 1.75 (m, 1H), 1.79 (d, *J* = 5.1 Hz, 1H), 1.73 (d, *J* = 5.2 Hz, 1 H), 1.69 – 1.62 (m, 2H), 1.61 – 1.51 (m, 1H), 1.49 – 1.33 (m, 4H), 1.16 (ddd, *J* = 14.1, 9.5, 3.0 Hz, 1H), 0.96 (d, *J* = 6.6 Hz, 3H), 0.90 (d, *J* = 6.5 Hz, 3 H).

^{13}C {^{1}H} NMR (201 MHz, $CDCl_3$) δ 142.4, 128.3, 128.2, 125.6, 74.8, 72.5, 40.0, 35.8, 31.4, 31.0, 25.6, 24.4, 23.7, 21.6.

HRMS (APCI) [M+H_2O-H] calc. for $C_{16}H_{26}O_2$=233.1905. found m/z=233.1903.

3. Synthesis and Characterization of Novel Dichloroalkenes and Aldehydes

General Procedure A. Synthesis of 1,1-Dichloroalkenes from Aldehydes

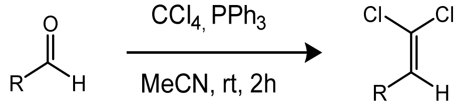

A flame-dried flask, equipped with a stir bar, was charged with PPh_3 (4.0 equiv). The flask was placed under an N_2 atmosphere, and MeCN (0.5 M) was added to create a slurry. In a separate flask was added aldehyde (1.0 equiv) and CCl_4 (2.0 equiv). The aldehyde/CCl_4 solution was added dropwise to the slurry over 5 min and the reaction was stirred for 2 h. Upon completion, the solution was diluted with Et_2O, washed with H_2O (x2) then brine. The organic layer was dried with Na_2SO_4 and concentrated to dryness. Triphenylphosphine oxide was filtered off and washed with Et_2O. The crude material was purified by flash chromatography.

Note: reactions larger than 20 mmol were cooled to 0 °C, and addition of the aldehyde solution was done over 30 min.

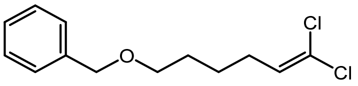

(((6,6-dichlorohex-5-en-1-yl)oxy)methyl)benzene (S1). The reaction was conducted with 5-(benzyloxy)pentanal[12] (1.24 g, 6.4 mmol), CCl_4 (1.24 mL, 12.8 mmol), and PPh_3 (6.7g, 25.6mmol) in MeCN (13 mL) without modification from general procedure A to provide **S1** as a clear, colorless oil.

Purification: SiO_2 column; 5% EtOAc/Hexanes

557mg, 33% Yield.

^1H NMR (500 MHz, $CDCl_3$) δ 7.39 – 7.25 (m, 5H), 5.86 (td, *J* = 7.4, 1.7 Hz, 1H), 4.51 (s, 2H), 3.49 (t, *J* = 6.3 Hz, 2H), 2.20 (q, *J* = 7.4 Hz, 2H), 1.70 – 1.58 (m, 2H), 1.58 – 1.48 (m, 2H).

$^{13}C\{^1H\}$ NMR (126 MHz, $CDCl_3$) δ 138.7, 129.9, 128.5, 127.8, 127.7, 120.2, 73.1, 70.0, 29.5, 29.3, 25.0.

HRMS (APCI) [M+H] calc. for $C_{13}H_{17}Cl_2O$: m/z=259.0651, found: m/z=259.0645

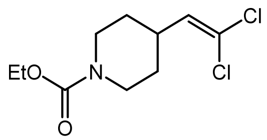

ethyl 4-(2,2-dichlorovinyl)piperidine-1-carboxylate (S2). The reaction was conducted with ethyl 4-formylpiperidine-1-carboxylate[13] (99658-58-9) (5.09 g, 27.5 mmol), CCl_4 (5.3 mL, 55.0 mmol), and PPh_3 (29.1 g, 110.0 mmol) in MeCN (13 mL) without modification from general procedure A to provide **S2** as a clear, colorless oil that solidified upon standing.

Purification: SiO_2 column; 10% EtOAc in Hexanes

3.578 g, 14% Yield

^1H NMR (500 MHz, $CDCl_3$) δ 5.69 (dd, *J* = 9.0, 1.2 Hz, 1H), 4.18 – 4.05 (m, 4H), 2.82 (t, *J* = 12.7 Hz, 2H), 2.60 – 2.48 (m, 1H), 1.74 – 1.67 (m, 2H), 1.37 – 1.27 (m, 2H), 1.25 (td, *J* = 7.1, 1.3 Hz, 3H).

$^{13}C\{^1H\}$ NMR (126 MHz, $CDCl_3$) δ 155.6, 133.0, 120.4, 61.5, 43.5, 37.4, 30.6, 14.8.

HRMS (APCI) [M+H] calc. for $C_{10}H_{16}Cl_2NO_2$: m/z=252.0553, found: m/z=252.0555

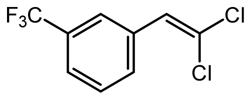

1-(2,2-dichlorovinyl)-3-(trifluoromethyl)benzene (S3). The reaction was conducted with 3-(trifluoromethyl)benzaldehyde (454-89-7) (1.00 g, 5.74 mmol), CCl_4 (1.11 mL, 11.5 mmol), and PPh_3 (6.02 g, 23.0 mmol) in MeCN (12 mL) without modification from general procedure A to provide **S3** as a clear, colorless oil.

Purification: SiO_2 column; 100% Hexanes

390 mg, 28% Yield

^{1}H NMR (500 MHz, $CDCl_3$) δ 7.79 (s, 1H), 7.71 (dd, *J* = 8.0, 1.9 Hz, 1H), 7.58 (d, *J* = 7.8 Hz, 1H), 7.50 (t, *J* = 7.8 Hz, 1H), 6.89 (s, 1H).

^{19}F NMR (470 MHz, $CDCl_3$) δ -64.39.

^{13}C{^{1}H} NMR (126 MHz, $CDCl_3$) δ 134.3, 131.9, 131.2 (q, $^2J_{CF}$ = 32.4 Hz), 129.2, 127.4, 125.6 (q, $^3J_{CF}$ = 4.0 Hz), 125.2 (q, $^3J_{CF}$ = 3.7 Hz), 124.0 (q, $^1J_{CF}$ = 272.4 Hz), 123.3.

HRMS (APCI) [M-] calc. for $C_9H_5Cl_2F_3$: m/z=239.9715, found: m/z=239.9710

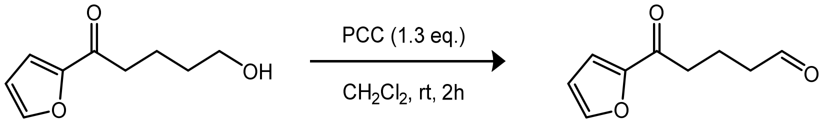

5-(furan-2-yl)-5-oxopentanal (S4). 1-(furan-2-yl)-5-hydroxypentan-1-one[14] (1.40 g, 8.30 mmol, 1 equiv) was dissolved in dry CH_2Cl_2 (14 mL, 0.6 M), in a flame-dried round bottom flask containing a stir bar. PCC (2.35 g, 10.9 mmol, 1.3 equiv) and celite (800 mg) were added to the round bottom flask containing the alcohol. The reaction was stirred at rt under N_2 for 2 h. Et_2O (15 mL) was added to the reaction mixture, and the mixture was filtered through celite. The filtrate was evaporated under reduced pressure. The crude product was purified using column chromatography to provide **S4** as an off-white solid. (Note: the aldehyde decomposes rapidly under air at ambient conditions. Once purified, the aldehyde was stored in a glovebox freezer.)

Purification: SiO_2, 20% EtOAc in pentane

118 mg, 8.5% Yield.

MP: 43-45 °C

^{1}H NMR (500 MHz, $CDCl_3$) δ 9.78 (s, 1H), 7.56 (dd, *J* = 1.8, 0.8 Hz, 1H), 7.18 (d, *J* = 3.6 Hz, 1H), 6.52 (dd, *J* = 3.6, 1.7 Hz, 1H), 2.88 (t, *J* = 7.2 Hz, 2H), 2.56 (td, *J* = 7.1, 1.3 Hz, 2H), 2.04 (p, *J* = 7.1 Hz, 2H).

^{13}C{^{1}H} NMR (126 MHz, $CDCl_3$) δ 201.9, 188.6, 152.7, 146.5, 117.2, 112.4, 43.1, 37.2, 16.6.

HRMS (APCI) [M+H] calc. for $C_9H_{11}O_3$: m/z=167.0708, found: m/z=167.0702

4. One Pot [2 + 2 + 1]-Cycloadditions/Deprotection and Characterization

General Procedure B (reactions with alkyl-substituted dichloroalkenes). In an N_2-filled glovebox, a 2-dram vial was charged with a magnetic stir bar, ($^{i\text{-Pr}}$NDI)Ni_2Cl_2 (**7**) (14.4 mg, 0.02 mmol, 10 mol%), and Zn powder (52.3 mg, 0.8 mmol, 4.0 equiv). DMA (0.2 mL, 2.0 M) was added. Immediately following DMA addition, a solution of 1,1-dichloroalkene (0.2 mmol, 1.0 equiv), aldehyde (0.4 mmol, 2.0 equiv) and Et_2O (0.8 mL, 0.25 M) was added. The vial was sealed with a Teflon cap, and the reaction mixture was stirred (600 rpm) at room temperature. After 24 h, the reaction vial was removed from the glovebox and opened to air.

The vial was placed in an ice bath, and a 33 vol% solution of trifluoroacetic acid (156 μL, 2.0 mmol, 10 equiv) in water was added slowly to the reaction mixture. The reaction was warmed to room temperature and stirred for 2 h. The aqueous layer was removed, and the organic layer was diluted with Et_2O (2 mL). The organic layer was washed with sat. $NaHCO_3$ (2 x 4 mL), washed with brine (1 x 4 mL), dried over Na_2SO_4, and concentrated to dryness. The crude product was redissolved in a minimal amount of CH_2Cl_2 and loaded onto a SiO_2 column for purification.

General Procedure C (reactions with aryl-substituted dichloroalkenes). In an N_2-filled glovebox, a 2-dram vial was charged with a magnetic stir bar, ($^{i\text{Pr}}$NDI)Ni_2Cl_2 (**7**) (14.4 mg, 0.02 mmol, 10 mol%), and Zn powder (52.3 mg, 0.8 mmol, 4.0 equiv). DMA (0.2 mL, 2.0 M) was added. Immediately following DMA addition, a solution of 1,1-dichloroalkene (0.2 mmol, 1.0 equiv), aldehyde (0.4 mmol, 2.0 equiv) and Et_2O (0.8 mL, 0.25 M) was added. The vial was sealed with a Teflon cap, and the reaction mixture was stirred (600 rpm) at room temperature. After 24 h, the reaction vial was removed from the glovebox and opened to air.

The vial was placed in an ice bath and trifluoroacetic acid (780 μL, 10.0 mmol, 50 equiv) was added slowly to the reaction mixture. The reaction was warmed to room temperature and stirred for 2 h. The reaction was diluted with Et_2O (2 mL) and washed with H_2O (1 x 4 mL). The organic layer was washed with sat. $NaHCO_3$ (2 x 4mL), washed with brine (1 x 4 mL), dried over Na_2SO_4, and concentrated to dryness. The crude product was redissolved in a minimal amount of CH_2Cl_2 and loaded onto a SiO_2 column for purification.

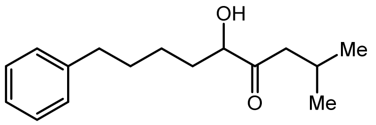

5-hydroxy-2-methyl-9-phenylnonan-4-one (5). The reaction was conducted with 1,1-dichloro-3-methylbut-1-ene (32363-91-0) (27.8 mg, 0.2 mmol) and 5-phenylpentanal (36884-28-3) (65.0 mg, 0.4 mmol) without modification from general procedure B to provide **5** as a light yellow oil.

Purification: SiO_2 column; 15% Et_2O in pentane

30.9 mg, 62% Yield.

TLC: R_f = 0.12 (15% Et_2O in pentane)

^{1}H NMR (500 MHz, $CDCl_3$) δ 7.31 – 7.24 (m, 2H), 7.21 – 7.15 (m, 3H), 4.12 (dt, *J* = 7.9, 4.4 Hz, 1H), 3.50 (d, *J* = 4.9 Hz, 1H), 2.63 (t, *J* = 7.7 Hz, 2H), 2.31 (d, *J* = 6.9 Hz, 2H), 2.19 (dp, *J* = 13.5, 6.7 Hz, 1H), 1.84 (ddt, *J* = 11.2, 8.1, 3.7 Hz, 1H), 1.76 – 1.57 (m, 2H), 1.57 – 1.46 (m, 2H), 1.49 – 1.37 (m, 1H), 0.92 (d, *J* = 6.6 Hz, 6H).

^{13}C{^{1}H} NMR (126 MHz, $CDCl_3$) δ 212.1, 142.5, 128.5, 128.5, 125.9, 76.7, 46.9, 35.9, 33.6, 31.4, 24.7, 24.7, 22.7, 22.7.

HRMS (APCI) [M-H] calc. for $C_{16}H_{23}O_2$: m/z= 247.1698, found: m/z= 247.17053

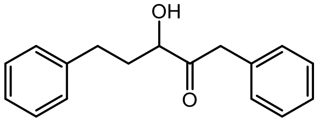

3-hydroxy-1,5-diphenylpentan-2-one (13). The reaction was conducted with (2,2-dichlorovinyl)benzene[15] (698-88-4) (34.6 mg, 0.200 mmol) and hydrocinnamaldehyde (104-53-0) (53.7 mg, 0.4 mmol) without modification from general procedure C to provide **13** as a light yellow oil.

Run 1: 33.7 mg, 66% Yield. Run 2: 28.9 mg, 57% Yield

Purification: SiO_2 column; 20% Et_2O in pentane

TLC: R_f = 0.12 (25% Et_2O in pentane)

^1H NMR (400 MHz, $CDCl_3$) δ 7.37 – 7.27 (m, 5H), 7.25 – 7.12 (m, 5H), 4.28 (ddd, J = 8.3, 5.0, 3.5 Hz, 1H), 3.74 (s, 2H), 3.42 (d, *J* = 5.1 Hz, 1H), 2.86 – 2.64 (m, 2H), 2.19 (dddd, *J* = 13.8, 9.6, 7.1, 3.5 Hz, 1H), 1.87 (dddd, *J* = 13.6, 9.4, 8.1, 5.2 Hz, 1H).

^{13}C {^1H} NMR (101 MHz, $CDCl_3$) δ 209.7, 141.2, 133.0, 129.5, 129.0, 128.8, 128.7, 127.5, 126.3, 75.4, 45.0, 35.6, 31.2.

HRMS (APCI) [M-H] calc. for $C_{17}H_{17}O_2$: m/z=253.1223, found: m/z=253.1226

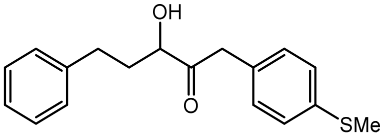

3-hydroxy-1-(4-(methylthio)phenyl)-5-phenylpentan-2-one (14). The reaction was conducted with (4-(2,2-dichlorovinyl)phenyl)(methyl)sulfane[3] (43.8 mg, 0.2 mmol) and hydrocinnamaldehyde (104-53-0) (53.7 mg, 0.4 mmol) without modification from general procedure C to provide **14** as a light yellow oil.

Run 1: 40.1 mg, 67% Yield. Run 2: 32.4 mg, 54% Yield

Purification: SiO_2 column; 30% Et_2O in pentane

TLC: R_f = 0.15 (40% Et_2O in pentane)

^1H NMR (500 MHz, $CDCl_3$) δ 7.30 (t, *J* = 7.5 Hz, 2H), 7.24 – 7.15 (m, 5H), 7.06 (d, *J* = 8.2 Hz, 2H), 4.26 (dd, *J* = 8.1, 3.5 Hz, 1H), 3.69 (d, *J* = 1.8 Hz, 2H), 3.40 (s, 1H), 2.83 – 2.62 (m, 2H), 2.47 (s, 1H), 2.18 (dddd, *J* = 13.4, 10.1, 7.1, 3.5 Hz, 1H), 1.86 (dtd, *J* = 13.8, 8.7, 5.1 Hz, 1H).

^{13}C NMR (126 MHz, $CDCl_3$) δ 209.7, 141.1, 137.8, 130.0, 129.7, 128.8, 128.7, 127.1, 126.3, 75.4, 44.4, 35.5, 31.2, 16.0.

HRMS (APCI) [M-H] calc. for $C_{18}H_{19}O_2S$: m/z=299.1100, found: m/z=299.1101

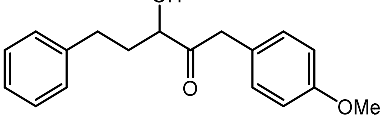

3-hydroxy-1-(4-methoxyphenyl)-5-phenylpentan-2-one (15). The reaction was conducted with 1-(2,2-dichlorovinyl)-4-methoxybenzene[9] (40.4 mg, 0.2 mmol) and hydrocinnamaldehyde (104-53-0) (53.7 mg, 0.4 mmol) without modification from general procedure C to provide **15** as a light yellow oil.

Run 1: 46.0 mg, 81% Yield. Run 2: 51.5 mg, 94%

Purification: SiO_2 column; 35% Et_2O in pentane

TLC: R_f = 0.19 (40% Et_2O in pentane)

^1H NMR (400 MHz, $CDCl_3$) δ 7.35 – 7.14 (m, 6H), 7.06 (d, *J* = 8.7 Hz, 2H), 6.84 (d, J = 8.9 Hz, 2H), 4.26 (dd, J = 8.1, 3.5 Hz, 1H), 3.79 (s, 3H), 3.67 (s, 2H), 2.83 – 2.60 (m, 2H), 2.18 (dddd, J = 13.4, 9.6, 7.1, 3.5 Hz, 1H), 1.93 – 1.79 (m, 1H).

^{13}C {^1H} NMR (101 MHz, $CDCl_3$) δ 210.1, 159.0, 141.2, 130.6, 128.8, 128.7, 126.3, 125.0, 114.4, 75.2, 55.4, 44.2, 35.6, 31.2.

HRMS (APCI) [M-H] calc. for $C_{18}H_{19}O_3$: m/z=283.1329, found: m/z=283.1330

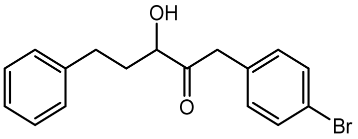

1-(4-bromophenyl)-3-hydroxy-5-phenylpentan-2-one (16). The reaction was conducted with 1-bromo-4-(2,2-dichlorovinyl)benzene[9] (50.4 mg, 0.2 mmol) and hydrocinnamaldehyde (104-53-0) (53.7 mg, 0.4 mmol) without modification from general procedure C to provide **16** as a light yellow oil.

Run 1: 36.3 mg, 54% Yield. Run 2: 37.7 mg, 57% Yield.

Purification: SiO_2 column; 25% Et_2O in pentane

TLC: R_f = 0.08 (25% Et_2O in pentane)

^1H NMR (400 MHz, $CDCl_3$) δ 7.44 (d, *J* = 8.5 Hz, 2H), 7.35 – 7.27 (m, 2H), 7.25 – 7.14 (m, 3H), 7.01 (d, *J* = 8.3 Hz, 2H), 4.25 (d, *J* = 8.2 Hz, 1H), 3.68 (s, 2H), 3.39 (s, 1H), 2.86 – 2.66 (m, 2H), 2.18 (dddd, *J* = 13.9, 9.2, 7.3, 3.5 Hz, 1H), 1.95 – 1.80 (m, 1H).

^{13}C {^1H} NMR (101 MHz, $CDCl_3$) δ 209.2, 141.0, 132.0, 131.9, 131.3, 128.8, 128.7, 126.4, 121.6, 75.5, 44.2, 35.5, 31.2.

HRMS (APCI) [M-H] calc. for $C_{17}H_{16}BrO_2$: m/z=331.0328, found: m/z=331.0329

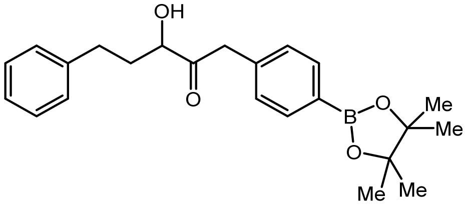

3-hydroxy-5-phenyl-1-(4-(4,4,5,5-tetramethyl-1,3,2-dioxaborolan-2-yl)phenyl)pentan-2-one (17). The reaction was conducted with 2-(4-(2,2-dichlorovinyl)phenyl)-4,4,5,5-tetramethyl-1,3,2-dioxaborolane[3] (59.8 mg, 0.2 mmol) and hydrocinnamaldehyde (104-53-0) (53.7 mg, 0.4 mmol) without modification from general procedure C to provide **17** as a light yellow oil.

Run 1: 36.0 mg, 47% Yield. Run 2: 31.5 mg, 41% Yield.

Purification: SiO_2 column: 25-30% Et_2O in pentane

TLC: R_f = 0.10 (25% Et_2O in pentane)

^1H NMR (400 MHz, $CDCl_3$) δ 7.79 – 7.73 (m, 2H), 7.33 – 7.26 (m, 2H), 7.24 – 7.11 (m, 5H), 4.25 (ddd, *J* = 8.3, 4.9, 3.5 Hz, 1H), 3.74 (s, 2H), 3.40 (d, *J* = 5.0 Hz, 1H), 2.83 – 2.63 (m, 2H), 2.17 (dddd, *J* = 13.9, 9.6, 7.2, 3.5 Hz, 1H), 1.93 – 1.78 (m, 1H), 1.34 (s, 12H).

^{13}C{^1H} NMR (101 MHz, $CDCl_3$) δ 209.5, 141.1, 136.1, 135.4, 128.9, 128.8, 128.7, 126.3, 84.0, 75.4, 45.3, 35.5, 31.2, 25.0.

HRMS (APCI) [M+H] calc. for $C_{23}H_{30}B^{10}O_4$: m/z=380.2268, found: m/z=280.2259

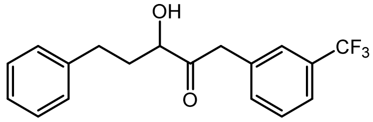

3-hydroxy-5-phenyl-1-(3-(trifluoromethyl)phenyl)pentan-2-one (18). The reaction was conducted with 1-(2,2-dichlorovinyl)-3-(trifluoromethyl)benzene **S3** (48.2 mg, 0.2 mmol) and hydrocinnamaldehyde (104-53-0) (53.7 mg, 0.4 mmol) without modification from general procedure C to provide **18** as a light yellow oil.

Run 1: 29.3 mg, 45% Yield. Run 2: 29.3 mg, 45% Yield

Purification: SiO_2 column; 25% Et_2O in pentane

TLC: R_f = 0.09 (25% Et_2O in pentane)

^{1}H NMR (800 MHz, $CDCl_3$) δ 7.54 (d, *J* = 7.8 Hz, 1H), 7.44 (t, *J* = 7.9, 2.6 Hz, 1H), 7.40 (s, 1H), 7.35 – 7.27 (m, 3H), 7.27 – 7.18 (m, 3H), 4.28 (ddd, *J* = 8.3, 5.0, 3.4 Hz, 1H), 3.78 (s, 2H), 3.36 (dd, *J* = 5.0, 1.7 Hz, 1H), 2.84 – 2.78 (m, 1H), 2.78 – 2.71 (m, 1H), 2.21 (dddt, *J* = 13.9, 9.0, 7.1, 3.3 Hz, 1H), 1.91 (dtd, *J* = 14.0, 8.6, 5.3 Hz, 1H).

^{19}F NMR (376 MHz, $CDCl_3$) δ -64.17.

^{13}C NMR (201 MHz, $CDCl_3$) δ 208.9, 140.9, 133.9, 133.0, 131.2 (q, $^2J_{CF}$ = 32.2 Hz), 129.3, 128.8, 128.8, 126.5, 126.4 (q, $^3J_{CF}$ = 3.7 Hz), 124.4 (q, $^3J_{CF}$ = 3.8 Hz), 124.1 (q, $^1J_{CF}$ = 272.4 Hz), 75.7, 44.4, 35.5, 31.2.

HRMS (ESI) [M+Na] calc. for $C_{18}H_{17}F_3O_2Na$: m/z=345.1073, found: m/z=345.1077

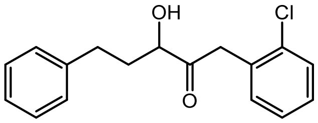

1-(2-chlorophenyl)-3-hydroxy-5-phenylpentan-2-one (19). The reaction was conducted with 1-chloro-2-(2,2-dichlorovinyl)benzen[3] (41.5 mg, 0.2 mmol) and hydrocinnamaldehyde (104-53-0) (53.7 mg, 0.4 mmol) without modification from general procedure C to provide **19** as an off-white solid.

Run 1: 33.7 mg, 58% Yield. Run 2: 31.4 mg, 54% Yield

Purification: SiO_2 column; 25% Et_2O in pentane

TLC: R_f = 0.10 (25% Et_2O in pentane)

MP: 55-57 °C

^{1}H NMR (400 MHz, $CDCl_3$) δ 7.41 – 7.35 (m, 1H), 7.34 – 7.28 (m, 2H), 7.27 – 7.07 (m, 6H), 4.33 (ddd, *J* = 8.4, 5.0, 3.5 Hz, 1H), 3.99 – 3.80 (m, 2H), 3.40 (d, *J* = 5.0 Hz, 1H), 2.89 – 2.69 (m, 2H), 2.25 (dddd, *J* = 13.9, 9.1, 7.7, 3.5 Hz, 1H), 1.93 (dtd, *J* = 14.3, 8.4, 6.1 Hz, 1H).

^{13}C {^{1}H} NMR (101 MHz, $CDCl_3$) δ 208.6, 141.2, 134.4, 131.9, 131.7, 129.7, 129.1, 128.8, 128.7, 127.2, 126.3, 75.7, 42.9, 35.7, 31.3.

HRMS (APCI) [M-H] calc. for $C_{17}H_{16}ClO_2$: m/z=287.0833, found: m/z=287.0838

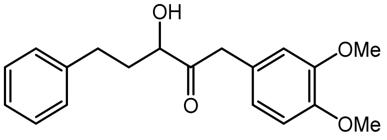

1-(3,4-dimethoxyphenyl)-3-hydroxy-5-phenylpentan-2-one (20). The reaction was conducted with 4-(2,2-dichlorovinyl)-1,2-dimethoxybenzene[3] (60561-55-9) (46.6 mg, 0.2 mmol) and hydrocinnamaldehyde (104-53-0) (53.7 mg, 0.4 mmol) without modification from general procedure C to provide **20** as a light-yellow oil.

Run 1: 38.8 mg, 62% Yield. Run 2: 39.7 mg, 63% Yield

Purification: SiO_2 column; 45% Et_2O in pentane

TLC: R_f= 0.12 in 45% Et_2O in pentane

^1H NMR (500 MHz, $CDCl_3$) δ 7.29 (t, *J* = 7.5 Hz, 2H), 7.21 (t, *J* = 7.3 Hz, 1H), 7.20 – 7.15 (m, 2H), 6.80 (d, *J* = 8.1 Hz, 1H), 6.71 – 6.64 (m, 2H), 4.27 (d, *J* = 8.0 Hz, 1H), 3.86 (s, 3H), 3.84 (s, 3H), 3.67 (s, 2H), 3.40 (s, 1H), 2.83 – 2.74 (m, 1H), 2.74 – 2.65 (m, 1H), 2.18 (dddd, *J* = 13.4, 10.4, 7.1, 3.5 Hz, 1H), 1.91 – 1.80 (m, 1H).

^{13}C {^1H} NMR (101 MHz, $CDCl_3$) δ 210.1, 149.2, 148.5, 141.1, 128.7, 128.7, 126.3, 125.4, 121.7, 112.5, 111.5, 75.2, 56.0, 44.6, 35.6, 31.2.

HRMS (APCI) [M-H] calc. for $C_{19}H_{21}O_4$: m/z=313.1434, found: m/z=313.1430

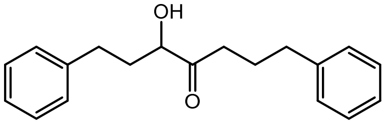

3-hydroxy-1,7-diphenylheptan-4-one (21). The reaction was conducted with (4,4-dichlorobut-3-en-1-yl)benzene[9] (40.2 mg, 0.2 mmol) and hydrocinnamaldehyde (104-53-0) (53.7 mg, 0.4 mmol) without modification from general procedure B to provide **21** as a light-yellow oil.

Run 1: 45.6 mg, 81% Yield. Run 2: 44.6 mg, 79% Yield.

Purification: SiO_2 column; 20% Et_2O in pentane

TLC: R_f = 0.15 (25% Et_2O in pentane)

^1H NMR (400 MHz, $CDCl_3$) δ 7.34 – 7.26 (m, 4H), 7.24 – 7.12 (m, 6H), 4.12 (d, *J* = 8.1 Hz, 1H), 3.54 (s, 1H), 2.83 – 2.65 (m, 2H), 2.62 (t, *J* = 7.5 Hz, 2H), 2.52 – 2.32 (m, 2H), 2.09 (dddd, *J* = 13.8, 9.7, 7.1, 3.5 Hz, 1H), 2.02 – 1.86 (m, 2H), 1.77 (dddd, *J* = 13.7, 9.4, 8.4, 5.2 Hz, 1H).

^{13}C {^1H} NMR (101 MHz, $CDCl_3$) δ 212.0, 141.2, 128.7, 128.7, 128.6, 128.6, 126.3, 75.8, 37.0, 35.7, 35.1, 31.3, 25.1.

HRMS (APCI) [M+H] calc. for $C_{19}H_{23}O_2$: m/z=283.1693, found: m/z=283.1686

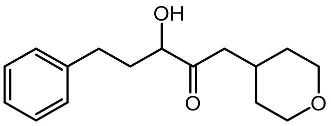

3-hydroxy-5-phenyl-1-(tetrahydro-2H-pyran-4-yl)pentan-2-one (22). The reaction was conducted with 4-(2,2-dichlorovinyl)tetrahydro-2H-pyran[5] (36.2 mg, 0.2 mmol) and hydrocinnamaldehyde (104-53-0) (53.7 mg, 0.4 mmol) without modification from general procedure B to provide **22** as a light yellow oil.

Run 1: 45.6 mg, 87% Yield; Run 2: 36.1 mg, 69% Yield

Purification: SiO_2 column; 50% Et_2O in pentane

TLC: R_f = 0.11 (50% Et_2O in pentane)

^{1}H NMR (400 MHz, $CDCl_3$) δ 7.35 – 7.27 (m, 2H), 7.24 – 7.17 (m, 3H), 4.11 (ddd, *J* = 8.3, 4.8, 3.4 Hz, 1H), 3.97 – 3.88 (m, 2H), 3.51 (d, *J* = 4.9 Hz, 1H), 3.40 (td, *J* = 11.8, 2.2 Hz, 2H), 2.86 – 2.66 (m, 2H), 2.43 – 2.27 (m, 2H), 2.19 – 2.03 (m, 1H), 1.79 (dtd, *J* = 14.1, 8.8, 5.4 Hz, 1H), 1.62 – 1.52 (m, 2H), 1.36 – 1.19 (m, 2H).

$^{13}C\{^1H\}$ NMR (101 MHz, $CDCl_3$) δ 211.2, 141.2, 128.7, 128.7, 126.4, 76.2, 67.8, 44.9, 35.7, 33.0, 32.9, 31.4, 30.9.

HRMS (APCI) [M+Na] calc. for $C_{16}H_{22}O_3Na$: m/z=285.1461, found: m/z=285.1464

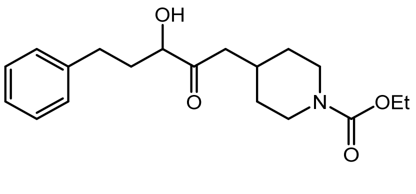

ethyl 4-(3-hydroxy-2-oxo-5-phenylpentyl)piperidine-1-carboxylate (23). The reaction was conducted with ethyl 4-(2,2-dichlorovinyl)piperidine-1-carboxylatediene **S2** (50.4 mg, 0.2 mmol) and hydrocinnamaldehyde (104-53-0) (53.7 mg, 0.4 mmol) without modification from general procedure B to provide **23** as a light yellow oil.

Run 1: 38.3 mg, 57% Yield. Run 2: 48.7 mg, 73% Yield

Purification: SiO_2 column; 10% Et_2O in CH_2Cl_2

TLC: R_f = 0.18 (10% Et_2O in CH_2Cl_2)

^{1}H NMR (400 MHz, $CDCl_3$) δ 7.34 – 7.25 (m, 2H), 7.25 – 7.14 (m, 3H), 4.11 (q, *J* = 6.8 Hz, 5H), 3.49 (d, *J* = 4.9 Hz, 1H), 2.85 – 2.68 (m, 4H), 2.42 – 2.25 (m, 2H), 2.17 – 1.95 (m, 2H), 1.77 (dtd, *J* = 14.1, 8.8, 5.5 Hz, 1H), 1.69 – 1.58 (m, 2H), 1.24 (t, *J* = 7.1 Hz, 3H), 1.18 – 1.00 (m, 2H).

$^{13}C\{^1H\}$ NMR (101 MHz, $CDCl_3$) δ 211.2, 155.6, 141.1, 128.7, 128.7, 126.4, 76.1, 61.4, 44.5, 43.9, 35.7, 32.1, 31.9, 31.8, 31.3, 14.8.

HRMS (APCI) [M+H] calc. for $C_{19}H_{28}NO_4$: m/z=334.2013, found: m/z=334.2011

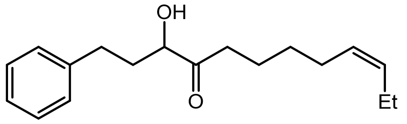

(*Z*)-3-hydroxy-1-phenyldodec-9-en-4-one (24). The reaction was conducted with (Z)-1,1-dichlorononа-1,6-diene[3] (38.6 mg, 0.2 mmol) hydrocinnamaldehyde (104-53-0) (53.7 mg, 0.4 mmol) without modification from general procedure B to provide **24** as a light yellow oil.

Run 1: 49.1 mg, 89% Yield. Run 2: 45.9 mg, 84% Yield.

Purification: SiO_2 column; 15% Et_2O in pentane

TLC: R_f = 0.21 (25% Et_2O in pentane)

^{1}H NMR (400 MHz, $CDCl_3$) δ 7.36 – 7.25 (m, 2H), 7.25 – 7.16 (m, 3H), 5.43 – 5.33 (m, 1H), 5.33 – 5.22 (m, 1H), 4.15 (dt, J = 8.2, 4.0 Hz, 1H), 3.56 (d, J = 4.7 Hz, 1H), 2.85 – 2.67 (m, 2H), 2.51 – 2.33 (m, 2H), 2.21 – 2.07 (m, 1H), 2.07 – 1.93 (m, 4H), 1.80 (dddd, J = 13.8, 9.3, 8.4, 5.3 Hz, 1H), 1.60 (dt, J = 15.3, 7.5 Hz, 2H), 1.39 – 1.23 (m, 2H), 0.95 (t, J = 7.6 Hz, 3H).

$^{13}C\ \{^1H\}$ NMR (101 MHz, $CDCl_3$) δ 212.3, 141.3, 132.4, 128.7, 128.7, 128.5, 126.3, 75.8, 37.9, 35.8, 31.3, 29.4, 26.9, 23.3, 20.7, 14.5.

HRMS (APCI) [M+H] calc. for $C_{18}H_{27}O_2$: m/z=275.2006, found: m/z=275.2009

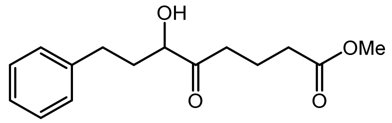

methyl 6-hydroxy-5-oxo-8-phenyloctanoate (25). The reaction was conducted with methyl 5,5-dichloropent-4-enoate[16] (36.6 mg, 0.2 mmol) and hydrocinnamaldehyde (104-53-0) (53.7 mg, 0.4 mmol) without modification from general procedure B to provide **25** as a light yellow oil.

Run 1: 36.9 mg, 70% Yield. Run 2: 37.7 mg, 71% Yield.

Purification: SiO_2 column; 40% Et_2O in pentane

TLC: R_f = 0.11 (40% Et_2O in pentane)

^{1}H NMR (400 MHz, $CDCl_3$) δ 7.34 – 7.27 (m, 2H), 7.24 – 7.17 (m, 3H), 4.15 (dd, *J* = 8.4, 3.5 Hz, 1H), 3.66 (s, 3H), 2.85 – 2.67 (m, 2H), 2.61 – 2.38 (m, 2H), 2.34 (t, *J* = 7.1 Hz, 2H), 2.13 (m, 1H), 1.92 (p, *J* = 7.1 Hz, 2H), 1.81 (m, 1H).

^{13}C {^{1}H} NMR (101 MHz, $CDCl_3$) δ 211.5, 173.4, 141.2, 128.8, 128.7, 126.3, 75.8, 51.8, 36.8, 35.7, 33.0, 31.3, 18.8.

HRMS (APCI) [M+H] calc. for $C_{15}H_{21}O_4$: m/z=265.1434, found: m/z=265.1440

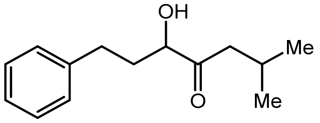

3-hydroxy-6-methyl-1-phenylheptan-4-one (26). The reaction was conducted with 1,1-dichloro-3-methylbut-1-ene (32363-91-0) (27.8 mg, 0.2 mmol) and hydrocinnamaldehyde (104-53-0) (53.7 mg, 0.4 mmol) without modification from general procedure B to provide **26** as a light yellow oil.

Run 1: 36.1 mg, 82% Yield. Run 2: 34.4 mg, 78% Yield

Purification: SiO_2 column; 20% Et_2O in pentane

TLC: R_f = 0.27 (25% Et_2O in pentane)

^{1}H NMR (400 MHz, $CDCl_3$) δ 7.35 – 7.27 (m, 2H), 7.24 – 7.15 (m, 3H), 4.12 (ddd, *J* = 8.3, 4.7, 3.4 Hz, 1H), 3.60 (d, *J* = 4.8 Hz, 1H), 2.86 – 2.66 (m, 2H), 2.34 – 2.28 (m, 1H), 2.27 – 2.06 (m, 2H), 1.78 (dddd, *J* = 13.8, 9.4, 8.4, 5.3 Hz, 1H), 0.92 (dd, *J* = 6.6, 4.4 Hz, 6H).

^{13}C {^{1}H} NMR (101 MHz, $CDCl_3$) δ 211.9, 141.3, 128.7, 128.6, 126.3, 76.1, 46.9, 35.7, 31.4, 24.6, 22.8, 22.6.

HRMS (APCI) [M+Na] calc. for $C_{14}H_{20}O_2Na$: m/z=243.1353, found: m/z=243.1357

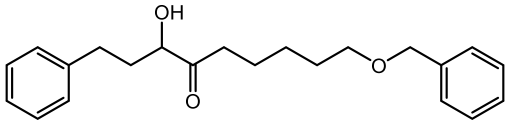

9-(benzyloxy)-3-hydroxy-1-phenylnonan-4-one (27).

The reaction was conducted with (((6,6-dichlorohex-5-en-1-yl)oxy)methyl)benzene **S1** (51.8 mg, 0.2 mmol) and hydrocinnamaldehyde (104-53-0) (53.7 mg, 0.4 mmol) without modification from general procedure B to provide **27** as a light yellow oil.

Run 1: 51.5 mg, 76% Yield. Run 2: 56.2 mg, 83% Yield.

Purification: SiO_2 column; 30% Et_2O in pentane

TLC: R_f = 0.22 (40% Et_2O in pentane)

^{1}H NMR (400 MHz, $CDCl_3$) δ 7.38 – 7.26 (m, 7H), 7.24 – 7.16 (m, 3H), 4.49 (s, 2H), 4.14 (dd, *J* = 8.4, 3.4 Hz, 1H), 3.45 (t, *J* = 6.5 Hz, 2H), 2.85 – 2.67 (m, 2H), 2.52 – 2.33 (m, 2H), 2.19 – 2.06 (m, 1H), 1.79 (dddd, *J* = 13.8, 9.3, 8.4, 5.3 Hz, 1H), 1.67 – 1.55 (m, 4H), 1.47 – 1.30 (m, 2H).

^{13}C {^{1}H} NMR (101 MHz, $CDCl_3$) δ 212.2, 141.3, 138.7, 128.7, 128.7, 128., 127.8, 127.7, 126.3, 75.8, 73.1, 70.1, 37.9, 35.7, 31.3, 29.6, 26.0, 23.5.

HRMS (APCI) [M+H] calc. for $C_{22}H_{29}O_3$: m/z=341.2111, found: m/z=341.2116

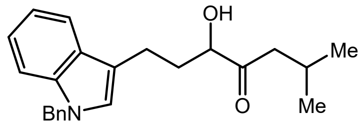

1-(1-benzyl-1H-indol-2-yl)-3-hydroxy-6-methylheptan-4-one (28). The reaction was conducted with 1,1-dichloro-3-methylbut-1-ene (32363-91-0) (27.8 mg, 0.2 mmol) and 3-(1-benzyl-1H-indol-2-yl)propanal[17] (105.3 mg, 0.4 mmol) without modification from general procedure B to provide **28** as a cream colored solid.

Run 1: 46.2 mg, 66% Yield. Run 2: 50.4 mg, 70% Yield

Purification: SiO_2 column; 20% Et_2O in pentane

TLC: R_f = 0.10 (25% Et_2O in pentane)

MP: 44-47 °C

^{1}H NMR (400 MHz, $CDCl_3$) δ 7.63 (dt, *J* = 7.7, 1.1 Hz, 1H), 7.34 – 7.07 (m, 8H), 6.95 (s, 1H), 5.28 (s, 2H), 4.21 – 4.12 (m, 1H), 3.61 (d, *J* = 4.8 Hz, 1H), 3.02 – 2.85 (m, 2H), 2.30 – 2.07 (m, 4H), 1.86 (dtd, *J* = 14.0, 8.4, 5.7 Hz, 1H), 0.88 (dd, *J* = 6.6, 1.8 Hz, 6H).

^{13}C {^{1}H} NMR (101 MHz, $CDCl_3$) δ 212.7, 137.8, 136.9, 128.9, 128.2, 127.7, 126.9, 126.1, 122.0, 120.2, 119.2, 114.5, 109.9, 76.3, 50.0, 46.8, 34.5, 24.7, 22.8, 22.6, 20.8.

HRMS (APCI) [M+H] calc. for $C_{13}H_{19}NO_2$: m/z=350.2115, found: m/z=350.2110

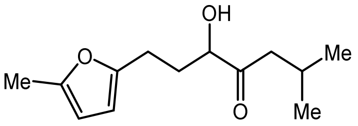

3-hydroxy-6-methyl-1-(5-methylfuran-2-yl)heptan-4-one (29). The reaction was conducted with 1,1-dichloro-3-methylbut-1-ene (32363-91-0) (27.8 mg, 0.2 mmol) and ethyl 3-(5-methylfuran-2-yl)propanal (34756-16-6) (55.3 mg, 0.4 mmol) without modification from general procedure B to provide **29** as a light yellow oil.

Run 1: 33.9 mg, 76% Yield. Run 2: 30.7 mg, 68% Yield.

Purification: SiO_2 column; 10% Et_2O in pentane

TLC: R_f = 0.18 (15% Et_2O in pentane)

^{1}H NMR (400 MHz, $CDCl_3$) δ 5.91 – 5.81 (m, 2H), 4.12 (ddd, *J* = 8.3, 4.5, 3.4 Hz, 1H), 3.56 (d, *J* = 4.8 Hz, 1H), 2.82 – 2.64 (m, 2H), 2.35 – 2.29 (m, 2H), 2.26 (s, 3H), 2.24 – 2.11 (m, 2H), 1.76 (dtd, *J* = 14.0, 8.5, 5.6 Hz, 1H), 0.92 (dd, *J* = 6.6, 3.9 Hz, 6H).

^{13}C {^{1}H} NMR (101 MHz, $CDCl_3$) δ 211.8, 153.0, 150.8, 106.5, 106.1, 76.0, 46.8, 32.4, 24.7, 23.8, 22.8, 22.7, 13.7.

HRMS (APCI) [M+H] calc. for $C_{13}H_{21}O_3$: m/z=225.1485, found: m/z=225.1483

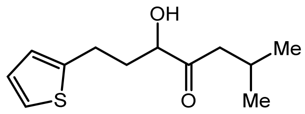

3-hydroxy-6-methyl-1-(thiophen-2-yl)heptan-4-one (30). The reaction was conducted with 1,1-dichloro-3-methylbut-1-ene (32363-91-0) (27.8 mg, 0.2 mmol) and 3-(thiophen-2-yl)propanal[18] (26359-21-7)(50.5mg, 0.4 mmol) without modification from general procedure B to provide **30** as a light yellow oil.

Run 1: 36.8 mg, 81% Yield. Run 2: 36.0 mg, 80% Yield.

Purification: SiO_2 column; 10% Et_2O in pentane

TLC: R_f = 0.18 (15% Et_2O in pentane)

^{1}H NMR (400 MHz, $CDCl_3$) δ 7.15 (dd, *J* = 5.1, 1.2 Hz, 1H), 6.93 (dd, *J* = 5.1, 3.4 Hz, 1H), 6.83 (dq, *J* = 3.3, 1.0 Hz, 1H), 4.14 (ddd, *J* = 8.5, 4.8, 3.3 Hz, 1H), 3.57 (d, *J* = 4.9 Hz, 1H), 3.11 – 2.86 (m, 2H), 2.36 – 2.29 (m, 2H), 2.28 – 2.12 (m, 2H), 1.81 (dtd, *J* = 13.8, 8.9, 5.0 Hz, 1H), 0.92 (dd, *J* = 6.6, 4.3 Hz, 6H).

^{13}C {^{1}H} NMR (101 MHz, $CDCl_3$) δ 211.8, 143.9, 127.1, 125.1, 123.6, 75.8, 46.9, 35.9, 25.6, 24.7, 22.8, 22.7.

HRMS (APCI) [M+H] calc. for $C_{12}H_{19}O_2S$: m/z=227.1100, found: m/z=227.1103

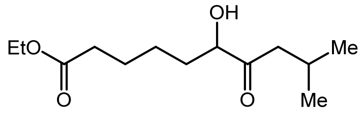

ethyl 6-hydroxy-9-methyl-7-oxodecanoate (31). The reaction was conducted with 1,1-dichloro-3-methylbut-1-ene (32363-91-0) (27.8 mg, 0.2 mmol) and ethyl 6-oxohexanoate[19] (63.3 mg, 0.4 mmol) without modification from general procedure B to provide **31** as a light yellow oil.

Run 1: 31.4 mg, 64% Yield. Run 2: 21.2 mg, 43% Yield.

Purification: SiO_2 column; 30% Et_2O in pentane

^{1}H NMR (500 MHz, $CDCl_3$) δ 4.23 – 4.10 (m, 3H), 3.55 (d, *J* = 4.9 Hz, 1H), 2.45 – 2.33 (m, 4H), 2.26 (dp, *J* = 13.5, 6.7 Hz, 1H), 1.96 – 1.82 (m, 1H), 1.80 – 1.64 (m, 2H), 1.62 – 1.48 (m, 2H), 1.50 – 1.37 (m, 1H), 1.30 (t, *J* = 7.1 Hz, 3H), 0.99 (dd, *J* = 6.6, 4.1 Hz, 6H).

^{13}C{^{1}H} NMR (101 MHz, $CDCl_3$) δ 212.0, 173.6, 76.6, 60.4, 46.9, 34.2, 33.4, 24.8, 24.7, 24.6, 22.7, 22.7, 14.4.

HRMS (APCI) [M+H] calc. for $C_{13}H_{25}O_4$: m/z=245.17526, found: m/z=245.17353

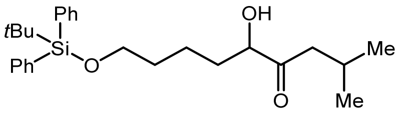

9-((tert-butyldiphenylsilyl)oxy)-5-hydroxy-2-methylnonan-4-one (32). The reaction was conducted with 1,1-dichloro-3-methylbut-1-ene (32363-91-0) (27.8 mg, 0.2 mmol)and 5-((tert-butyldiphenylsilyl)oxy)pentanal[20] (136.2 mg, 0.4 mmol) without modification from general procedure B to provide **32** as a light yellow oil.

Run 1: 53.2 mg, 62% Yield. Run 2: 34.7 mg, 41% Yield.

Purification: 15% Et_2O in pentane

TLC: R_f = 0.17 (15% Et_2O in pentane)

^1H NMR (400 MHz, $CDCl_3$) δ 7.70 – 7.62 (m, 4H), 7.47 – 7.33 (m, 6H), 4.15 – 4.06 (m, 1H), 3.66 (t, J = 6.1 Hz, 2H), 3.49 (d, *J* = 4.9 Hz, 1H), 2.32 (d, *J* = 6.9 Hz, 2H), 2.20 (ddd, *J* = 12.9, 7.3, 6.4 Hz, 1H), 1.85 – 1.74 (m, 1H), 1.69 – 1.34 (m, 5H), 1.04 (s, 9H), 0.93 (dd, *J* = 6.6, 1.9 Hz, 6H).

$^{13}C\{^1H\}$ NMR (101 MHz, $CDCl_3$) δ 212.1, 135.7, 134.1, 129.7, 127.8, 63.7, 46.9, 33.5, 32.4, 27.0, 24.7, 22.8, 22.7, 21.4, 19.4.

HRMS (APCI) [M+H] calc. for $C_{26}H_{39}O_3Si$: m/z=427.2663, found: m/z=427.2666

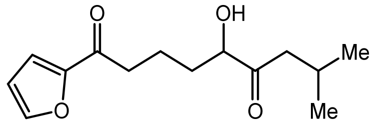

1-(furan-2-yl)-5-hydroxy-8-methylnonane-1,6-dione (33). The reaction was conducted with 1,1-dichloro-3-methylbut-1-ene (32363-91-0) (27.8 mg, 0.2 mmol) and 5-(furan-2-yl)-5-oxopentanal (**S4**) (66.5 mg, 0.4 mmol) without modification from general procedure B to provide **33** as a light yellow oil.

Run 1: 25.2 mg, 50% Yield. Run 2: 25.6 mg, 51% Yield

Purification: SiO_2 column; 5% Et_2O in CH_2Cl_2

TLC: R_f = 0.19 (5% Et_2O in CH_2Cl_2)

^1H NMR (400 MHz, $CDCl_3$) δ 7.57 (dd, *J* = 1.7, 0.8 Hz, 1H), 7.18 (dd, *J* = 3.6, 0.8 Hz, 1H), 6.53 (dd, *J* = 3.5, 1.7 Hz, 1H), 4.15 (ddd, *J* = 8.1, 4.9, 3.4 Hz, 1H), 3.56 (d, *J* = 4.8 Hz, 1H), 2.89 (td, *J* = 6.9, 2.0 Hz, 2H), 2.45 – 2.29 (m, 2H), 2.20 (dp, *J* = 13.4, 6.7 Hz, 1H), 1.98 – 1.72 (m, 3H), 1.66 – 1.50 (m, 1H), 0.93 (dd, *J* = 6.6, 4.7 Hz, 6H).

$^{13}C\{^1H\}$ NMR (101 MHz, $CDCl_3$) δ 212.0, 189.2, 146.5, 117.1, 112.4, 76.6, 46.9, 37.8, 32.9, 24.7, 22.8, 22.7, 19.6.

HRMS (APCI) [M+Na] calc. for $C_{14}H_{20}O_4Na$: m/z=275.1254, found: m/z=275.1255

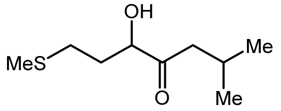

3-hydroxy-6-methyl-1-(methylthio)heptan-4-one (34). The reaction was conducted with 1,1-dichloro-3-methylbut-1-ene (32363-91-0) (27.8 mg, 0.2 mmol) and 3-(methylthio)propionaldehyde (3268-49-3) (41.7 mg, 0.4 mmol) without modification from general procedure B to provide **34** as a light yellow oil.

Run 1: 8.1 mg, 23% Yield. Run 2: 5.0 mg, 13% Yield.

Purification: SiO_2 column; 20% Et_2O in pentane

TLC: R_f = 0.18 (25% Et_2O in pentane)

^1H NMR (400 MHz, $CDCl_3$) δ 4.25 (ddd, *J* = 8.4, 4.9, 3.3 Hz, 1H), 3.55 (d, *J* = 4.9 Hz, 1H), 2.75 – 2.53 (m, 2H), 2.37 (dd, J = 7.0, 2.0 Hz, 2H), 2.29 – 2.14 (m, 1H), 2.13 (s, 3H), 2.14 – 2.02 (m, 1H), 1.74 (dtd, J = 13.8, 8.3, 5.2 Hz, 1H), 0.94 (dd, *J* = 6.7, 2.8 Hz, 6H).

$^{13}C\{^1H\}$ NMR (101 MHz, $CDCl_3$) δ 211.6, 75.7, 47.0, 33.4, 30.2, 24.8, 22.8, 22.7, 15.9.

HRMS (APCI) [M+H] calc. for $C_9H_{19}O_2S$: m/z=191.1100, found: m/z=191.1102

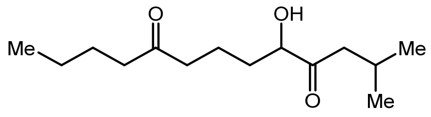

5-hydroxy-2-methyltridecane-4,9-dione (35). The reaction was conducted with 1,1-dichloro-3-methylbut-1-ene (32363-91-0) (27.8 mg, 0.2 mmol) and 5-oxononanal[21] (62.5 mg, 0.4 mmol) without modification from general procedure B to provide **35** as a light yellow oil.

Run 1: 22.8 mg, 47% Yield. Run 2: 31.4 mg, 65% Yield.

Purification: SiO_2 column; 25% Et_2O in pentane

TLC: R_f = 0.07 (25% Et_2O in pentane)

^1H NMR (400 MHz, $CDCl_3$) δ 4.09 (dt, *J* = 7.8, 3.9 Hz, 1H), 3.55 (d, *J* = 4.8 Hz, 1H), 2.50 – 2.32 (m, 6H), 2.18 (dp, *J* = 13.4, 6.7 Hz, 1H), 1.93 – 1.42 (m, 6H), 1.35 – 1.21 (m, 2H), 0.96 – 0.82 (m, 9H).

$^{13}C\{^1H\}$ NMR (101 MHz, $CDCl_3$) δ 211.9, 211.1, 76.6, 46.8, 42.7, 42.1, 32.9, 26.1, 24.6, 22.7, 22.6, 22.5, 19.2, 14.0.

HRMS (APCI) [M-H] calc. for $C_{14}H_{25}O_3$: m/z=241.1798, found: m/z=241.1801

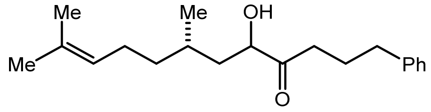

(7*S*)-5-hydroxy-7,11-dimethyl-1-phenyldodec-10-en-4-one (36). The reaction was conducted with (4,4-dichlorobut-3-en-1-yl)benzene (40.2 mg, 0.2 mmol) and (*S*)-citronellal (5949-05-3) (61.7 mg, 0.4 mmol) without modification from general procedure B to provide **36** as a light yellow oil.

Run 1: 47.6 mg, 79% Yield. Run 2: 45.7 mg, 76% Yield. 1:1 d.r.

Purification: SiO_2 column; 10% Et_2O in pentane

TLC: R_f = 0.21 (15% Et_2O in pentane)

^1H NMR (400 MHz, $CDCl_3$) δ 7.33 – 7.25 (m, 2H), 7.25 – 7.13 (m, 3H), 5.10 (dtp, *J* = 9.9, 7.1, 1.4 Hz, 1H), 4.20 – 4.11 (m, 1H), 3.37 (t, *J* = 4.9 Hz, 1H), 2.64 (td, *J* = 7.6, 1.6 Hz, 2H), 2.58 – 2.34 (m, 2H), 2.05 – 1.87 (m, 2H), 1.87 – 1.72 (m, 1H), 1.75 – 1.56 (m, 7H), 1.58 – 1.47 (m, 1H), 1.51 – 1.37 (m, 1H), 1.41 – 1.27 (m, 1H), 1.27 (ddd, *J* = 7.9, 4.5, 1.9 Hz, 1H), 1.26 – 1.06 (m, 1H), 0.95 (dd, *J* = 15.1, 6.7 Hz, 3H).

$^{13}C\{^1H\}$ NMR (101 MHz, $CDCl_3$) δ 212.7, 141.3, 131.5, 128.6, 128.6, 126.2, 124.7, 124.6, 75.3, 74.9, 41.5, 41.3, 38.1, 37.1, 37.0, 35.8, 35.1, 29.5, 29.2, 25.9, 25.6, 25.4, 25.2, 20.5, 18.7, 17.8.

HRMS (APCI) [M+H] calc. for $C_{20}H_{31}O_2$: m/z=303.2319, found: m/z=303.2321

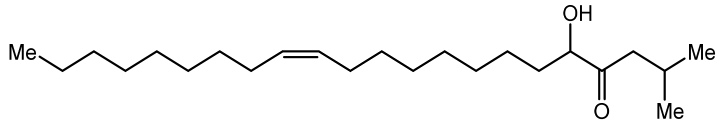

(*Z*)-5-hydroxy-2-methyldocos-13-en-4-one (37). The reaction was conducted with 1,1-dichloro-3-methylbut-1-ene (32363-91-0) (27.8 mg, 0.2 mmol) and olealdehyde[22] (106.6 mg, 0.4 mmol) without modification from general procedure B to provide **37** as a light yellow oil.

Run 1: 35.1 mg, 50% Yield. Run 2: 35.0 mg, 50% Yield

Purification: 5% Et_2O in pentane

TLC: R_f = 0.08 (5% Et_2O in pentane)

^{1}H NMR (500 MHz, $CDCl_3$) δ 5.40 – 5.29 (m, 2H), 4.15 – 4.09 (m, 1H), 3.48 (d, *J* = 5.0 Hz, 1H), 2.38 – 2.32 (m, 2H), 2.21 (dp, *J* = 13.4, 6.7 Hz, 1H), 2.09 – 1.97 (m, 4H), 1.86 – 1.76 (m, 1H), 1.53 – 1.42 (m, 1H), 1.37 – 1.32 (m, 2H), 1.35 – 1.24 (m, 22H), 0.94 (dd, *J* = 6.6, 4.6 Hz, 6H), 0.88 (t, *J* = 6.9 Hz, 3H).

$^{13}C\{^1H\}$ NMR (126 MHz, C_6D_6) δ 211.5, 130.3, 130.2, 76.8, 46.5, 34.1, 32.3, 30.3, 30.2, 30.0, 29.9, 29.9, 29.8, 29.8, 29.7, 27.7, 27.7, 25.4, 24.5, 23.1, 22.6, 22.5, 14.4.

HRMS (APCI) [M-H] calc. for $C_{23}H_{43}O_2$: m/z=351.3263; found: m/z=351.3276

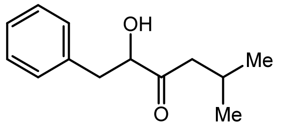

2-hydroxy-5-methyl-1-phenylhexan-3-one (38). The reaction was conducted with 1,1-dichloro-3-methylbut-1-ene (32363-91-0) (27.8 mg, 0.2 mmol) and 2-phenylacetaldehyde (122-78-1) (48.0 mg, 0.4 mmol) without modification from general procedure B to provide **38** as a light yellow oil.

Run 1: 30.2 mg, 73% Yield. Run 2: 30.3 mg, 73% Yield.

Purification: SiO_2 column; 10% Et_2O in pentane

TLC: R_f = 0.15 (15% Et_2O in pentane)

^{1}H NMR (400 MHz, $CDCl_3$) δ 7.36 – 7.27 (m, 2H), 7.31 – 7.20 (m, 3H), 4.37 (dt, *J* = 7.5, 4.8 Hz, 1H), 3.43 (d, *J* = 5.4 Hz, 1H), 3.13 (dd, *J* = 14.1, 4.5 Hz, 1H), 2.83 (dd, *J* = 14.1, 7.6 Hz, 1H), 2.37 (d, *J* = 6.9 Hz, 2H), 2.18 (dp, *J* = 13.5, 6.7 Hz, 1H), 0.93 (d, *J* = 6.7 Hz, 6H).

$^{13}C\{^1H\}$ NMR (101 MHz, $CDCl_3$) δ 211.3, 136.8, 129.4, 128.7, 127.0, 77.6, 47.6, 40.2, 29.8, 24.7, 22.8, 22.7.

HRMS (APCI) [M+H] calc. for $C_{13}H_{19}O_2$: m/z=207.1380, found: m/z=207.1378

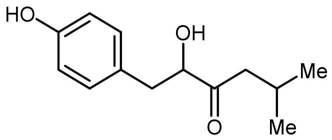

2-hydroxy-1-(4-hydroxyphenyl)-5-methylhexan-3-one [(±) 4-hydroxysattabacin] (39). The reaction was conducted with 1,1-dichloro-3-methylbut-1-ene (32363-91-0) (27.8 mg, 0.2 mmol) and 2-(4-((trimethylsilyl)oxy)phenyl)acetaldehyde[23] (83.3 mg, 0.4 mmol) without modification from general procedure B to provide **39** as a light yellow oil.

Run 1: 17.8 mg, 40% Yield. Run 2: 18.6 mg, 42% Yield

Purification: 40% Et_2O in pentane

TLC: R_f = 0.13 (40% Et_2O in pentane)

^{1}H NMR (400 MHz, $CDCl_3$) δ 7.09 (d, *J* = 8.5 Hz, 2H), 6.75 (d, *J* = 8.5 Hz, 2H), 4.92 (s, 1H), 4.34 (dt, *J* = 7.3, 4.8 Hz, 1H), 3.45 (d, *J* = 5.4 Hz, 1H), 3.07 (dd, *J* = 14.3, 4.5 Hz, 1H), 2.76 (dd, *J* = 14.3, 7.4 Hz, 1H), 2.37 (d, *J* = 6.9 Hz, 2H), 2.27 – 2.09 (m, 1H), 0.92 (d, *J* = 6.7 Hz, 5H).

$^{13}C\{^1H\}$ NMR (101 MHz, $CDCl_3$) δ 211.5, 154.7, 130.6, 128.7, 115.6, 77.7, 47.6, 39.3, 24.8, 22.8, 22.7.

HRMS (APCI) [M+H] calc. for $C_{13}H_{19}O_3$=223. 1329; found m/z=223.1335

5. Mechanistic Studies

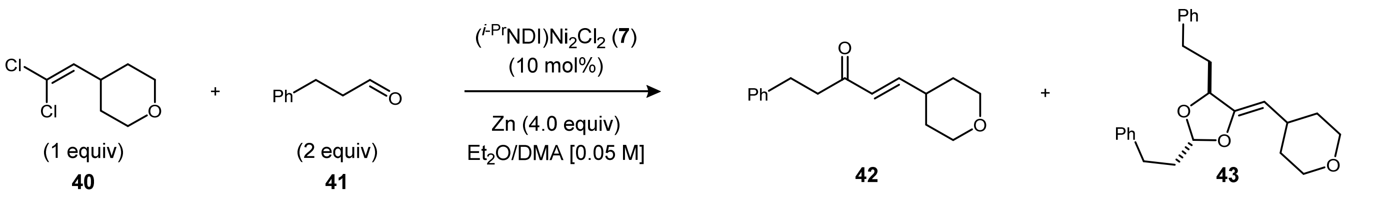

13% yield, >20:1 *E/Z* 61% yield, 2:1 dr, >20:1 *Z/E*

Catalytic C(sp^2)–H bond insertion vs. [2 + 2 + 1]-cycloaddition under dilute conditions. In an N_2-filled glovebox, a 2-dram vial was charged with a magnetic stir bar, ($^{i\text{-Pr}}$NDI)Ni_2Cl_2 (**7**) (7.2 mg, 0.01 mmol, 10 mol%), and Zn powder (26.1 mg, 0.4 mmol, 4.0 equiv). DMA (0.4 mL) was added. A stock solution of 1,1-dichloroalkene **40**[5] (9.0 mg, 0.10 mmol, 1.0 equiv), aldehyde **41** (104-53-0) (13.4 mg, 0.2 mmol, 2.0 equiv), mesitylene (12 mg, 0.10 mmol, 1.0 equiv), and Et_2O (0.4 mL) was added. The reaction was diluted with additional Et_2O (1.2 mL). The vial was sealed, and the reaction mixture was stirred (600 rpm) at room temperature for 24 h. The reaction was removed from the glovebox and exposed to air. An aliquot was removed, filtered through a small pad of silica (approx. 1 cm) eluting with $CDCl_3$, and analyzed by ^1H NMR spectroscopy. The product yields were determined by ^1H NMR integration against mesitylene. The products were isolated via column chromatography (10 to 25% Et_2O in pentane). COSY and NOESY were used to determine stereochemistry of **43**. See figure S79 for crude ^1H NMR.

(*E*)-5-phenyl-1-(tetrahydro-2H-pyran-4-yl)pent-1-en-3-one (42).

13 % NMR Yield

TLC: R_f = 0.19 (20% EtOAc in hexanes)

^1H NMR (500 MHz, $CDCl_3$) δ 7.33 – 7.26 (m, 2H), 7.24 – 7.16 (m, 4H), 6.72 (dd, *J* = 16.0, 6.6 Hz, 1H), 6.07 (dd, *J* = 16.0, 1.4 Hz, 1H), 3.99 (ddd, *J* = 11.6, 4.6, 2.0 Hz, 2H), 3.43 (td, *J* = 11.8, 2.2 Hz, 2H), 3.02 – 2.79 (m, 4H), 2.44 – 2.28 (m, 1H), 1.65 (ddd, *J* = 13.2, 4.1, 2.1 Hz, 2H), 1.56 – 1.39 (m, 2H).

$^{13}C\{^1H\}$ NMR (126 MHz, $CDCl_3$) δ 199.8, 150.2, 141.4, 128.6, 128.6, 128.5, 126.3, 67.5, 42.0, 38.0, 31.5, 30.2.

HRMS (APCI) [M-H] calc. for $C_{16}H_{19}O_2$=243.1385. found m/z=243.1395

(*Z*)-4-((2,5-diphenethyl-1,3-dioxolan-4-ylidene)methyl)tetrahydro-2H-pyran (43).

61% NMR Yield; 2:1 d.r.; >20:1 Z/E

TLC: R_f = 0.62 (20% EtOAc in hexanes)

^1H NMR (500 MHz, $CDCl_3$) δ 7.34 – 7.17 (m, 10H), **43-*anti*** – 5.39 (t, *J* = 4.8 Hz, 1H), **43-*syn*** – 5.21 (t, *J* = 4.5 Hz, 1H), **43-*anti*** – 4.56 (ddd, *J* = 8.5, 4.2, 1.4 Hz, 1H), **43-*syn*** – 4.43 (dt, *J* = 8.1, 2.5 Hz, 1H), 4.07 (dd, *J* = 8.5, 1.4 Hz, 1H), **43-*syn*** – 4.03 (dd, *J* = 8.5, 1.7 Hz, 2H), **43-*anti*** – 3.93 (ddd, *J* = 11.6, 4.3, 2.3 Hz, 2H), 3.46 (tt, *J* = 11.6, 2.2 Hz, 2H), 2.88 – 2.68 (m, 4H), 2.64 – 2.53 (m, 1H), **43-*syn*** – 2.15 – 2.08 (m, 1H), 2.08 – 1.99 (m, 2H), 1.97 – 1.81 (m, 2H), 1.67 – 1.57 (m, 2H), 1.48 – 1.35 (m, 2H).

$^{13}C\{^1H\}$ NMR (126 MHz, $CDCl_3$) δ 151.7, 141.6, 141.4, 128.6, 128.6, 128.6, 126.1, 104.6, 99.6, 76.7, 68.0, 36.2, 36.0, 33.5, 33.2, 32.1, 31.7, 29.9.

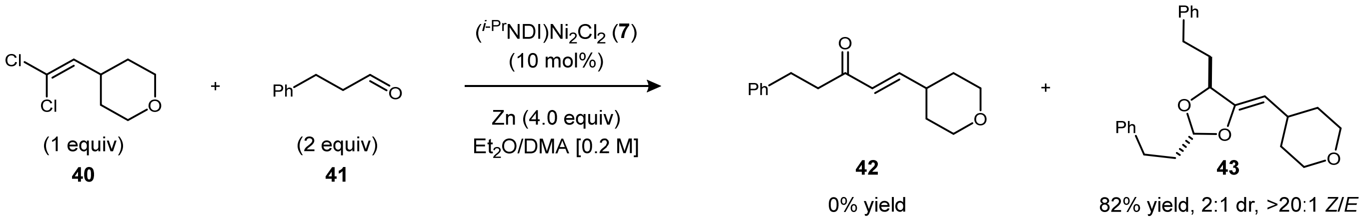

Catalytic C(sp^2)–H bond insertion vs. [2 + 2 + 1]-cycloaddition under concentrated conditions. In an N_2-filled glovebox, a 2-dram vial was charged with a magnetic stir bar, ($^{i\text{-Pr}}$NDI)Ni_2Cl_2 (**7**) (7.2 mg, 0.01 mmol, 10 mol%), and Zn powder (26.1 mg, 0.4 mmol, 4.0 equiv). DMA (0.1 mL) was added. A stock solution of 1,1-dichloroalkene **40** (18.1 mg, 0.1 mmol, 1.0 equiv), aldehyde **41** (13.4 mg, 0.2 mmol, 2.0 equiv), mesitylene (12 mg, 0.10 mmol, 1.0 equiv) and Et_2O (0.4 mL) was added. The vial was sealed, and the reaction mixture was stirred (600 rpm) at room temperature for 24 h. The reaction was removed from the glovebox and exposed to air. An aliquot was removed, filtered through a small pad of silica (approx. 1 cm) eluting with $CDCl_3$, and analyzed by ^{1}H NMR spectroscopy. The product yields were determined by ^{1}H NMR integration against mesitylene. See figure S80 for crude ^{1}H NMR analysis.

43: 82% Yield; 2:1 d.r.; >20:1 Z/E
42: 0 % Yield

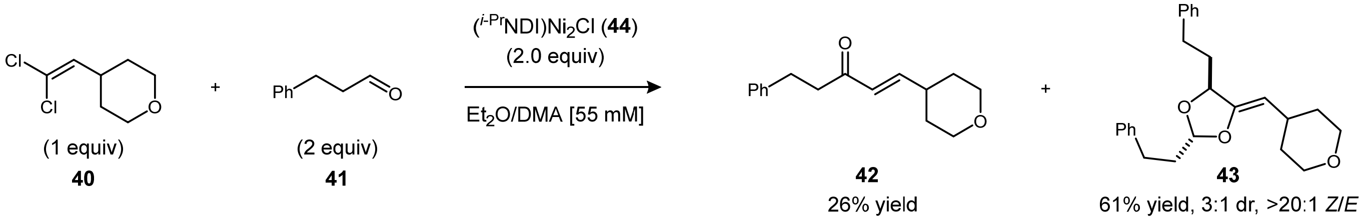

Stoichiometric [2 + 2 + 1]-cycloaddition using an isolable low-valent (NDI)Ni_2Cl complex with concentrated conditions. In an N_2 filled glovebox, a 2-dram vial was charged with a magnetic stir bar and ($^{i\text{-Pr}}$NDI)Ni_2Cl (**44**) (15.0 mg, 22.0 µmol, 2.00 equiv). DMA (40 µL) was added to the vial, forming a dark purple solution. A solution of 1,1-dichloroalkene **40** (2.0 mg, 11 µmol, 1.0 equiv), hydrocinnamaldehyde (**41**) (3.0 mg, 11 µmol, 2.0 equiv), and mesitylene (1.9 mg, 16 µmol, 1.6 equiv) in Et_2O (160 µL) was added to the purple solution. The reaction was stirred (600 rpm) at room temperature for 1.5 h. The reaction was removed from the glovebox and exposed to air. An aliquot was removed, filtered through a small pad of silica (approx. 1 cm) eluting with $CDCl_3$, and analyzed by ^{1}H NMR spectroscopy. The yield of dioxolane was determined by ^{1}H NMR integration against mesitylene. See figure S81 for crude ^{1}H NMR analysis.

42: 26% Yield.
43: 61% Yield. 3:1 d.r. (anti:syn). >20:1 Z/E

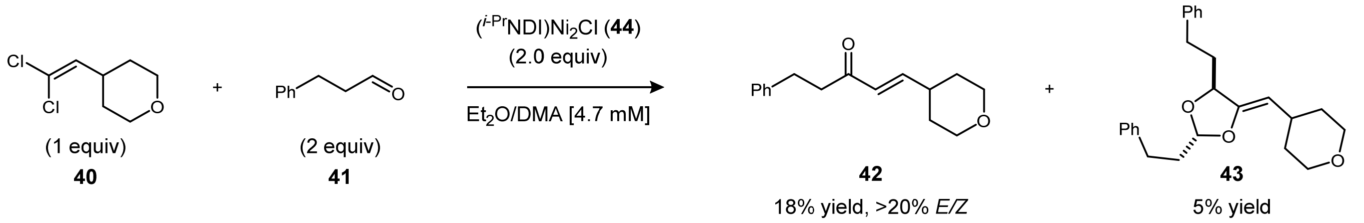

Stoichiometric [2 + 2 + 1]-cycloaddition using an isolable low-valent (NDI)Ni_2Cl complex with dilute conditions. In an N_2 filled glovebox, a 2-dram vial was charged with a magnetic stir bar and ($^{i\text{-}Pr}$NDI)Ni_2Cl (**44**) (22.7 mg, 33.1 µmol, 2.00 equiv). DMA (700 µL) was added to the vial, forming a dark purple solution. A 0.5 M stock solution (32 µL) of 1,1-dichloroalkene **40** (2.9 mg, 17 µmol, 1.0 equiv), hydrocinnamaldehyde (**41**) (4.6 mg, 34 µmol, 2.0 equiv), and trimethoxybenzene (5.7 mg, 34 µmol, 2.0 equiv) in Et_2O was added to the purple solution. An addition portion of Et_2O (2.8 mL) was added. The reaction was stirred (600 rpm) at room temperature for 24 h. The reaction was removed from the glovebox and exposed to air. An aliquot was removed, filtered through a small pad of silica (approx. 1 cm) eluting with $CDCl_3$, and analyzed by ^1H NMR spectroscopy. The yield of **42** and **43** were determined by ^1H NMR integration against trimethoxybenzene. See figure S82 for crude ^1H NMR.

42: 18% Yield. >20:1 E/Z
43: 5% Yield.

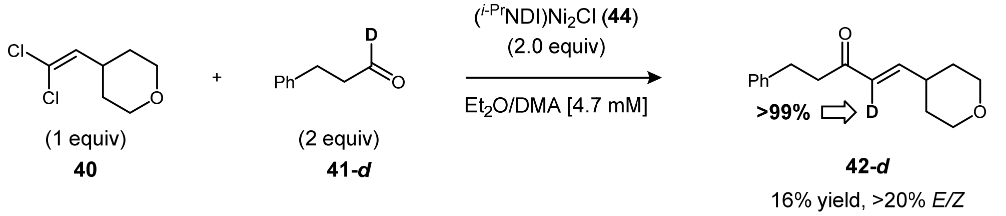

Stoichiometric bond insertion reaction with deuterium labeled substrate. In an N_2 filled glovebox, a 2-dram vial was charged with a magnetic stir bar and ($^{i\text{-}Pr}$NDI)Ni_2Cl (**44**) (22.7 mg, 33.1 µmol, 2.00 equiv). DMA (700 µL) was added to the vial, forming a dark purple solution. A 0.5 M stock solution (32 µL) of 1,1-dichloroalkene **40** (2.9 mg, 17 µmol, 1.0 equiv), aldehyde **41-*d***[24] (4.6 mg, 34 µmol, 2.0 equiv), and trimethoxybenzene (5.7 mg, 34 µmol, 2.0 equiv) in Et_2O was added to the purple solution. An additional portion of Et_2O (2.8 mL) was added. The reaction was stirred (600 rpm) at room temperature for 24 h. The reaction was removed from the glovebox and exposed to air. An aliquot was removed, filtered through a small pad of silica (approx. 1 cm) eluting with $CDCl_3$, and analyzed by ^1H NMR spectroscopy. The crude reaction mixture was then concentrated and purified using a pipette column of silica, eluting with 20% Et_2O/pentane. The yield of **42-*d*** was determined from ^1H NMR integration against trimethoxybenzene. See figure S83 for purified NMR highlighting deuterium incorporation.

42-*d*: 16% Yield. >20:1 E/Z

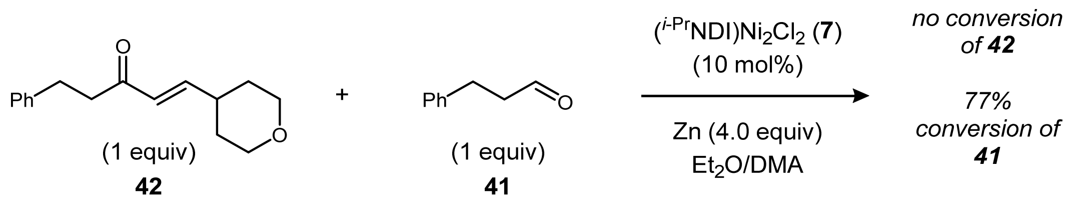

Resubjection of enone to catalytic conditions. In an N_2-filled glovebox, a 2-dram vial was charged with a magnetic stir bar, ($^{i\text{-Pr}}$NDI)Ni_2Cl_2 (3.4 mg, 4.8 µmol, 10 mol%), and Zn powder (12.4 mg, 190 µmol, 4.00 equiv). DMA (50 µL) was added. A solution of (*E*)-5-phenyl-1-(tetrahydro-2H-pyran-4-yl)pent-1-en-3-one (11.6 mg, 47.5 µmol, 1.00 equiv), hydrocinnamaldehyde (6.37 mg, 47.5 µmol, 1.00 equiv), mesitylene (14.3 mg, 0.12 mmol, 2.5 equiv), and Et_2O (190 µL) was added. The vial was sealed, and the reaction mixture was stirred (600 rpm) at room temperature for 24 h. The reaction was removed from the glovebox and exposed to air. An aliquot was removed, filtered through a small pad of silica (approx. 1 cm) eluting with $CDCl_3$, and analyzed by ^{1}H NMR spectroscopy. Conversion of **42** was determined by ^{1}H NMR integration against mesitylene. See figure S84 for crude ^{1}H NMR.

6. NMR Spectral Data

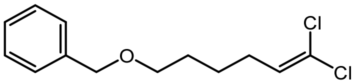

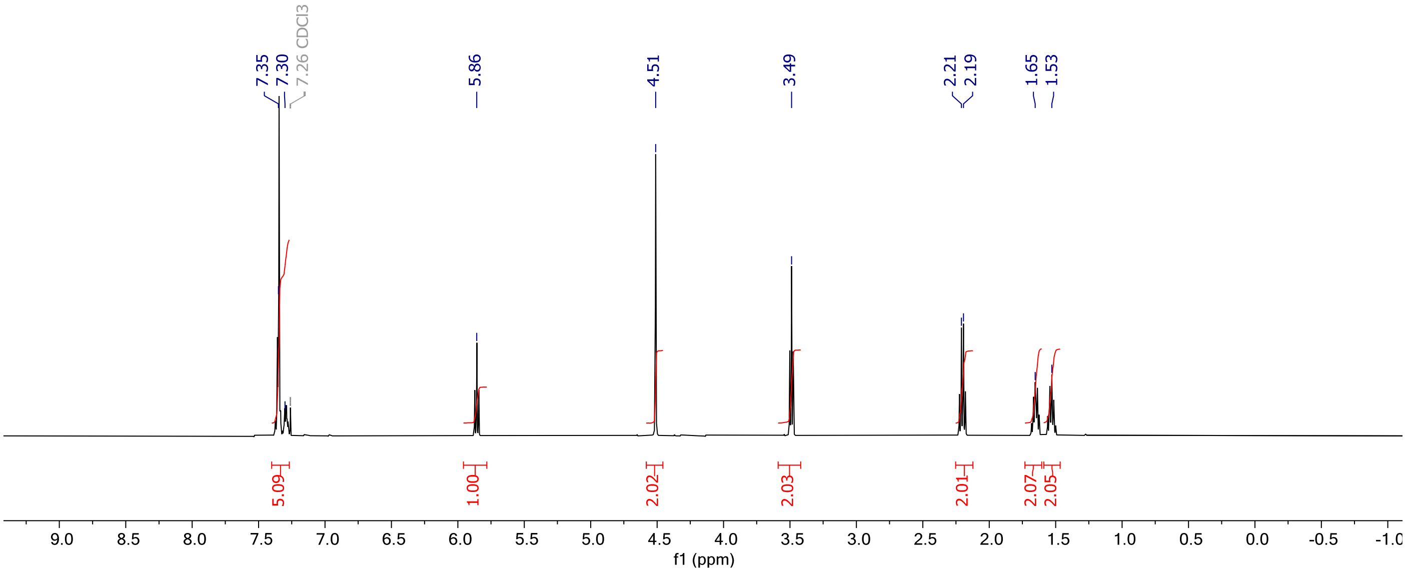

Figure S1. ^{1}H NMR spectrum for **S1** (500 MHz, $CDCl_3$, room temperature).

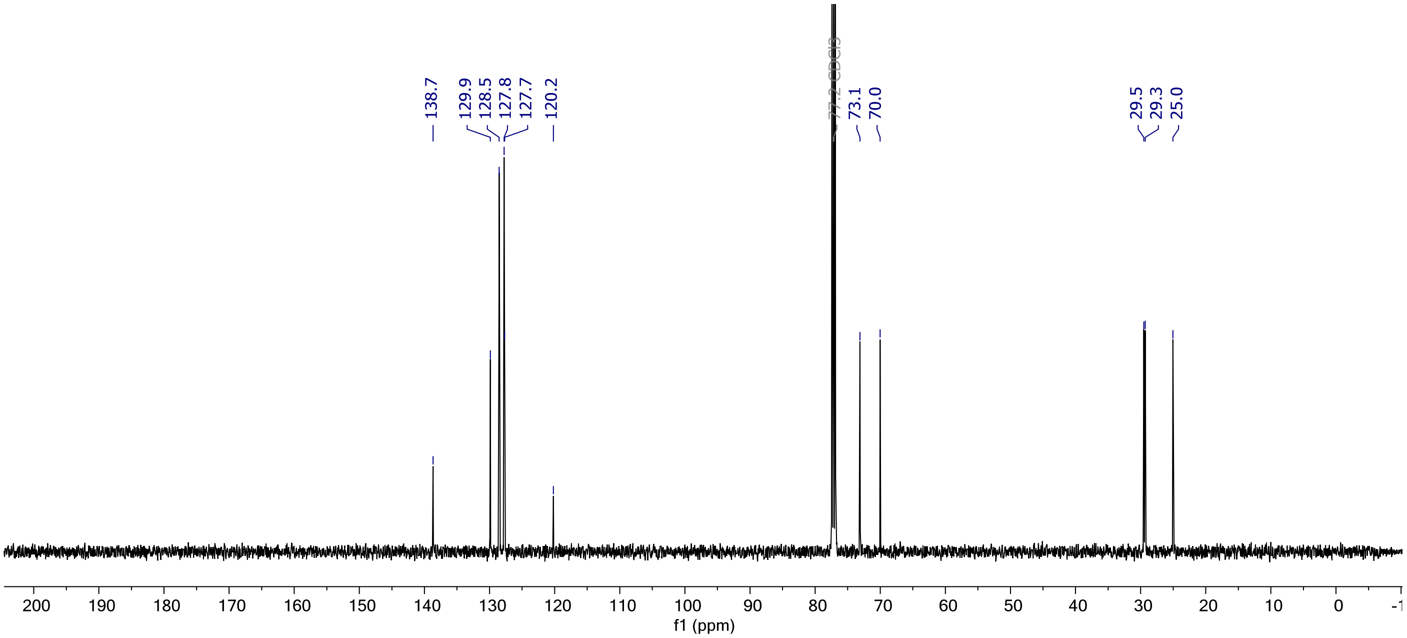

Figure S2. $^{13}C\{^{1}H\}$ NMR spectrum for **S1** (126 MHz, $CDCl_3$, room temperature).

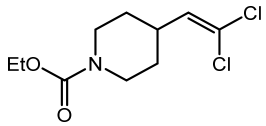

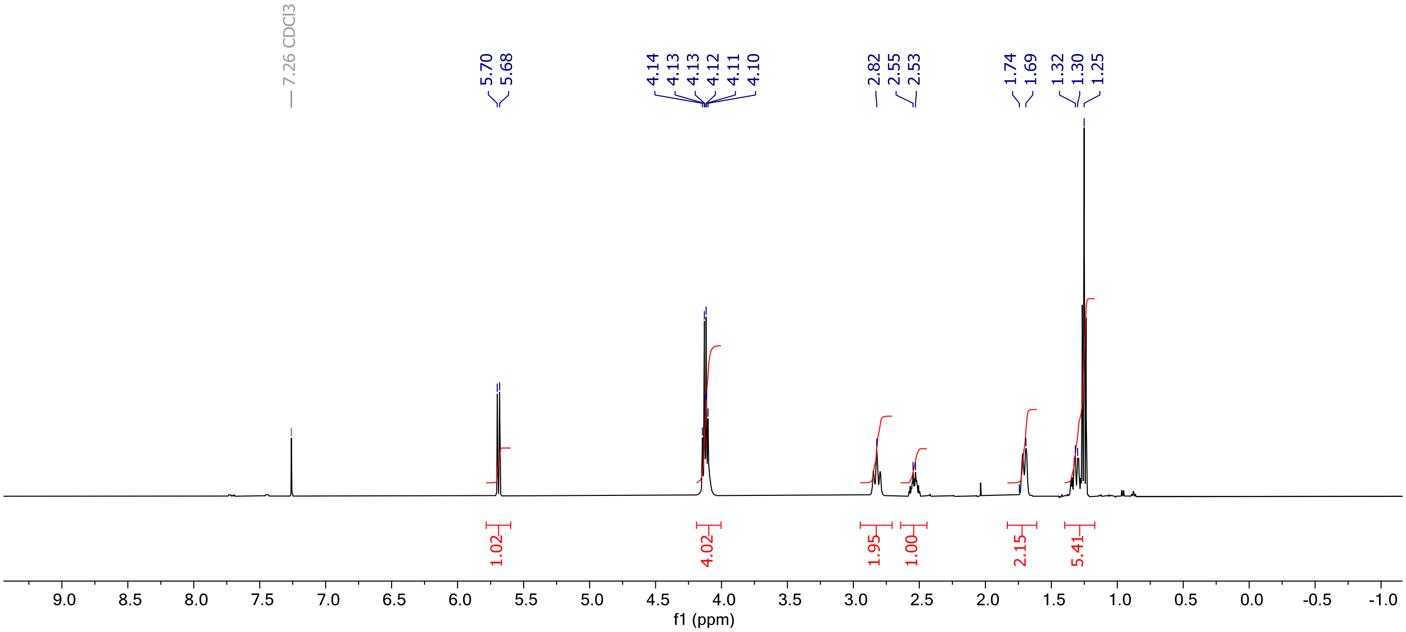

Figure S3. ^{1}H NMR spectrum for **S2** (500 MHz, $CDCl_3$, room temperature).

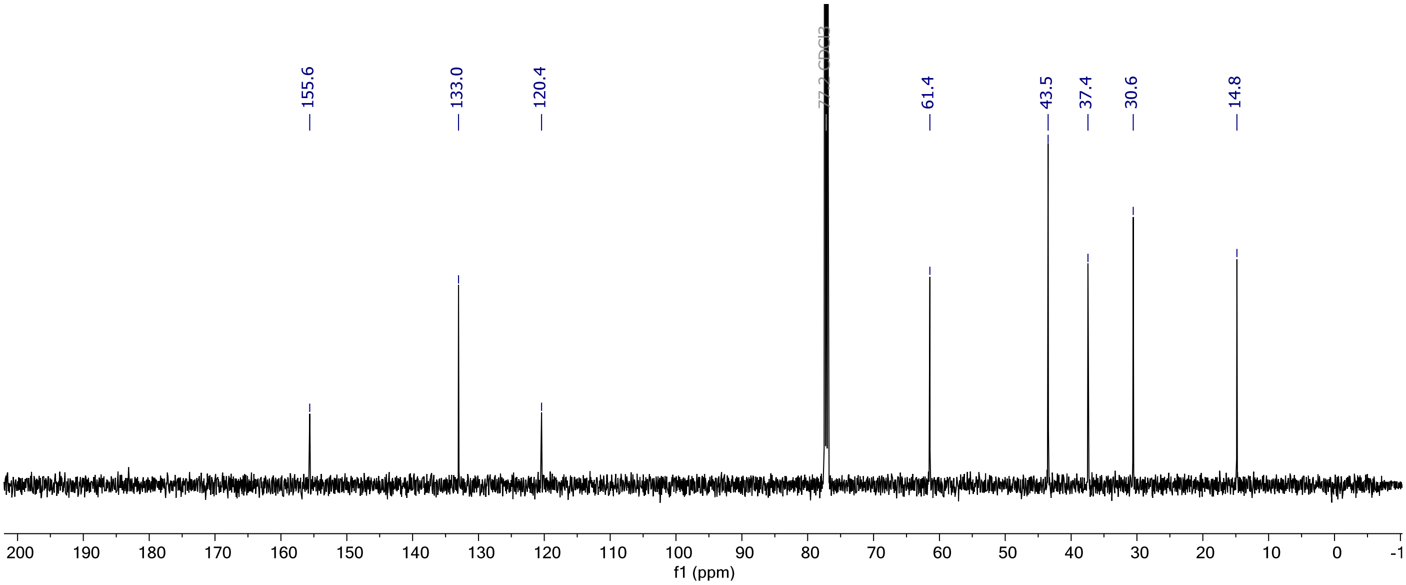

Figure S4. $^{13}C\{^{1}H\}$ NMR spectrum for **S2** (126 MHz, $CDCl_3$, room temperature).

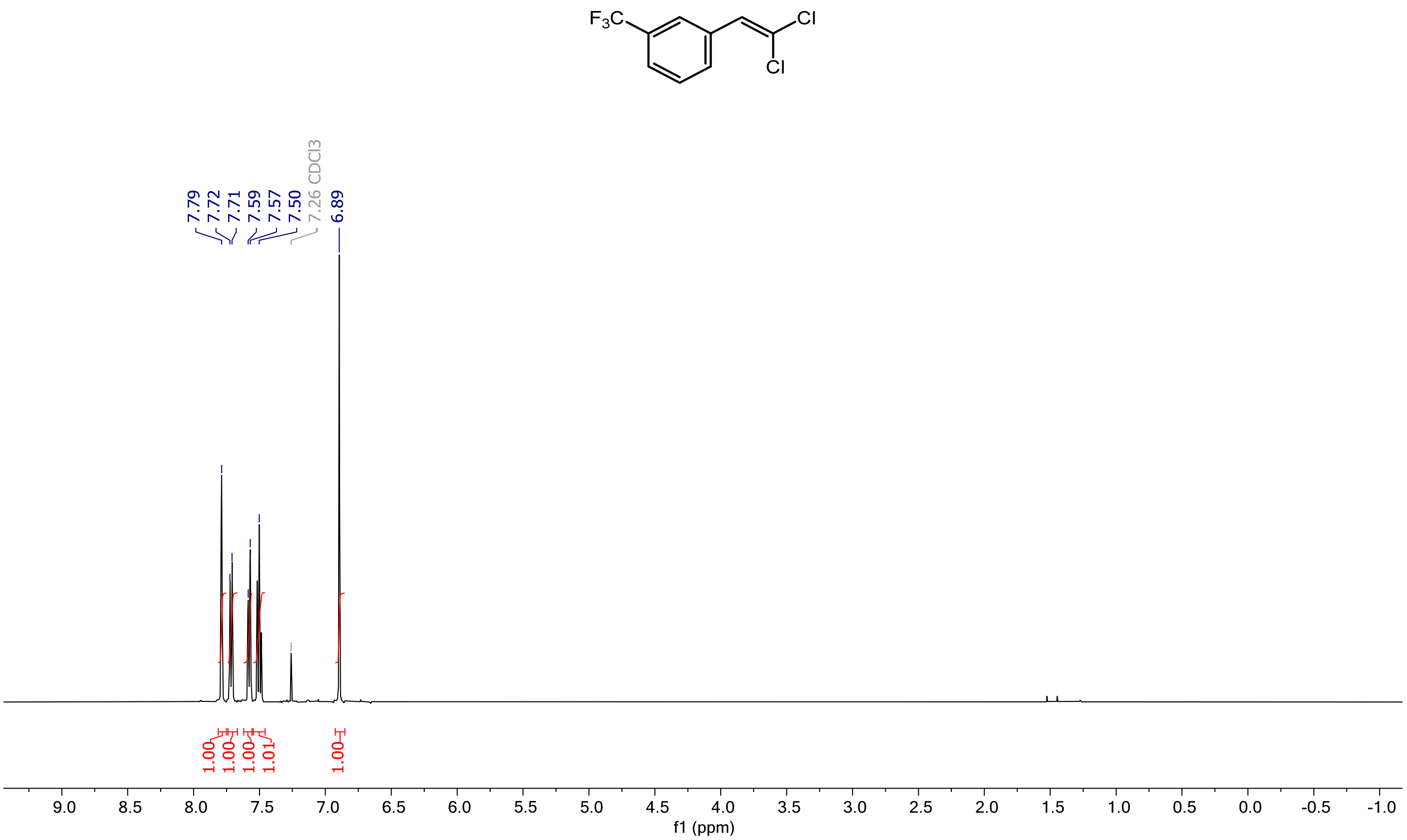

Figure S5. ^{1}H NMR spectrum for **S3** (500 MHz, $CDCl_3$, room temperature).

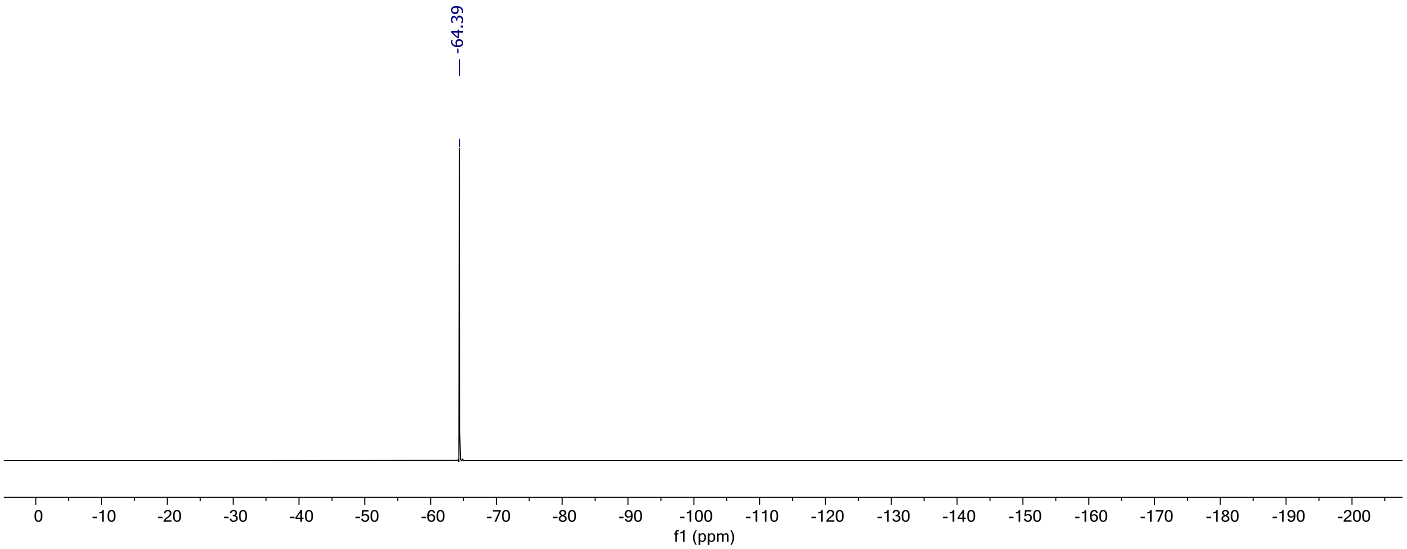

Figure S6. ^{19}F NMR spectrum for **S3** (470 MHz, $CDCl_3$, room temperature).

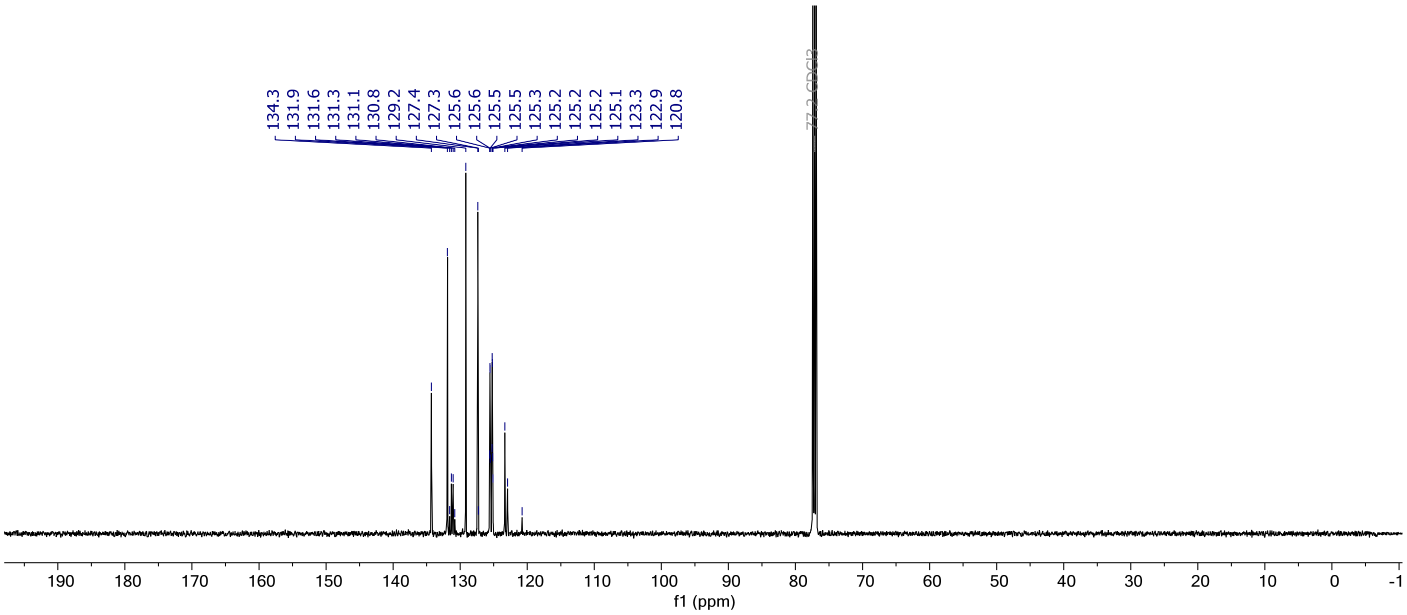

Figure S7. $^{13}C\{^{1}H\}$ NMR spectrum for **S3** (126 MHz, $CDCl_3$, room temperature).

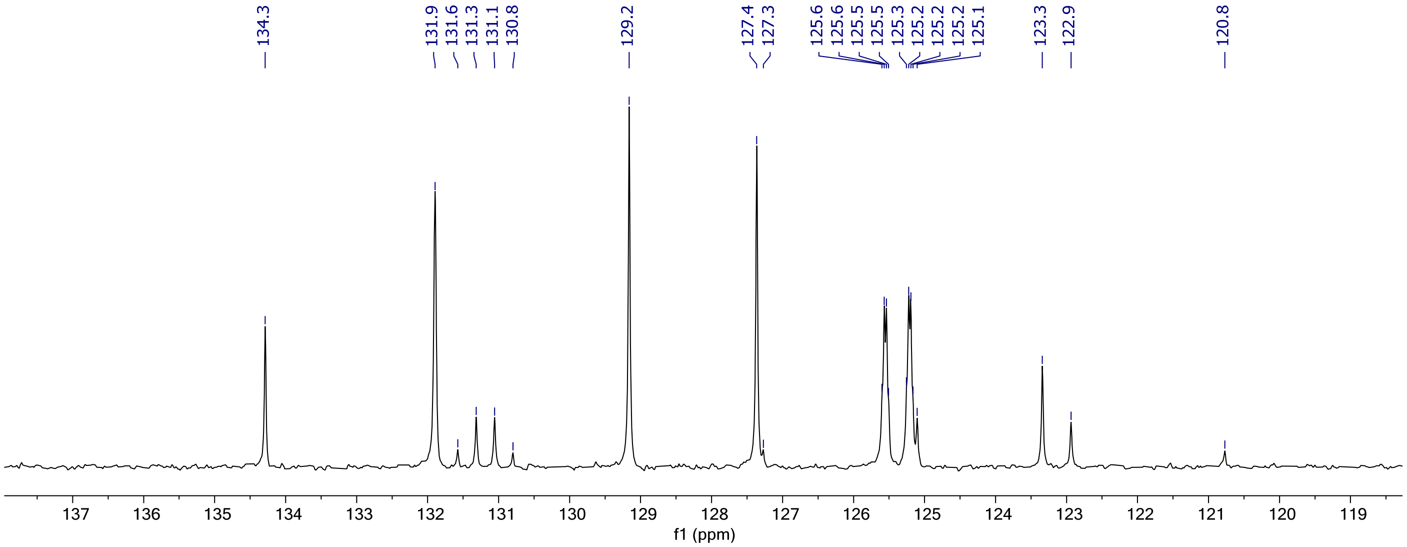

Figure S8. Expansion of $^{13}C\{^{1}H\}$ NMR spectrum for **S3** (126 MHz, $CDCl_3$, room temperature).

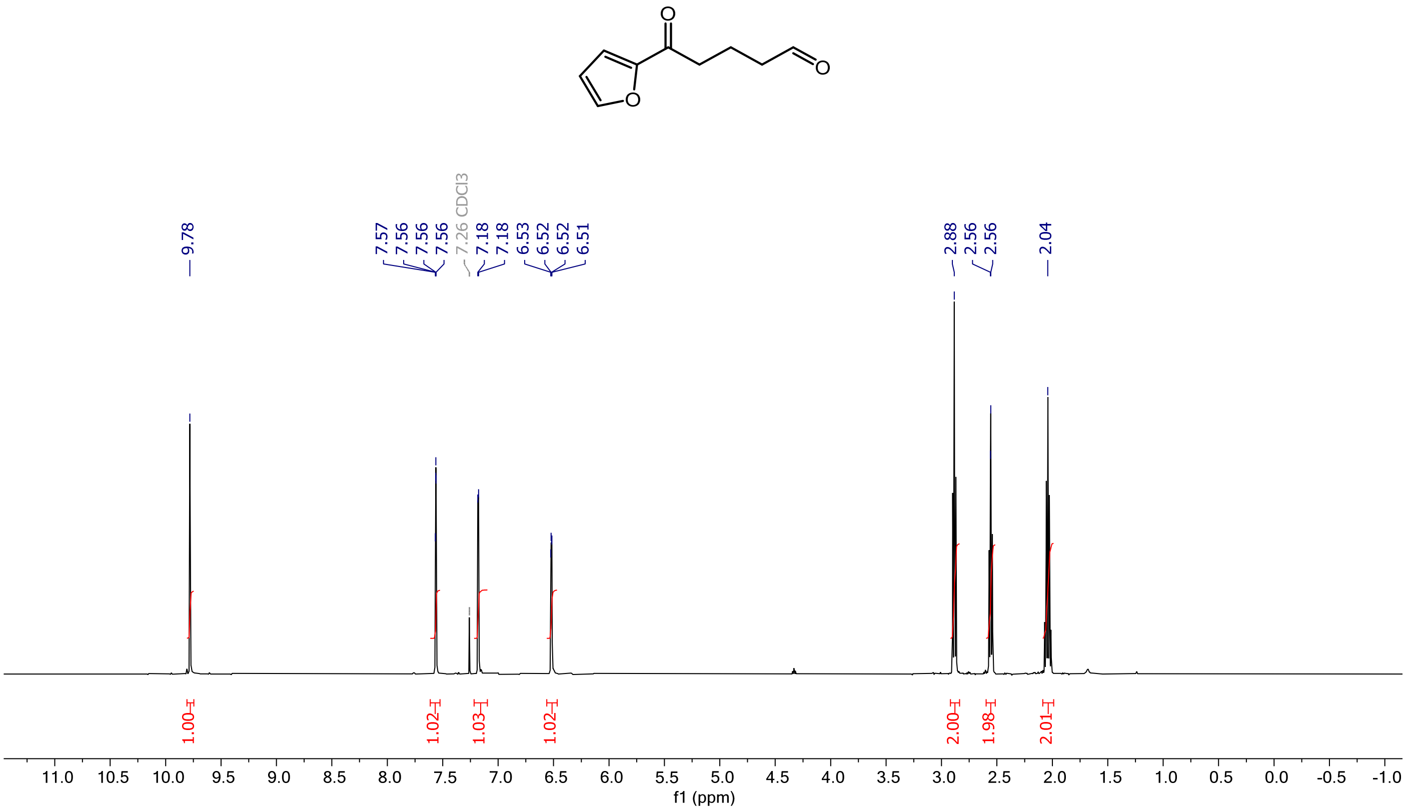

Figure S9. ^{1}H NMR spectrum for **S4** (500 MHz, $CDCl_3$, room temperature).

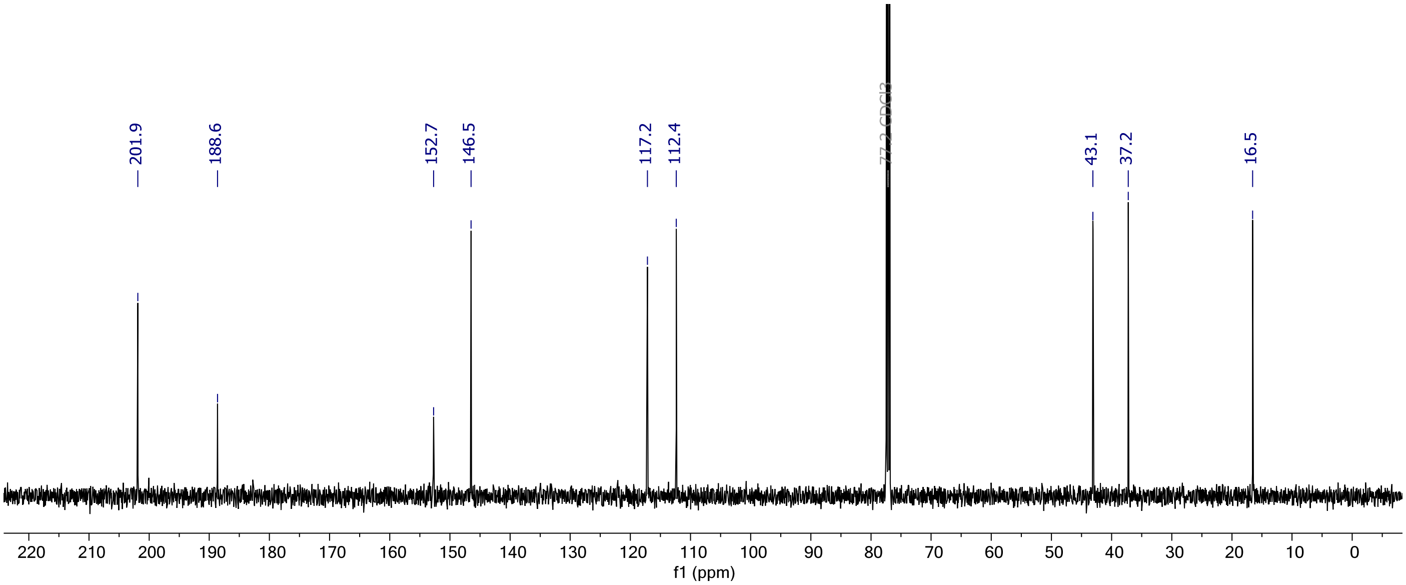

Figure S10. $^{13}C\{^{1}H\}$ NMR spectrum for **S4** (126 MHz, $CDCl_3$, room temperature).

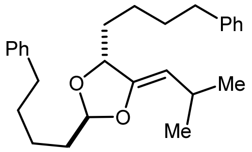

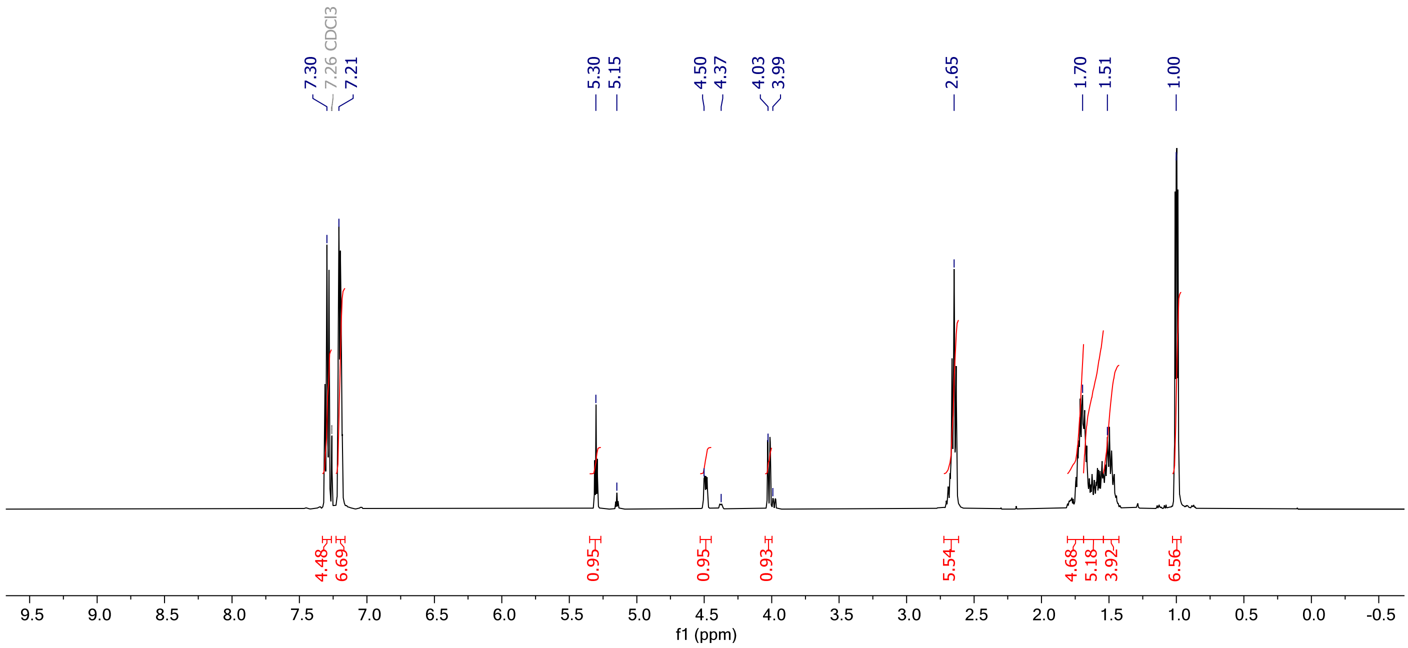

Figure S11. ^{1}H NMR spectrum for **4** (500 MHz, $CDCl_3$, room temperature).

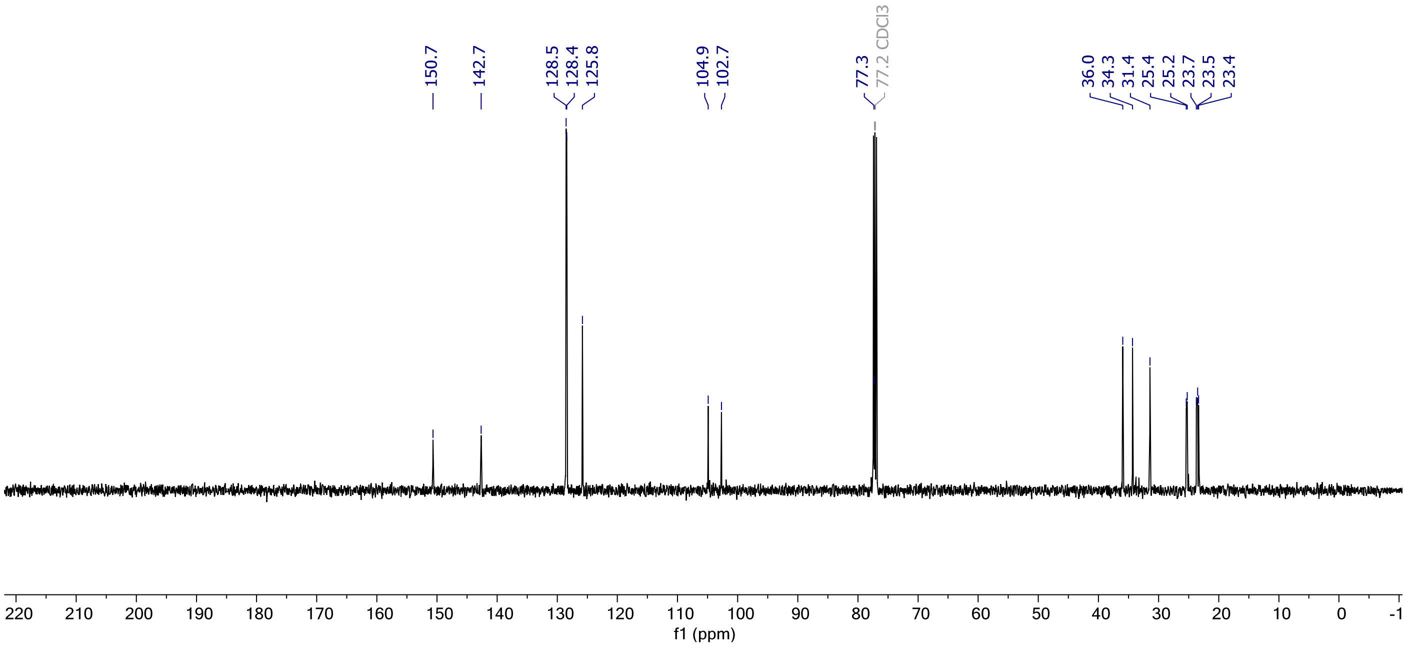

Figure S12. $^{13}C\{^{1}H\}$ NMR spectrum for **4** (126 MHz, $CDCl_3$, room temperature).

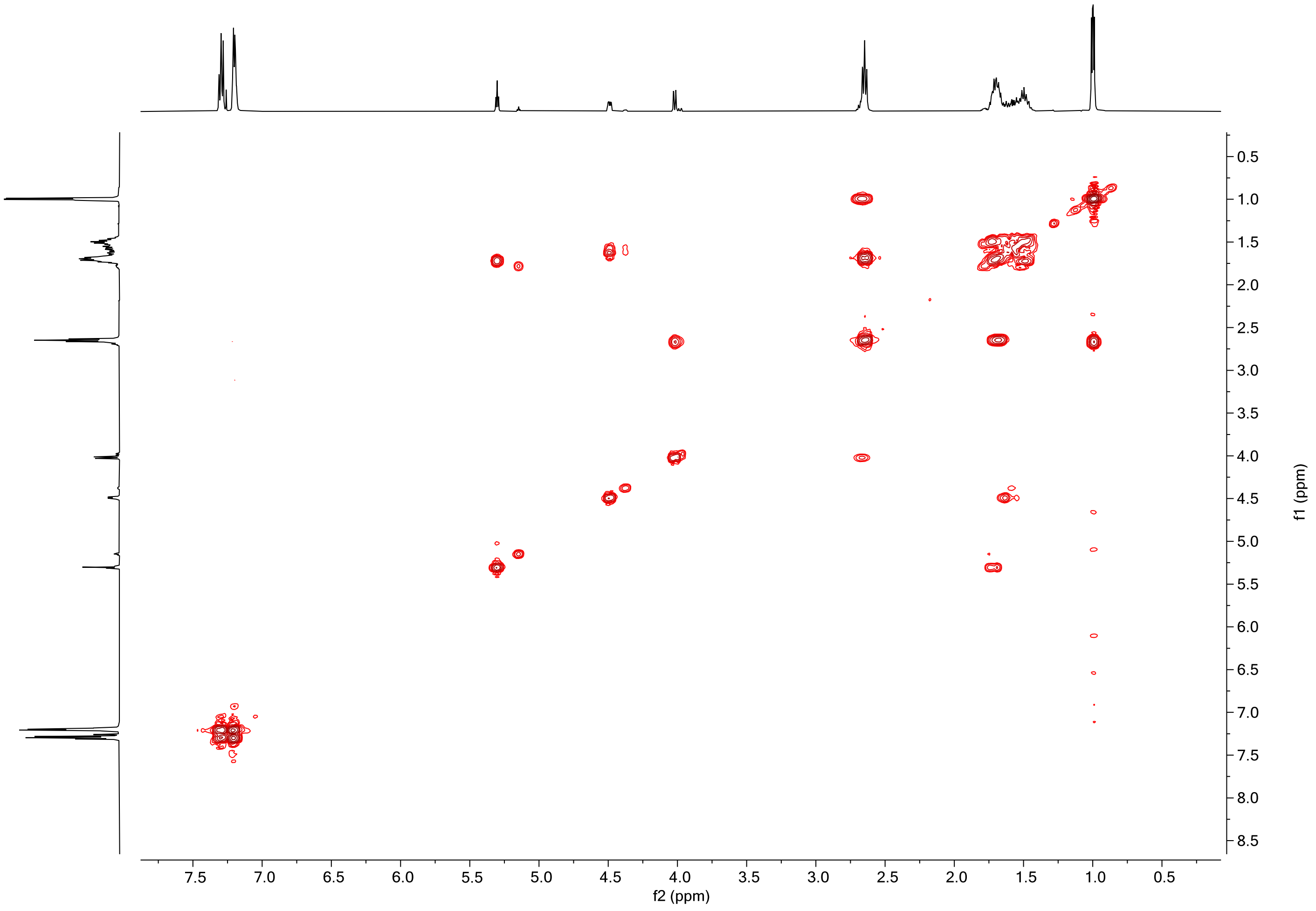

Figure S13. COSY NMR spectrum for **4** (500 MHz, $CDCl_3$, room temperature).

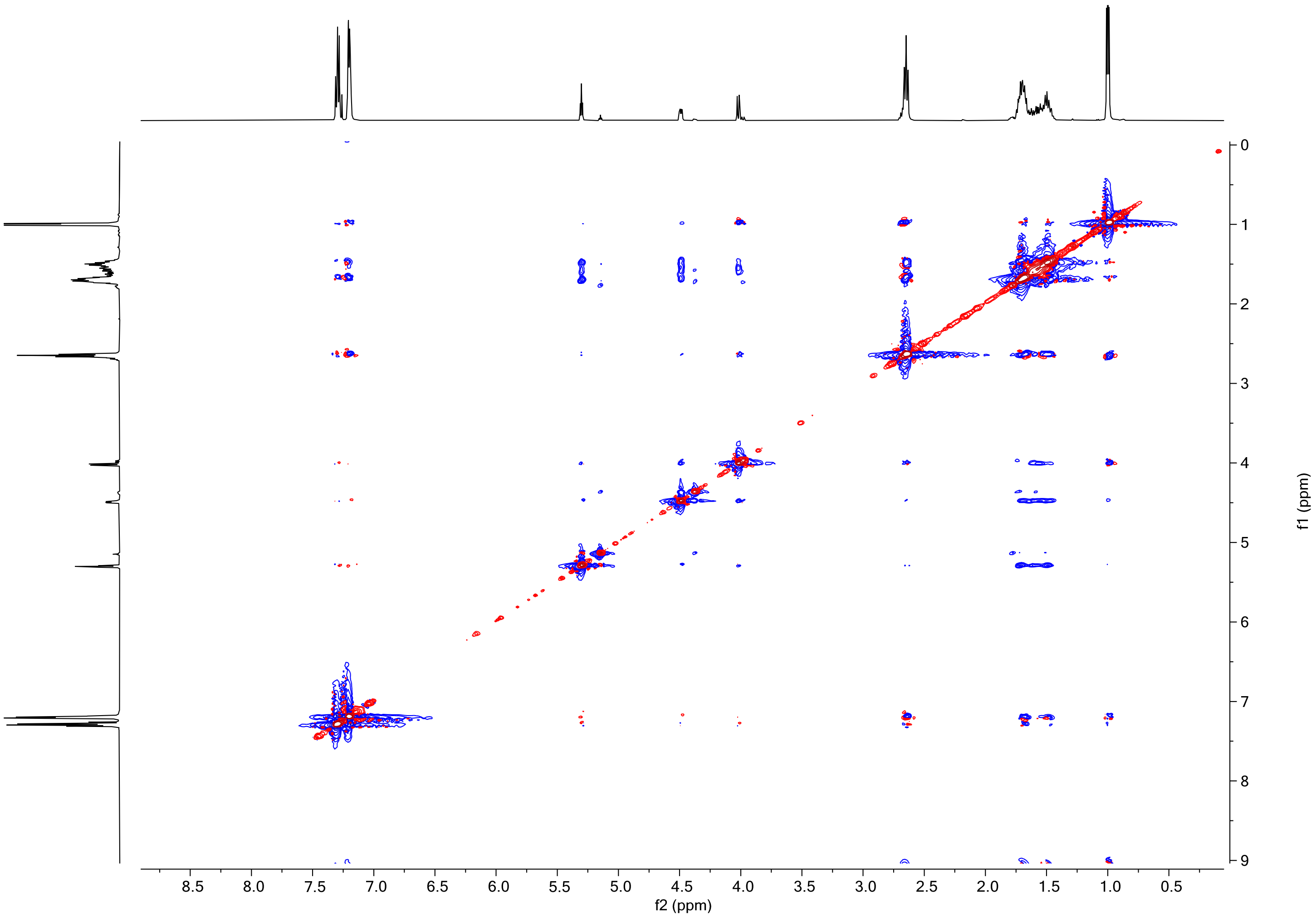

Figure S14. NOESY NMR spectrum for **4** (500 MHz, $CDCl_3$, room temperature).

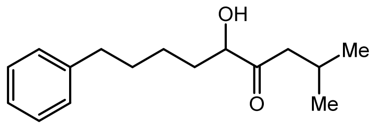

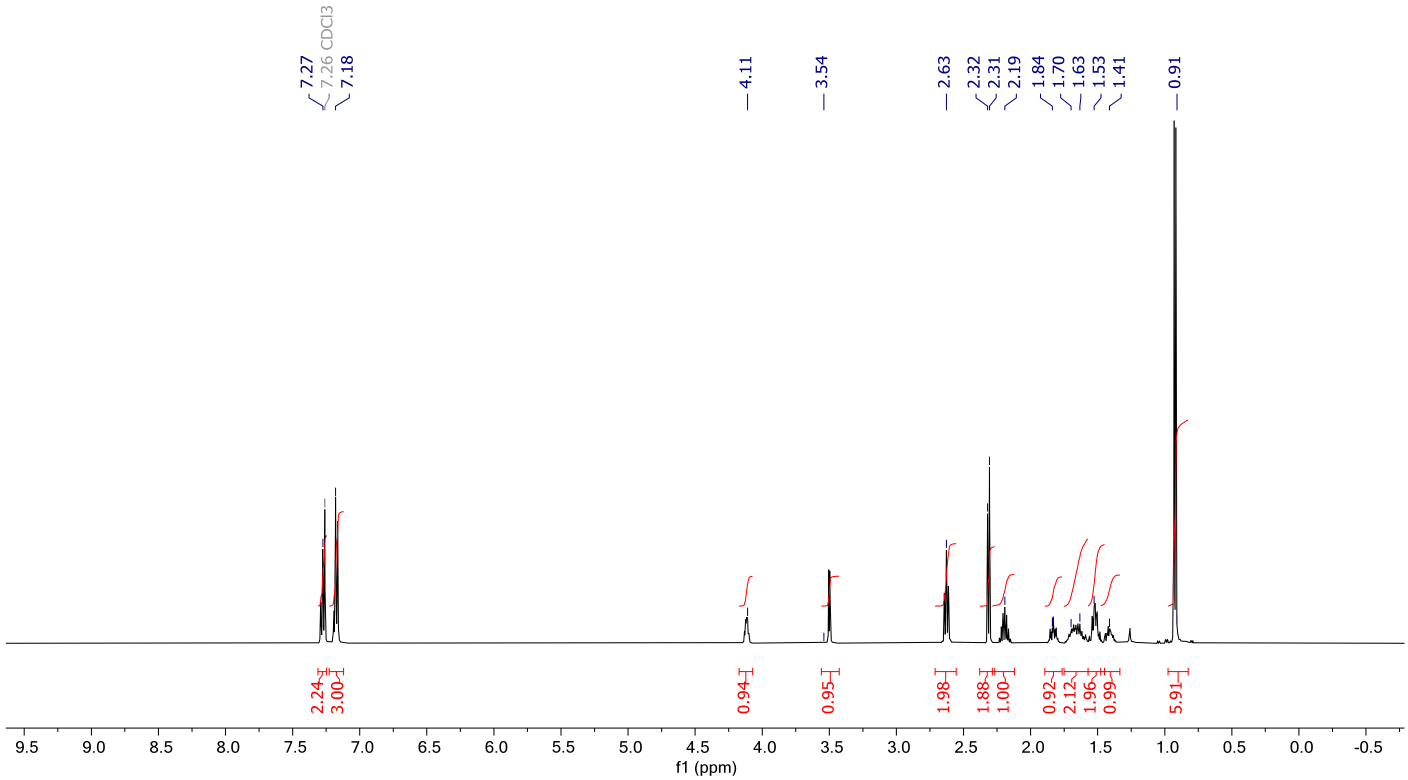

Figure S15. ^{1}H NMR spectrum for **5** (500 MHz, $CDCl_3$, room temperature).

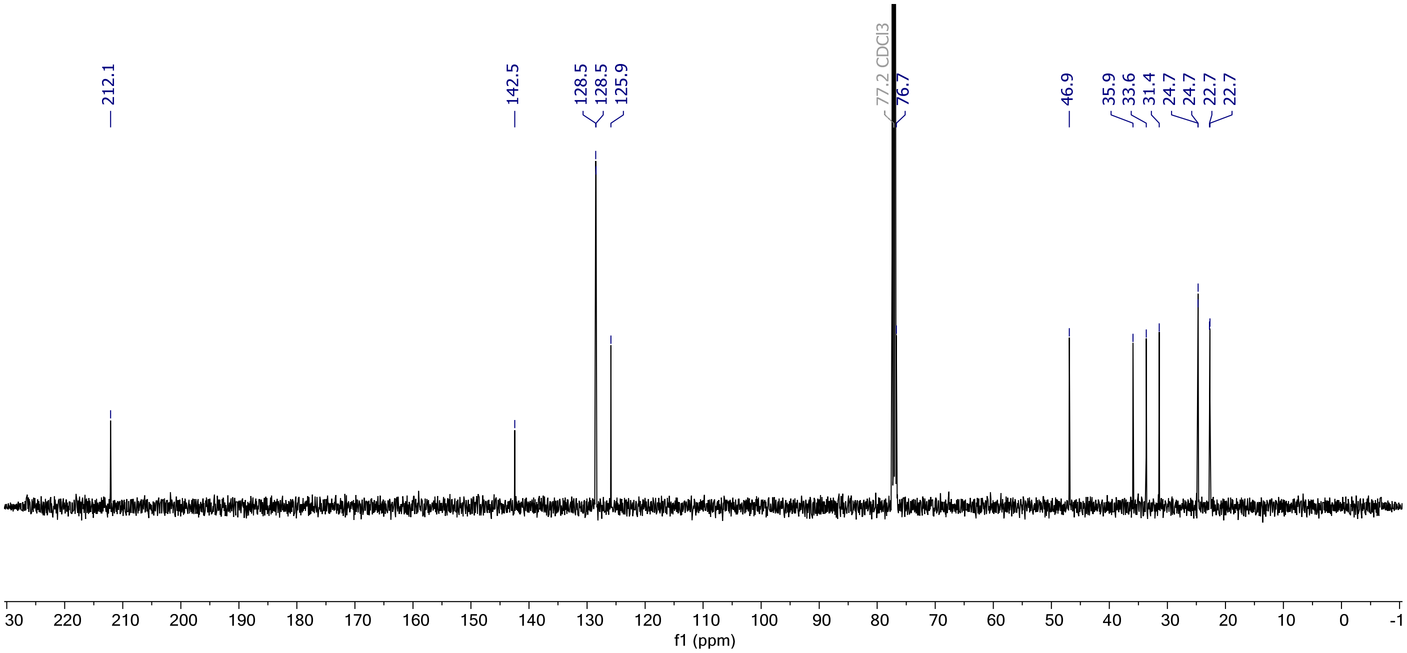

Figure S16. $^{13}C\{^1H\}$ NMR spectrum for **5** (126 MHz, $CDCl_3$, room temperature).

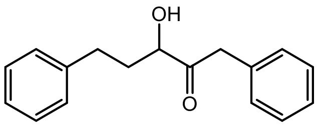

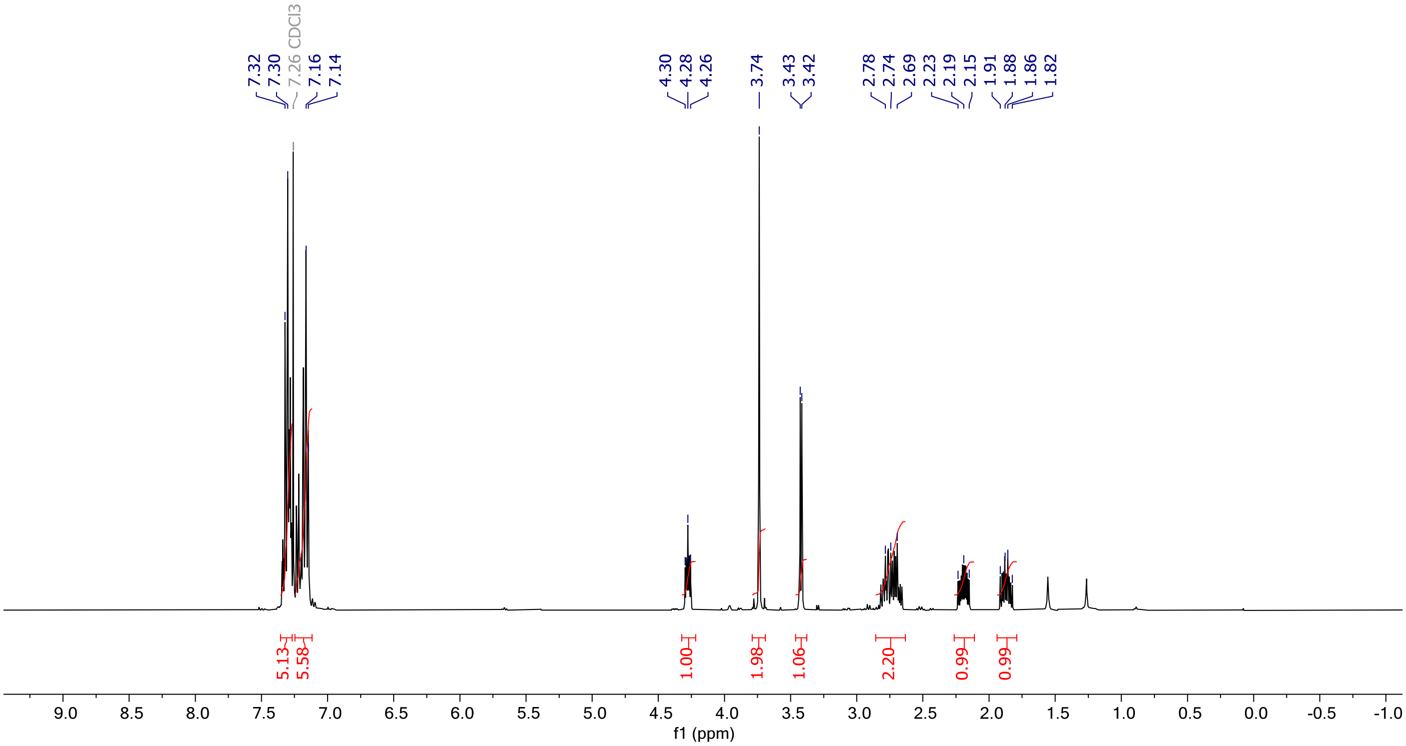

Figure S17. ^{1}H NMR spectrum for **13** (400 MHz, $CDCl_3$, room temperature).

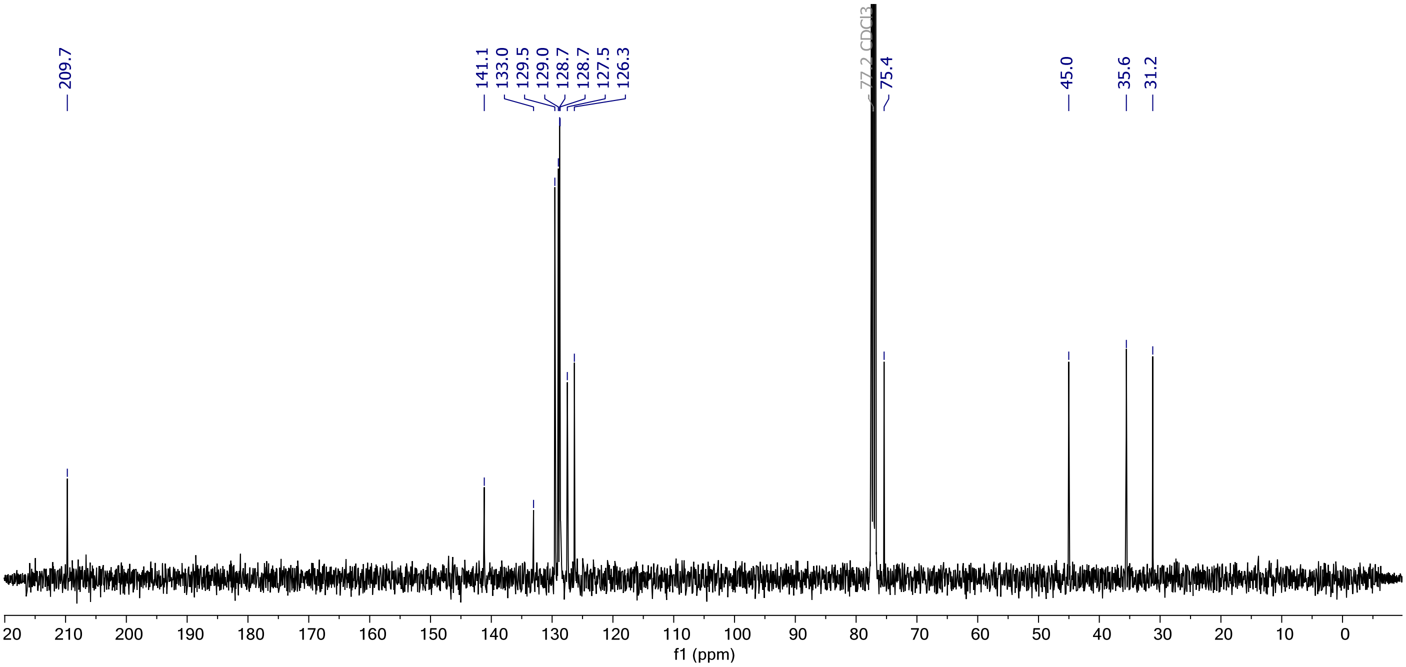

Figure S18. $^{13}C\{^{1}H\}$ NMR spectrum for **13** (101 MHz, $CDCl_3$, room temperature).

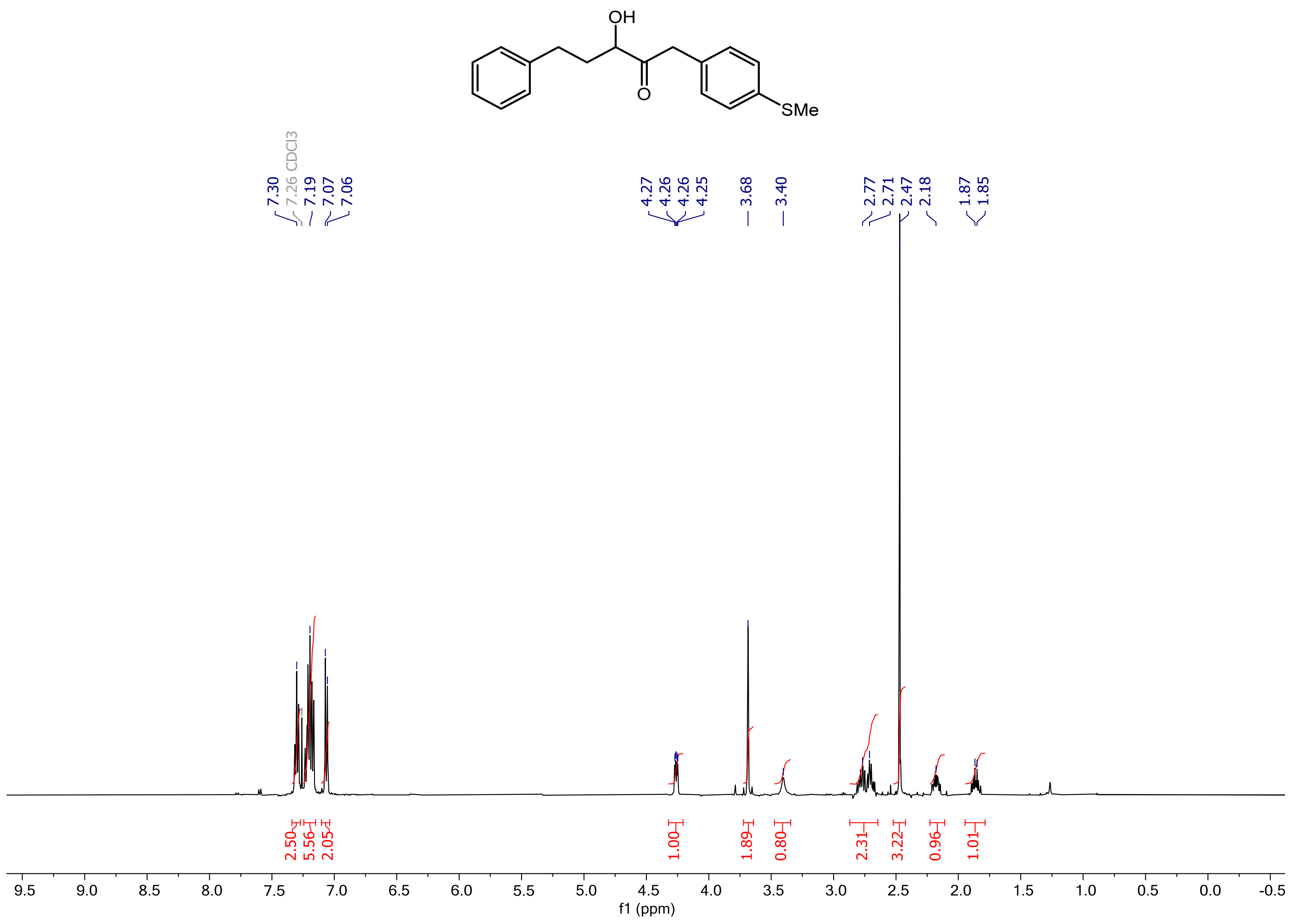

Figure S19. ^{1}H NMR spectrum for **14** (500 MHz, $CDCl_3$, room temperature).

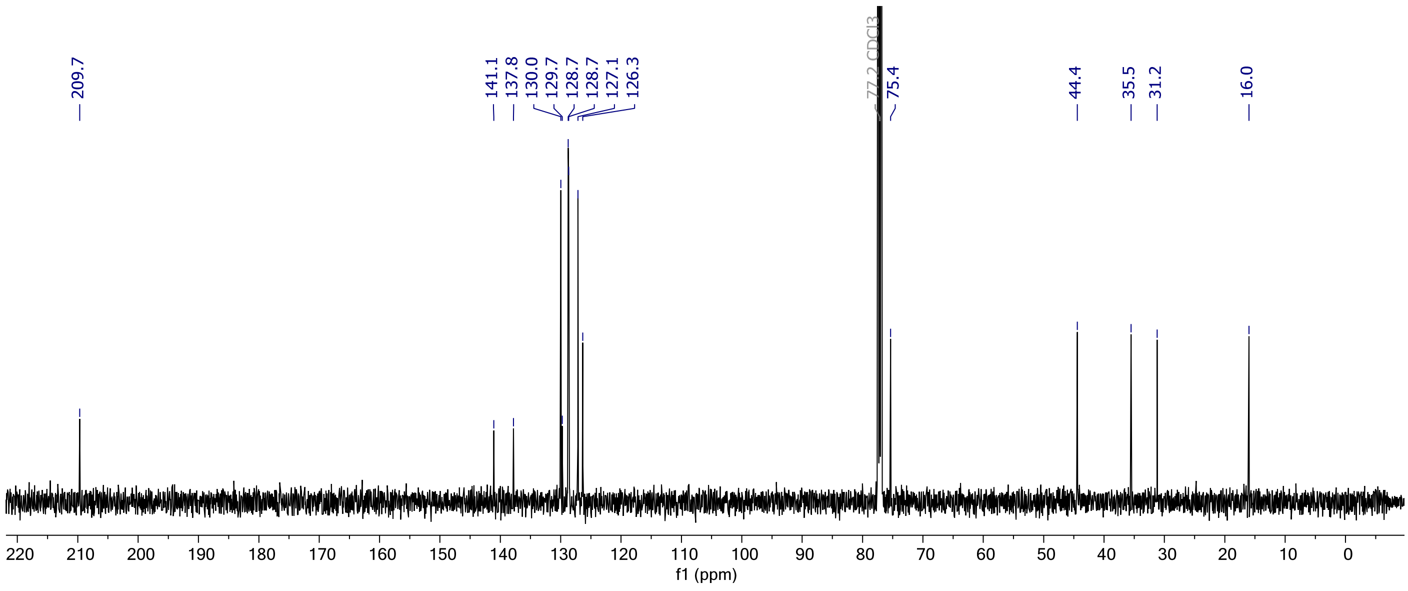

Figure S20. $^{13}C\{^{1}H\}$ NMR spectrum for **14** (126 MHz, $CDCl_3$, room temperature).

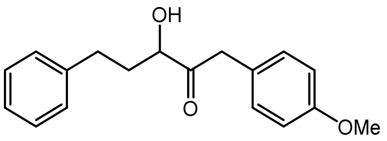

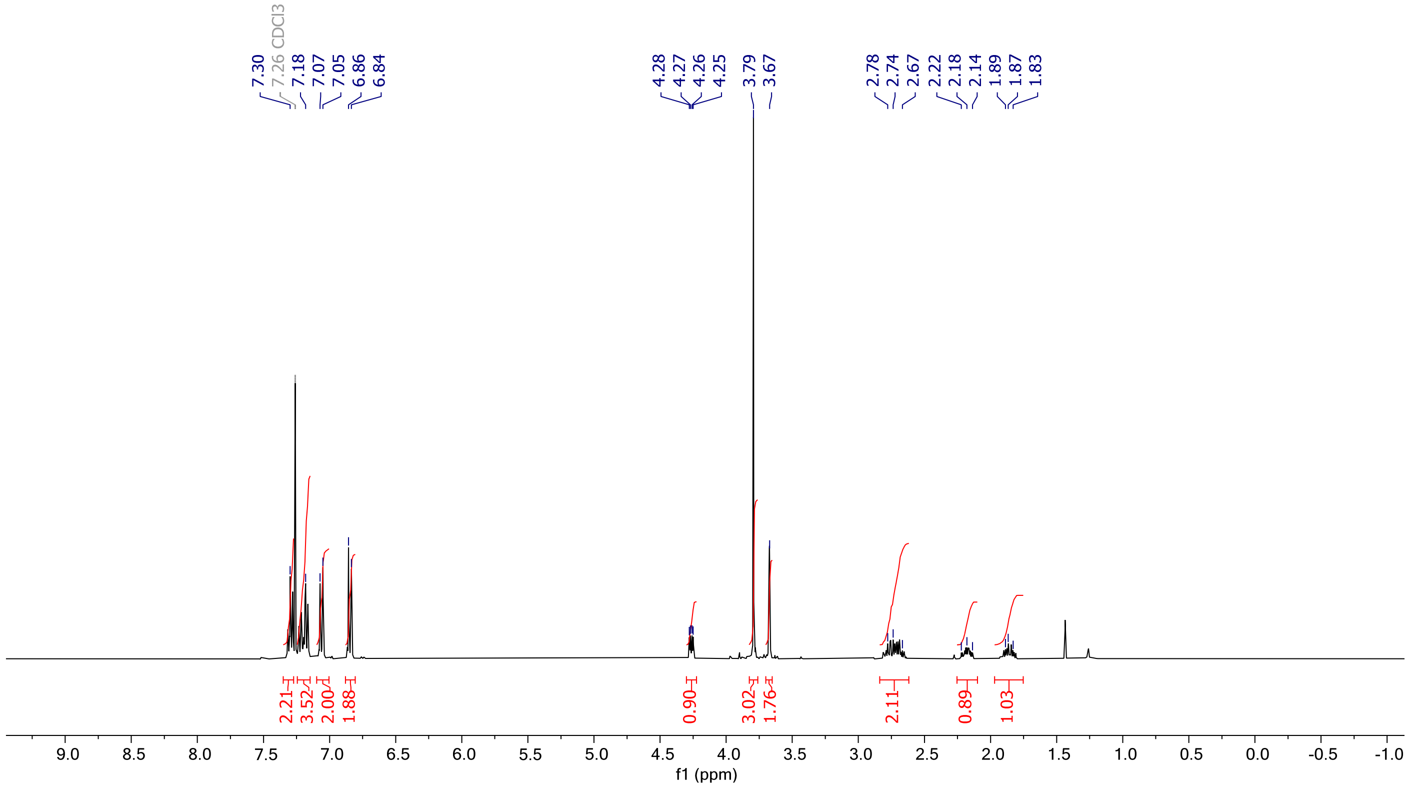

Figure S21. ^{1}H NMR spectrum for **15** (400 MHz, $CDCl_3$, room temperature).

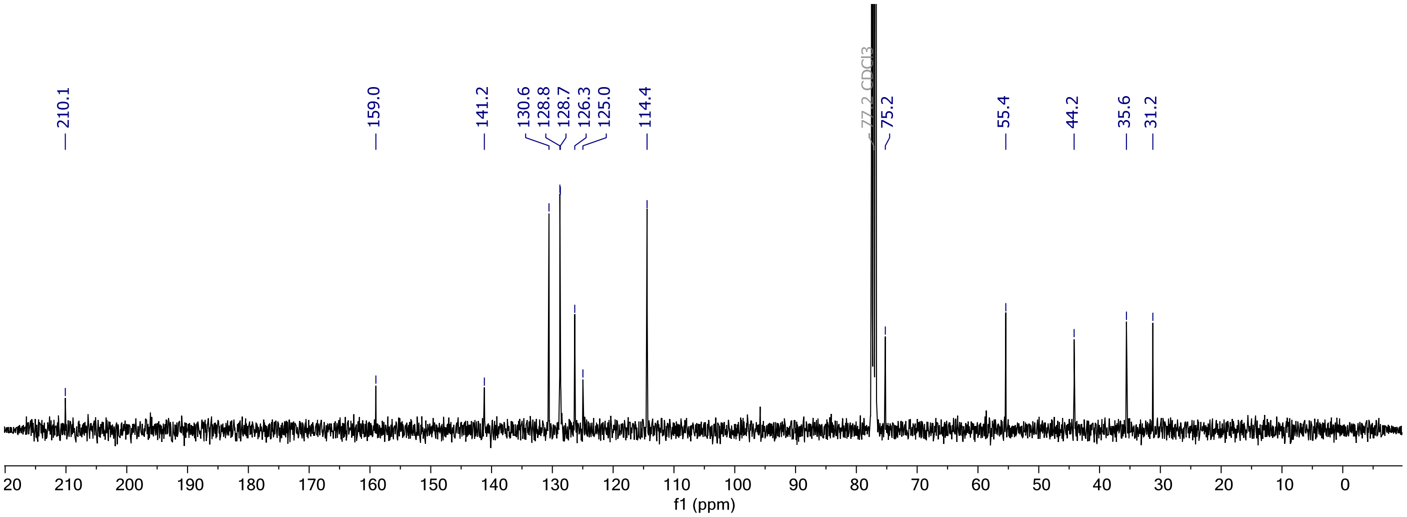

Figure S22. $^{13}C\{^{1}H\}$ NMR spectrum for **15** (101 MHz, $CDCl_3$, room temperature).

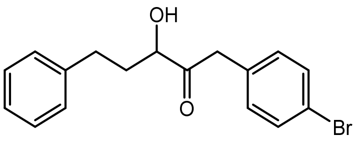

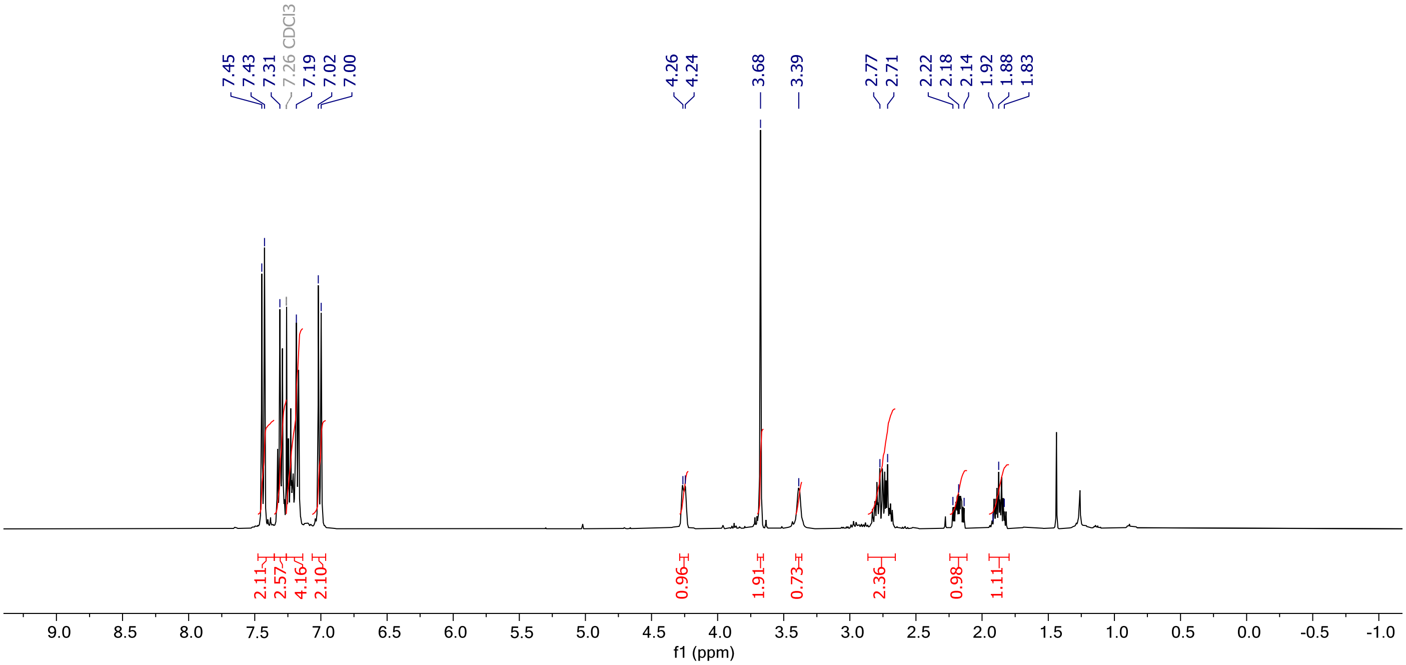

Figure S23. ^{1}H NMR spectrum for **16** (400 MHz, $CDCl_3$, room temperature).

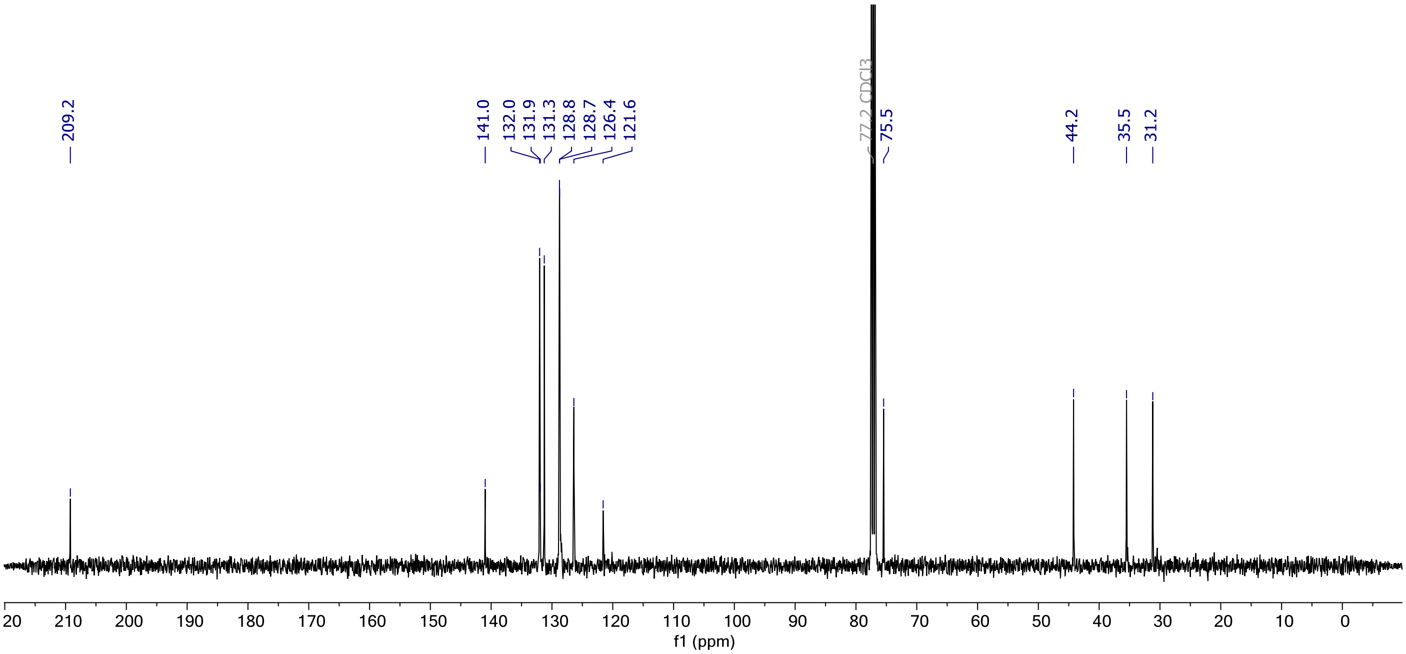

Figure S24. $^{13}C\{^{1}H\}$ NMR spectrum for **16** (101 MHz, $CDCl_3$, room temperature).

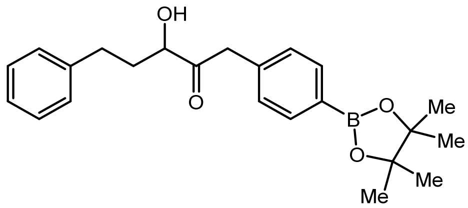

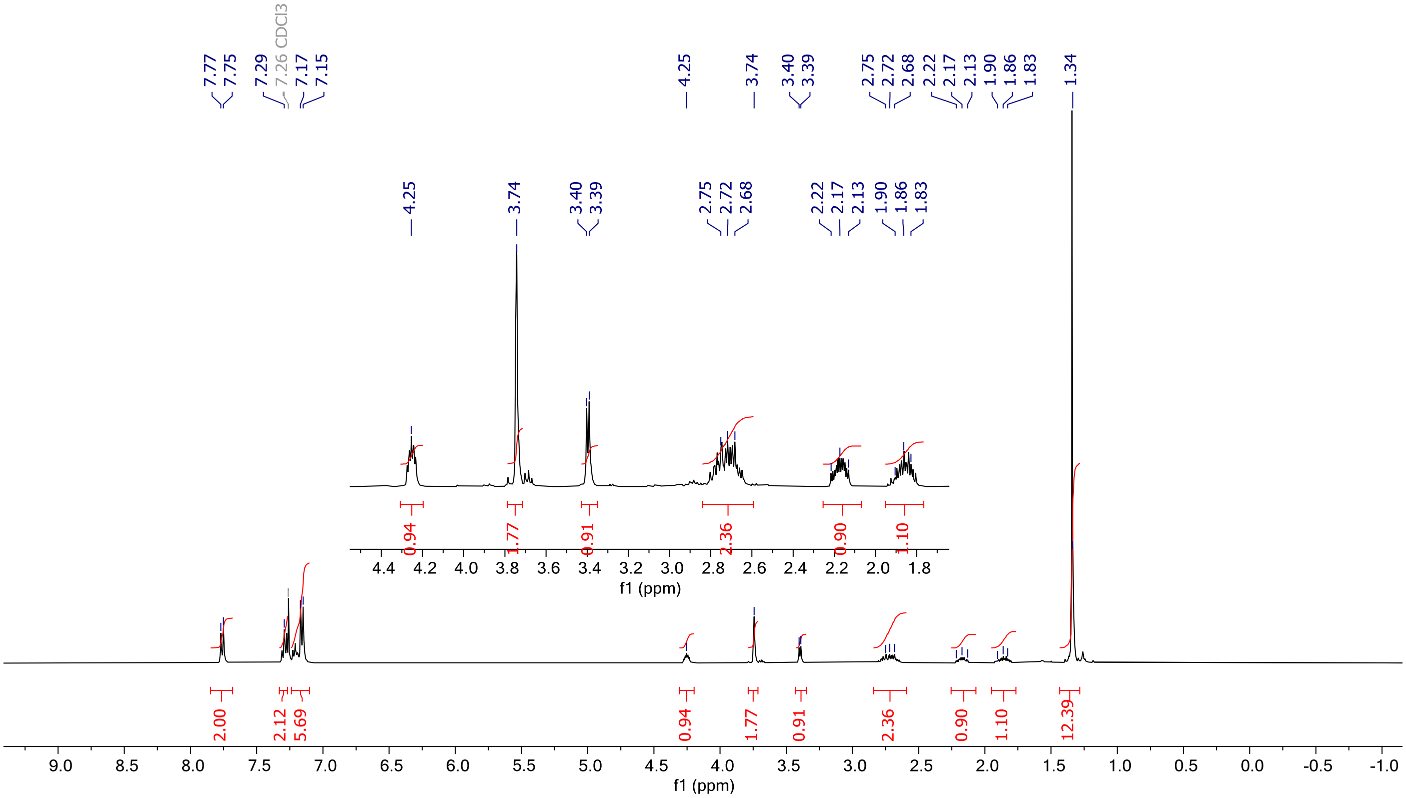

Figure S25. ^{1}H NMR spectrum for **17** (400 MHz, $CDCl_3$, room temperature).

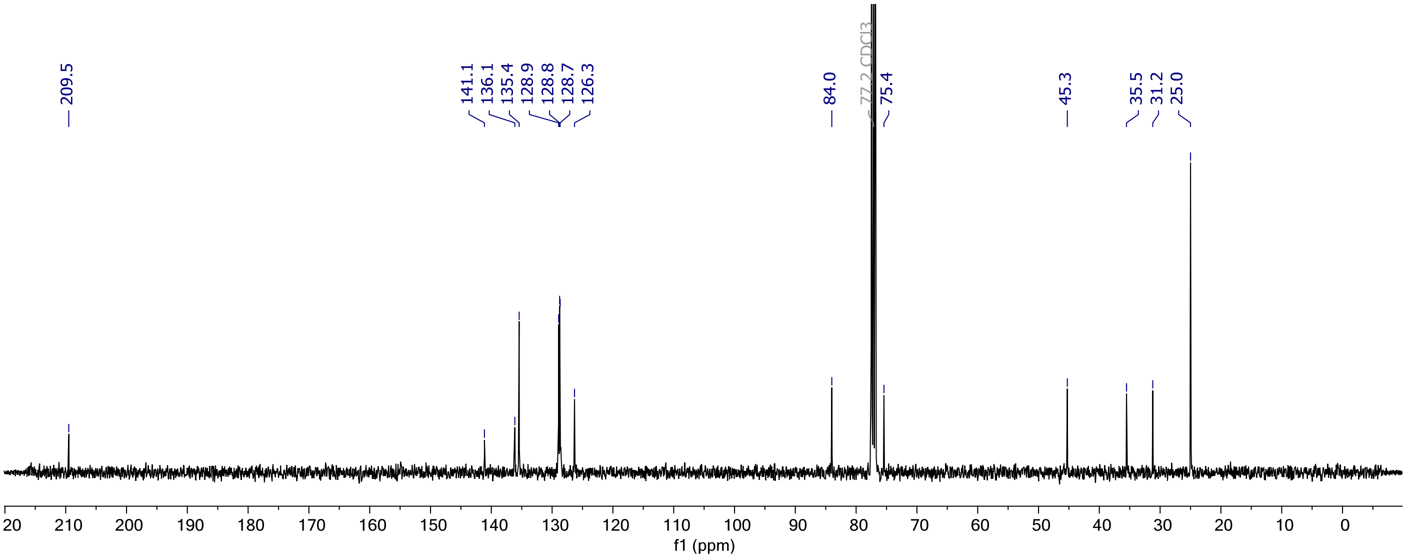

Figure S26. $^{13}C\{^{1}H\}$ NMR spectrum for **17** (101 MHz, $CDCl_3$, room temperature).

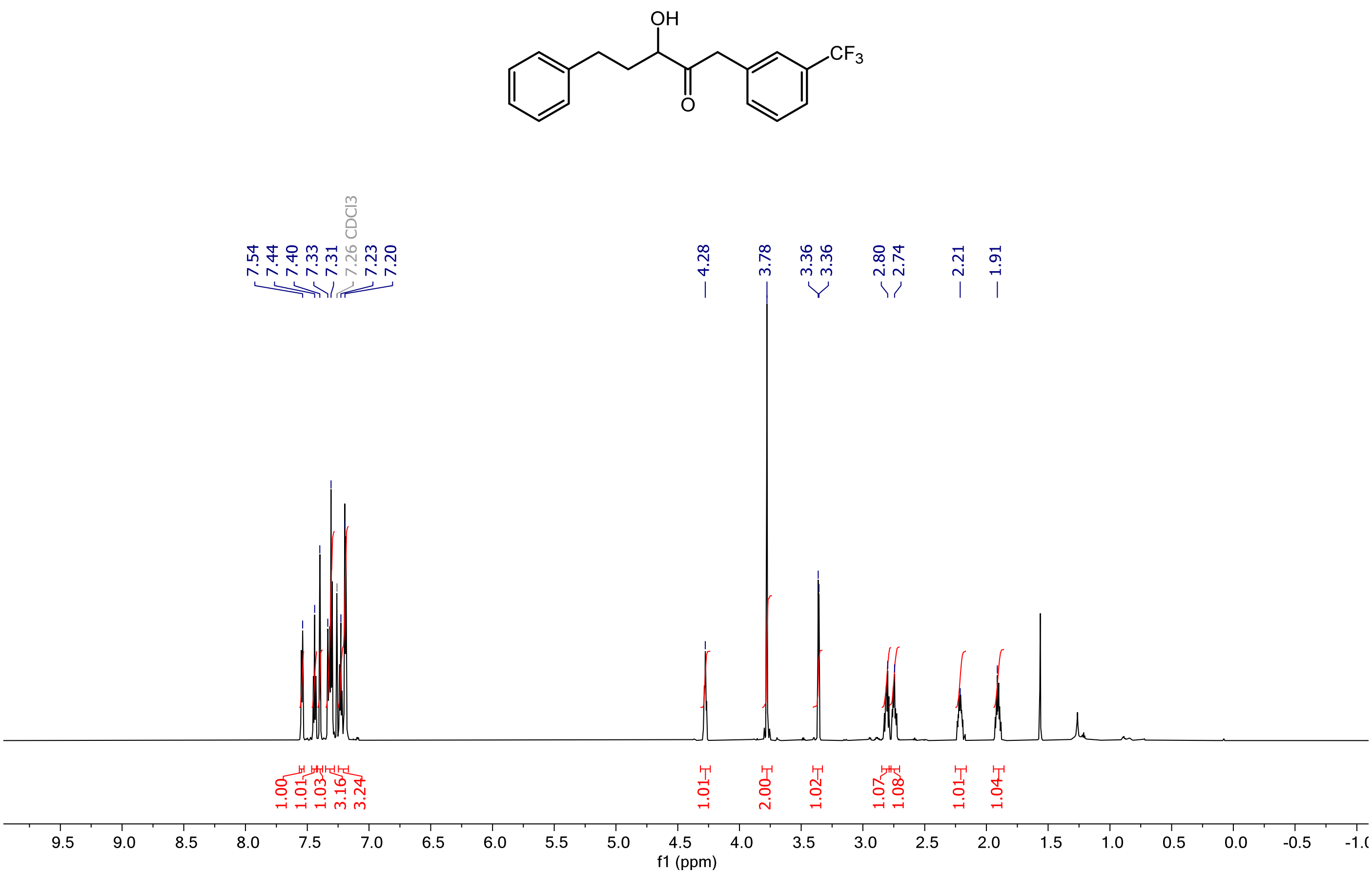

Figure S27. ^{1}H NMR spectrum for **18** (800 MHz, $CDCl_3$, room temperature).

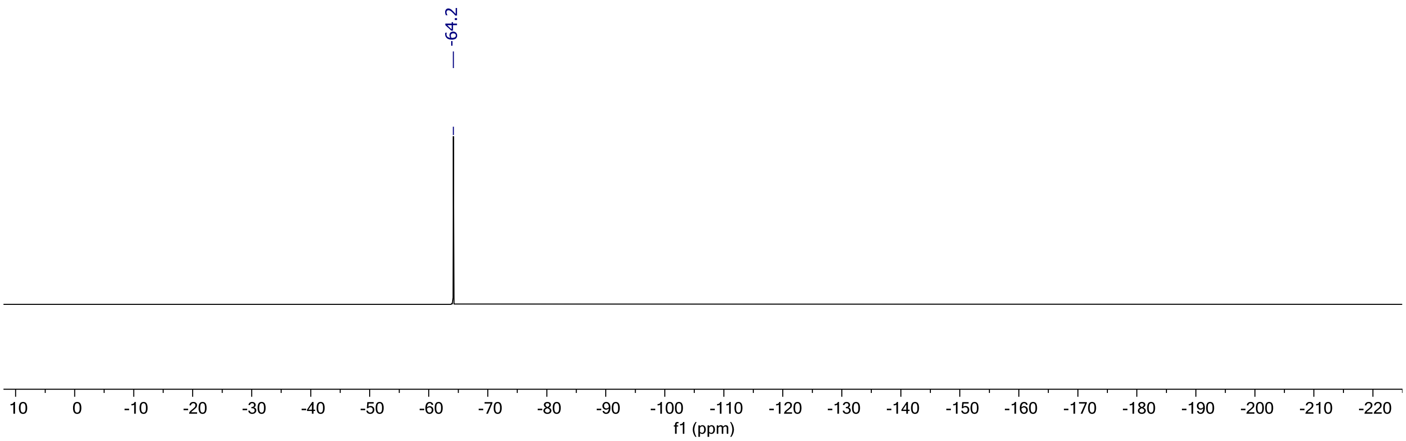

Figure S28. ^{19}F NMR spectrum for **18** (376 MHz, $CDCl_3$, room temperature).

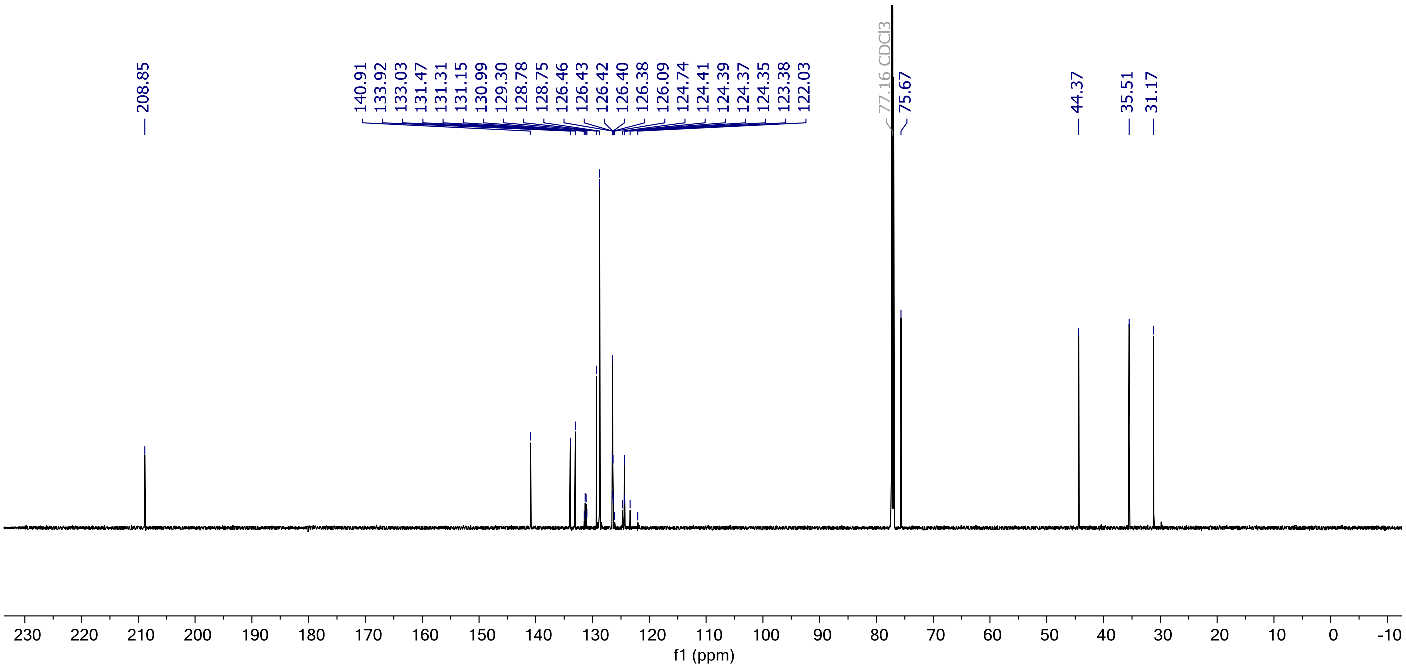

Figure S29. $^{13}C\{^{1}H\}$ NMR spectrum for **18** (201 MHz, $CDCl_3$, room temperature).

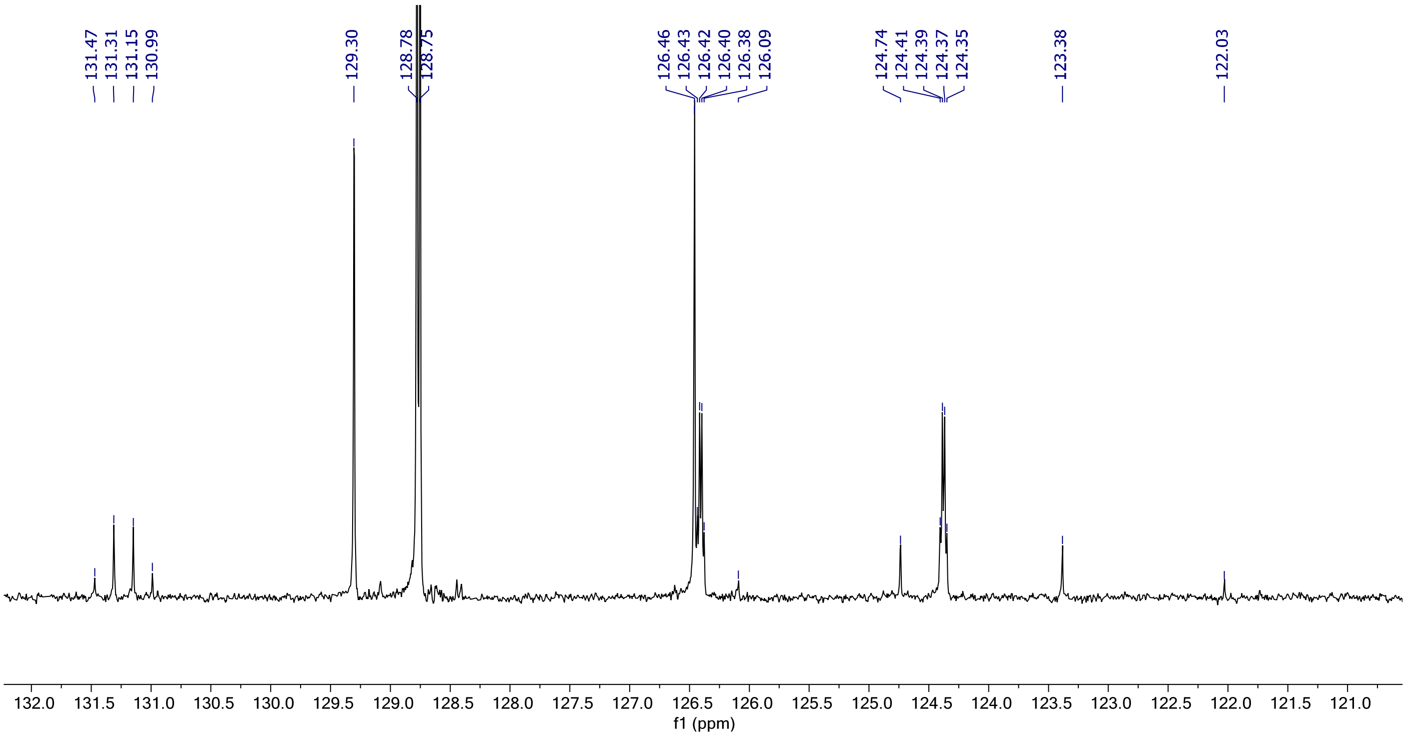

Figure S30. $^{13}C\{^{1}H\}$ NMR spectrum for **18** (201 MHz, $CDCl_3$, room temperature).

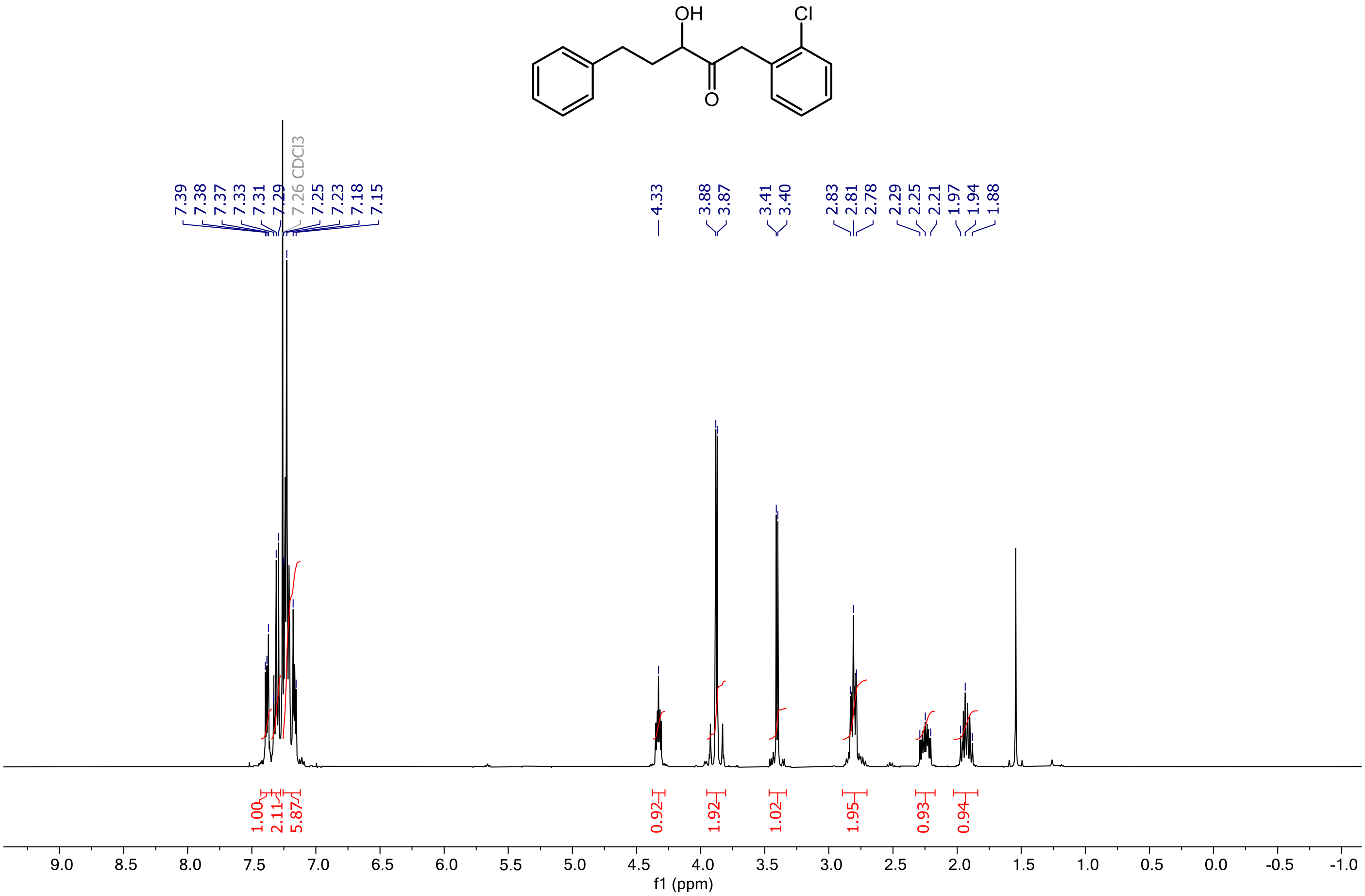

Figure S31. ^{1}H NMR spectrum for **19** (400 MHz, $CDCl_3$, room temperature).

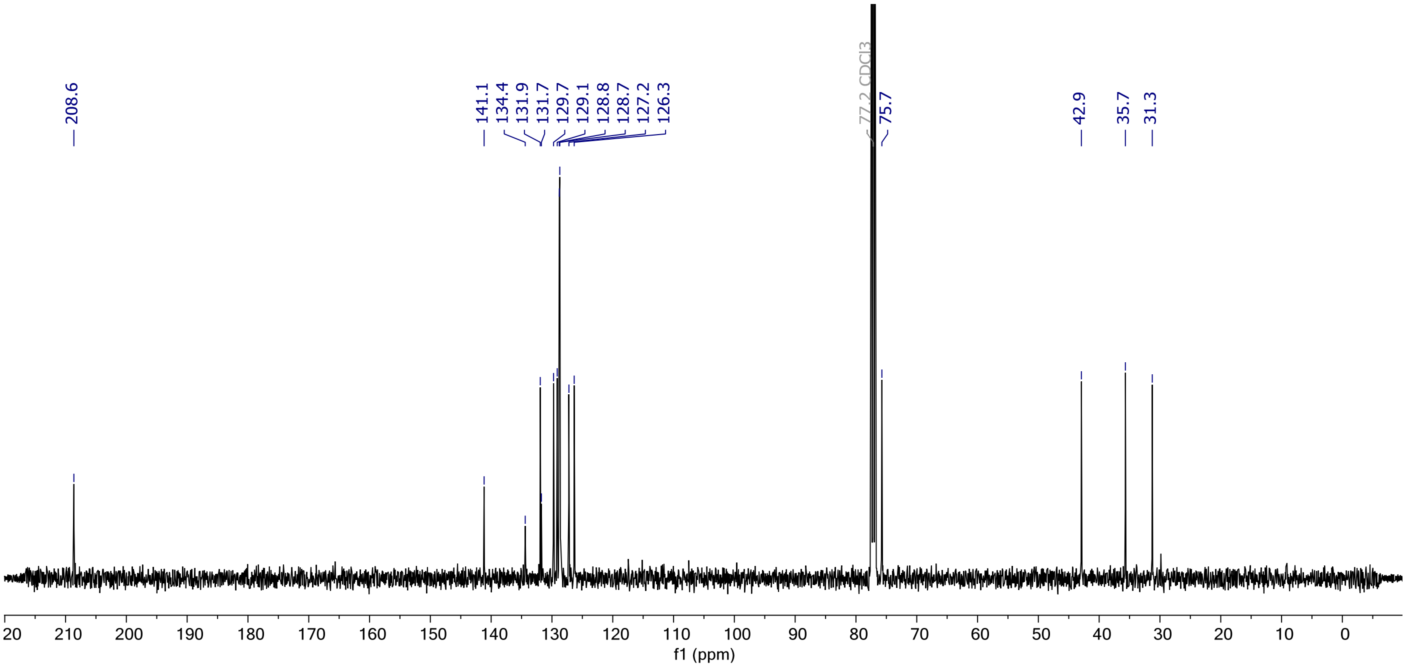

Figure S32. $^{13}C\{^{1}H\}$ NMR spectrum for **19** (101 MHz, $CDCl_3$, room temperature).

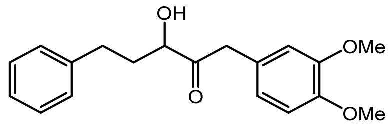

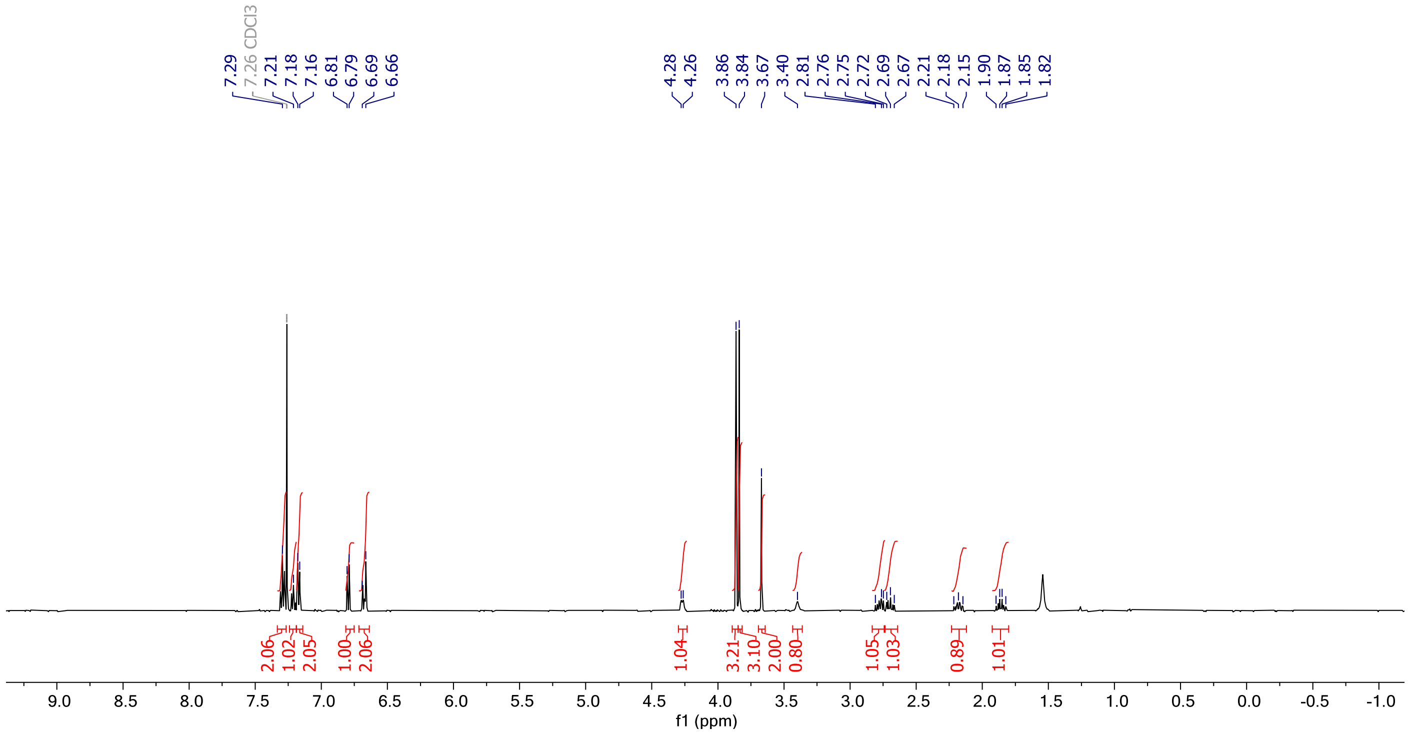

Figure S33. ^{1}H NMR spectrum for **20** (500 MHz, $CDCl_3$, room temperature).

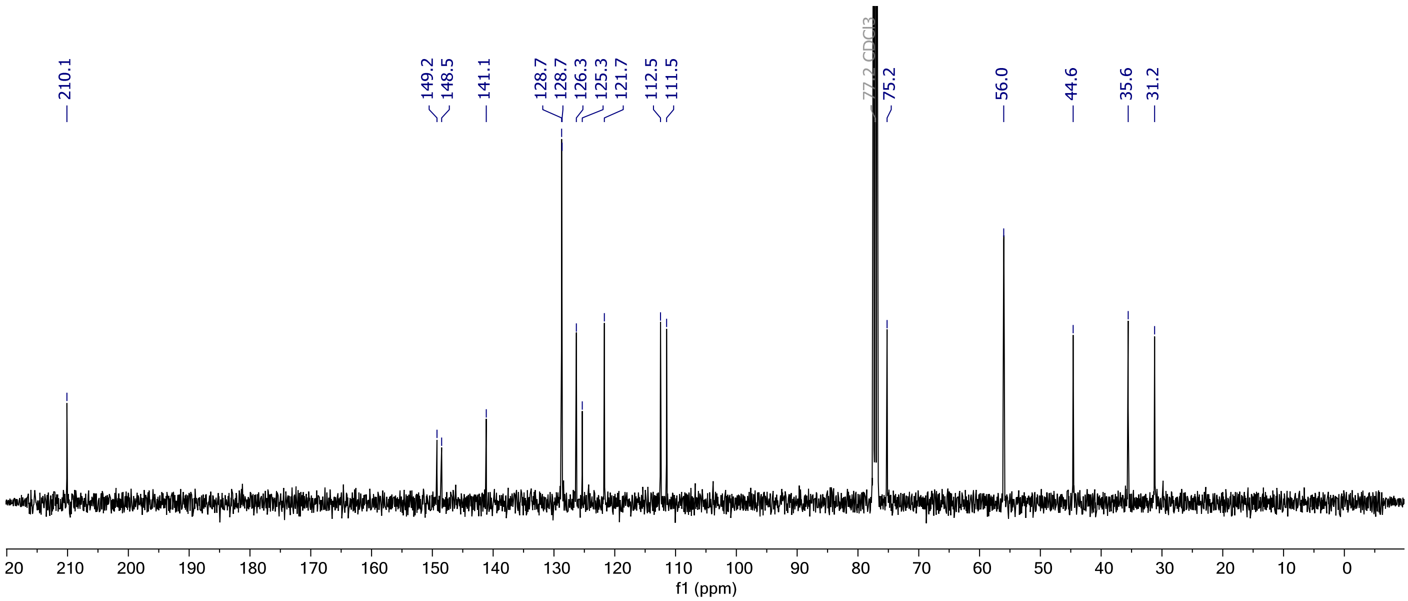

Figure S34. $^{13}C\{^{1}H\}$ NMR spectrum for **20** (101 MHz, $CDCl_3$, room temperature).

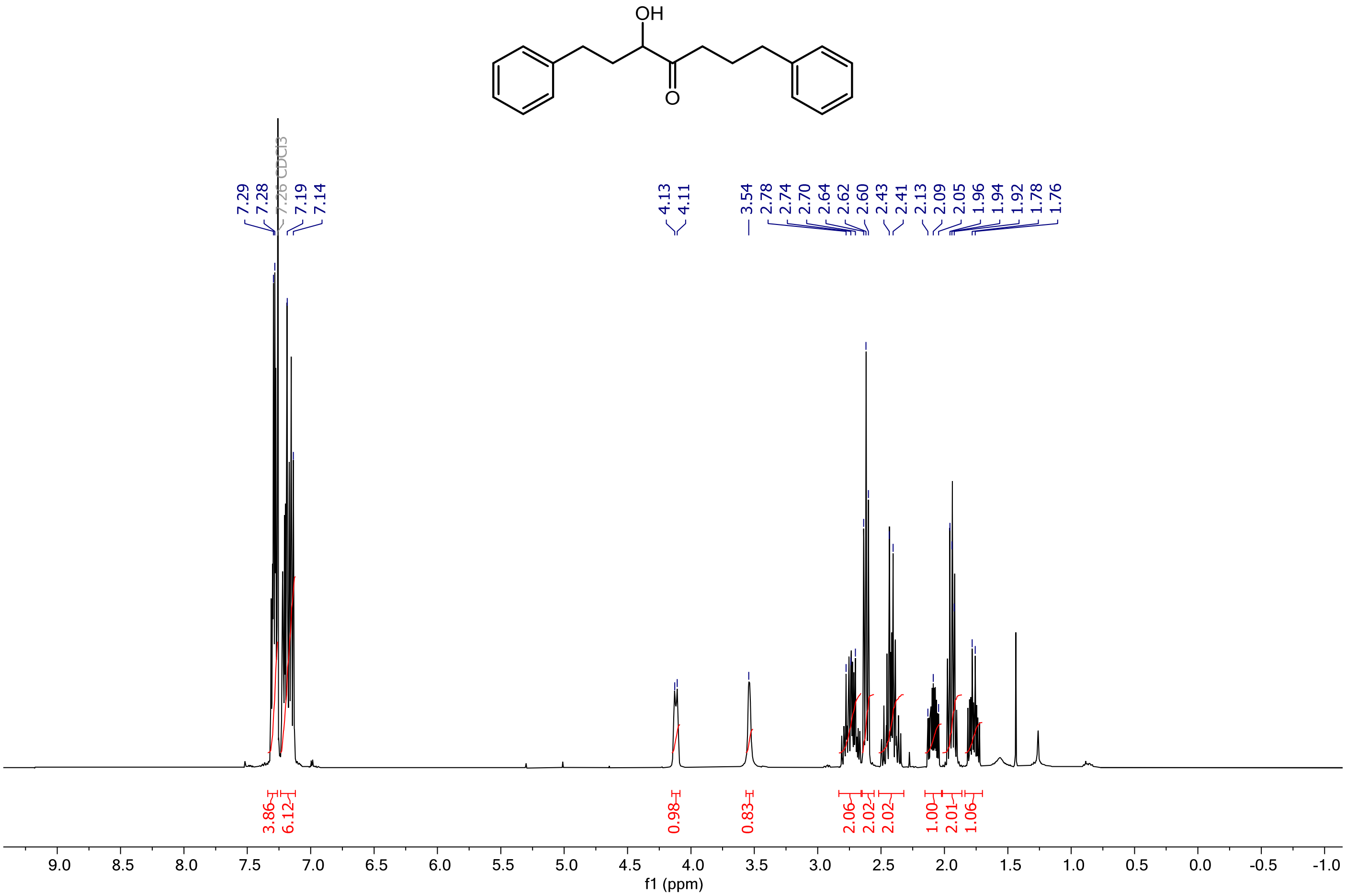

Figure S35. ^{1}H NMR spectrum for **21** (400 MHz, $CDCl_3$, room temperature).

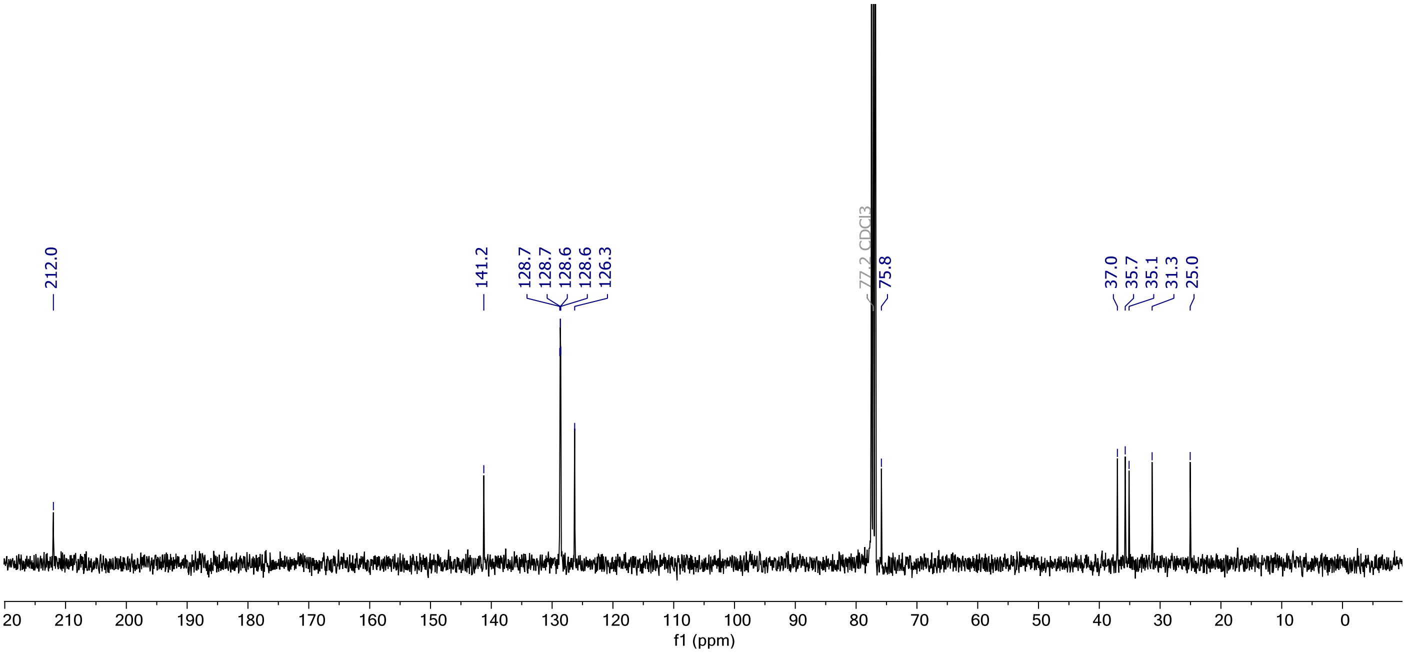

Figure S36. $^{13}C\{^{1}H\}$ NMR spectrum for **21** (101 MHz, $CDCl_3$, room temperature).

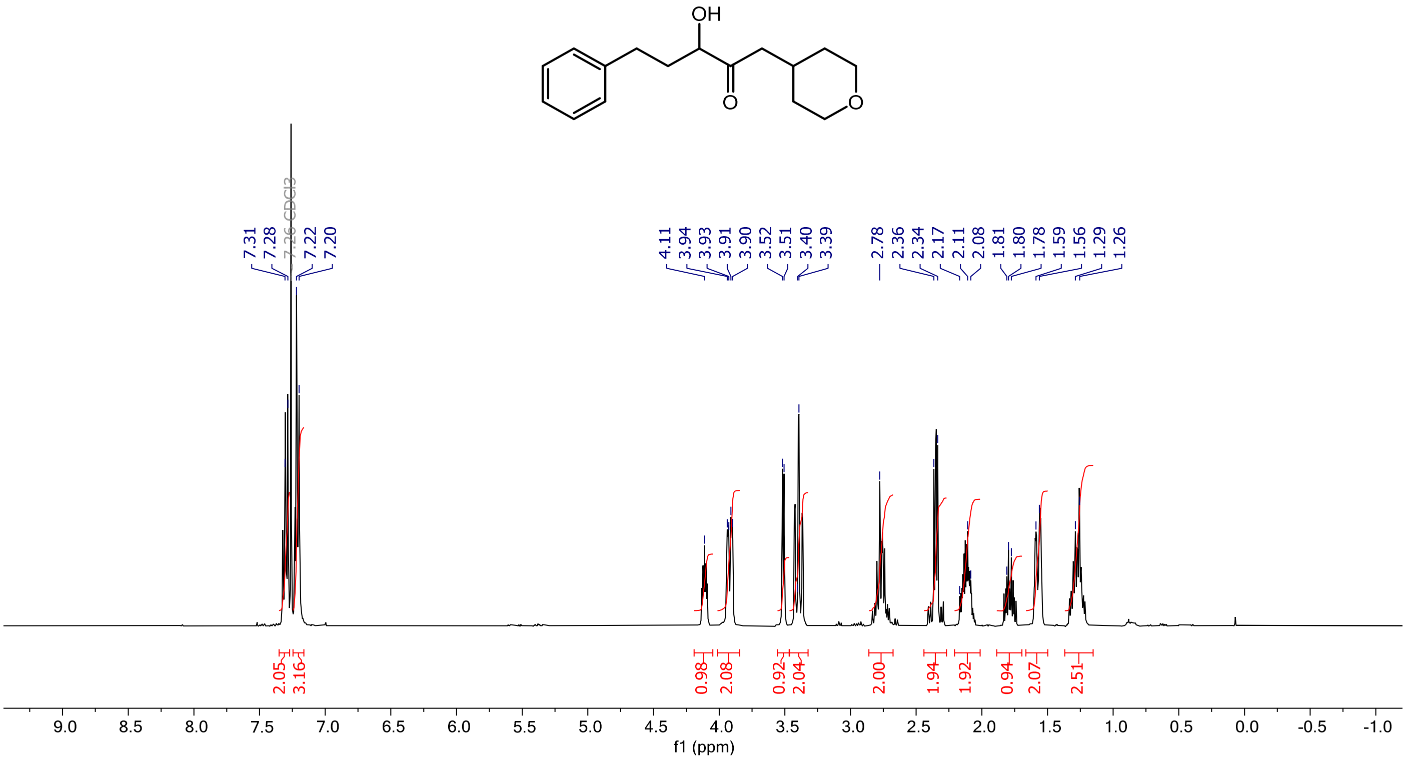

Figure S37. ^{1}H NMR spectrum for **22** (400 MHz, $CDCl_3$, room temperature).

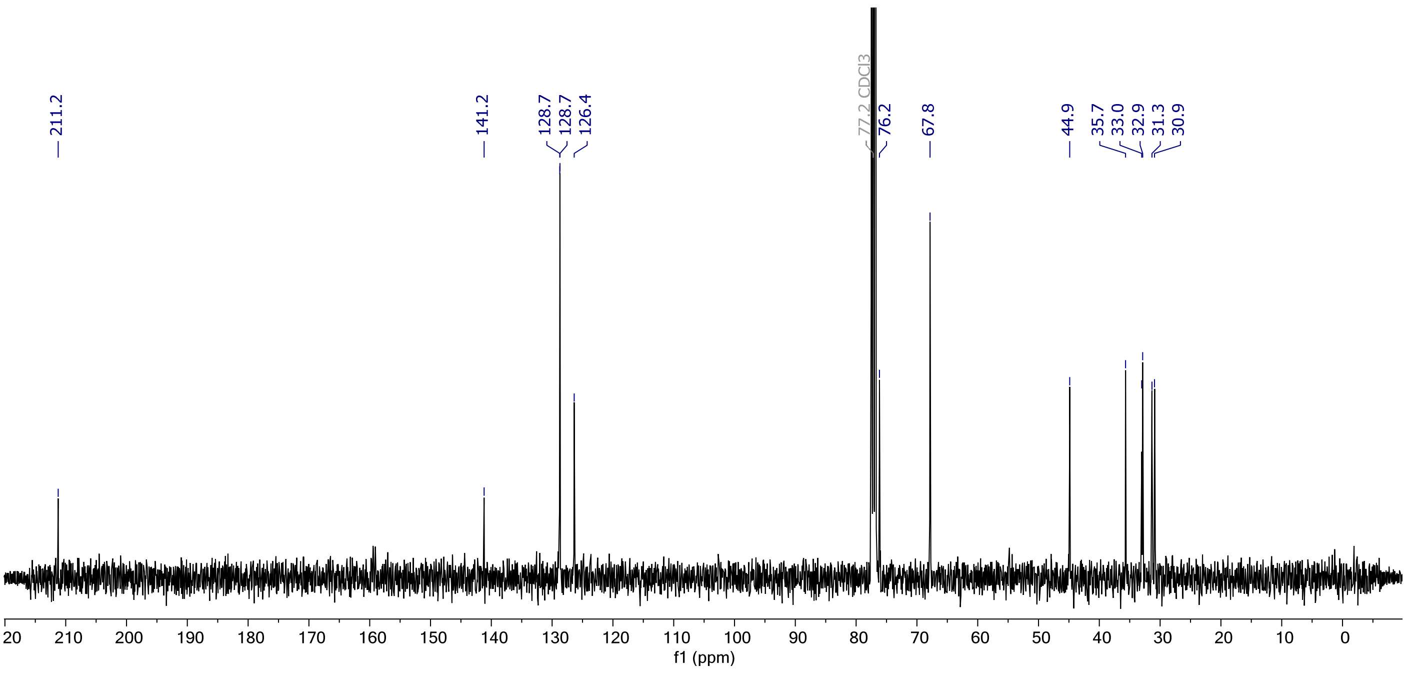

Figure S38. $^{13}C\{^1H\}$ NMR spectrum for **22** (101 MHz, $CDCl_3$, room temperature).

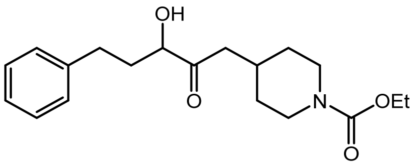

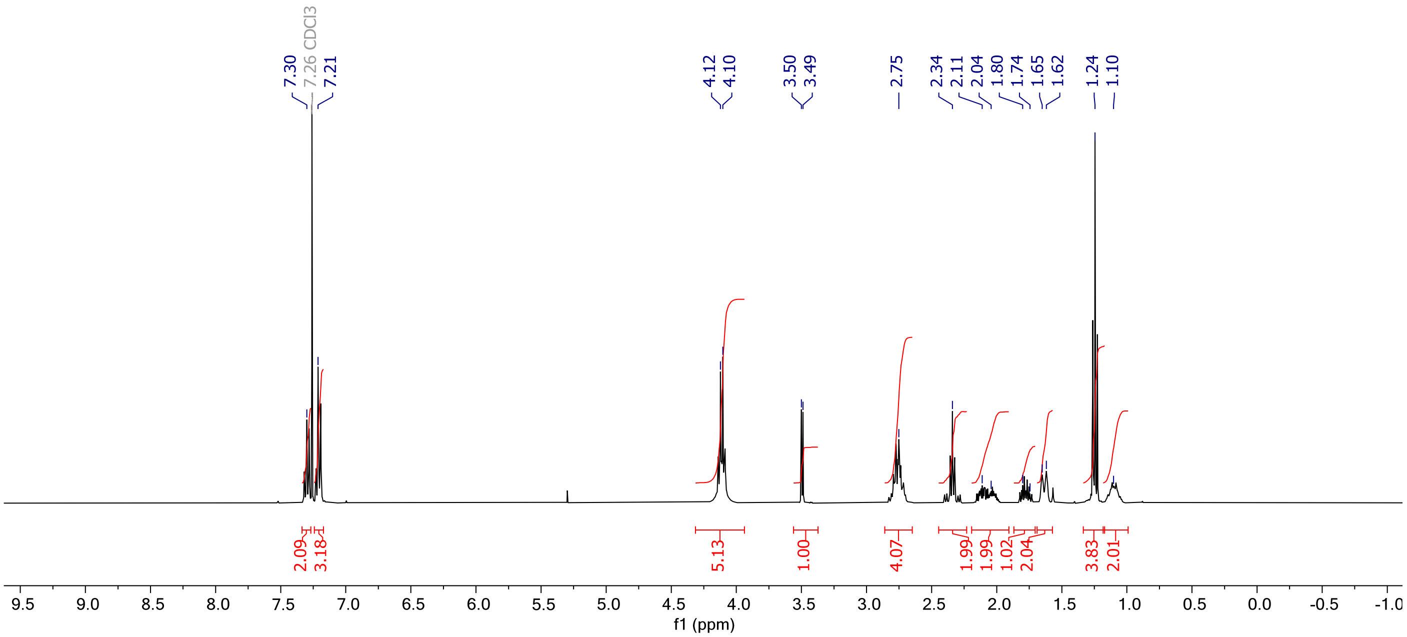

Figure S39. ^{1}H NMR spectrum for **23** (400 MHz, $CDCl_3$, room temperature).

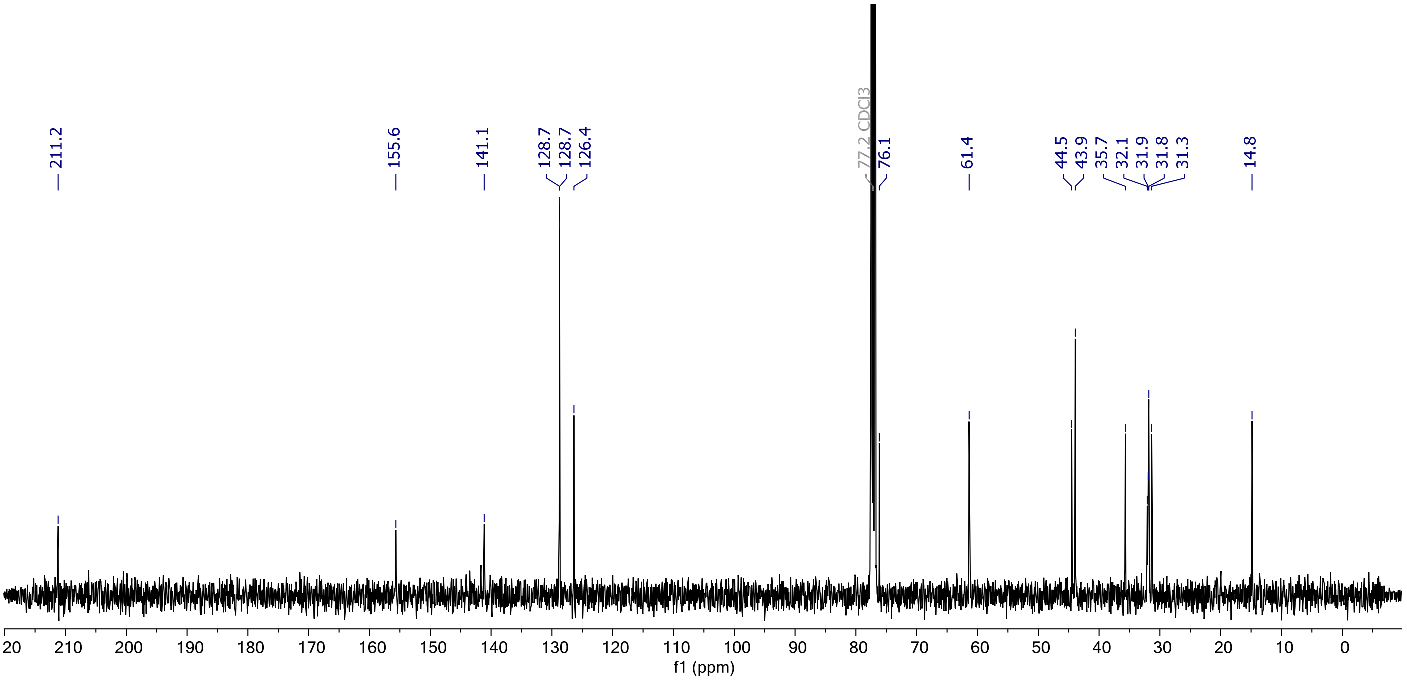

Figure S40. $^{13}C\{^{1}H\}$ NMR spectrum for **23** (101 MHz, $CDCl_3$, room temperature).

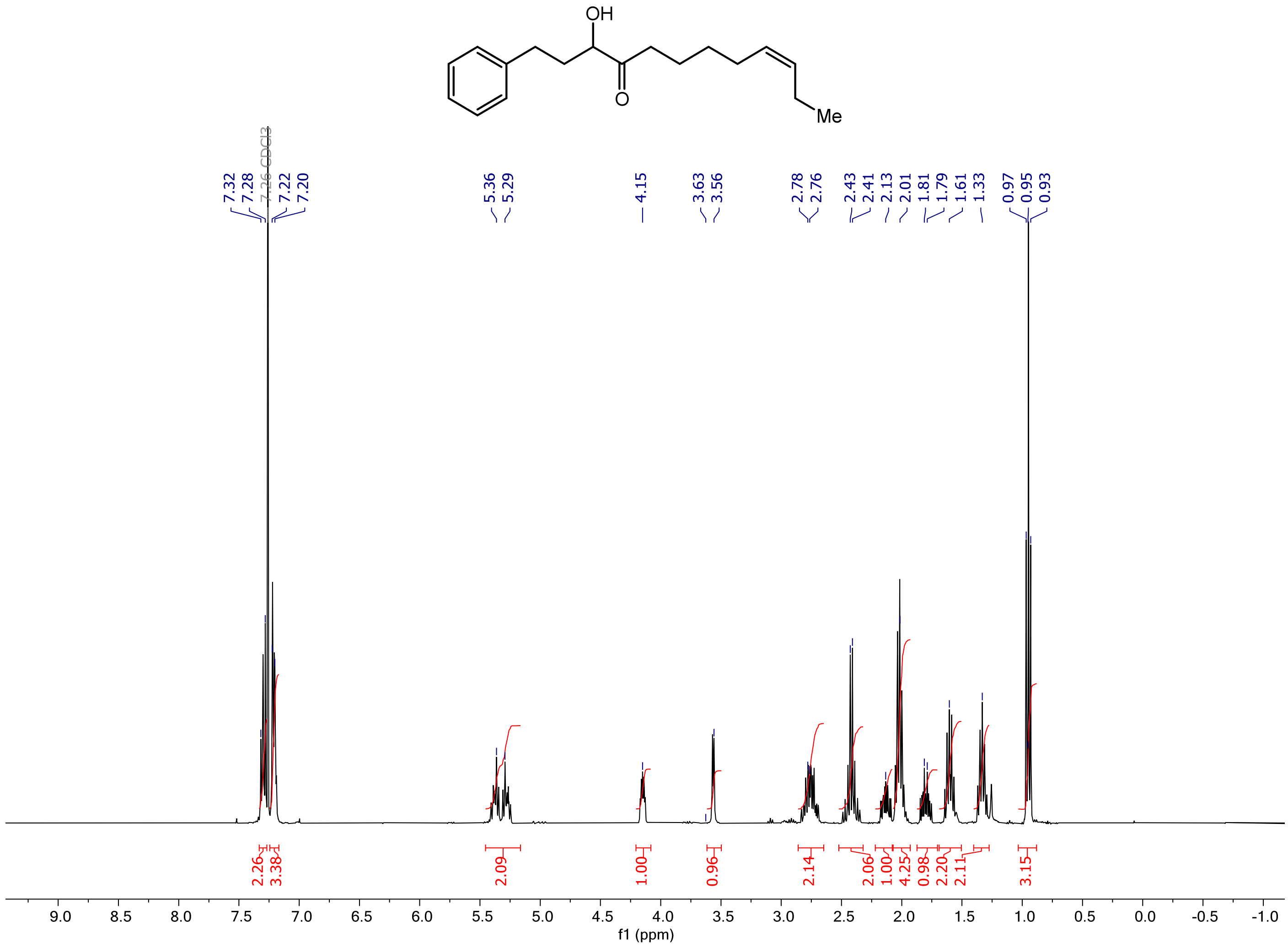

Figure S41. ^{1}H NMR spectrum for **24** (400 MHz, $CDCl_3$, room temperature).

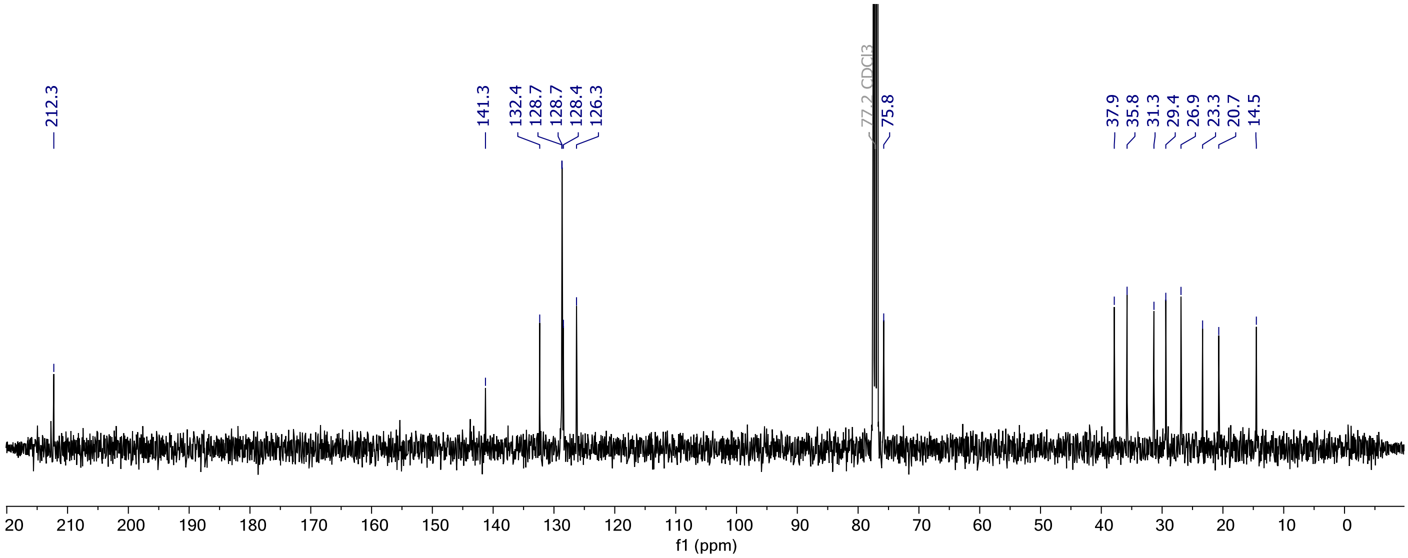

Figure S42. $^{13}C\{^{1}H\}$ NMR spectrum for **24** (101 MHz, $CDCl_3$, room temperature).

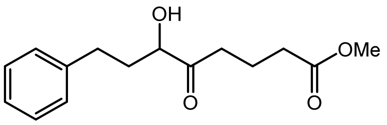

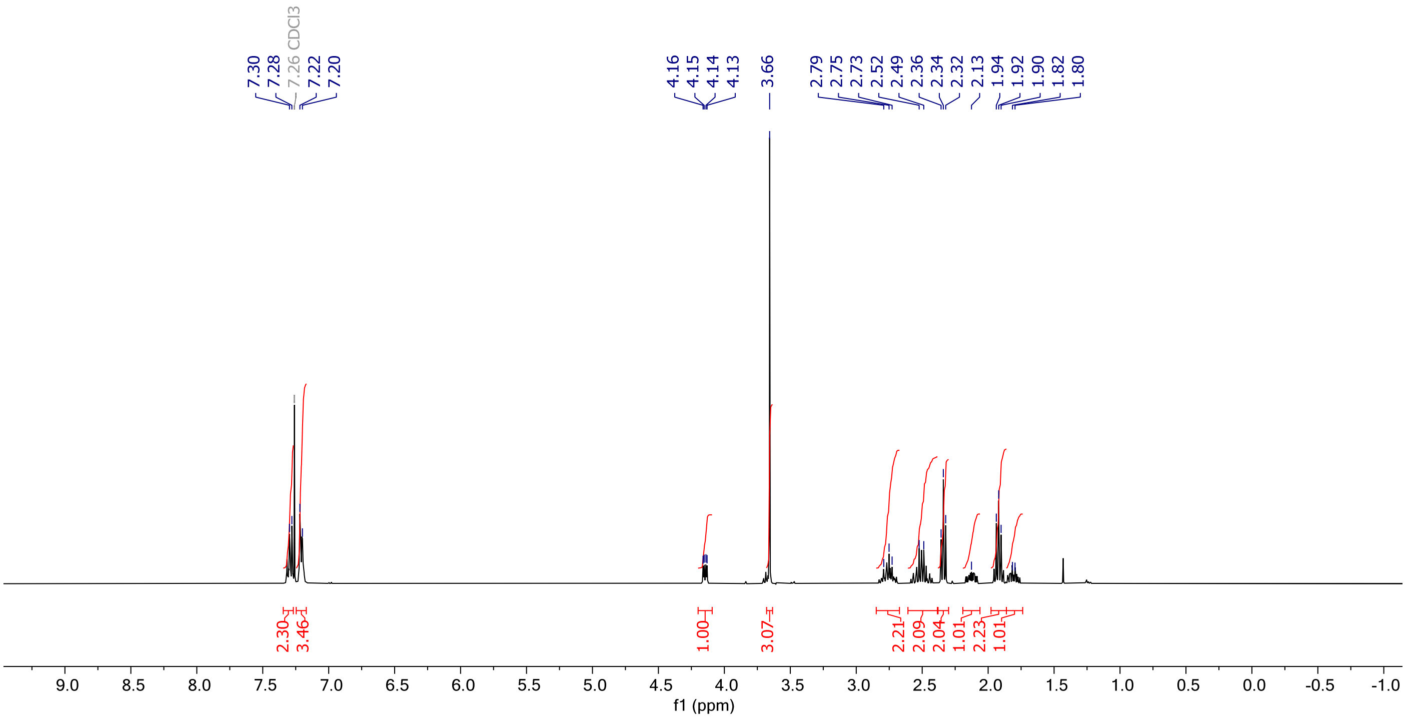

Figure S43. ^{1}H NMR spectrum for **25** (400 MHz, $CDCl_3$, room temperature).

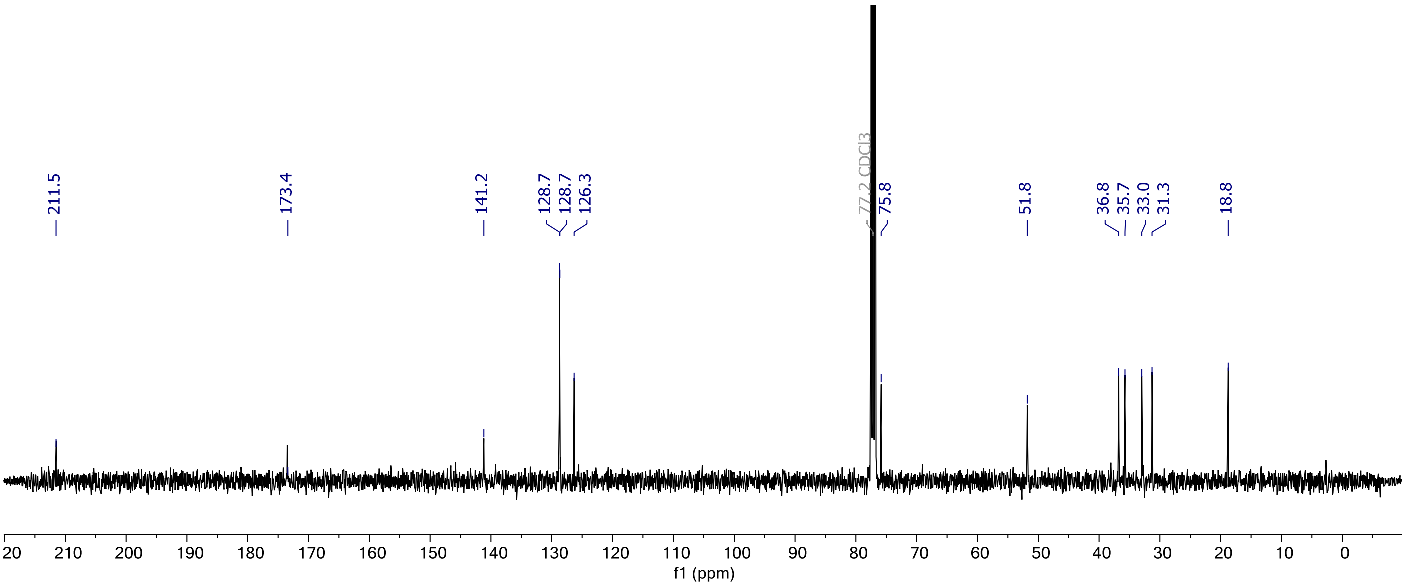

Figure S44. $^{13}C\{^{1}H\}$ NMR spectrum for **25** (101 MHz, $CDCl_3$, room temperature).

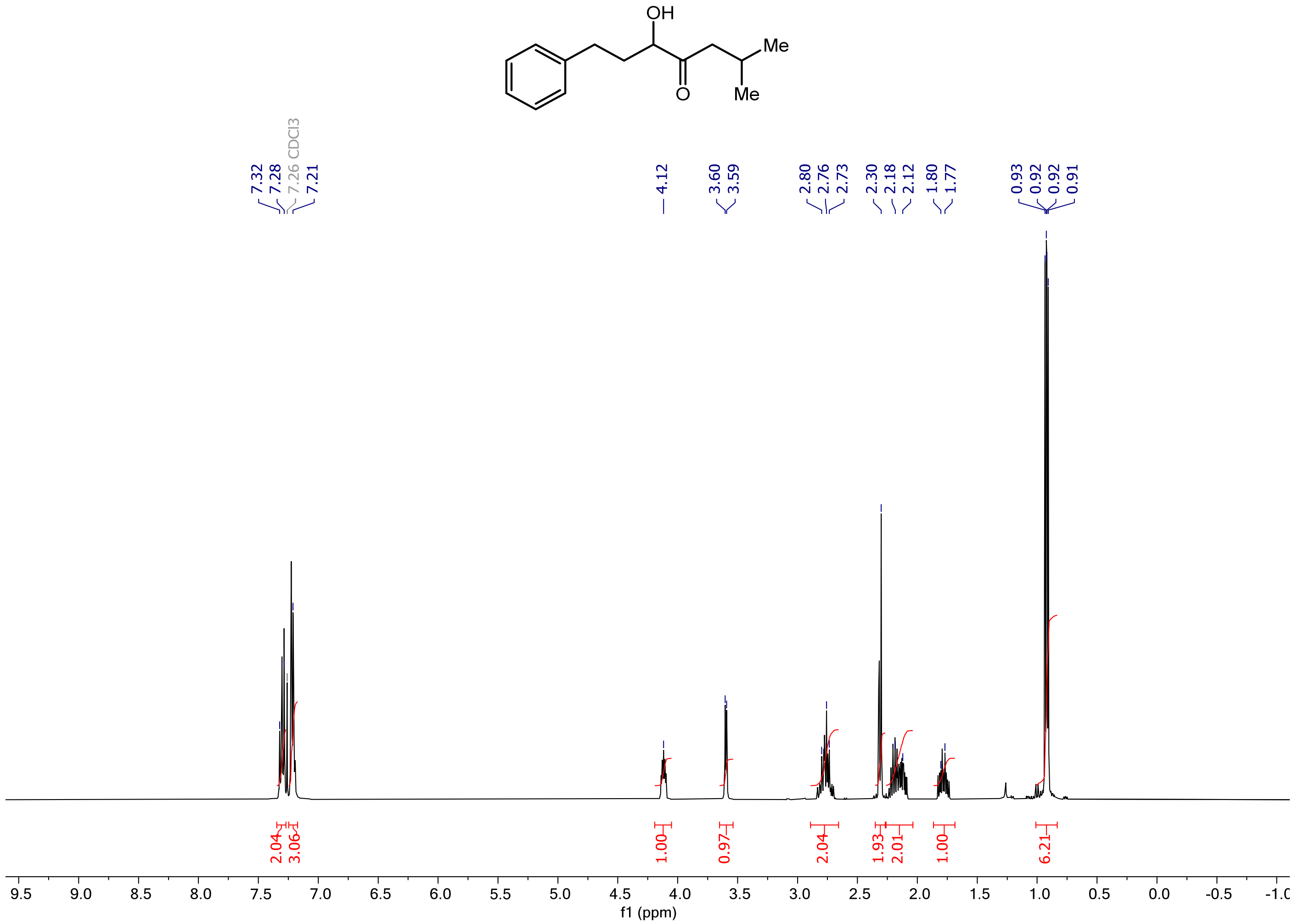

Figure S45. ^{1}H NMR spectrum for **26** (400 MHz, $CDCl_3$, room temperature).

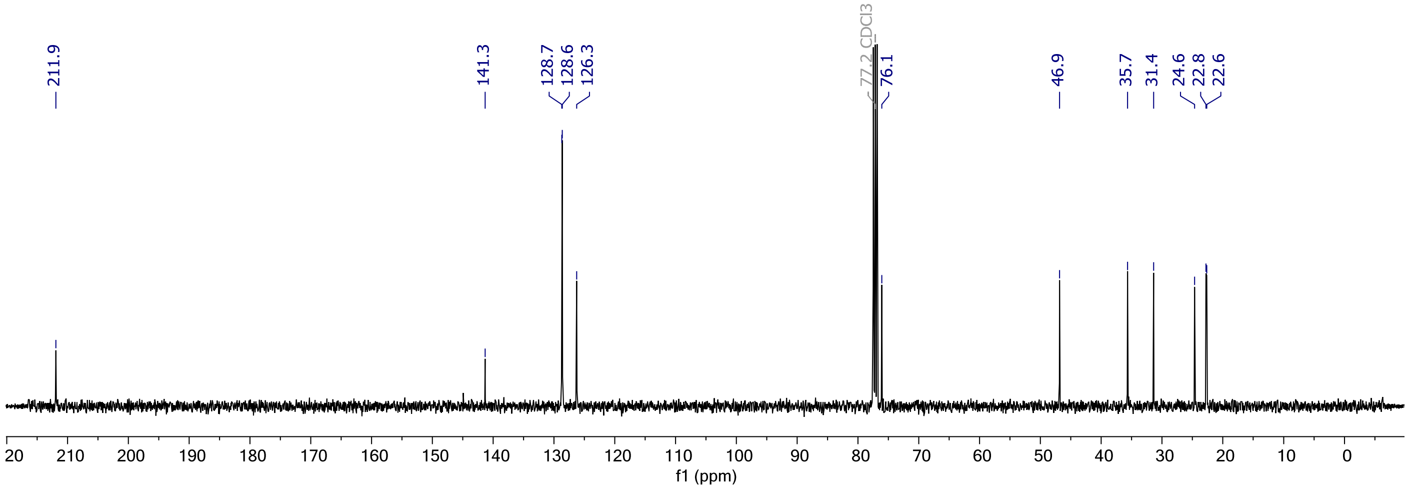

Figure S46. $^{13}C\{^{1}H\}$ NMR spectrum for **26** (101 MHz, $CDCl_3$, room temperature).

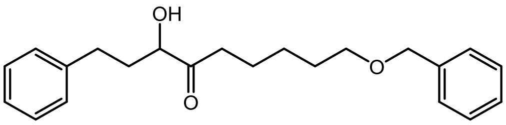

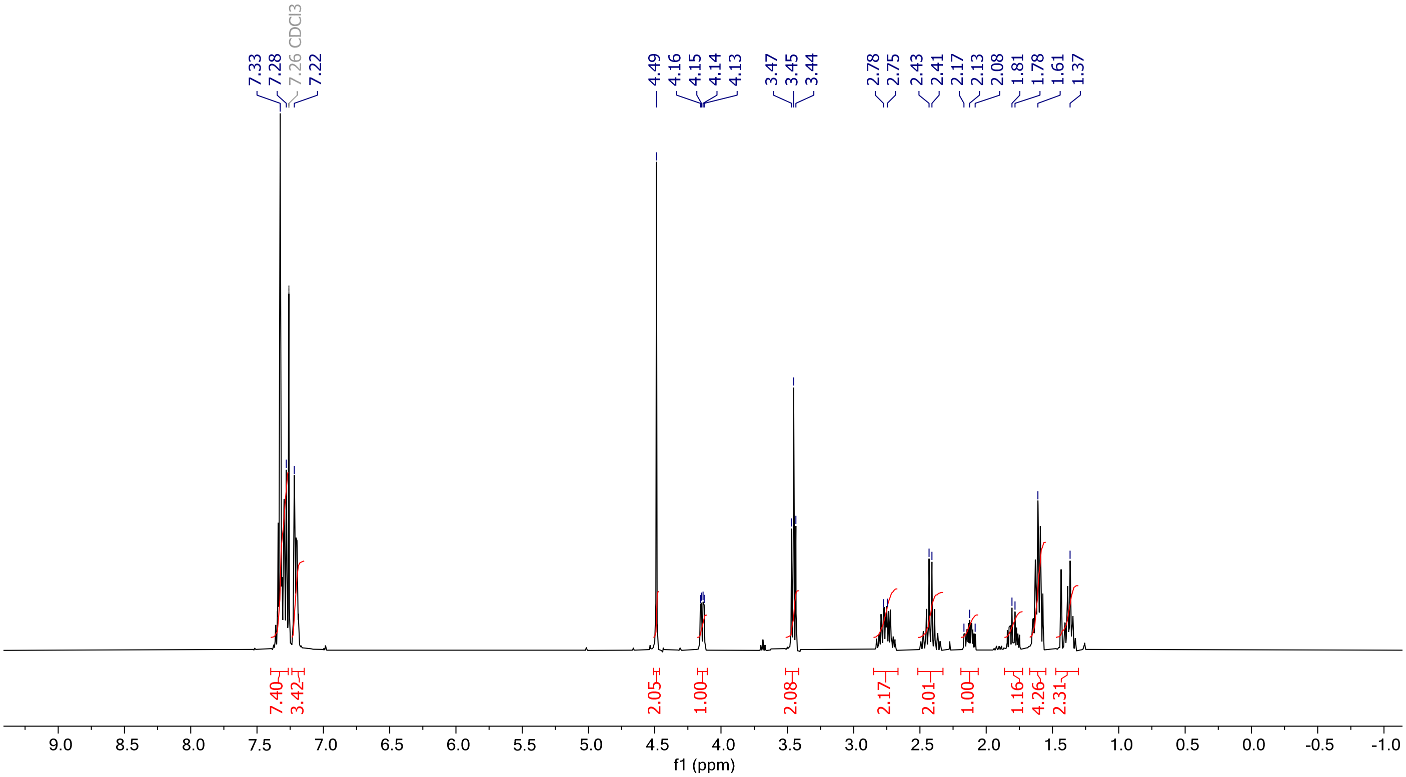

Figure S47. ^{1}H NMR spectrum for **27** (400 MHz, $CDCl_3$, room temperature).

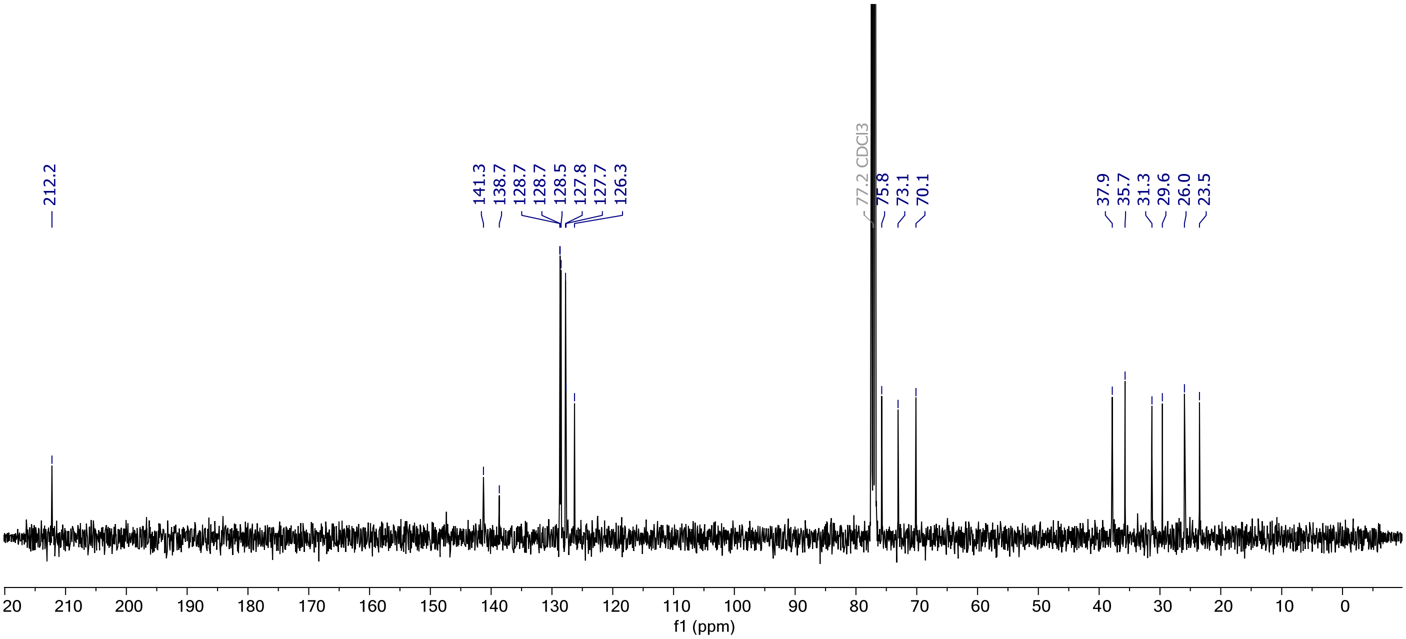

Figure S48. $^{13}C\{^{1}H\}$ NMR spectrum for **27** (101 MHz, $CDCl_3$, room temperature).

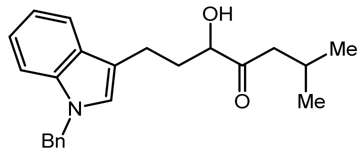

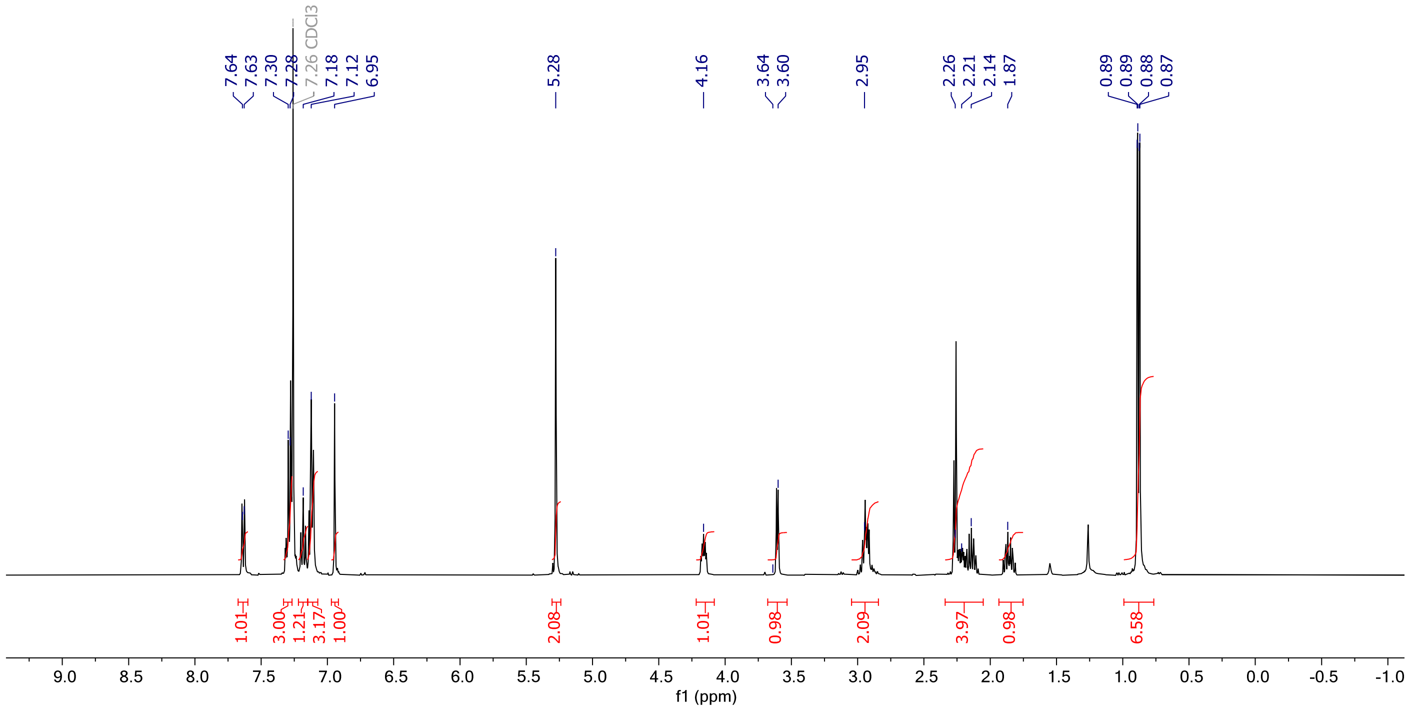

Figure S49. ^{1}H NMR spectrum for **28** (400 MHz, $CDCl_3$, room temperature).

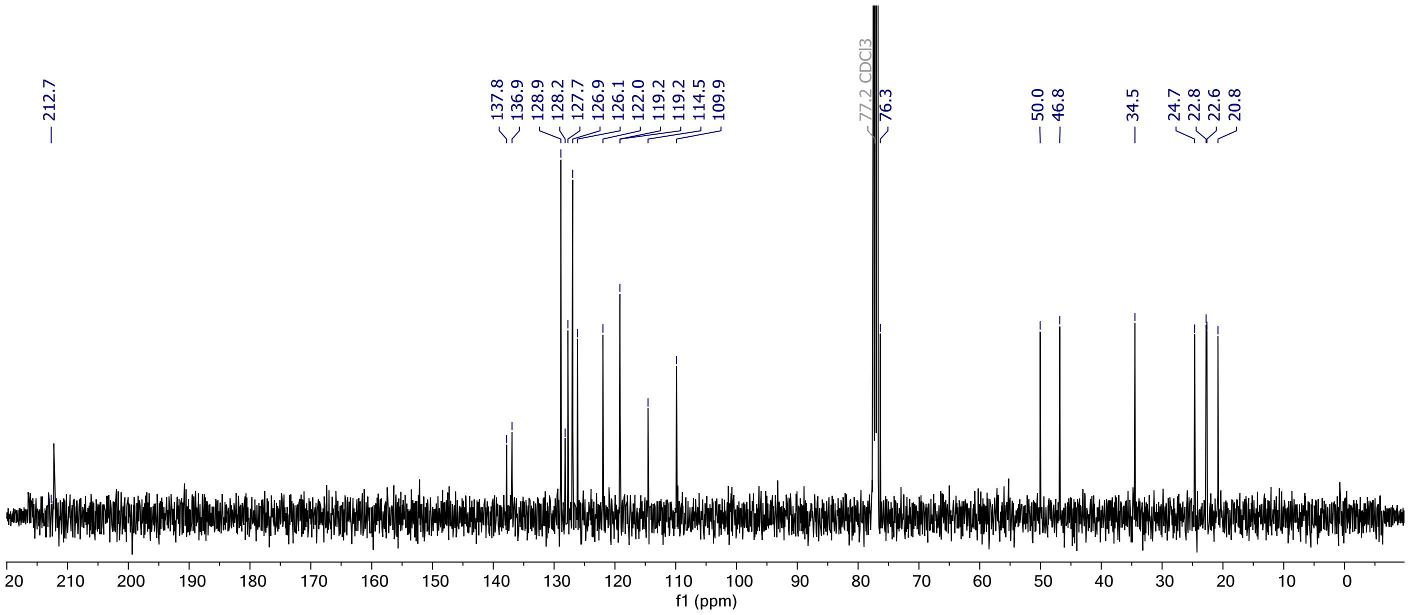

Figure S50. $^{13}C\{^{1}H\}$ NMR spectrum for **28** (101 MHz, $CDCl_3$, room temperature).

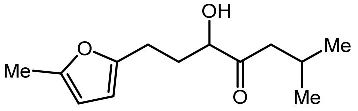

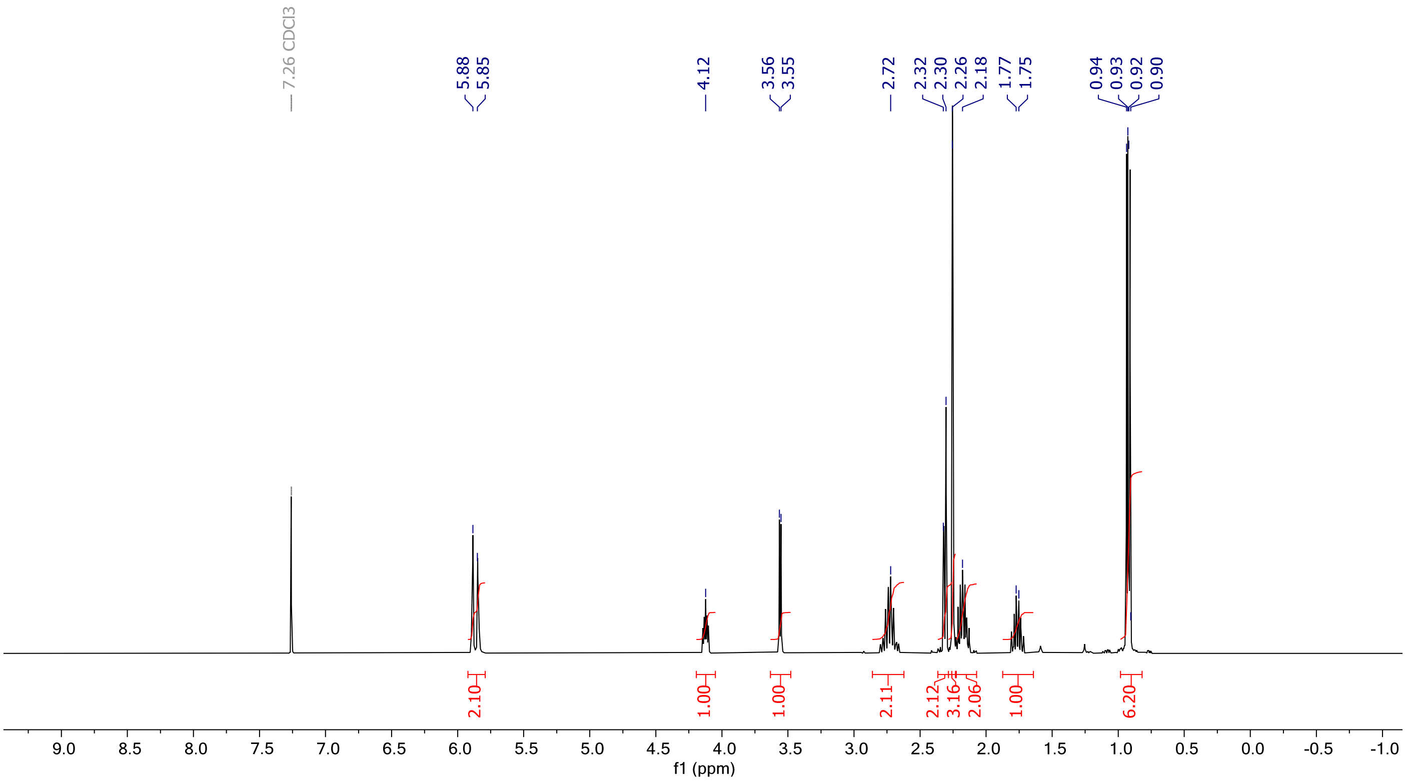

Figure S51. ^{1}H NMR spectrum for **29** (400 MHz, $CDCl_3$, room temperature).

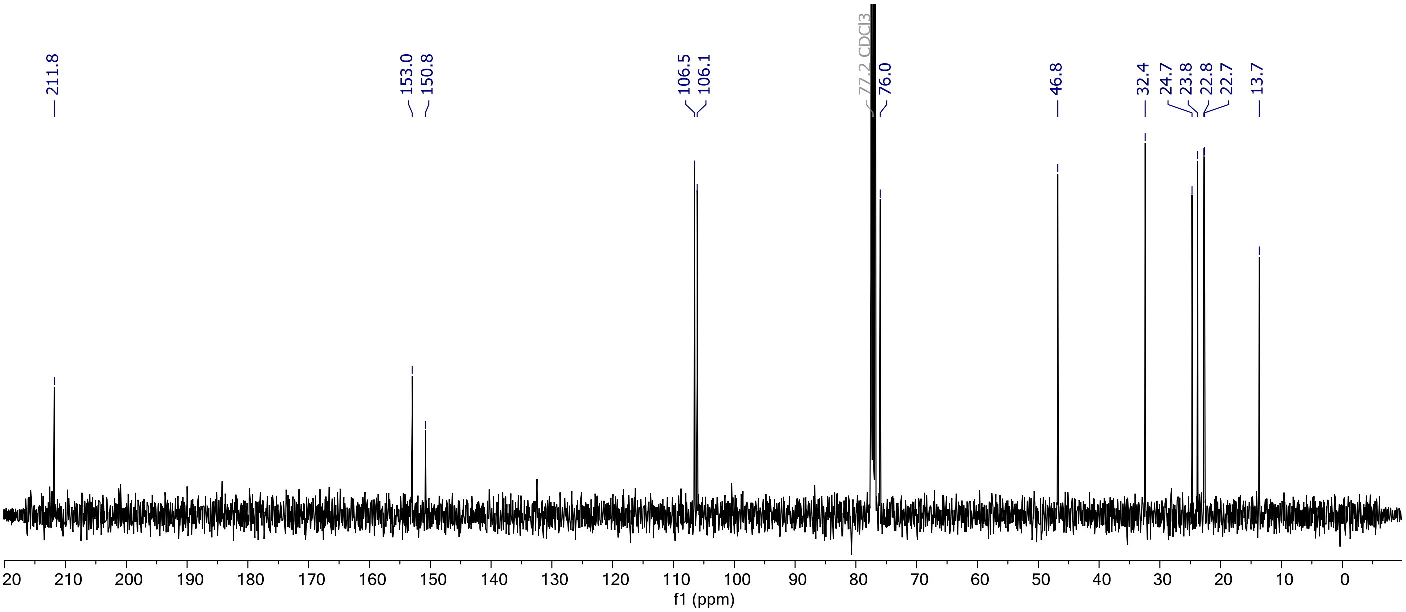

Figure S52. $^{13}C\{^{1}H\}$ NMR spectrum for **29** (101 MHz, $CDCl_3$, room temperature).

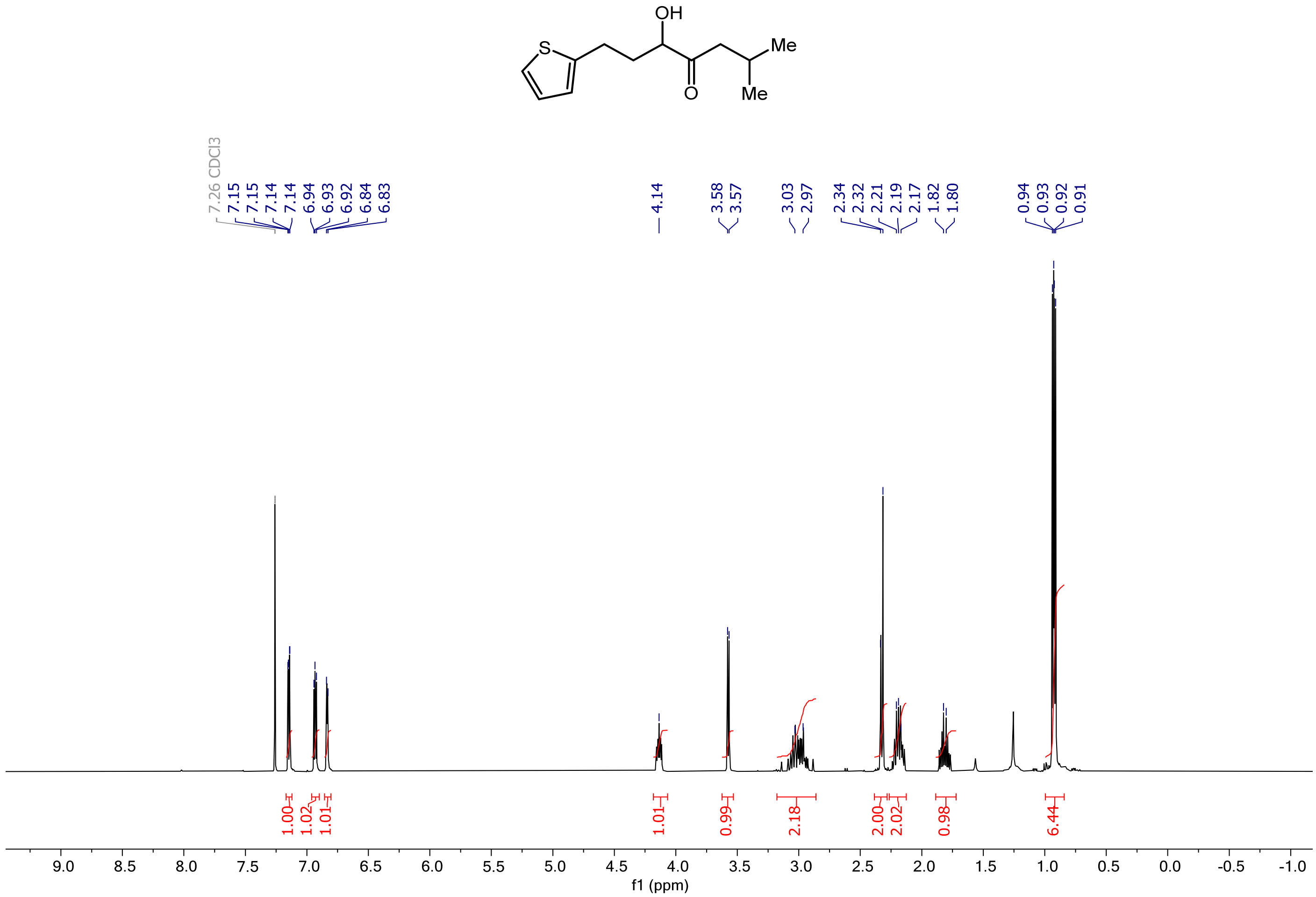

Figure S53. ^{1}H NMR spectrum for **30** (400 MHz, $CDCl_3$, room temperature).

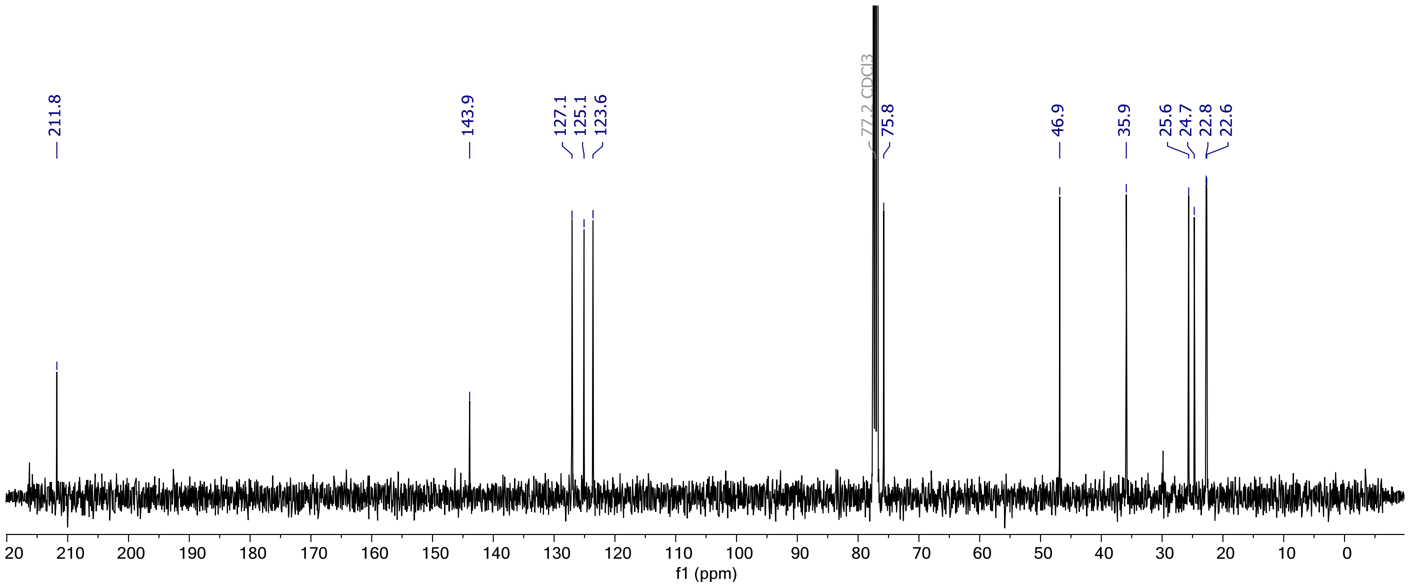

Figure S54. $^{13}C\{^{1}H\}$ NMR spectrum for **30** (101 MHz, $CDCl_3$, room temperature).

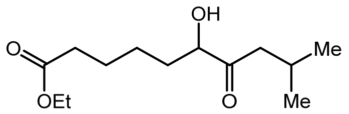

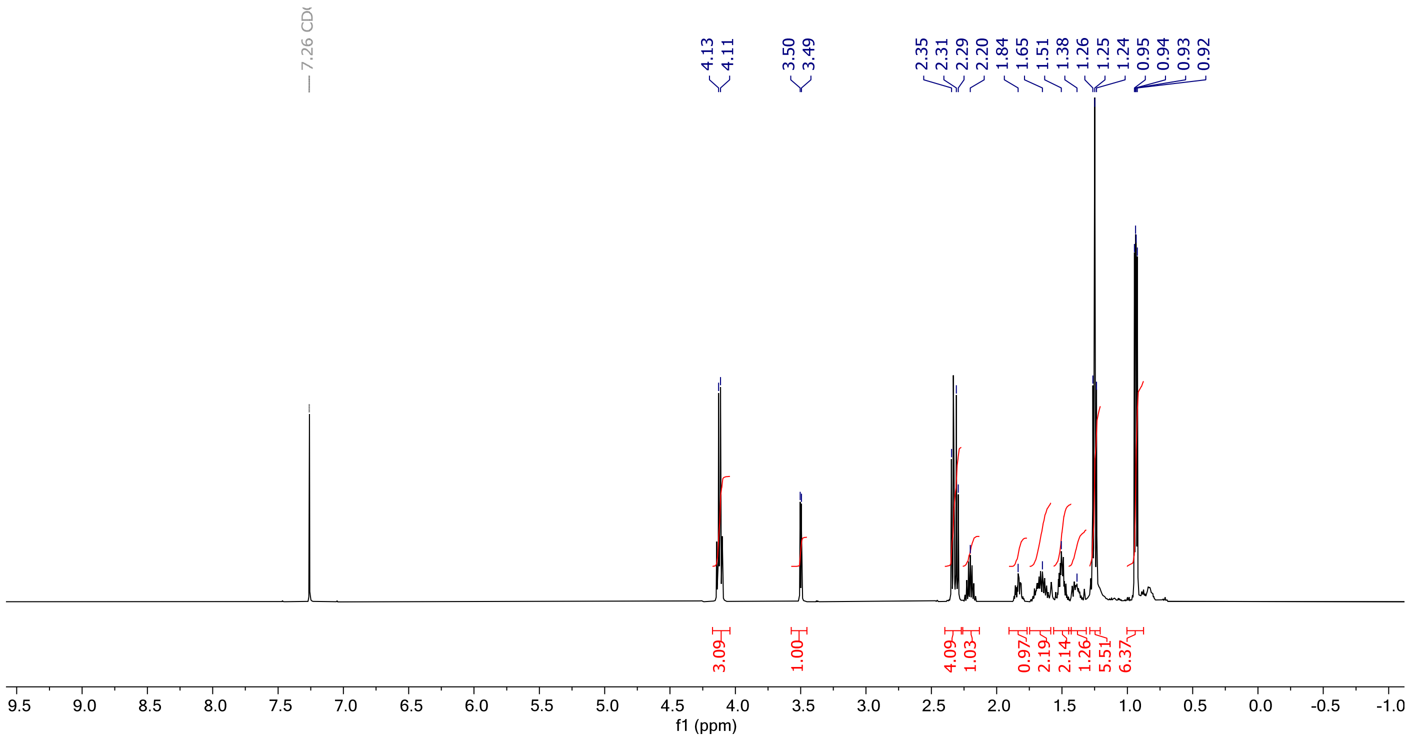

Figure S55. ^{1}H NMR spectrum for **31** (500 MHz, $CDCl_3$, room temperature).

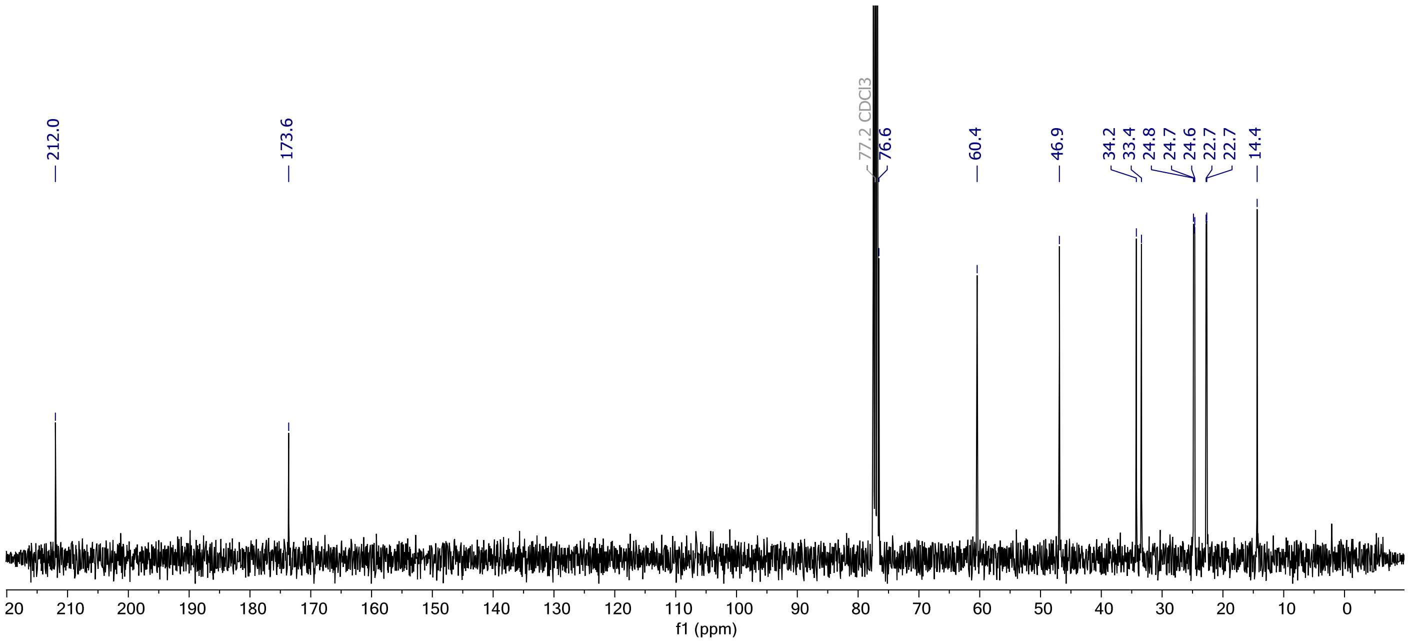

Figure S56. $^{13}C\{^{1}H\}$ NMR spectrum for **31** (101 MHz, $CDCl_3$, room temperature).

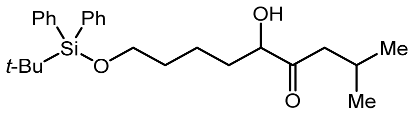

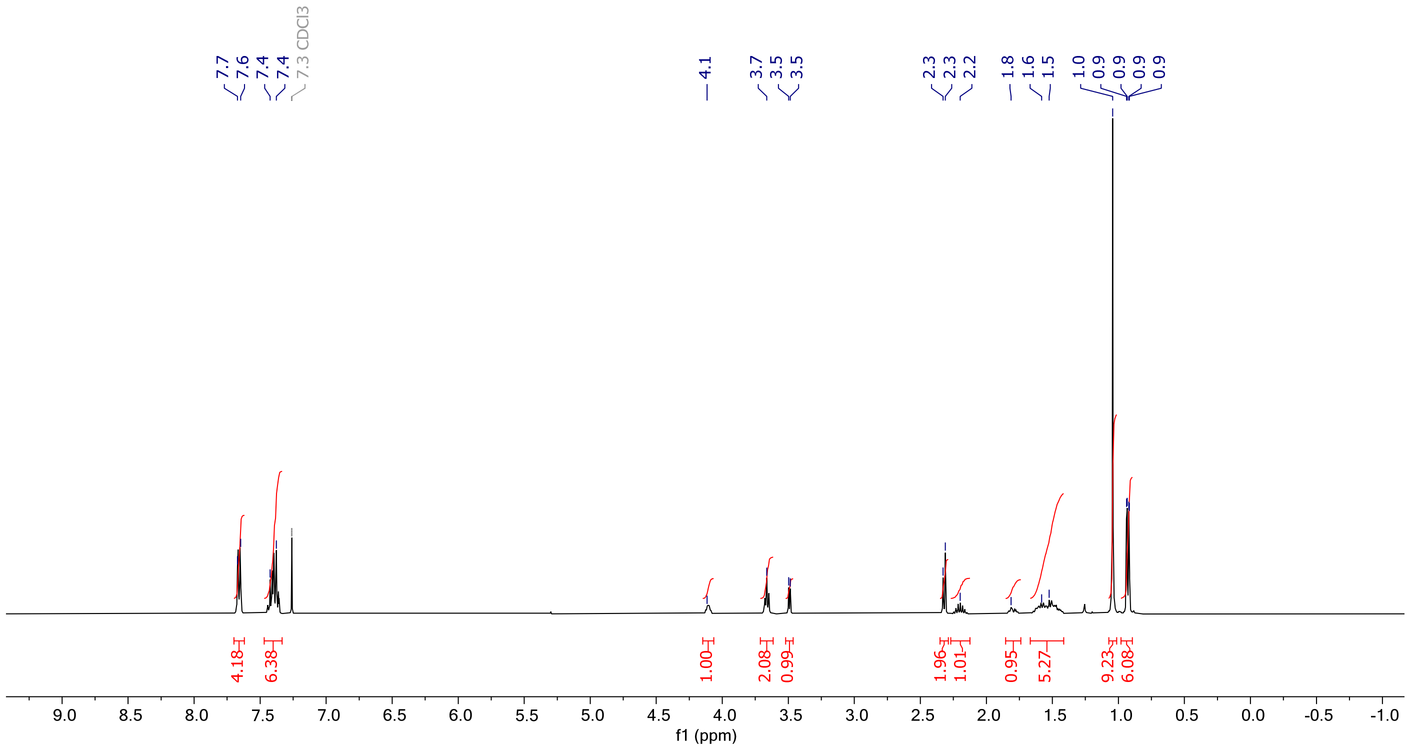

Figure S57. ^{1}H NMR spectrum for **32** (400 MHz, $CDCl_3$, room temperature).

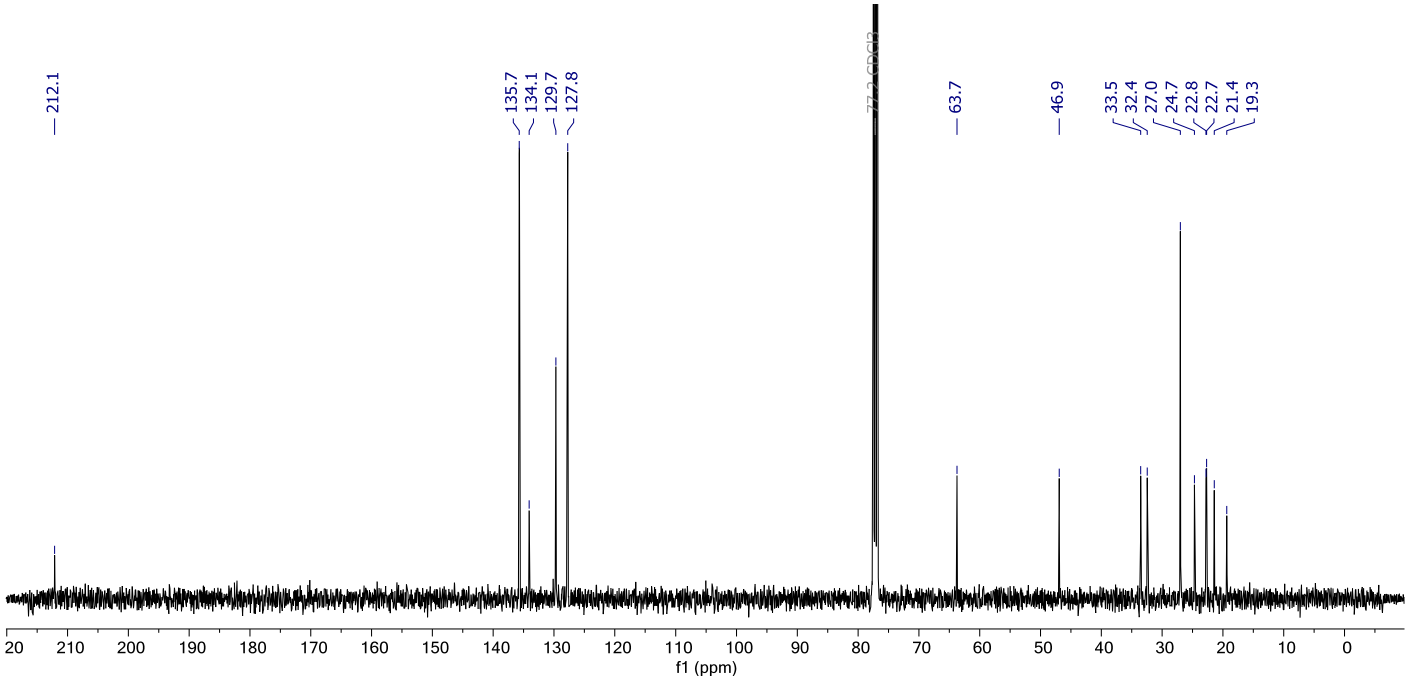

Figure S58. ^{13}C{^{1}H} NMR spectrum for **32** (101 MHz, $CDCl_3$, room temperature).

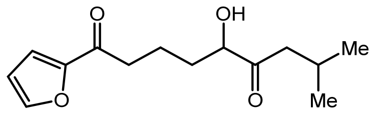

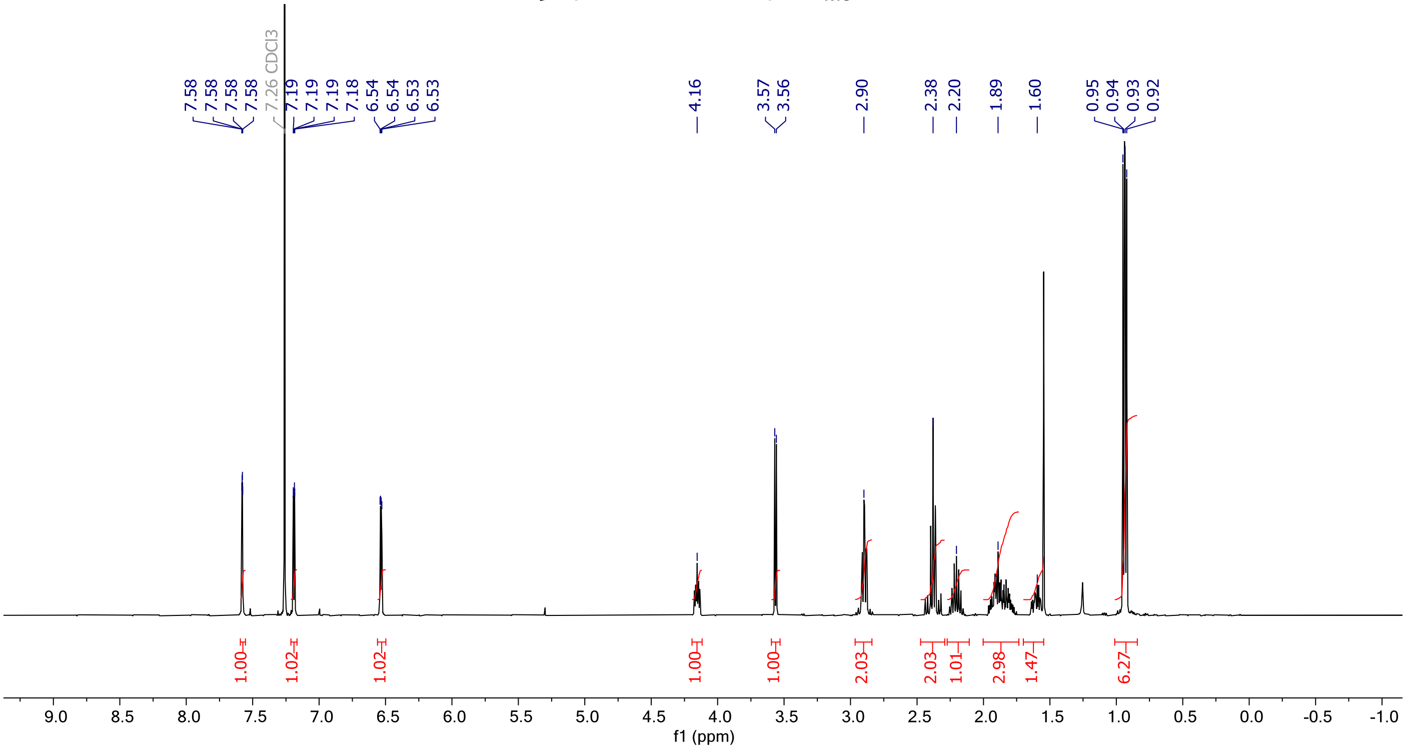

Figure S59. ^{1}H NMR spectrum for **33** (400 MHz, $CDCl_3$, room temperature).

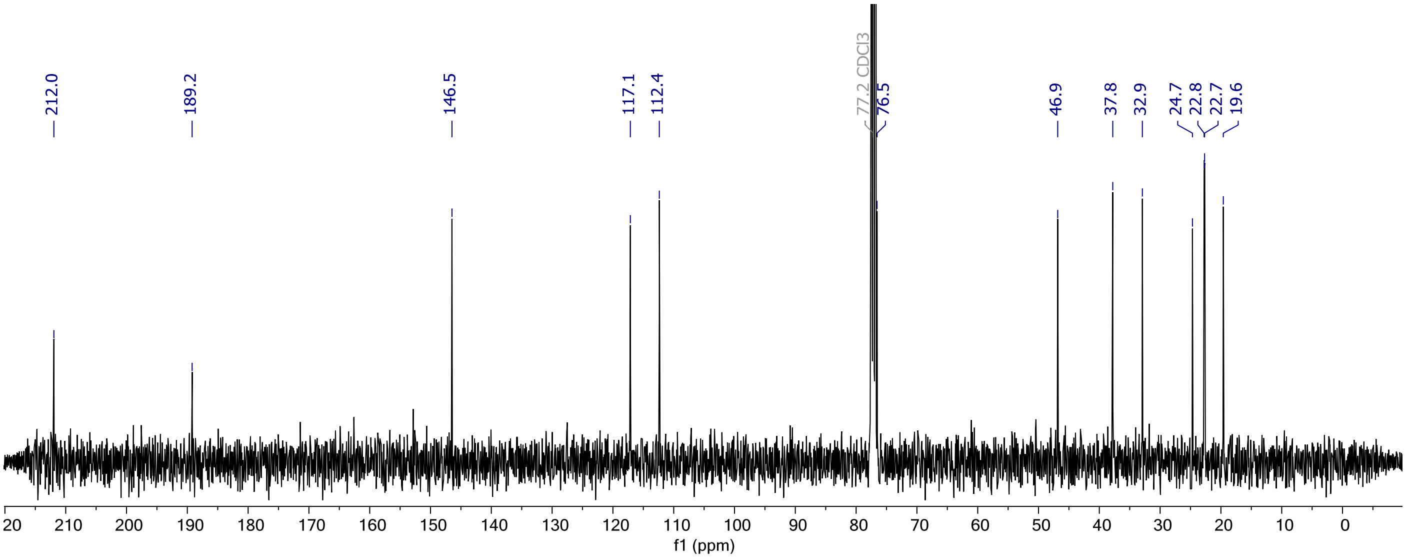

Figure S60. $^{13}C\{^{1}H\}$ NMR spectrum for **33** (101 MHz, $CDCl_3$, room temperature).

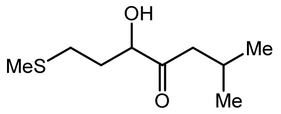

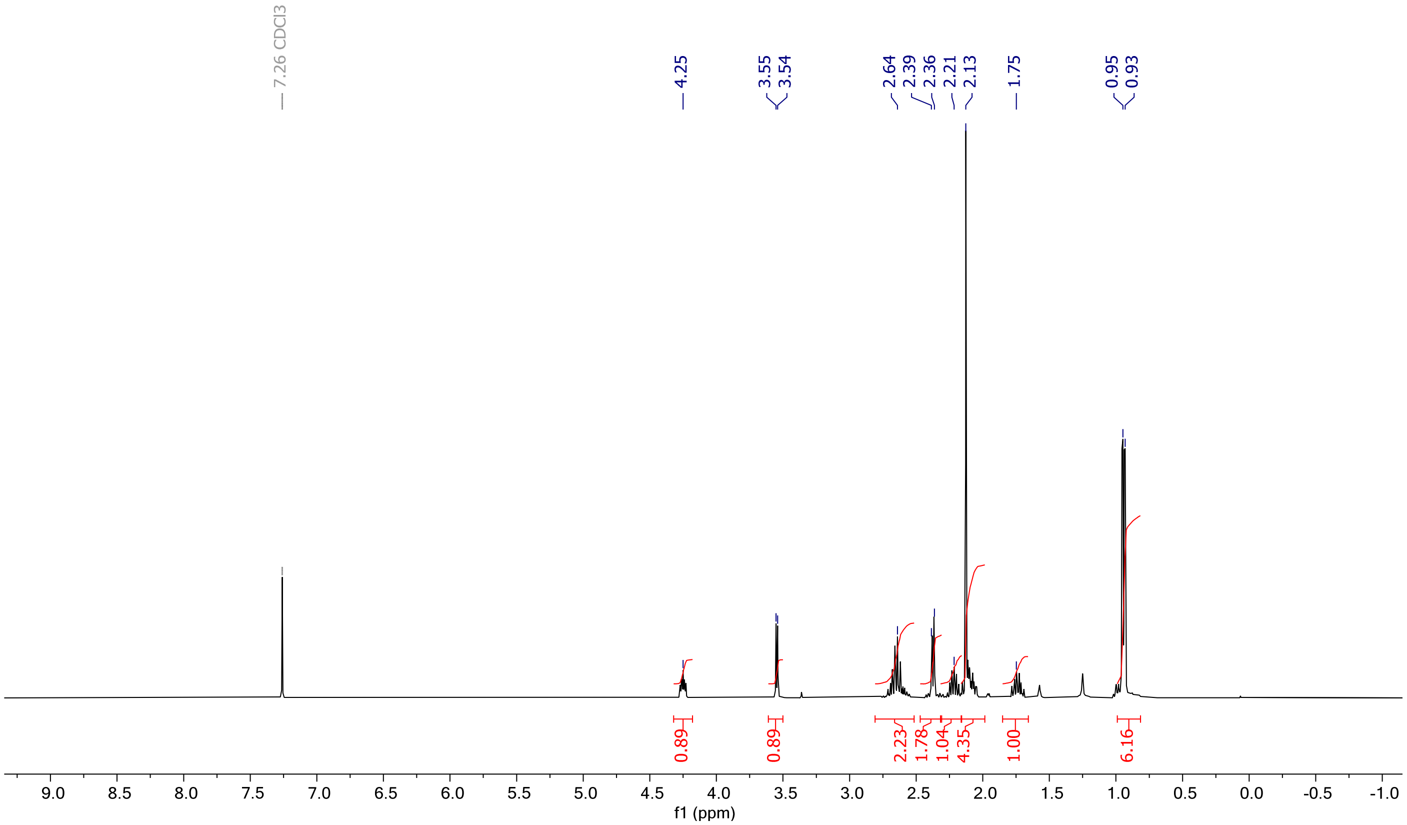

Figure S61. ^{1}H NMR spectrum for **34** (400 MHz, $CDCl_3$, room temperature).

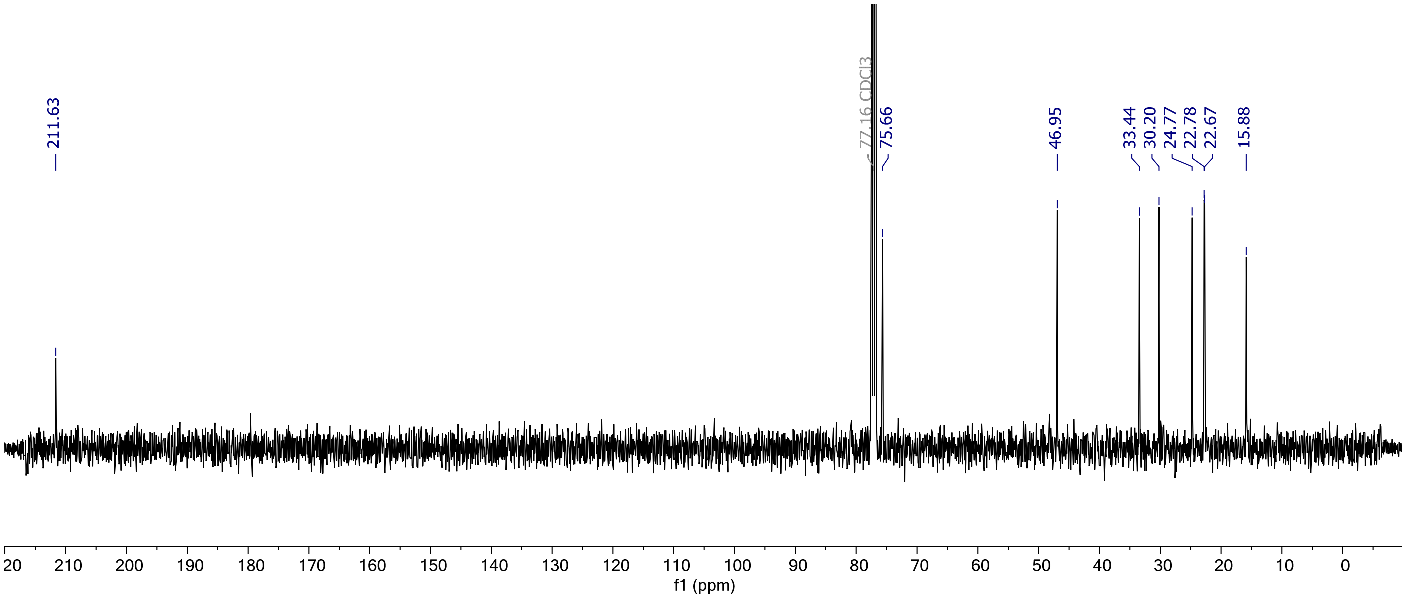

Figure S62. $^{13}C\{^{1}H\}$ NMR spectrum for **34** (101 MHz, $CDCl_3$, room temperature).

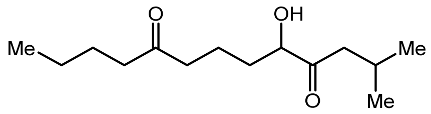

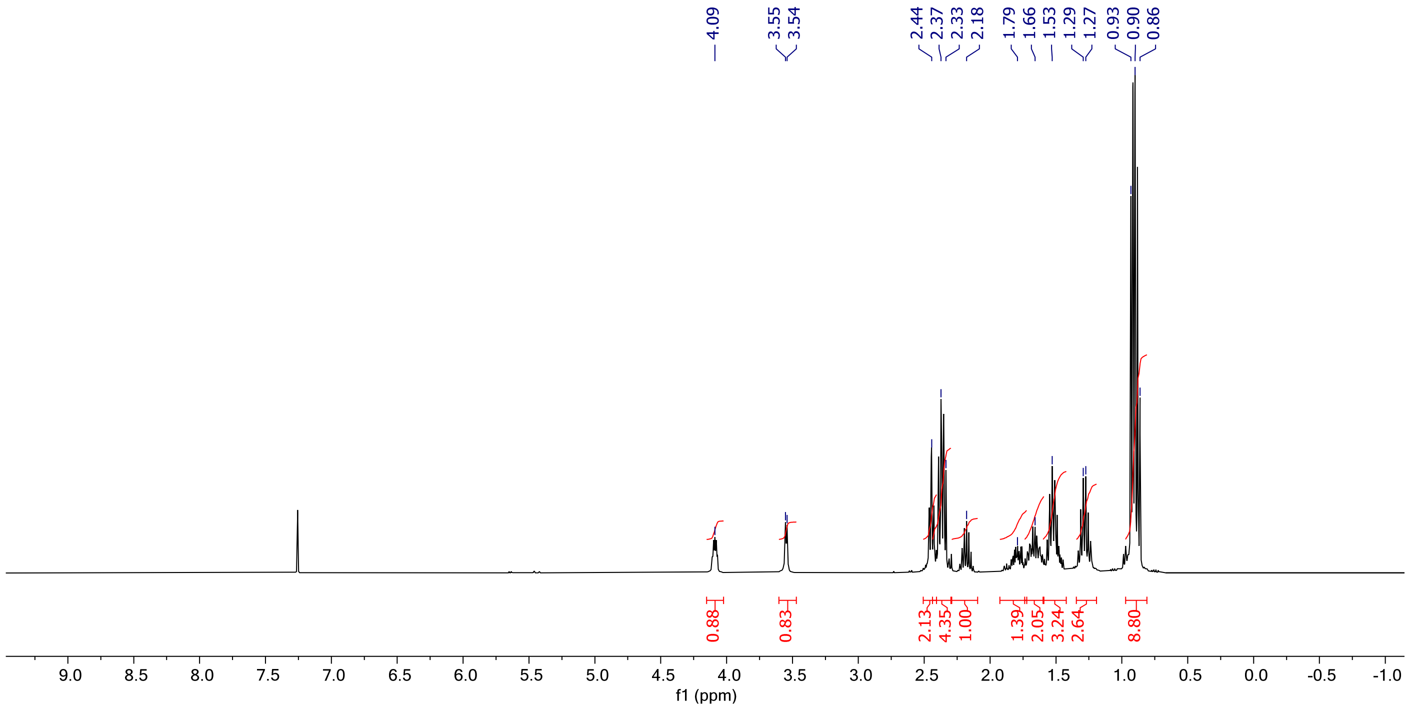

Figure 63. ^{1}H NMR spectrum for **35** (400 MHz, $CDCl_3$, room temperature).

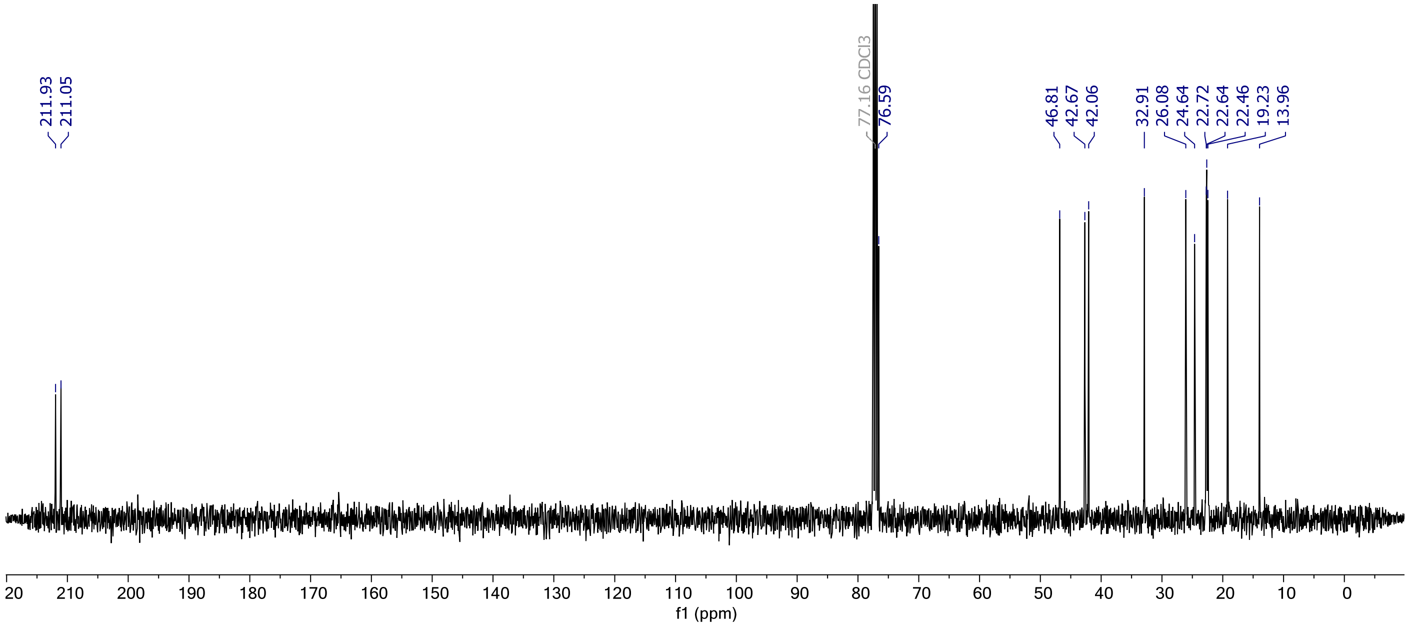

Figure S64. ^{13}C{^{1}H} NMR spectrum for **35** (101 MHz, $CDCl_3$, room temperature).

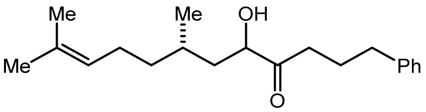

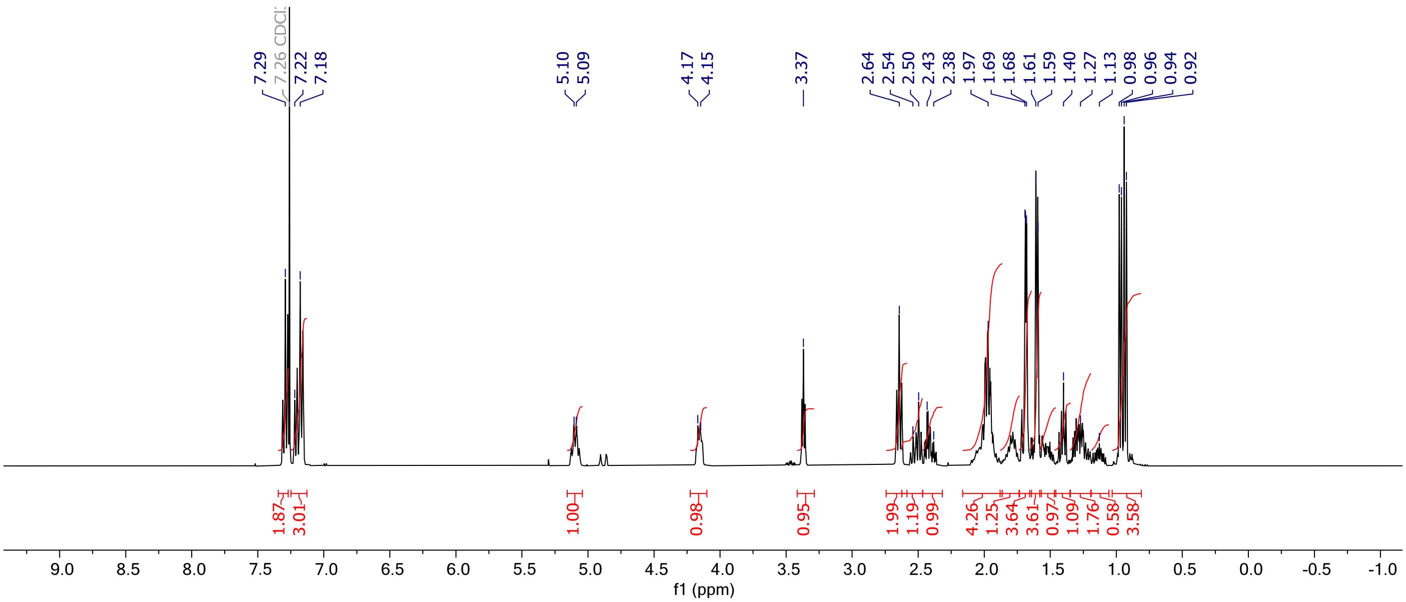

Figure S65. ^{1}H NMR spectrum for **36** (400 MHz, $CDCl_3$, room temperature).

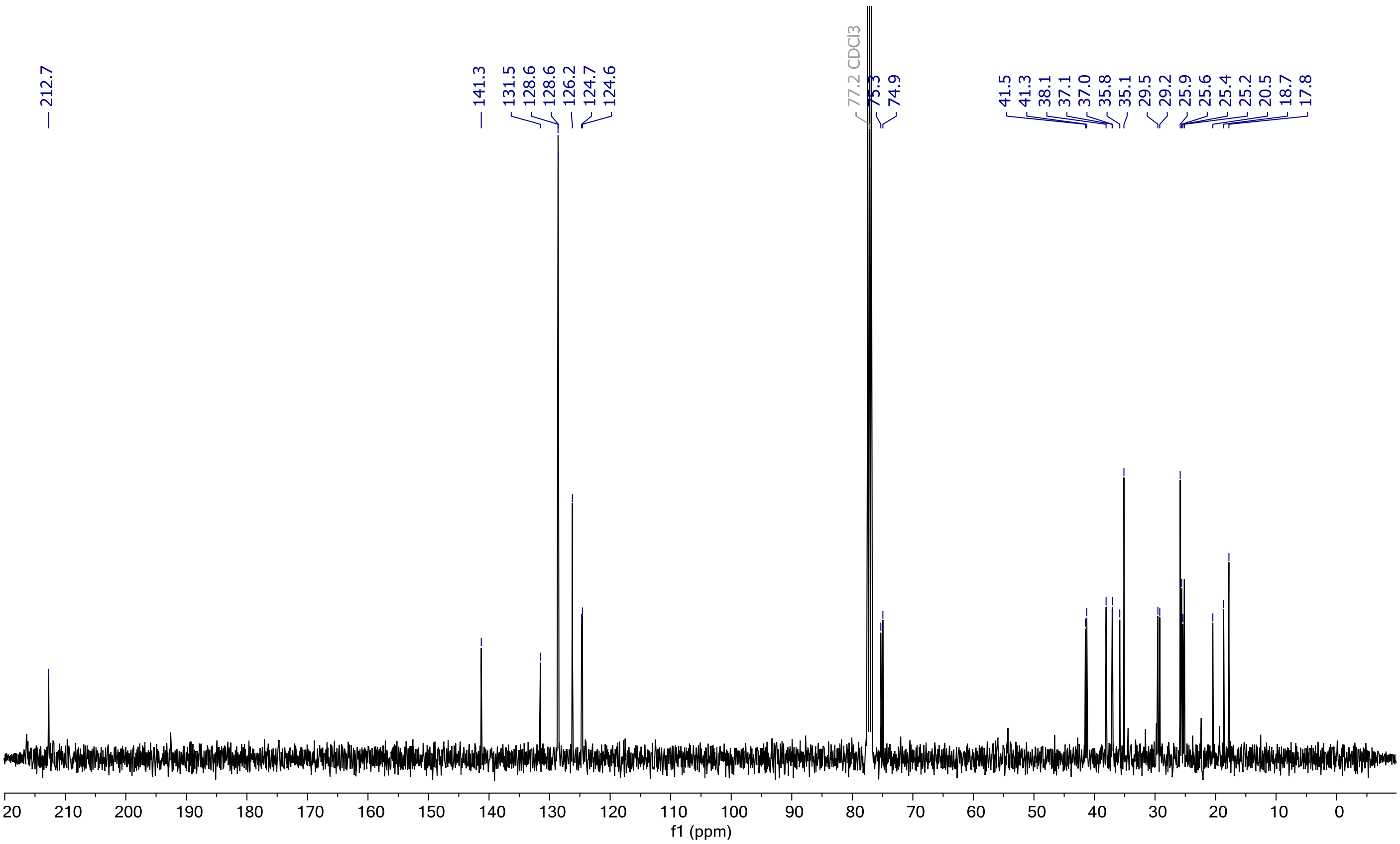

Figure S66. $^{13}C\{^{1}H\}$ NMR spectrum for **36** (101 MHz, $CDCl_3$, room temperature).

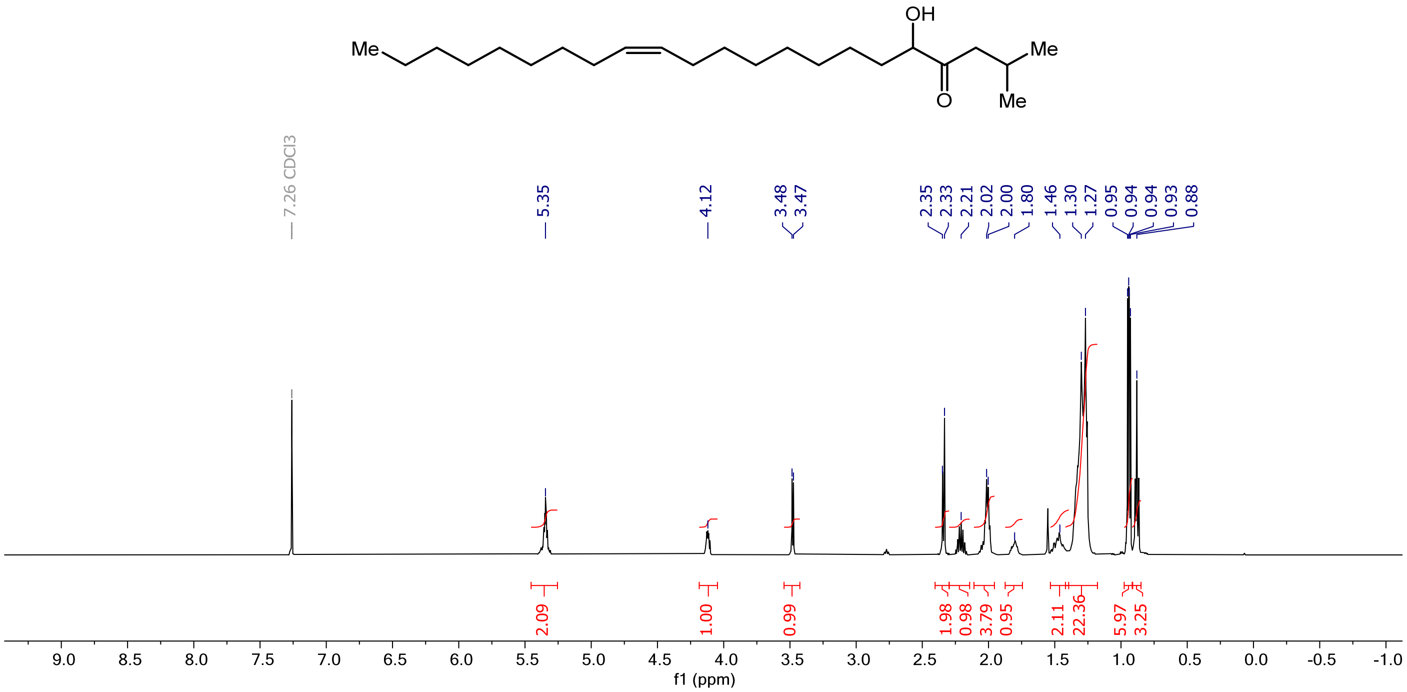

Figure S67. ^{1}H NMR spectrum for **37** (500 MHz, $CDCl_3$, room temperature).

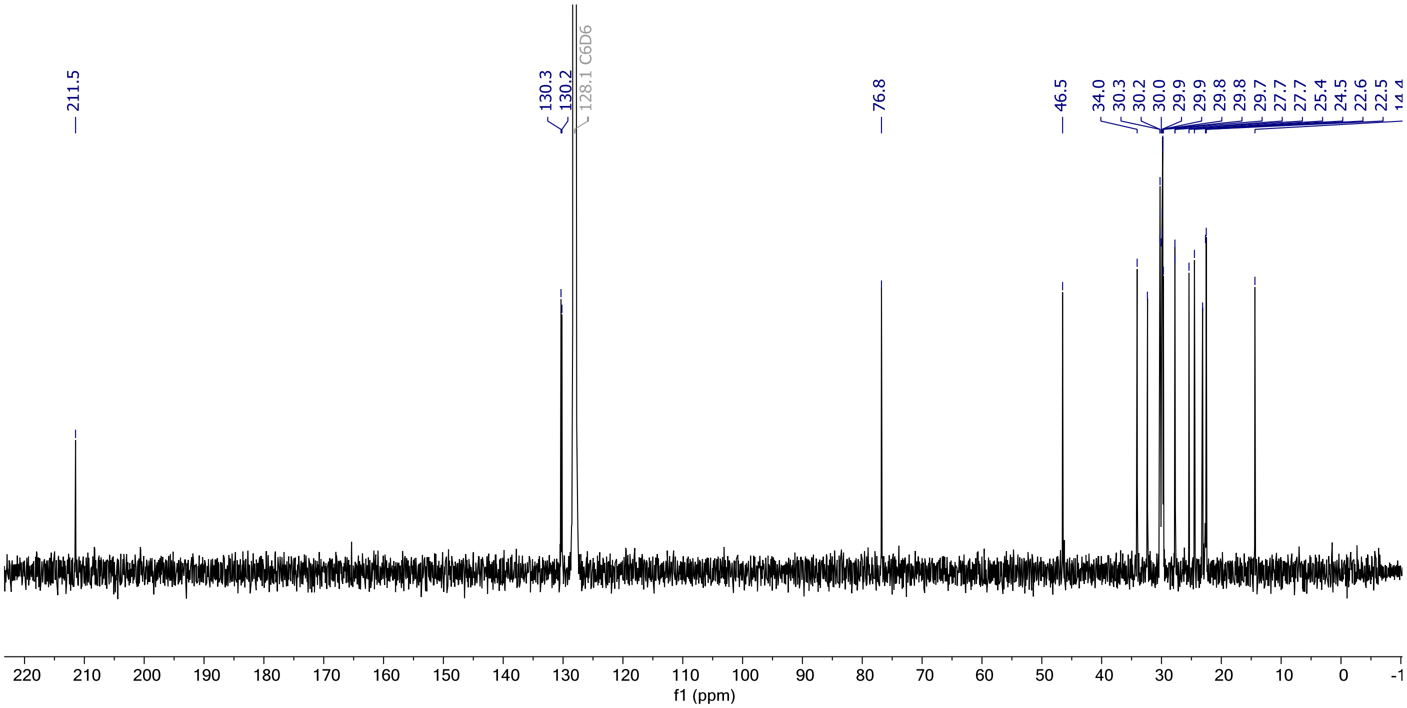

Figure S68. $^{13}C\{^{1}H\}$ NMR spectrum for **37** (126 MHz, C_6D_6, room temperature).

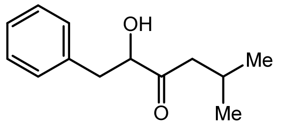

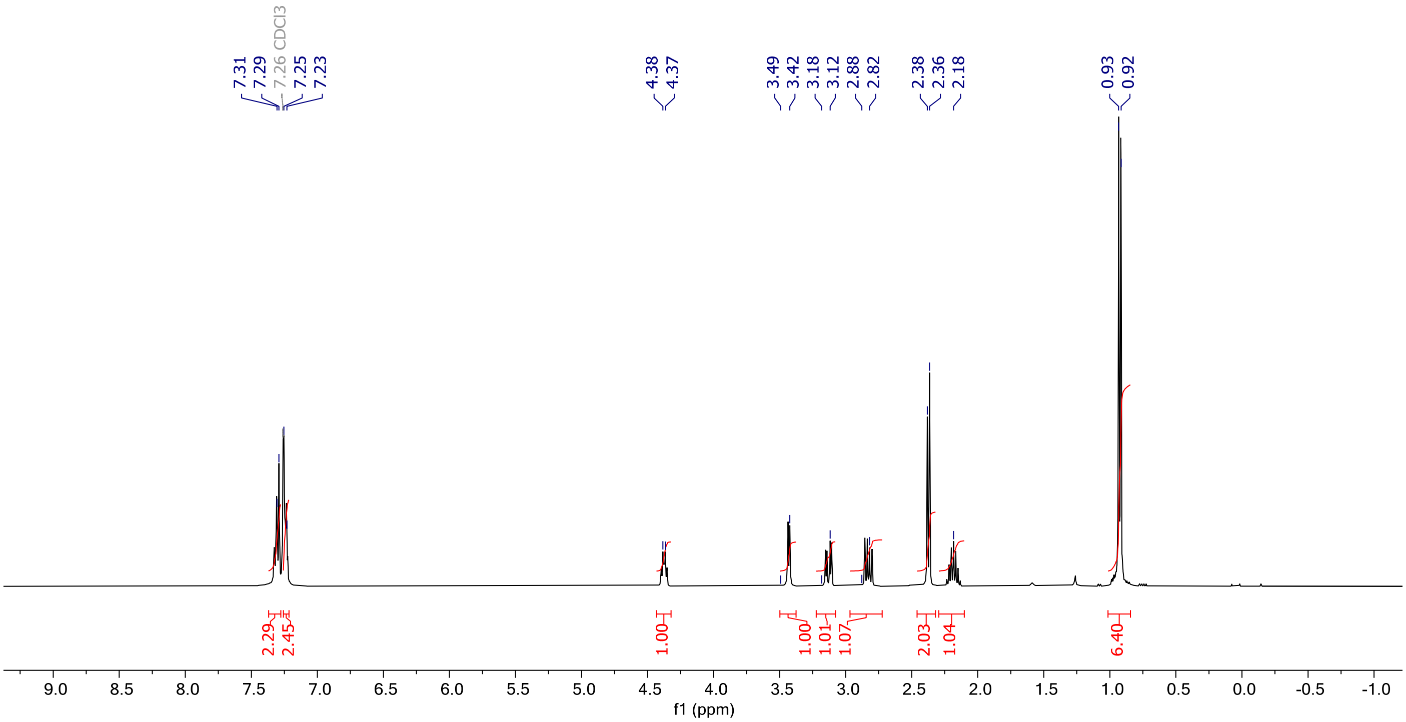

Figure S69. ^{1}H NMR spectrum for **38** (400 MHz, $CDCl_3$, room temperature).

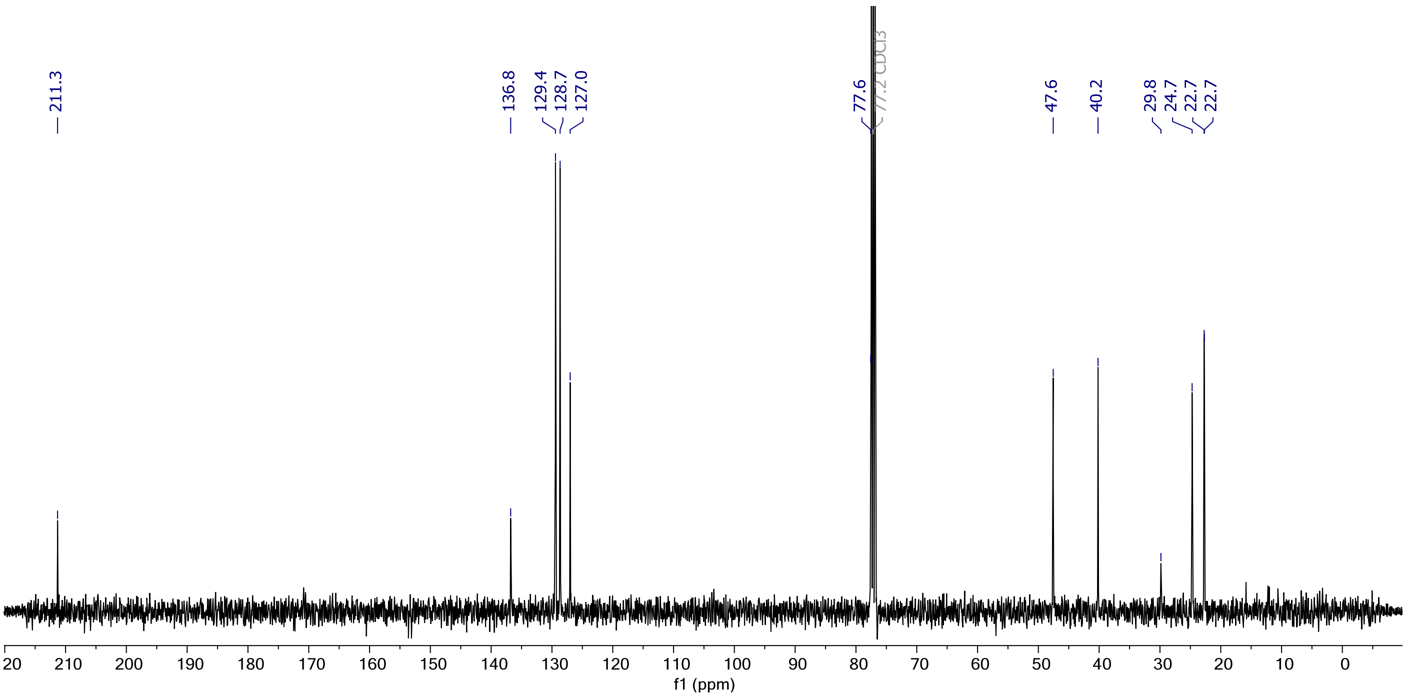

Figure S70. $^{13}C\{^{1}H\}$ NMR spectrum for **38** (101 MHz, $CDCl_3$, room temperature)

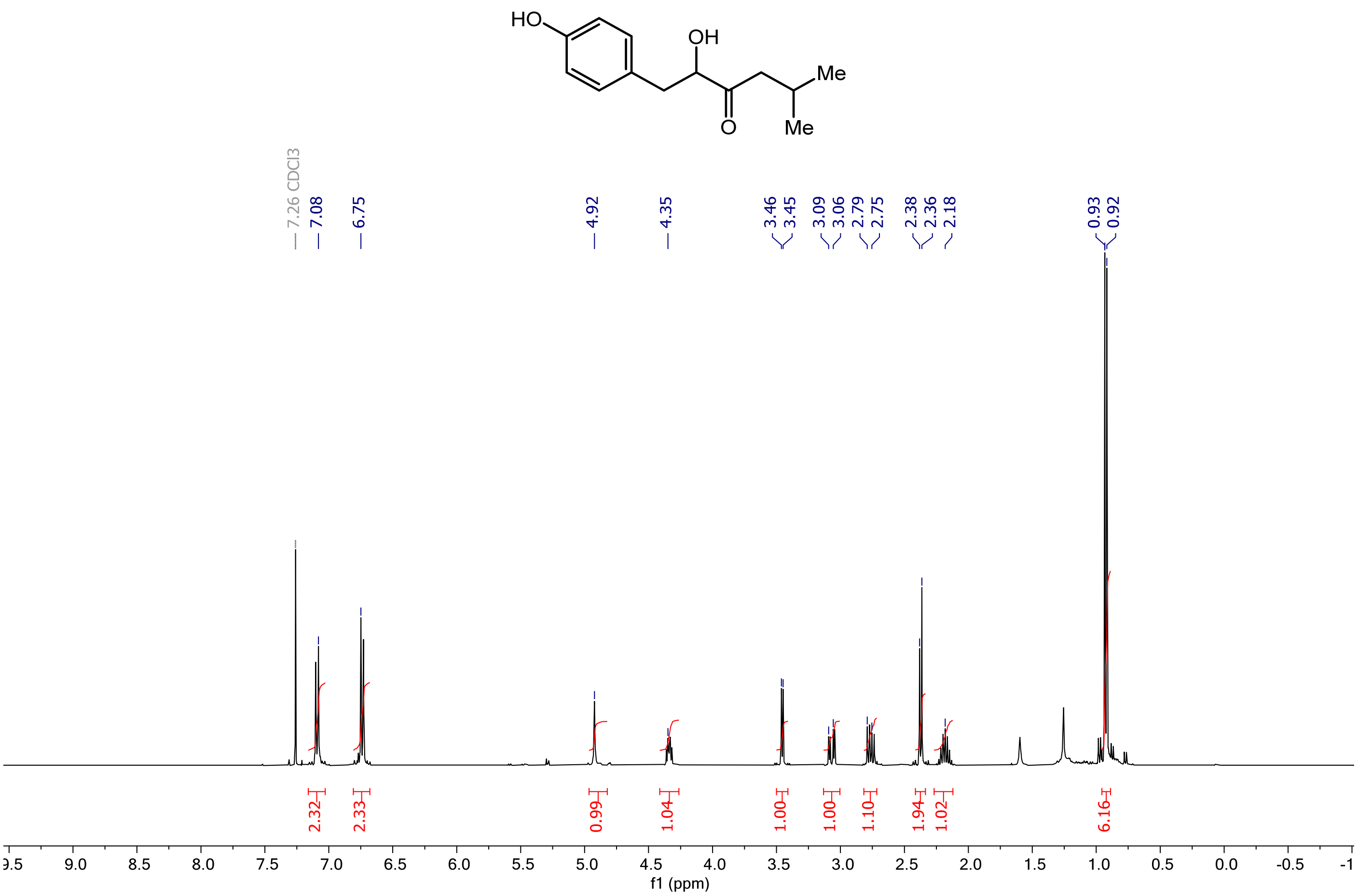

Figure S71. ^{1}H NMR spectrum for **39** (400 MHz, $CDCl_3$, room temperature).

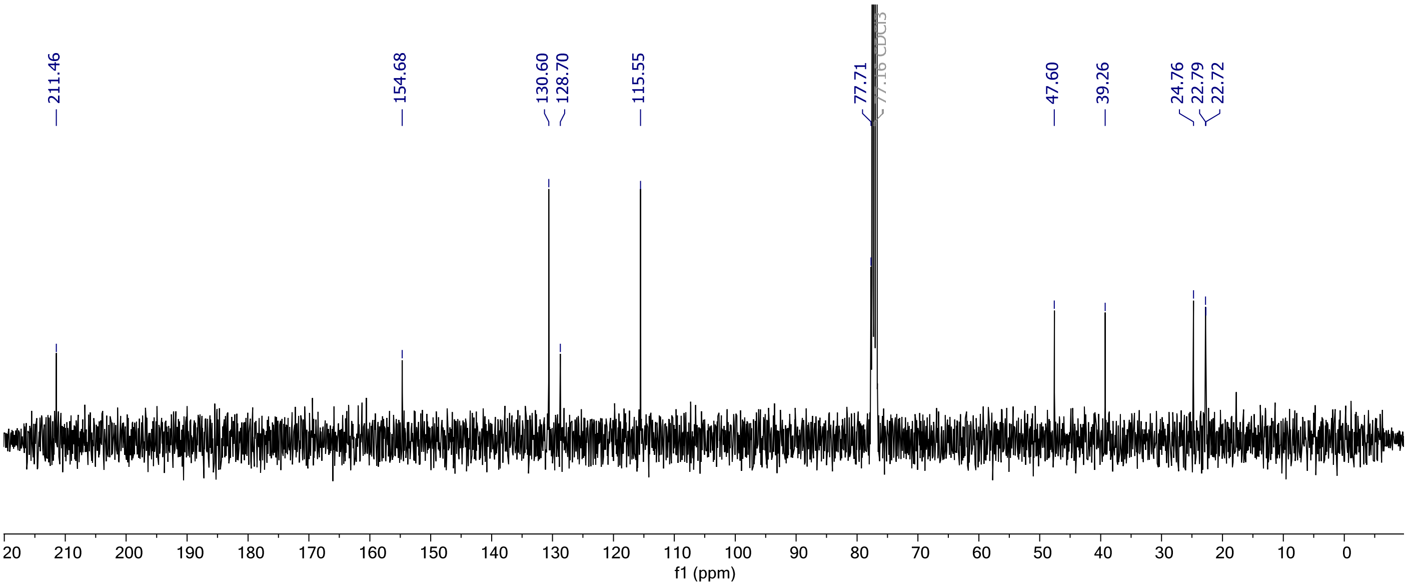

Figure S72. $^{13}C\{^{1}H\}$ NMR spectrum for **39** (101 MHz, $CDCl_3$, room temperature)

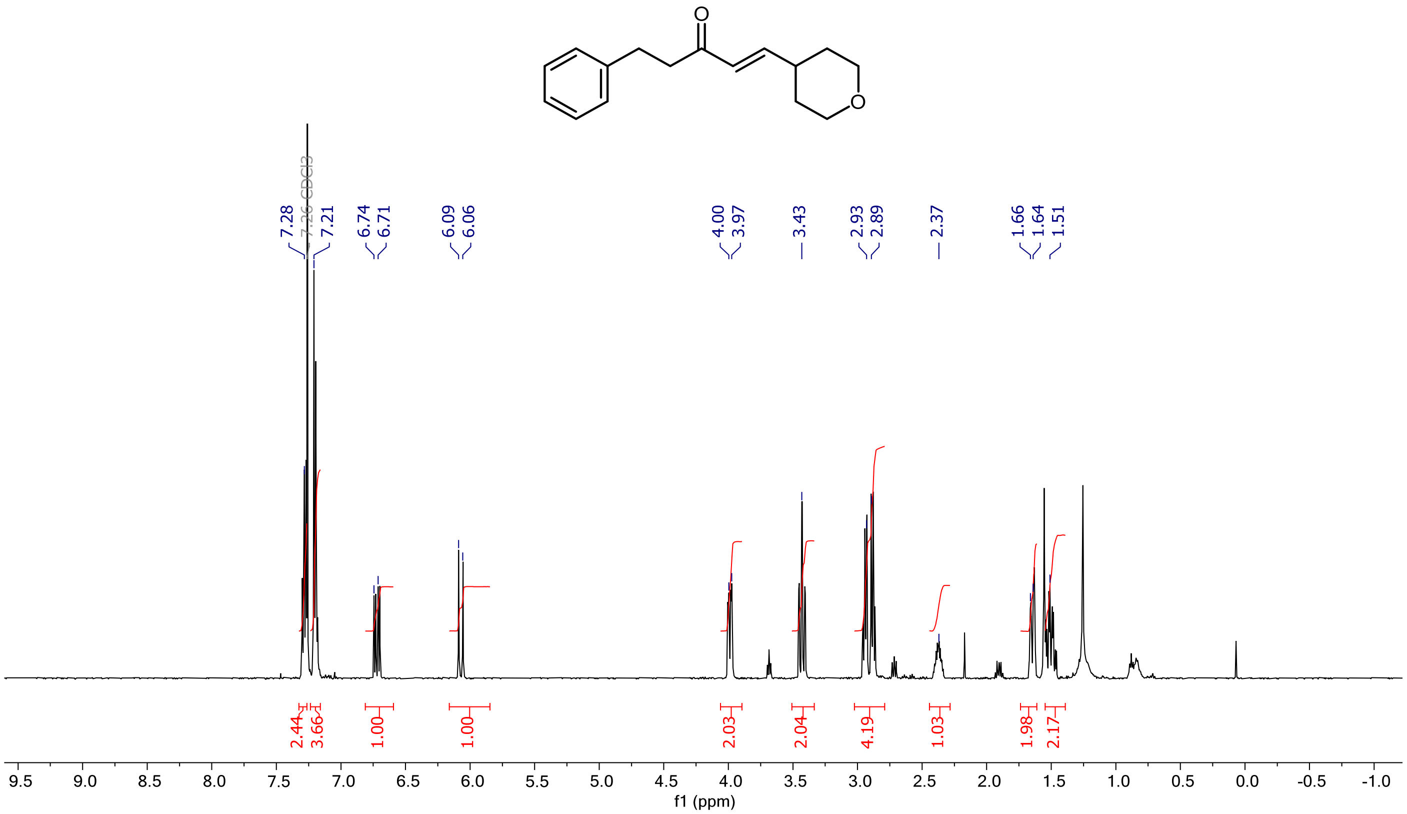

Figure S73. ^{1}H NMR spectrum for **42** (500 MHz, $CDCl_3$, room temperature).

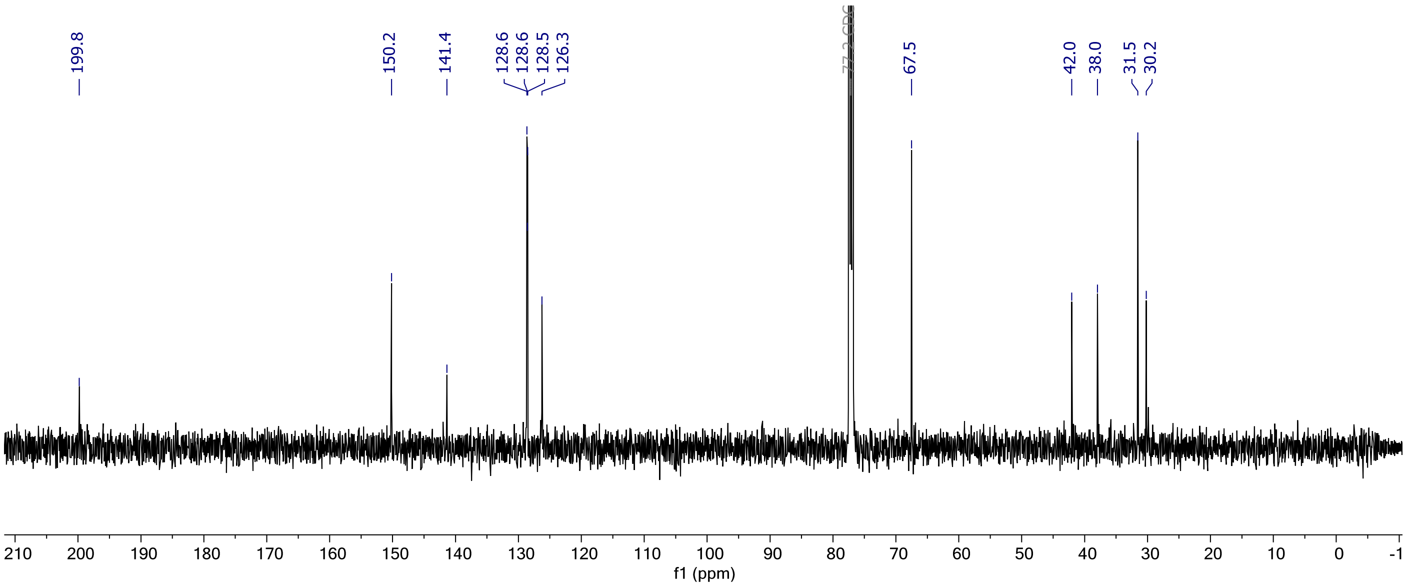

Figure S74. $^{13}C\{^{1}H\}$ NMR spectrum for **42** (126 MHz, $CDCl_3$, room temperature).

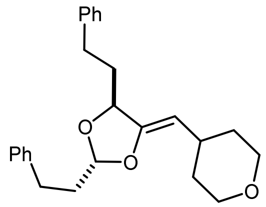

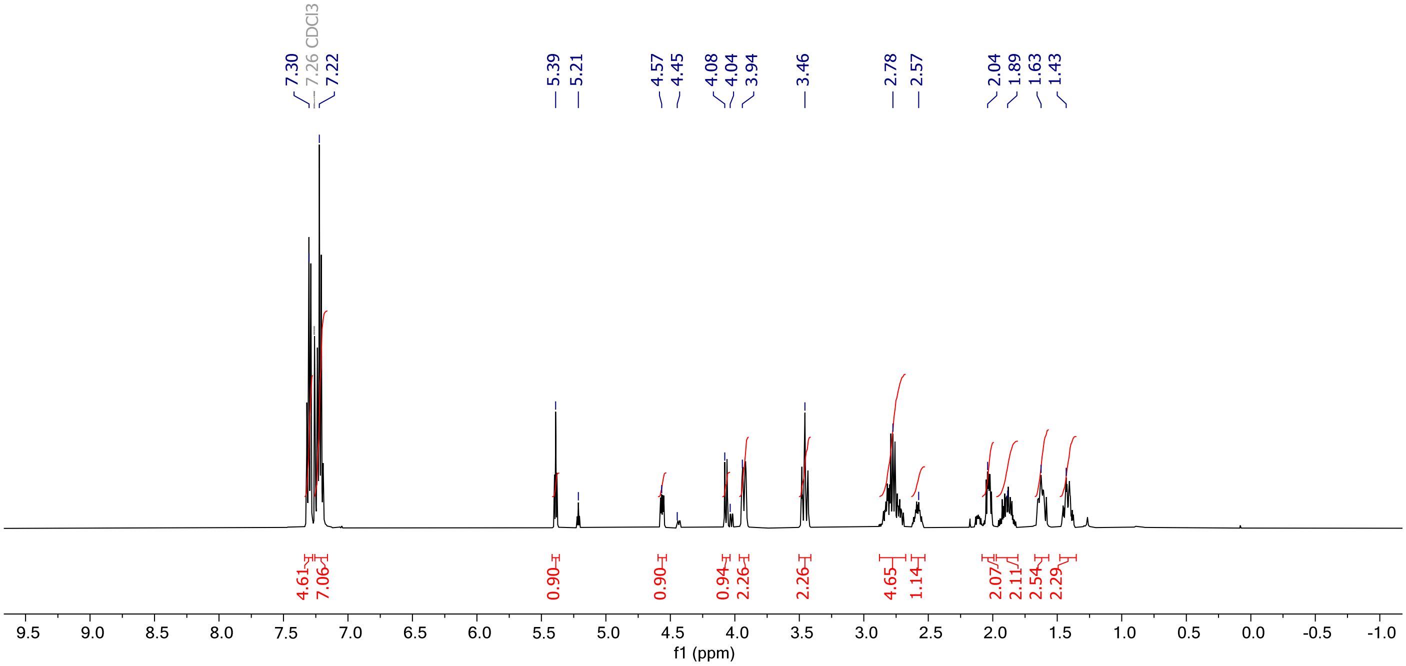

Figure S75. ^{1}H NMR spectrum for **43** (500 MHz, $CDCl_3$, room temperature).

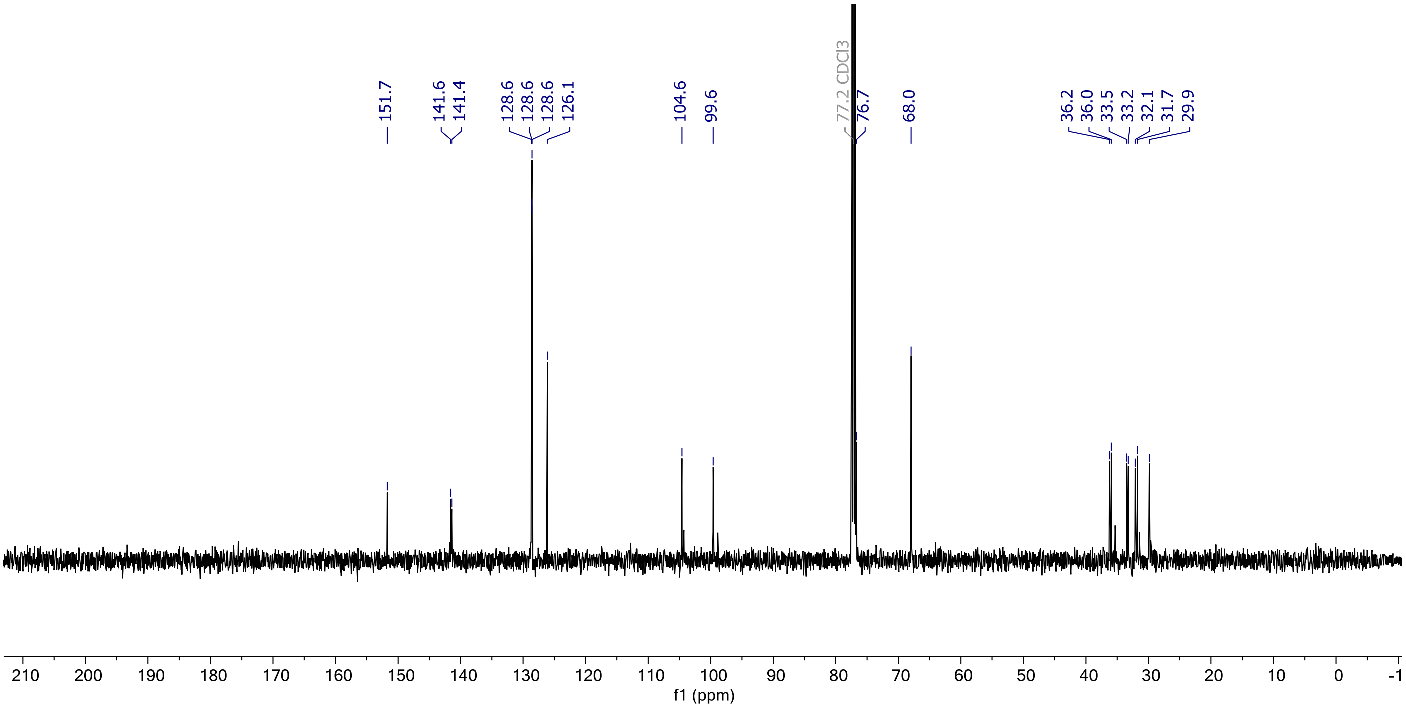

Figure S76. ^{13}C {^{1}H} NMR spectrum for **43** (126 MHz, $CDCl_3$, room temperature)

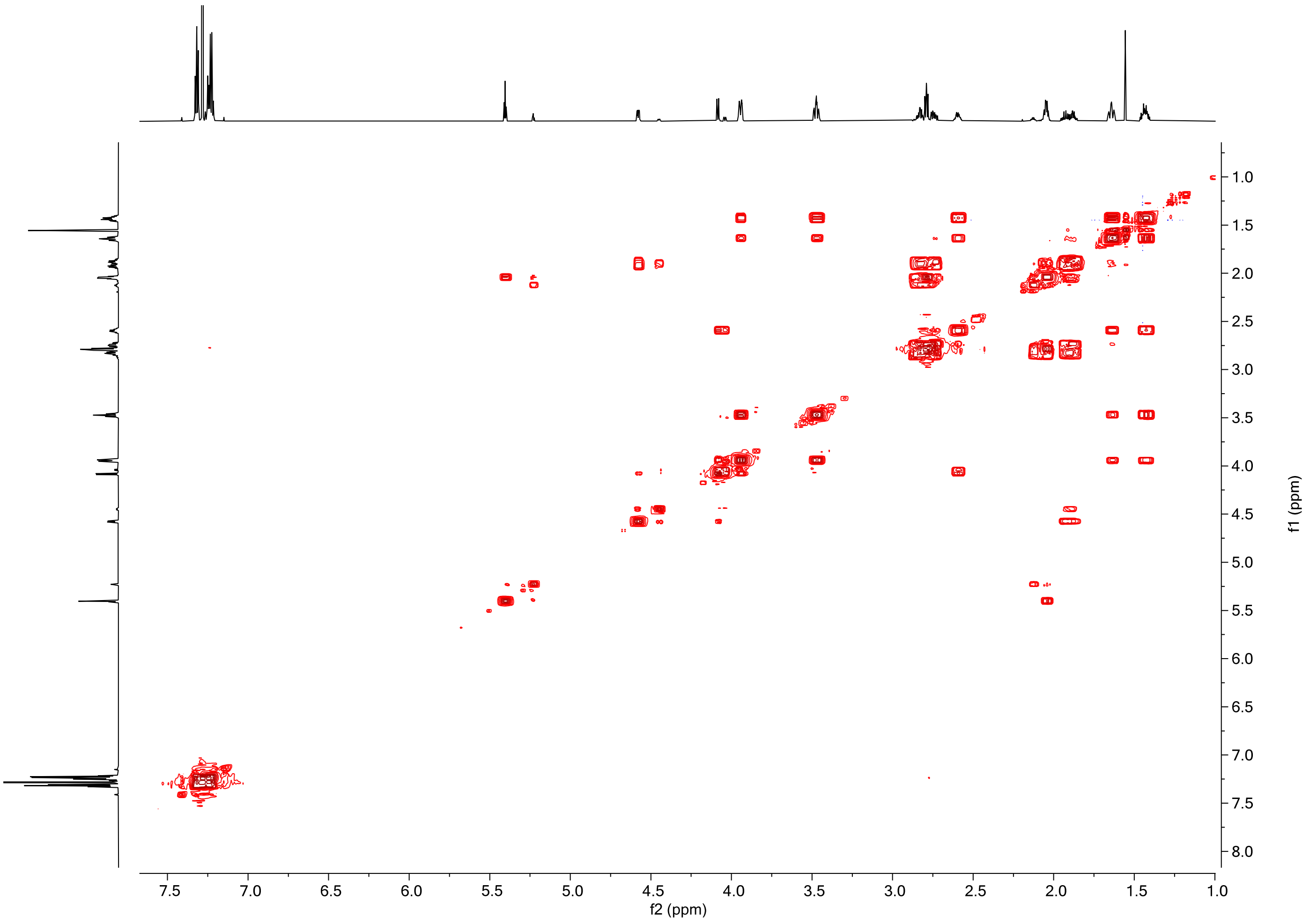

Figure S77. COSY NMR spectrum for **43** (800 MHz, $CDCl_3$, room temperature)

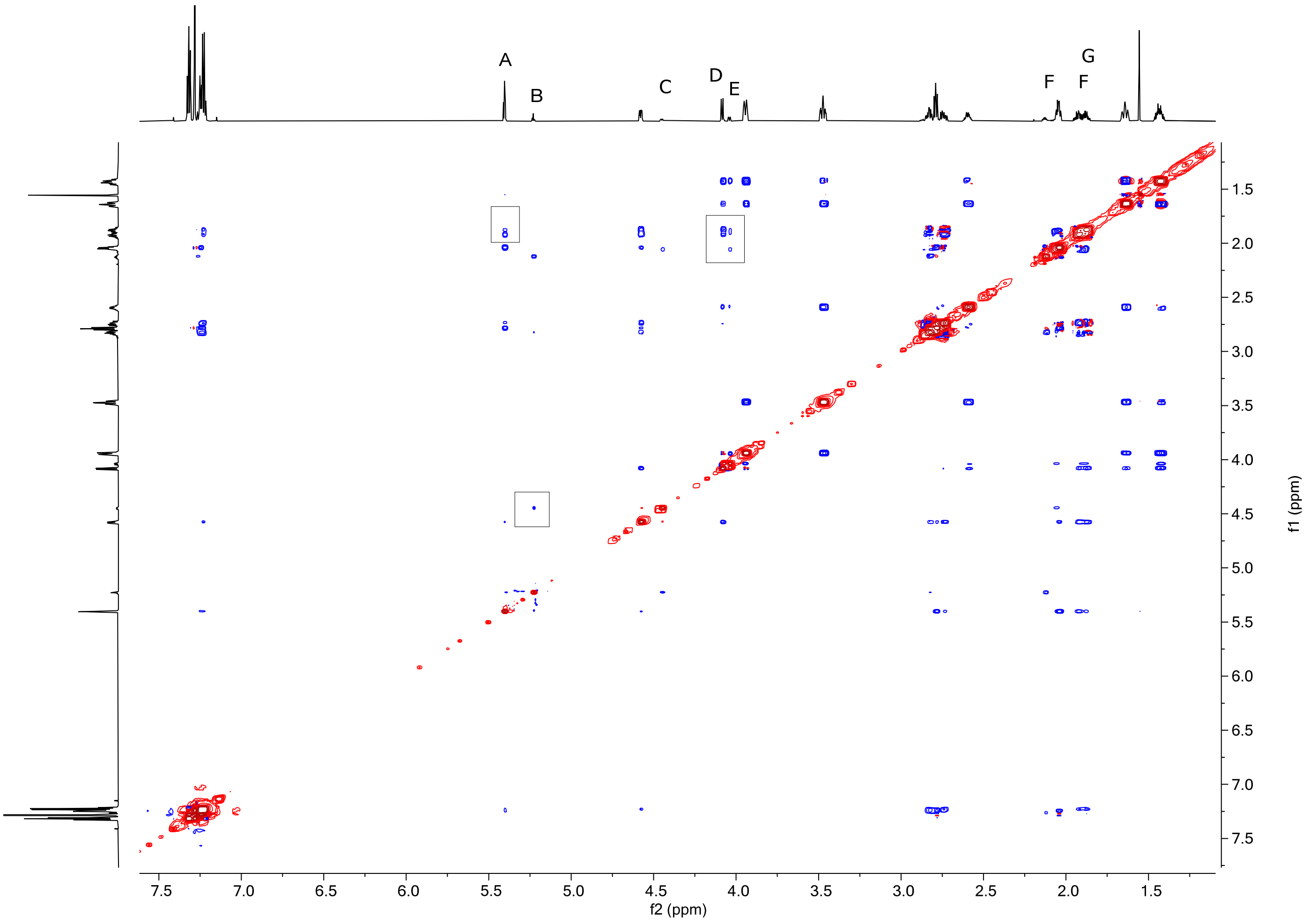

Figure S78. NOESY NMR spectrum for **43** (800 MHz, $CDCl_3$, room temperature)

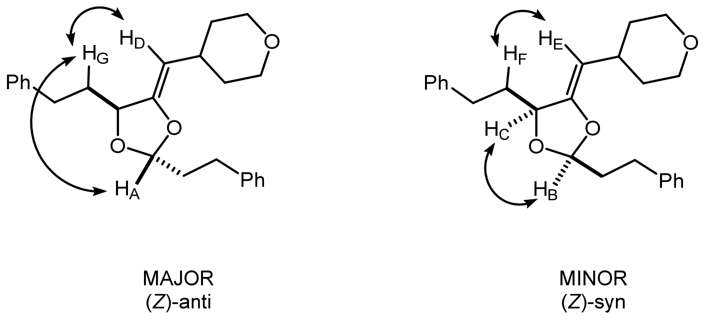

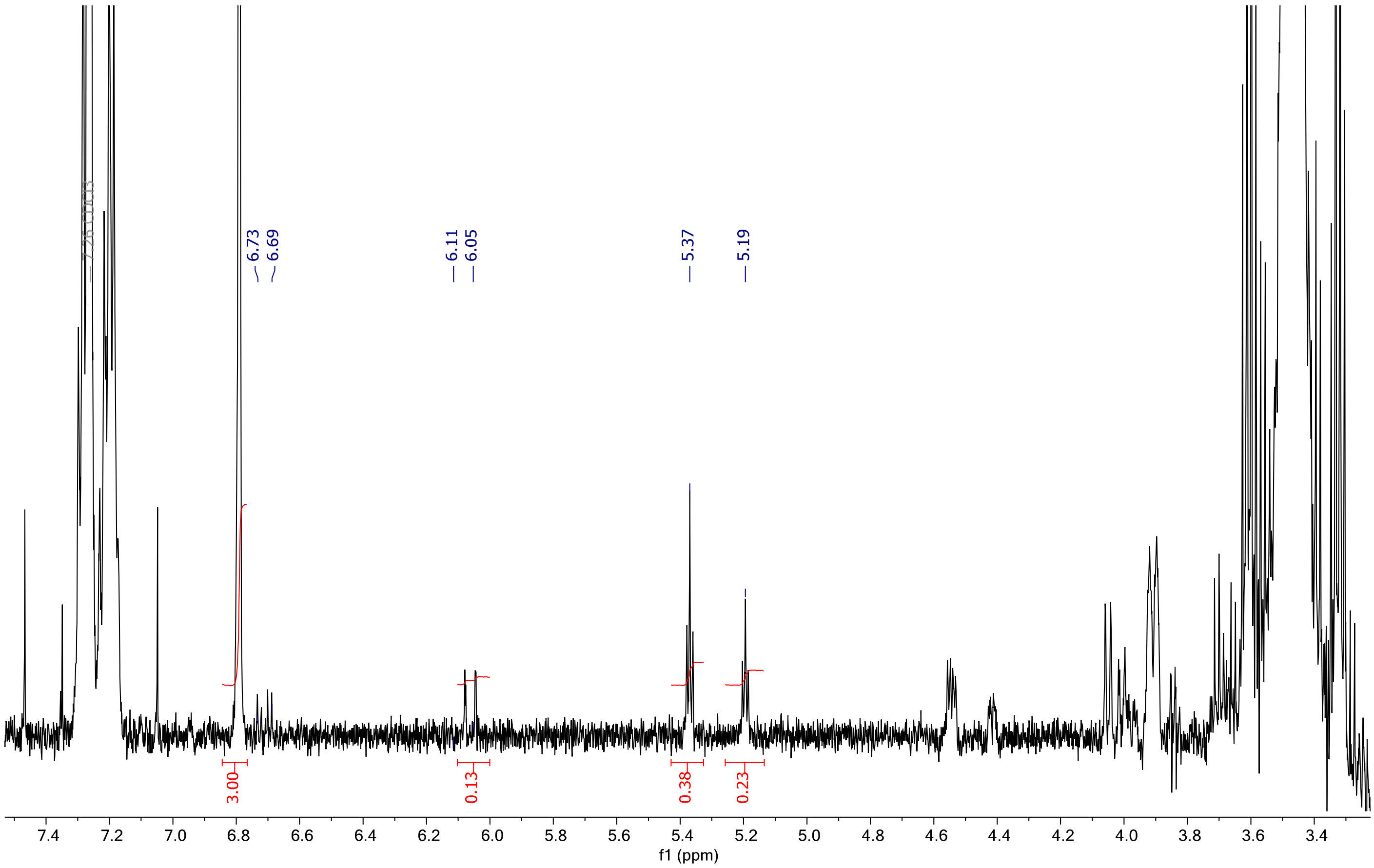

Figure S79. Crude ^{1}H NMR spectra for formation of **42** and **43,** integrated against mesitylene, under dilute catalytic conditions.

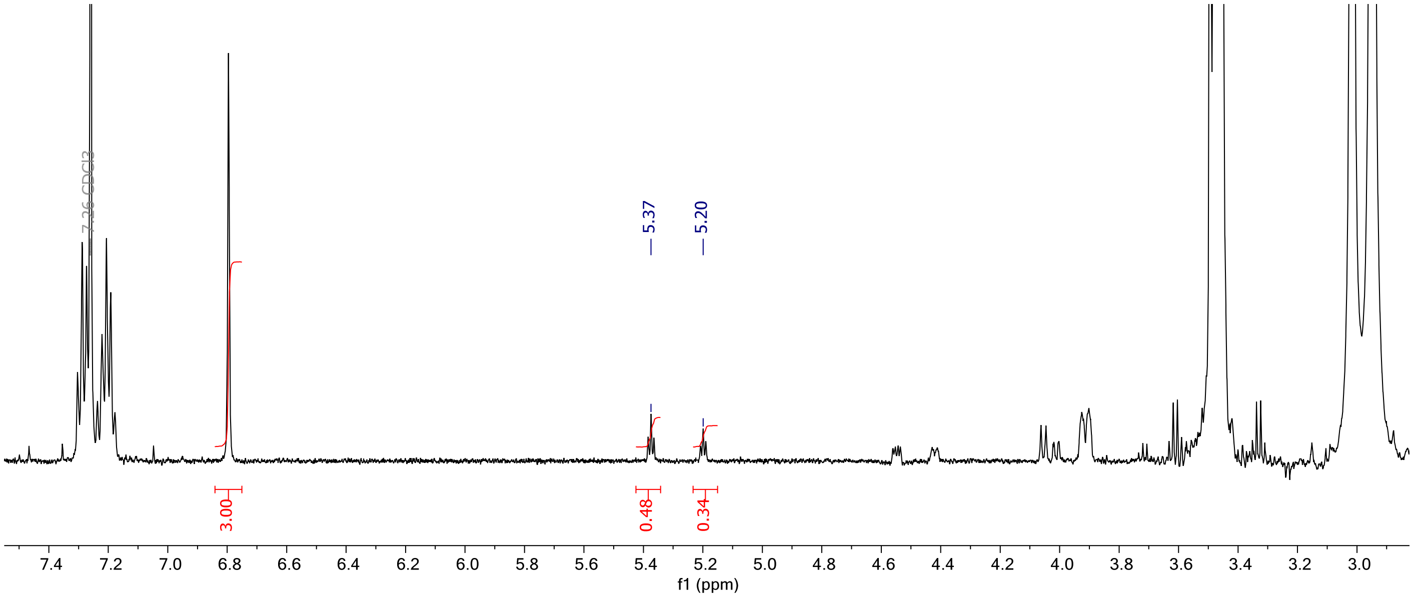

Figure S80. Crude ^{1}H NMR spectra for formation of **43,** integrated against mesitylene, under standard catalytic conditions.

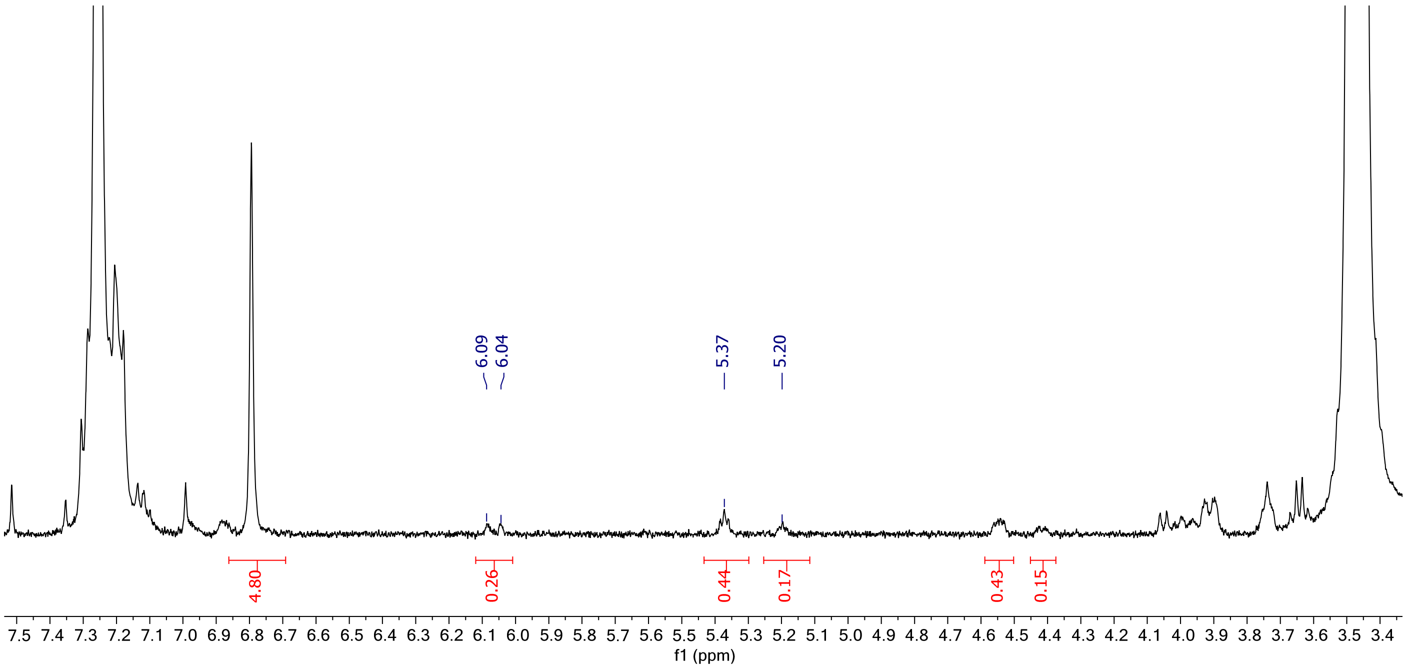

Figure S81. Crude ^{1}H NMR spectra for formation of **42** and **43,** integrated against mesitylene, under concentrated stoichiometric conditions.

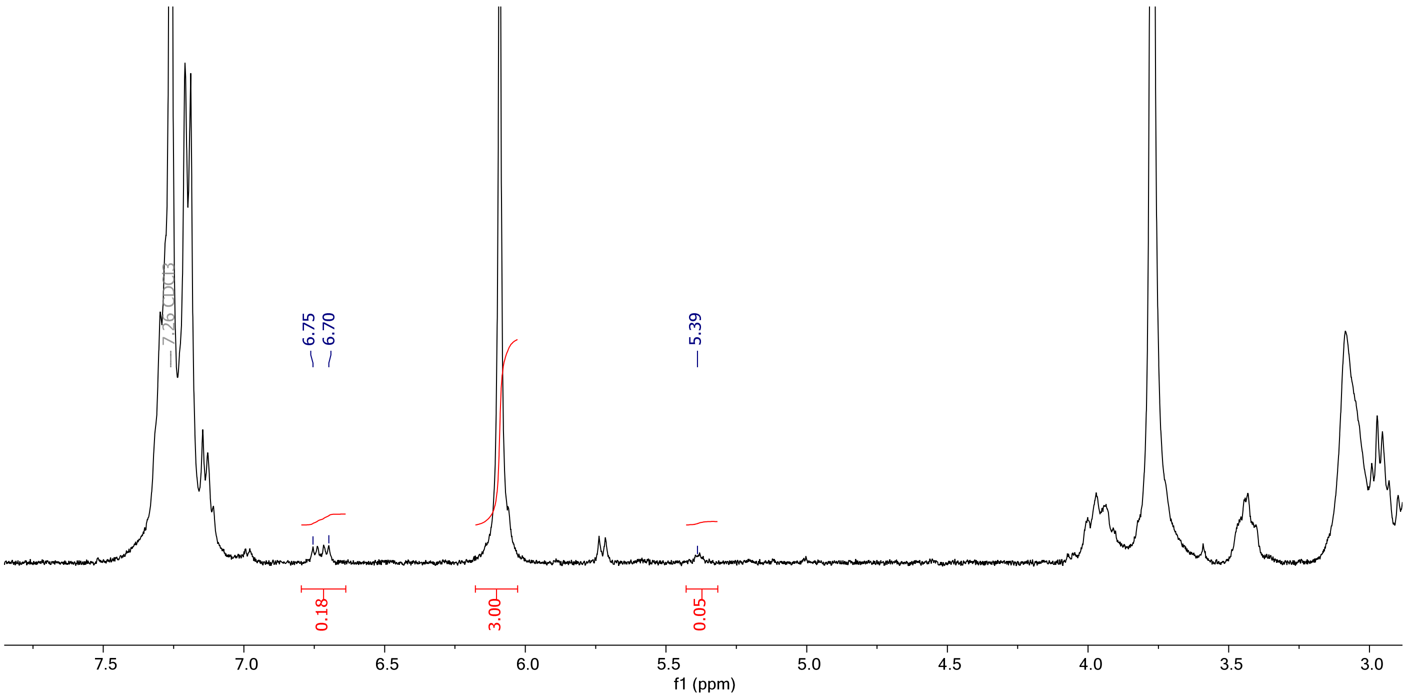

Figure S82. Crude ^{1}H NMR spectra for formation of **42** and **43,** integrated against trimethoxybenzene, under dilute stoichiometric conditions.

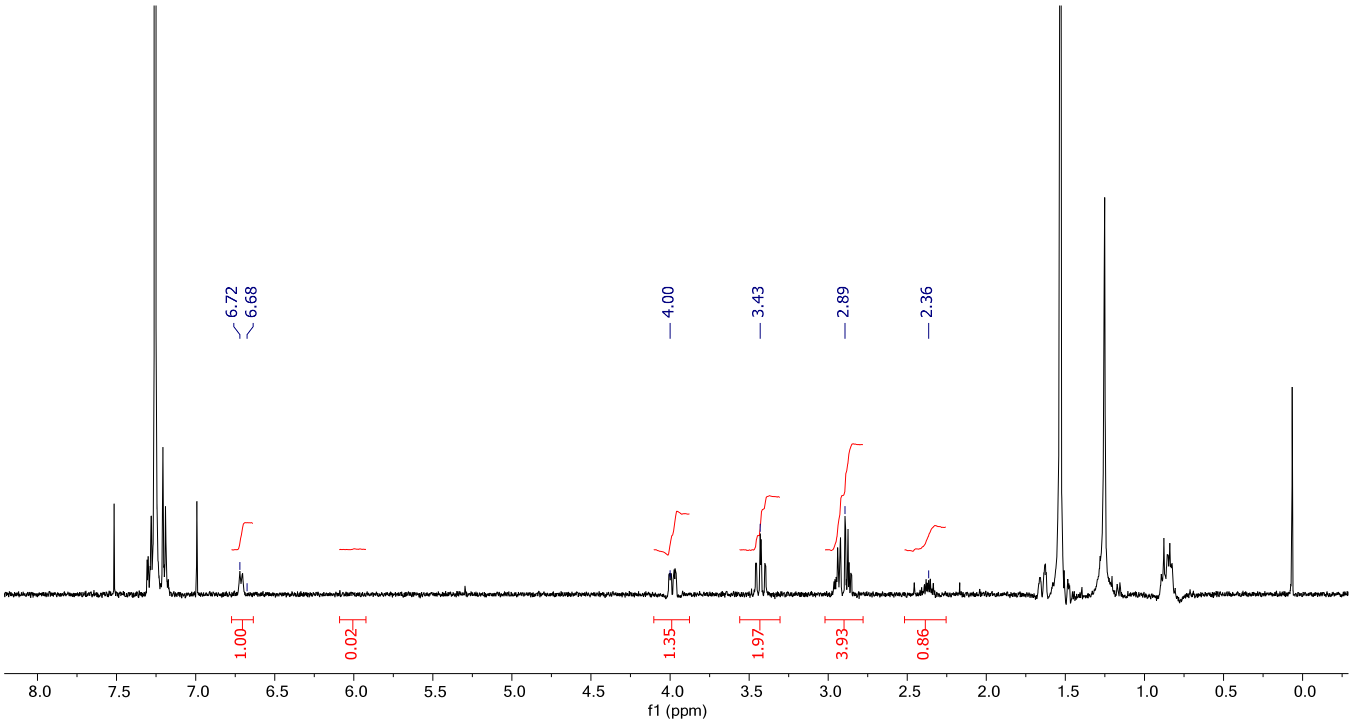

Figure S83. ^{1}H NMR spectra showing deuterium incorporation of **42-*d***.

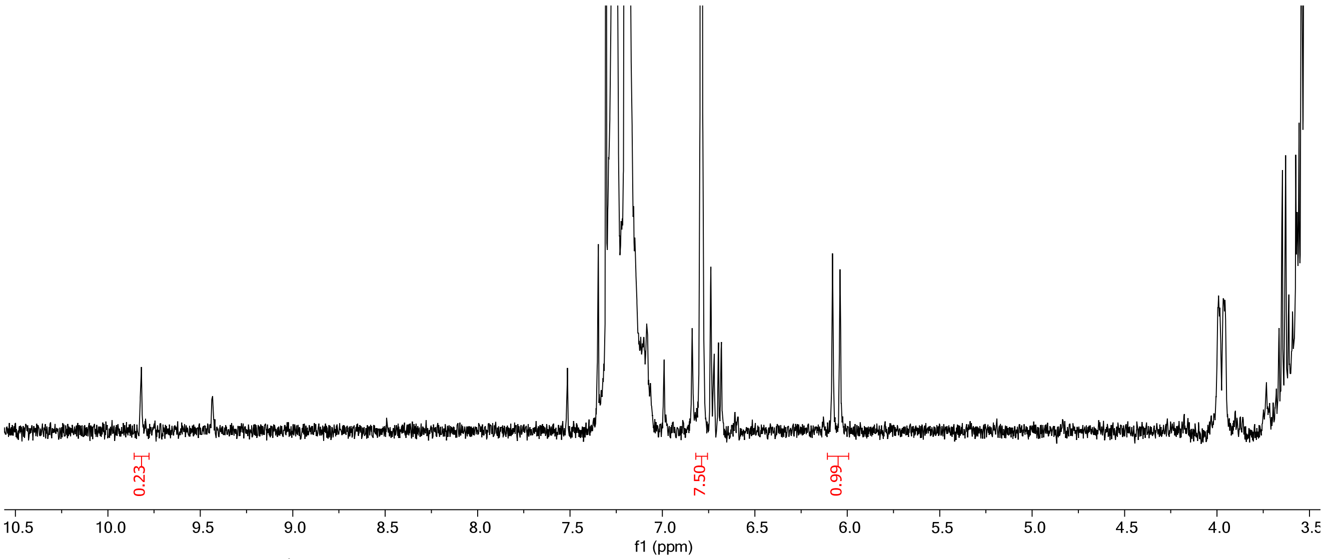

Figure S84. Crude ^{1}H NMR spectra showing conversion of **41** and **42,** integrated against mesitylene.

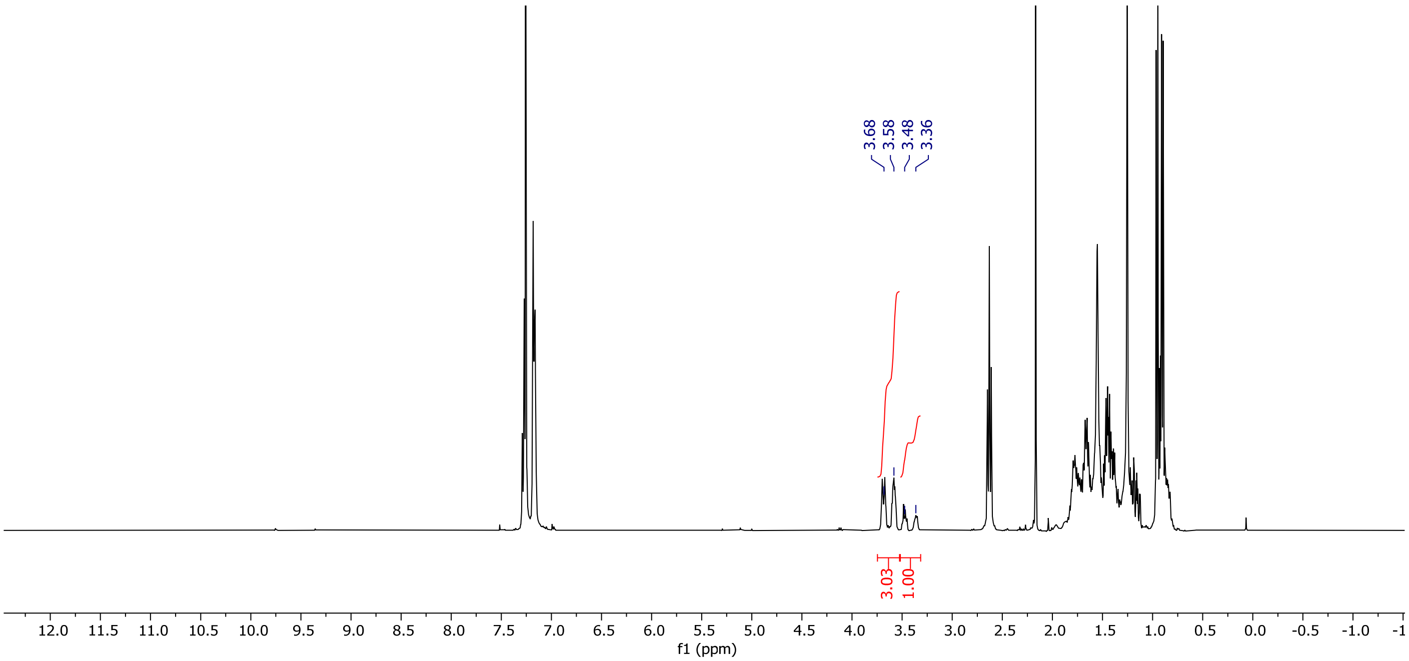

Figure S85. ^{1}H NMR spectra showing the diastereomeric ratio of compound **6**.

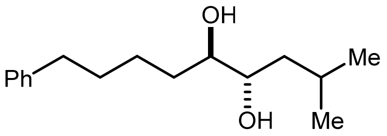

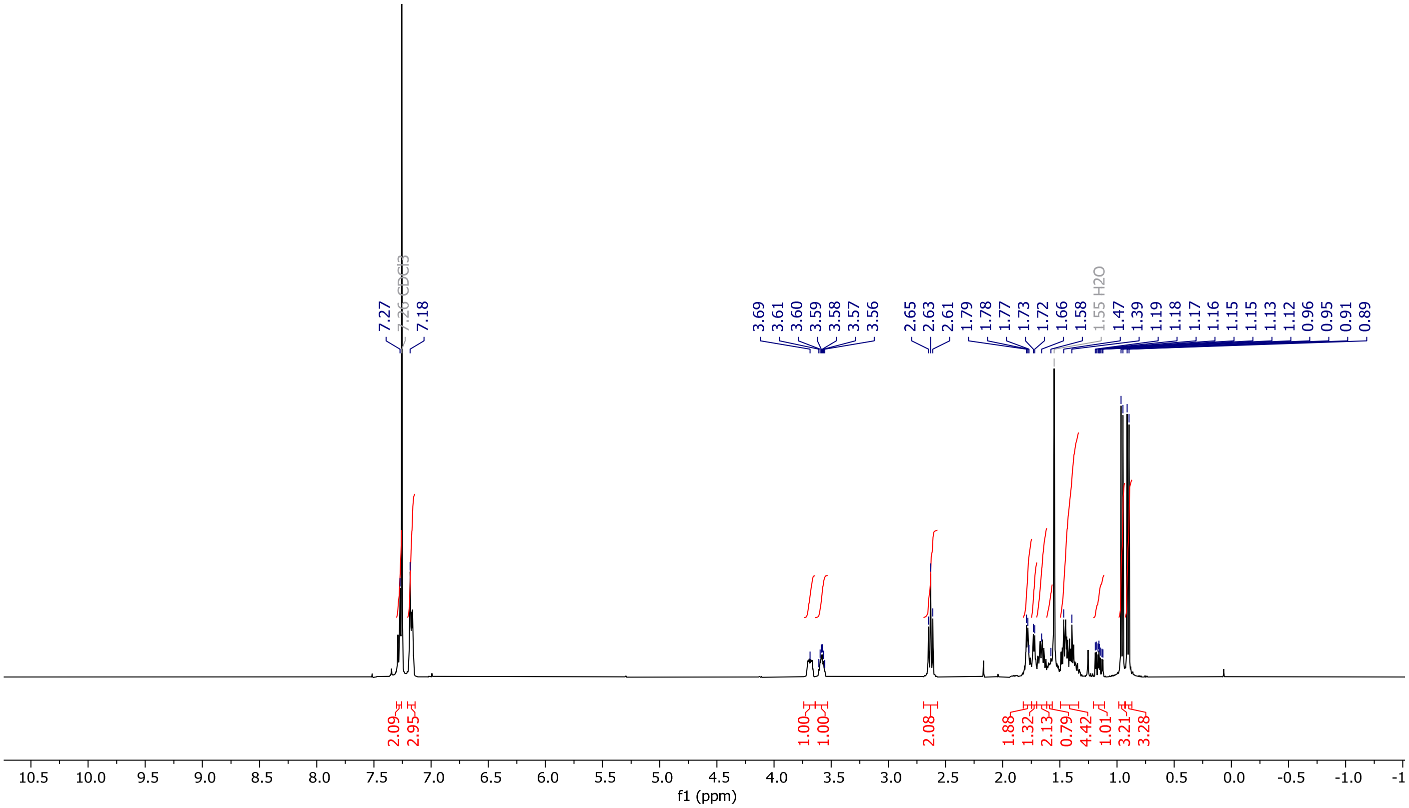

Figure S86. ^{1}H NMR spectrum for *anti*-**6** (400 MHz, $CDCl_3$, room temperature).

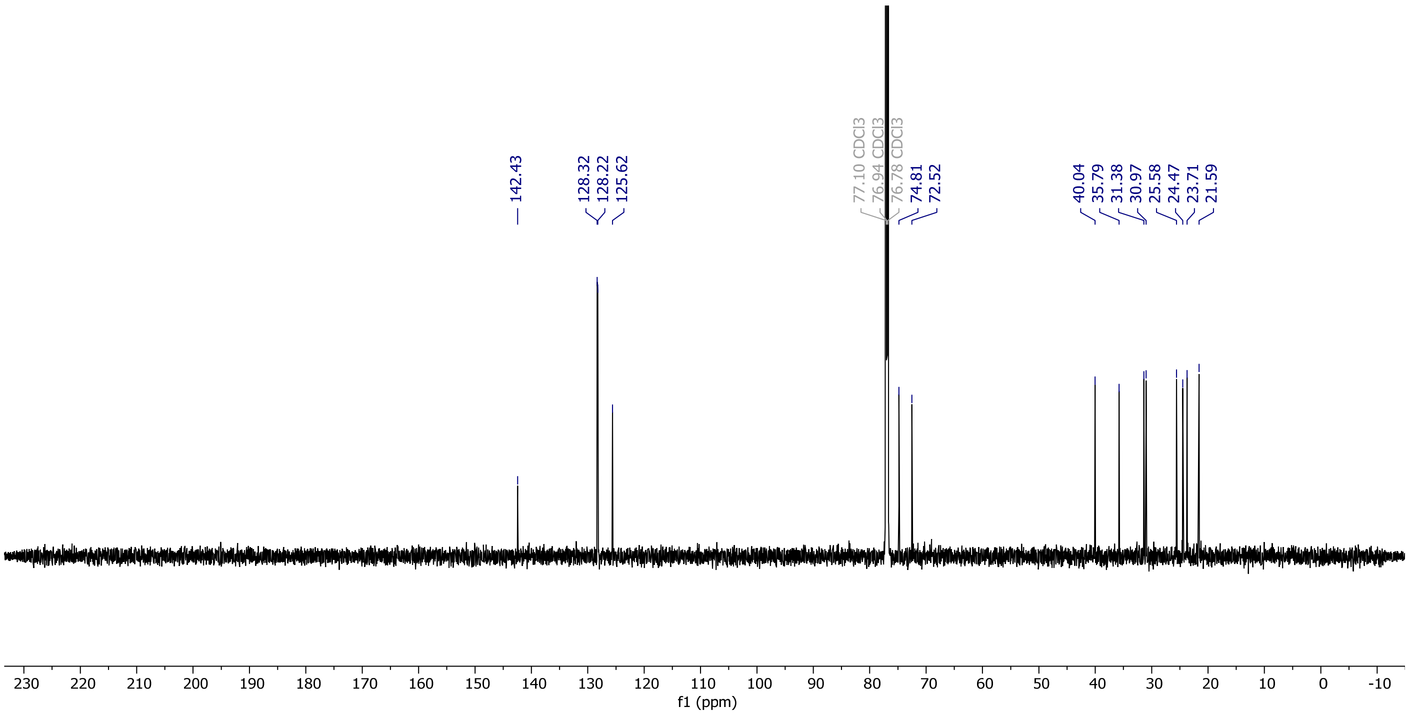

Figure S87. $^{13}C\{^{1}H\}$ NMR spectrum for *anti*-**6** (201 MHz, $CDCl_3$, room temperature)

6. FT-IR Data

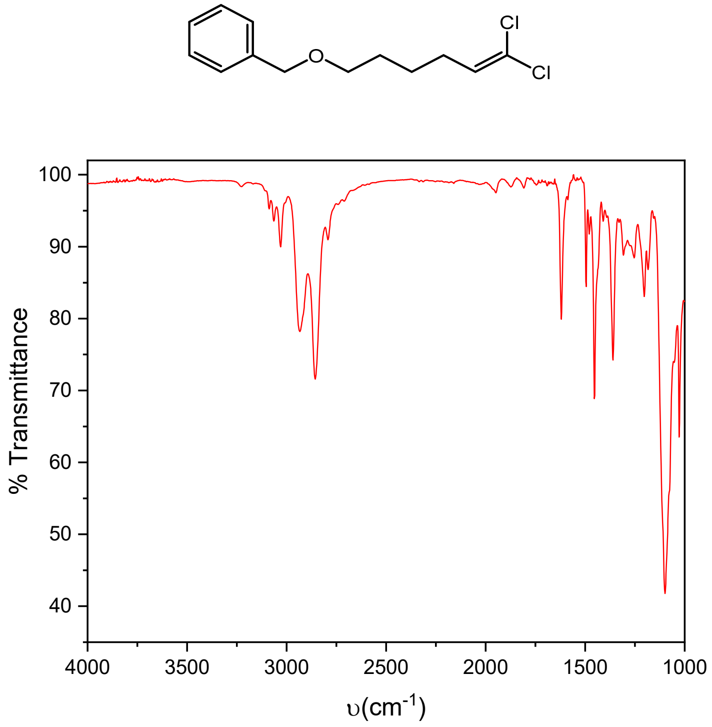

Figure S88. FT-IR spectrum for **S1**.

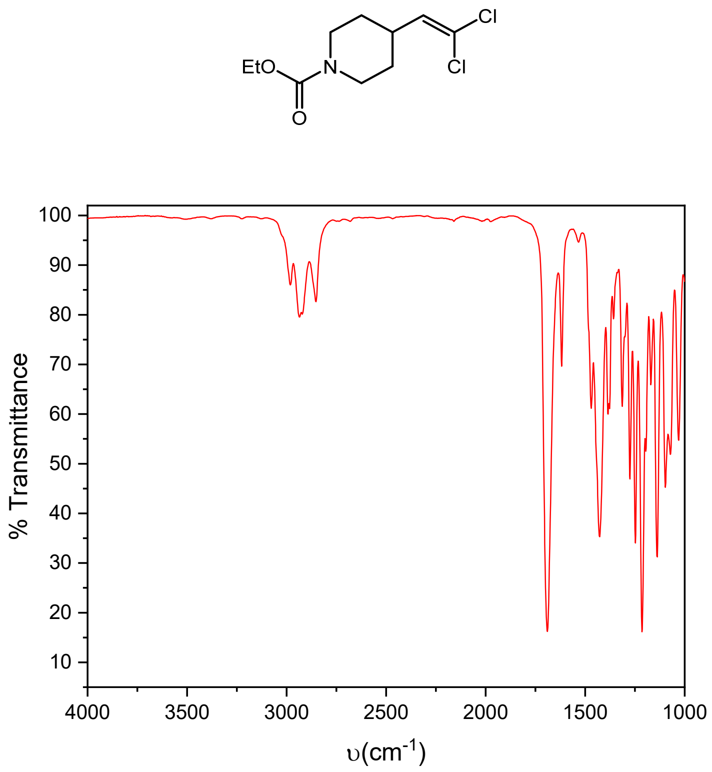

Figure S89. FT-IR spectrum for **S2**.

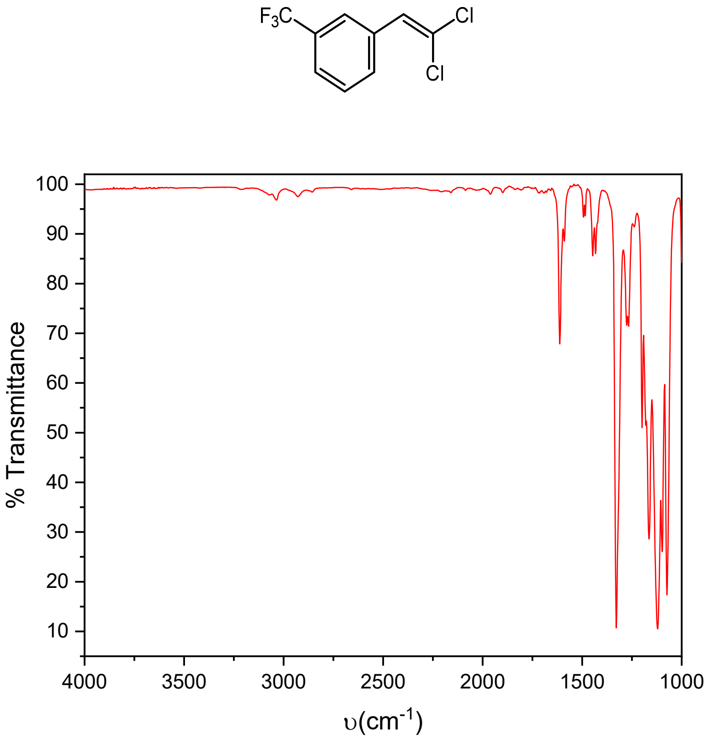

Figure S90. FT-IR spectrum for **S3**.

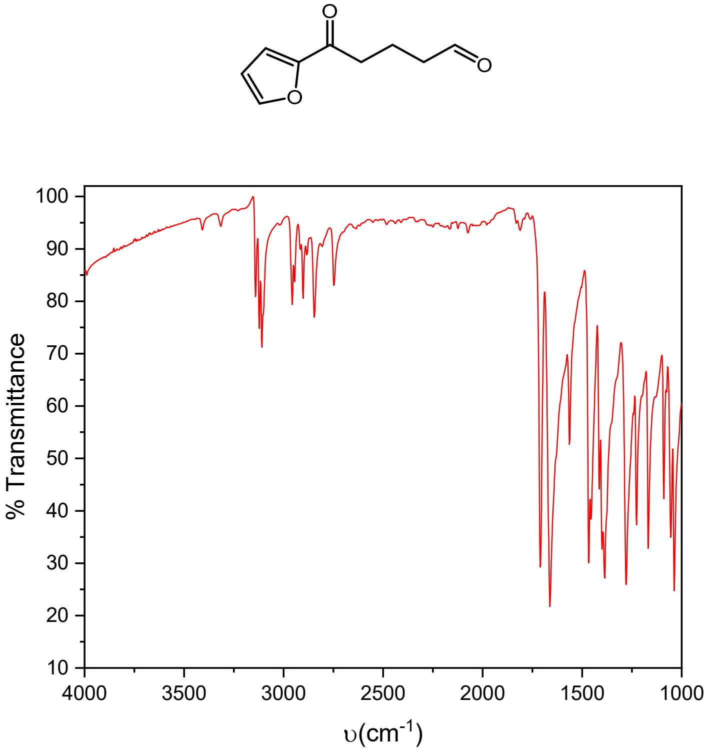

Figure S91. FT-IR spectrum for **S4**.

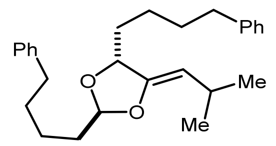

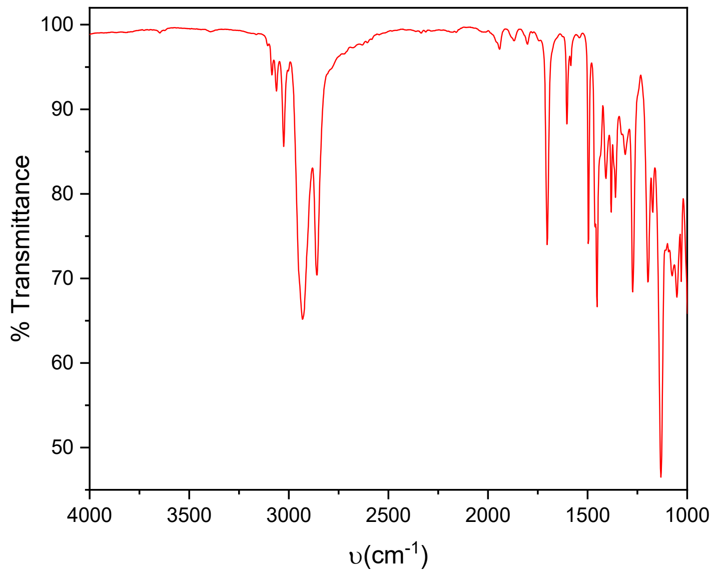

Figure S92. FT-IR spectrum for **4**.

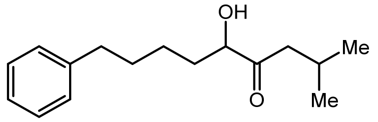

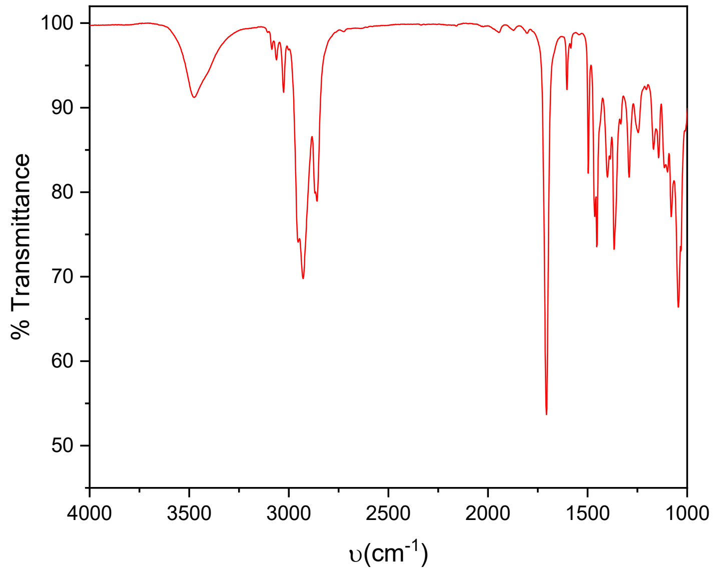

Figure S93. FT-IR spectrum for **5**.

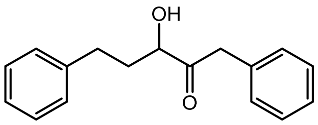

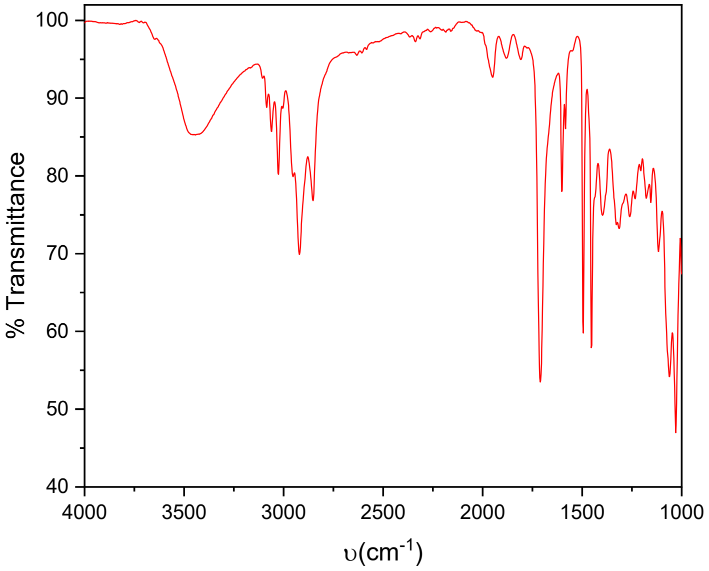

Figure S94. FT-IR spectrum for **13**.

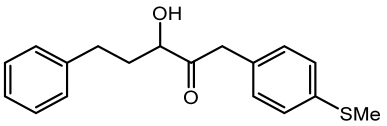

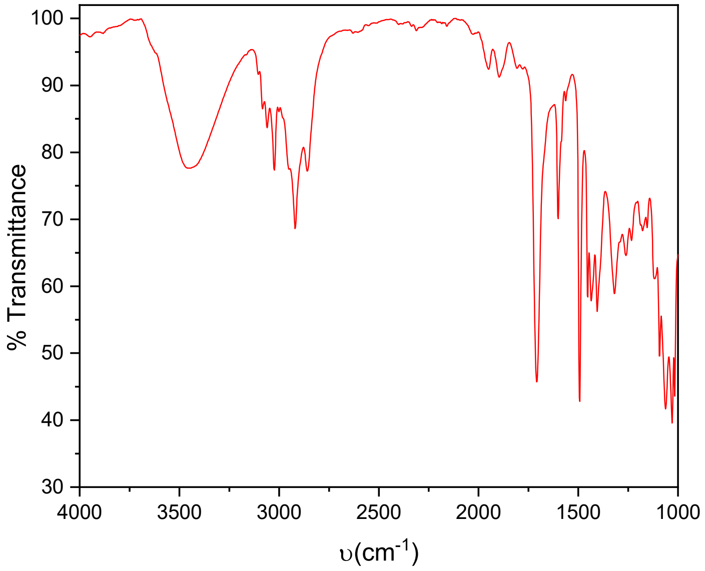

Figure S95. FT-IR spectrum for **14**.

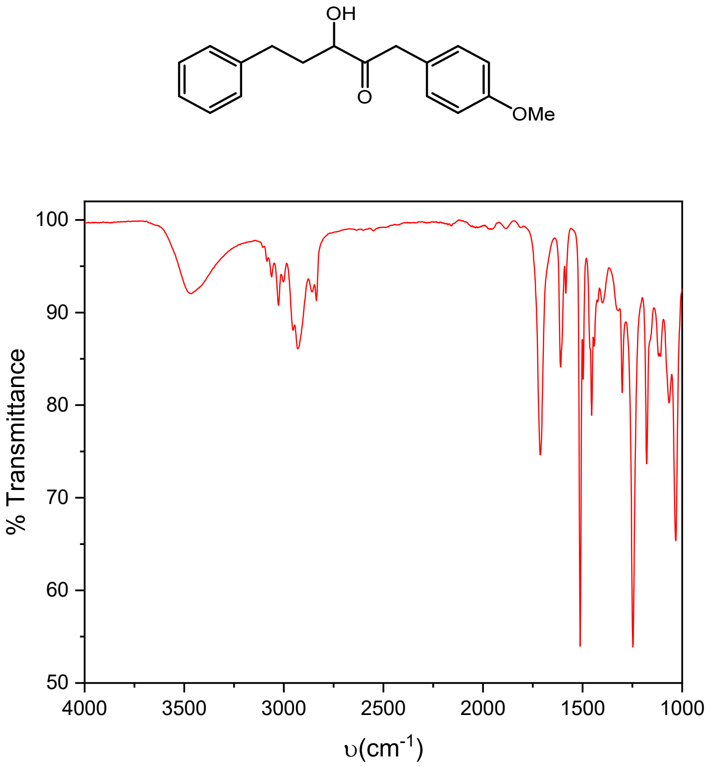

Figure S96. FT-IR spectrum for **15**.

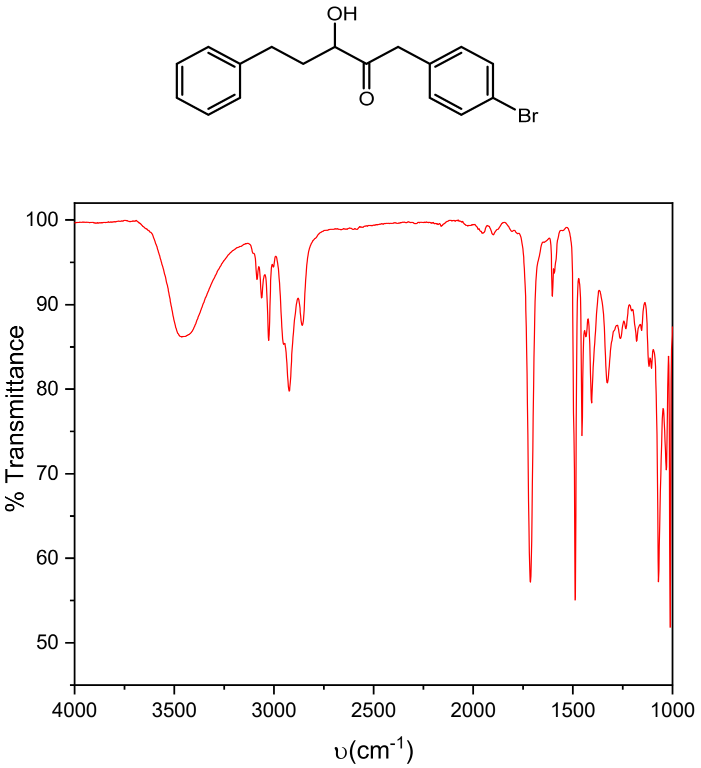

Figure S97. FT-IR spectrum for **16**.

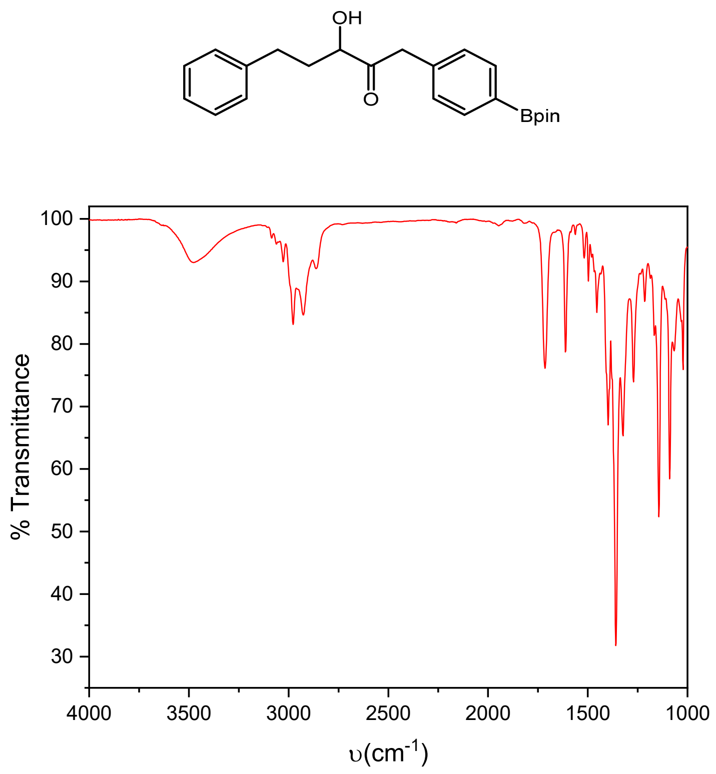

Figure S98 FT-IR spectrum for **17**.

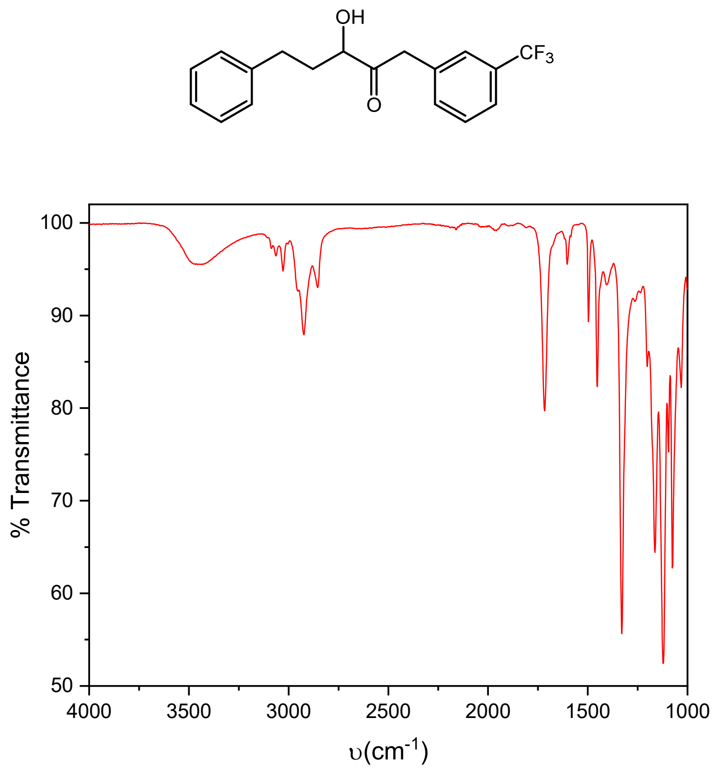

Figure S99. FT-IR spectrum for **18**.

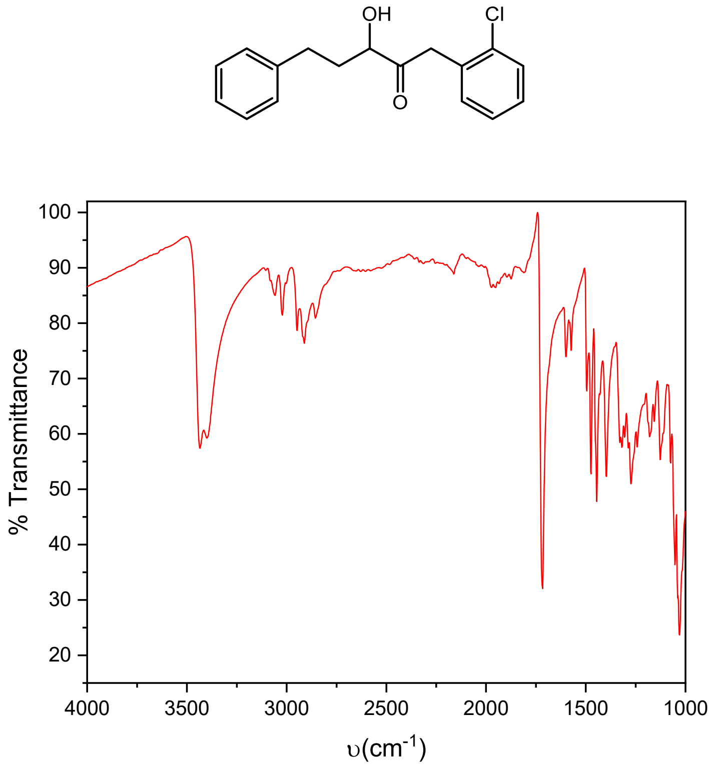

Figure S100. FT-IR spectrum for **19**.

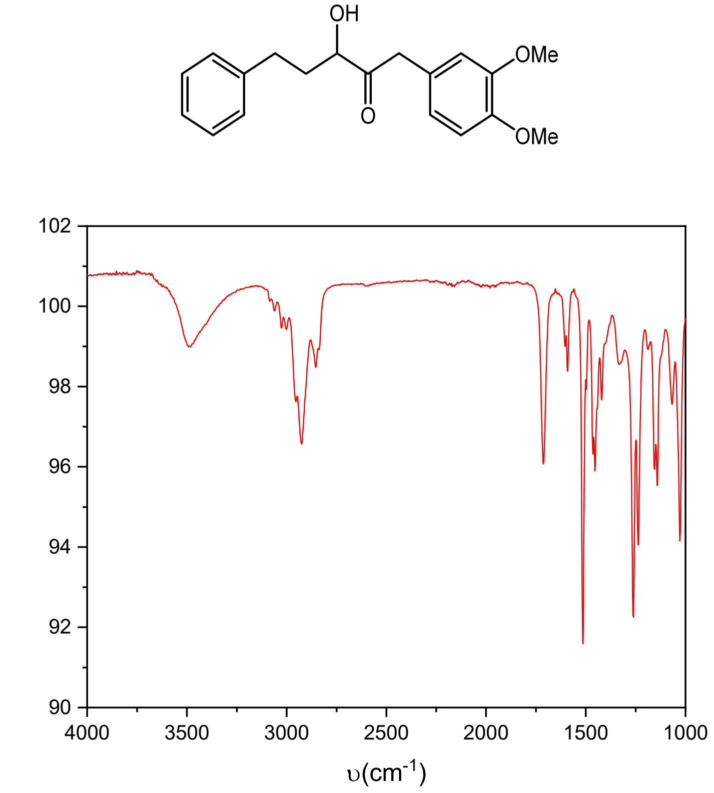

Figure S101. FT-IR spectrum for **20**.

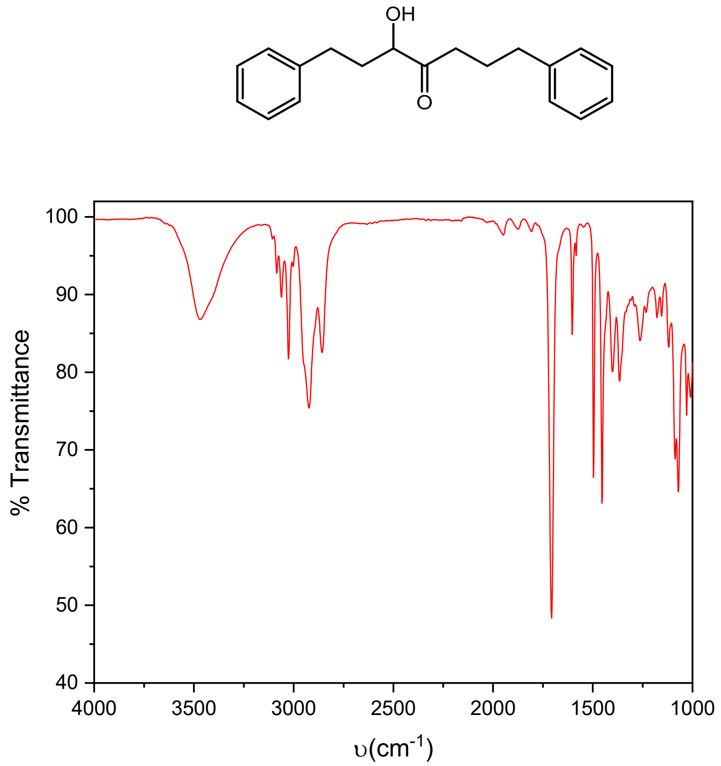

Figure S102. FT-IR spectrum for **21**.

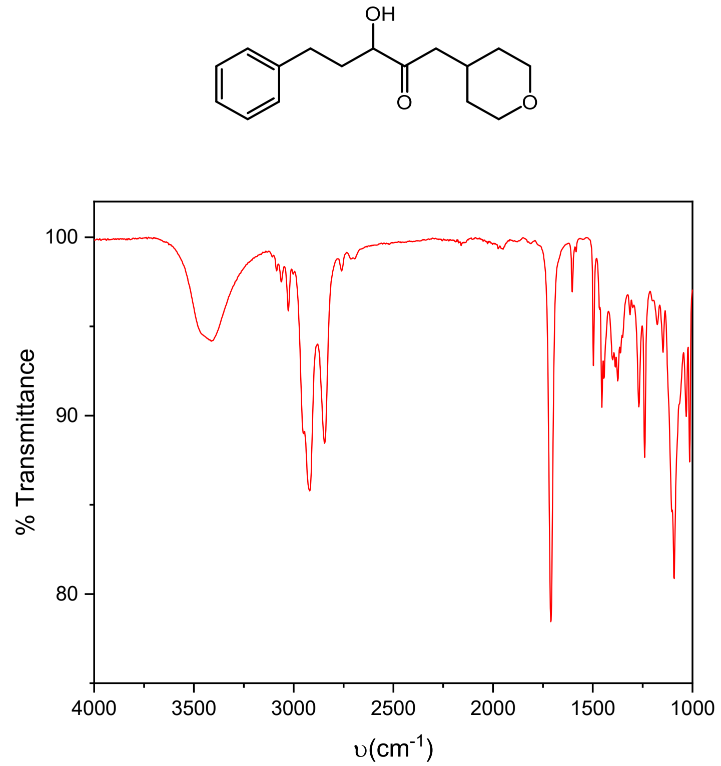

Figure S103. FT-IR spectrum for **22**.

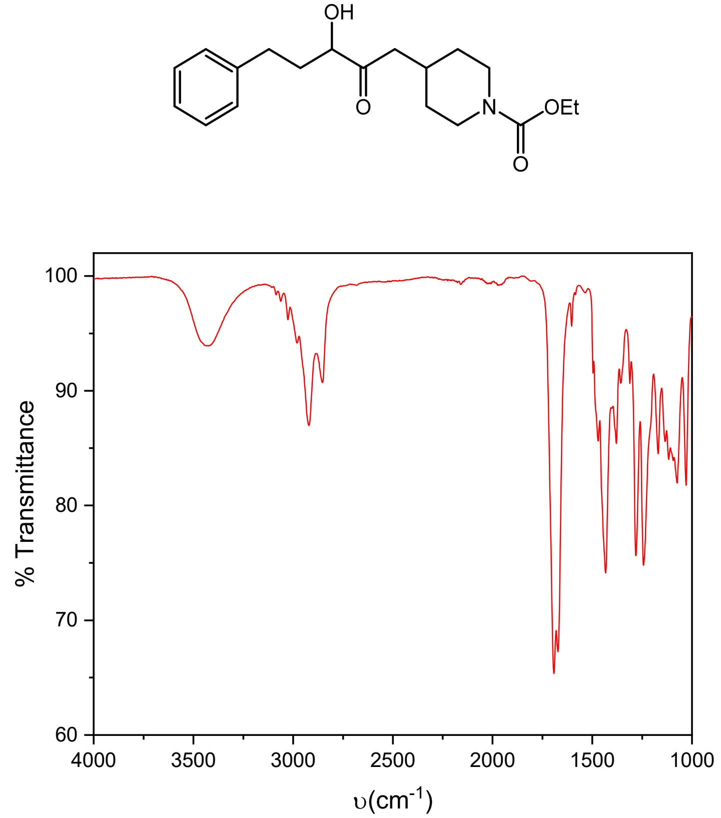

Figure S104. FT-IR spectrum for **23**.

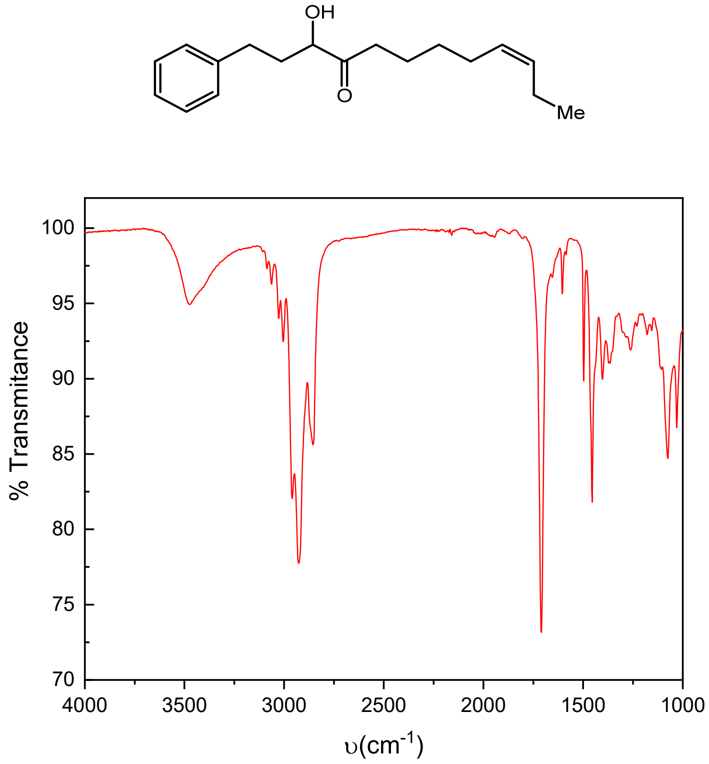

Figure S105. FT-IR spectrum for **24**.

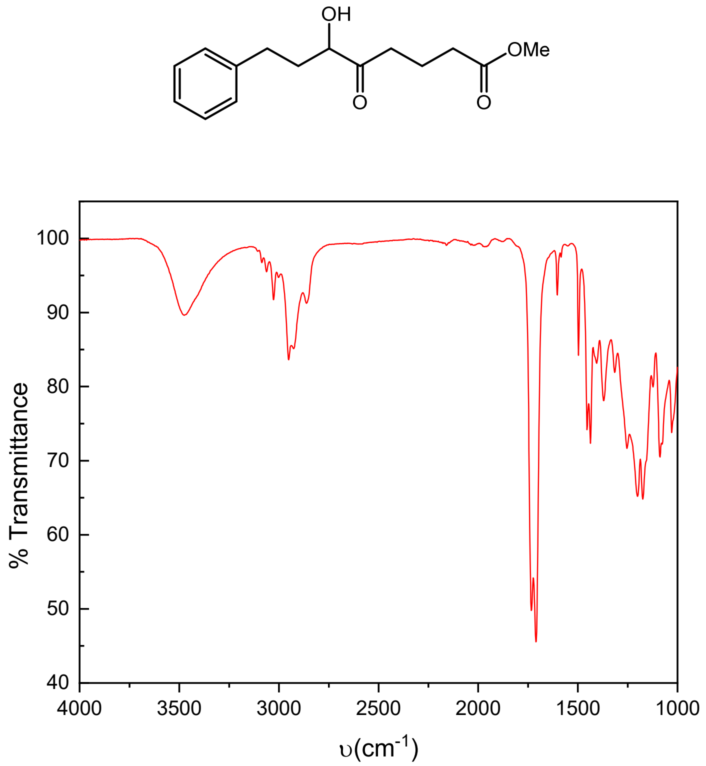

Figure S106. FT-IR spectrum for **25**.

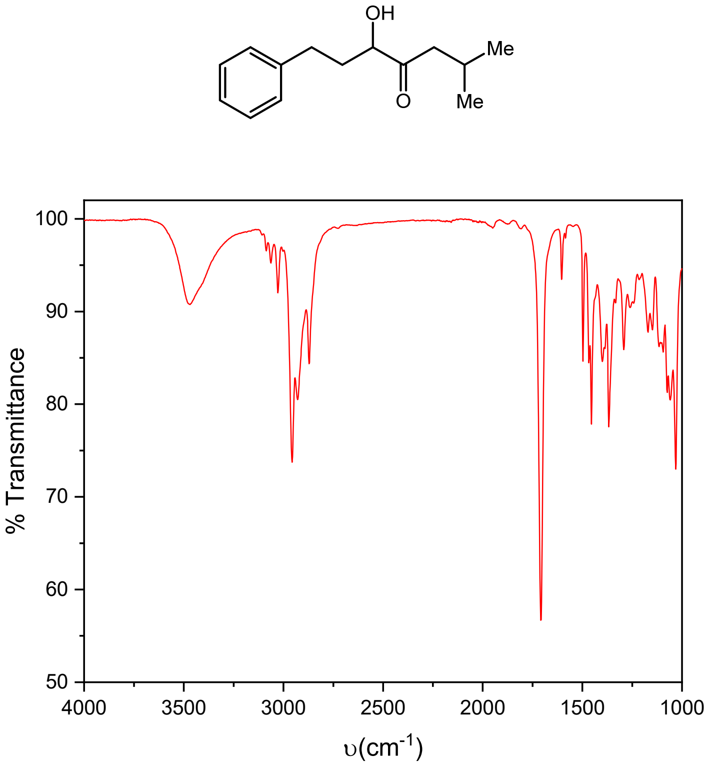

Figure S107. FT-IR spectrum for **26**.

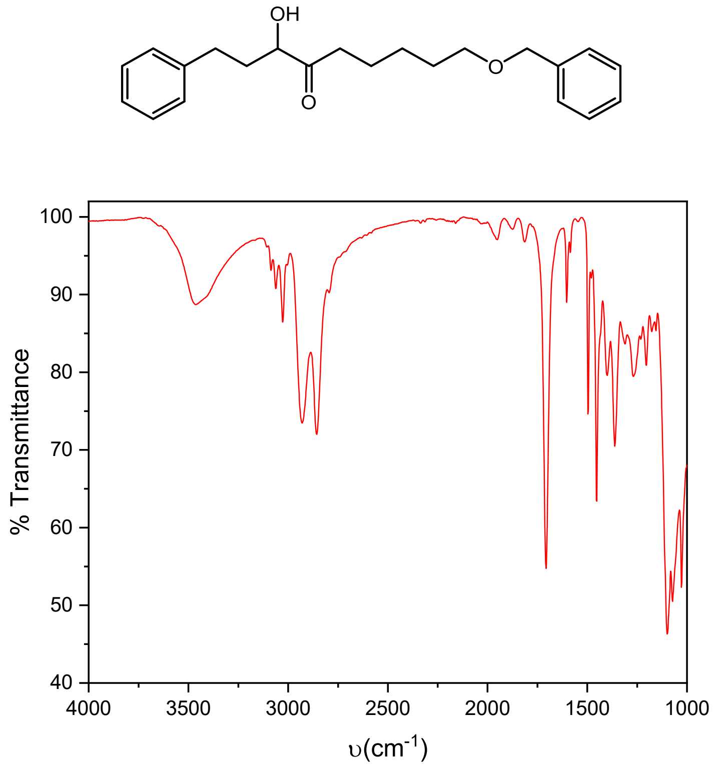

Figure S108. FT-IR spectrum for **27**.

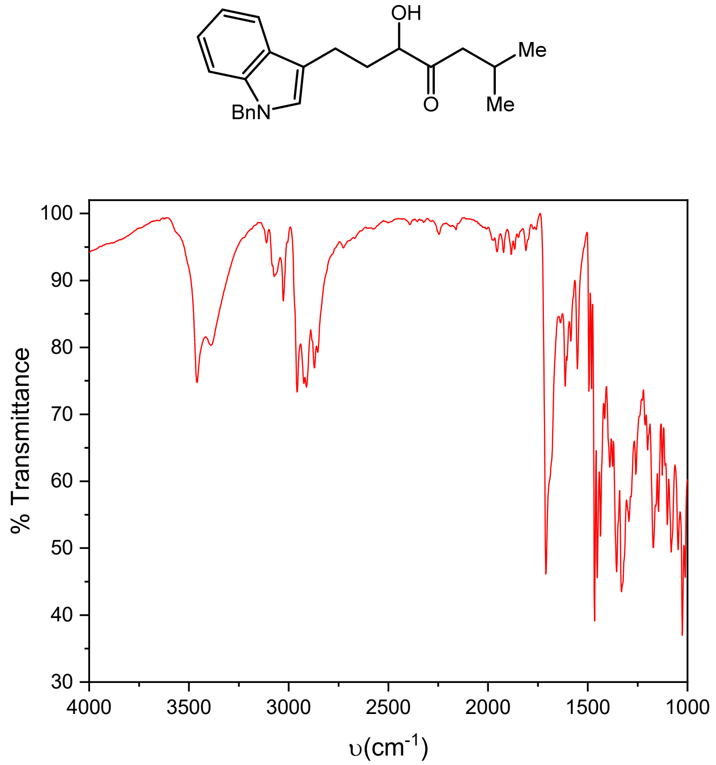

Figure S109. FT-IR spectrum for **28**.

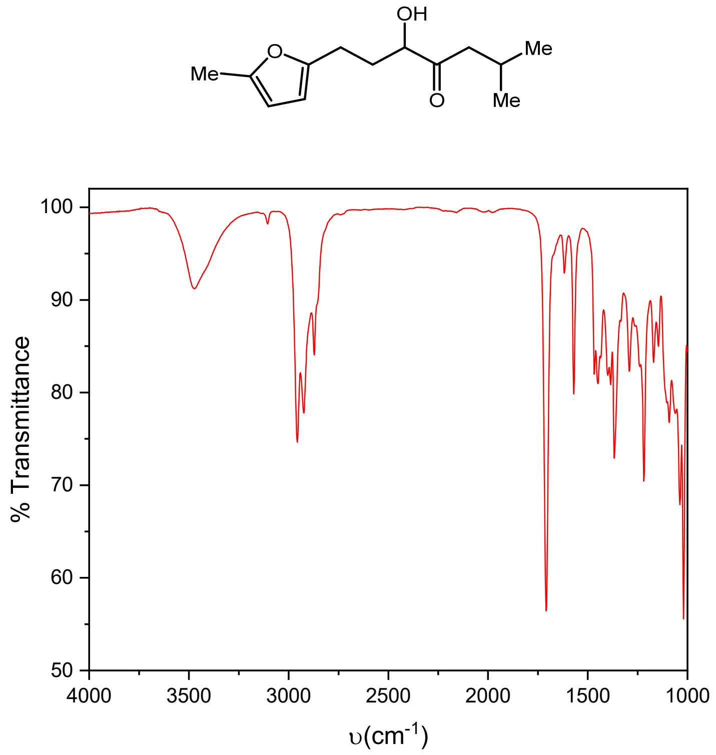

Figure S110. FT-IR spectrum for **29**.

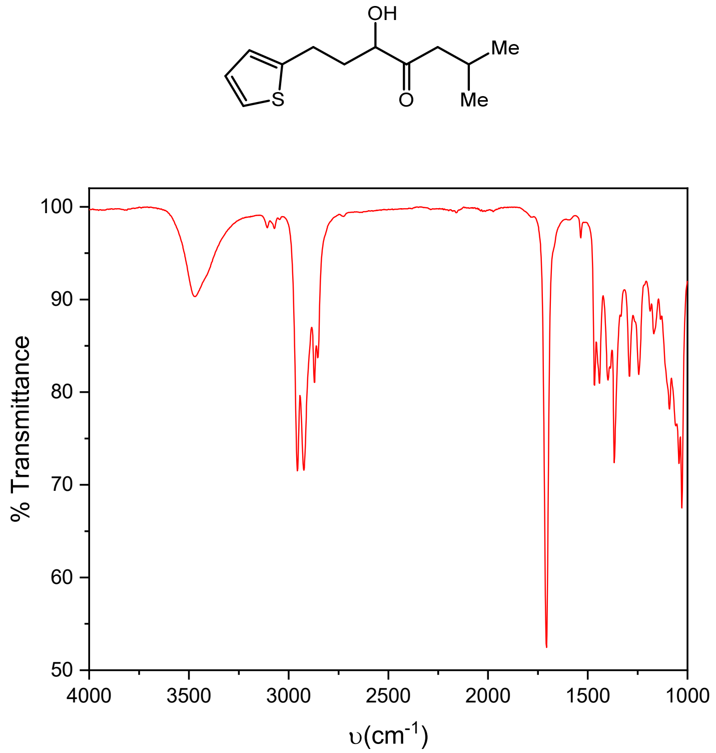

Figure S111. FT-IR spectrum for **30**.

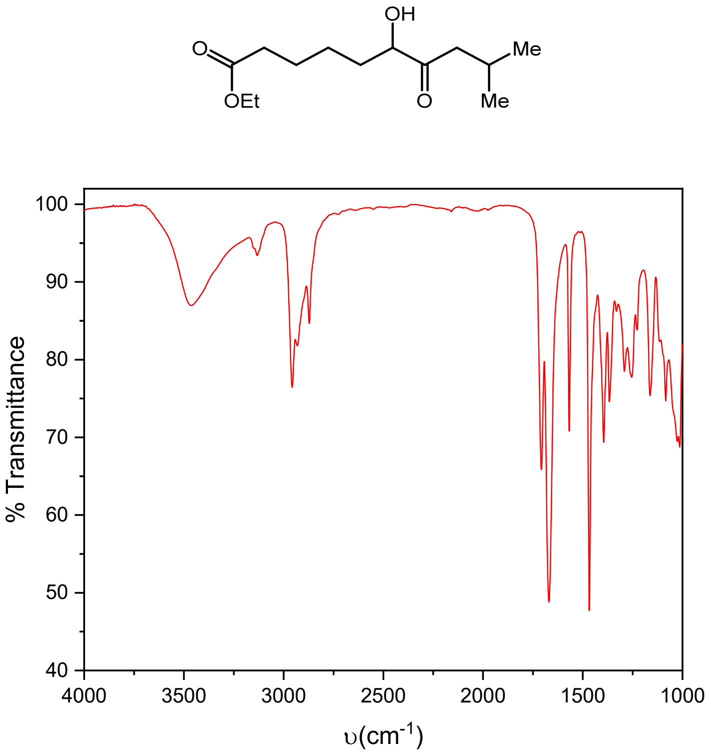

Figure S112. FT-IR spectrum for **31**.

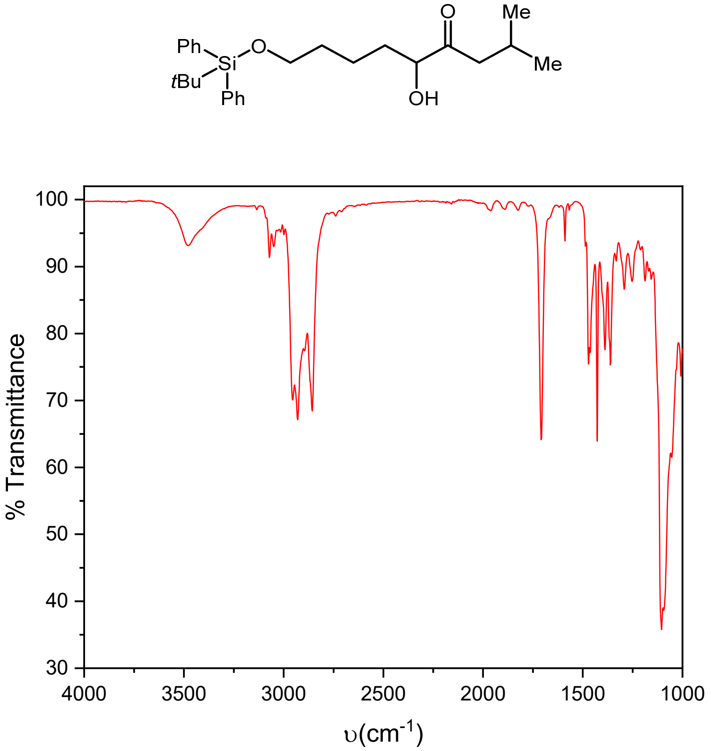

Figure S113. FT-IR spectrum for **32**.

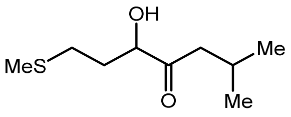

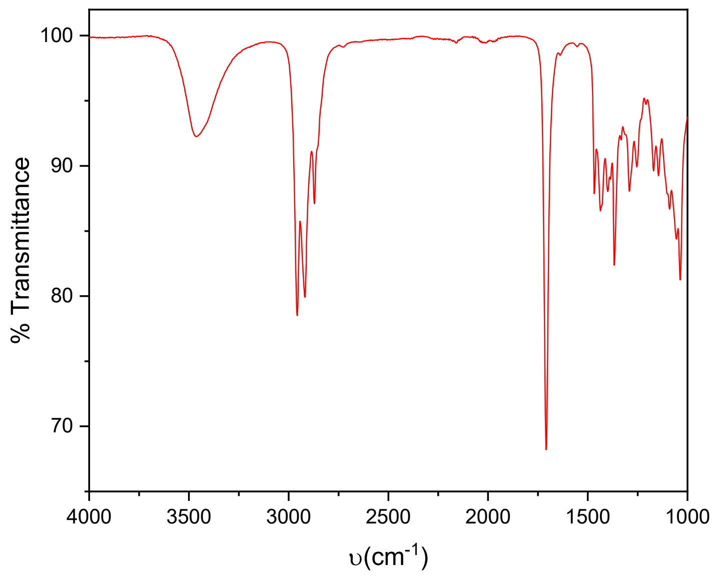

Figure S114. FT-IR spectrum for **34**.

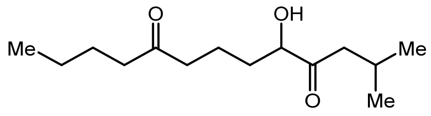

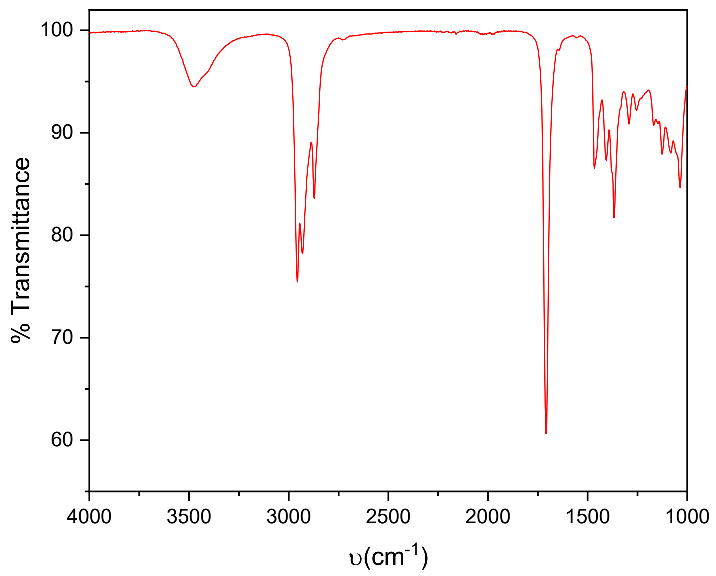

Figure S115. FT-IR spectrum for **35**.

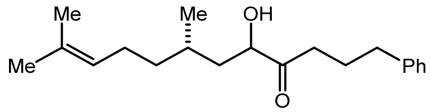

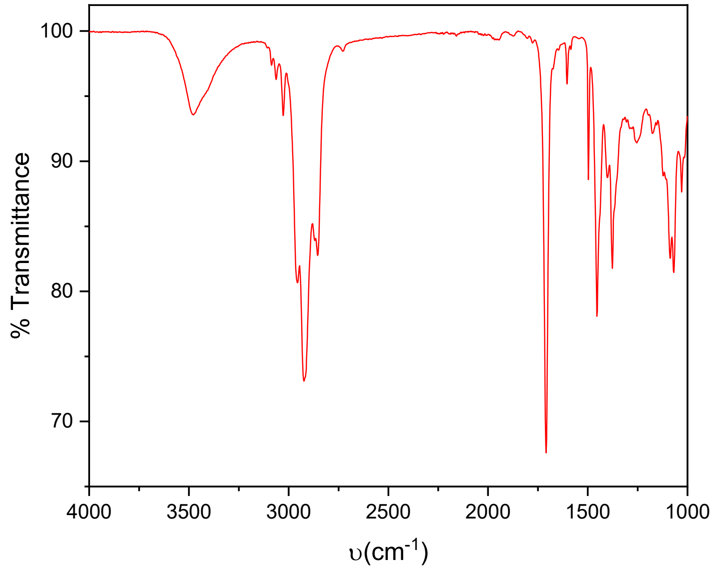

Figure S116. FT-IR spectrum for **36**.

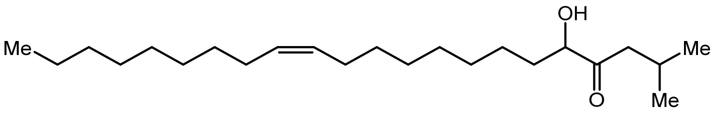

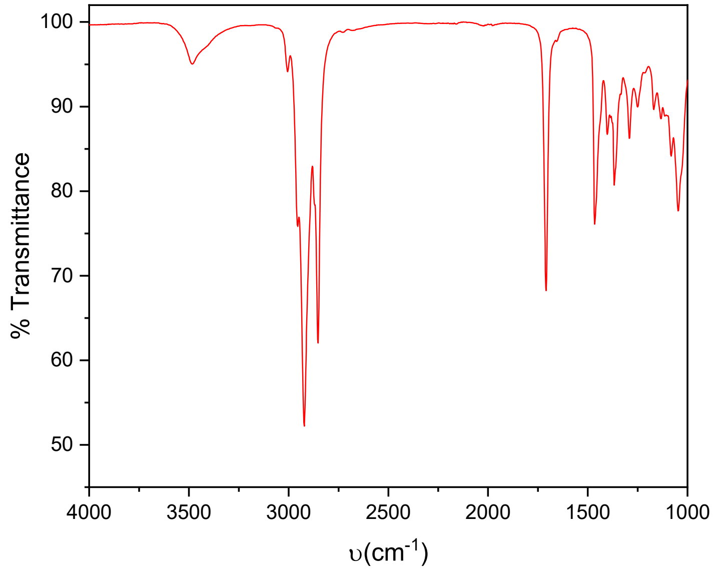

Figure S117. FT-IR spectrum for **37**.

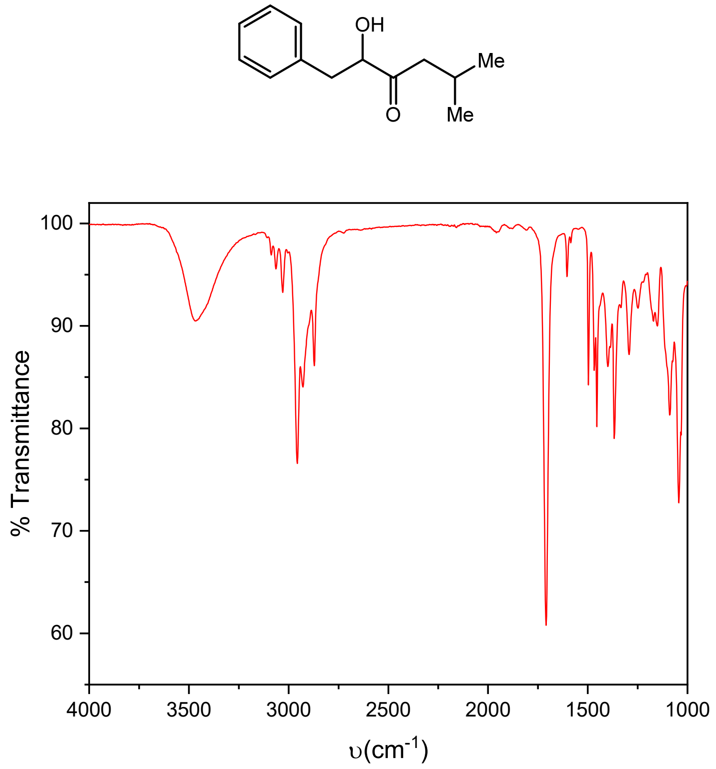

Figure S118. FT-IR spectrum for **38**.

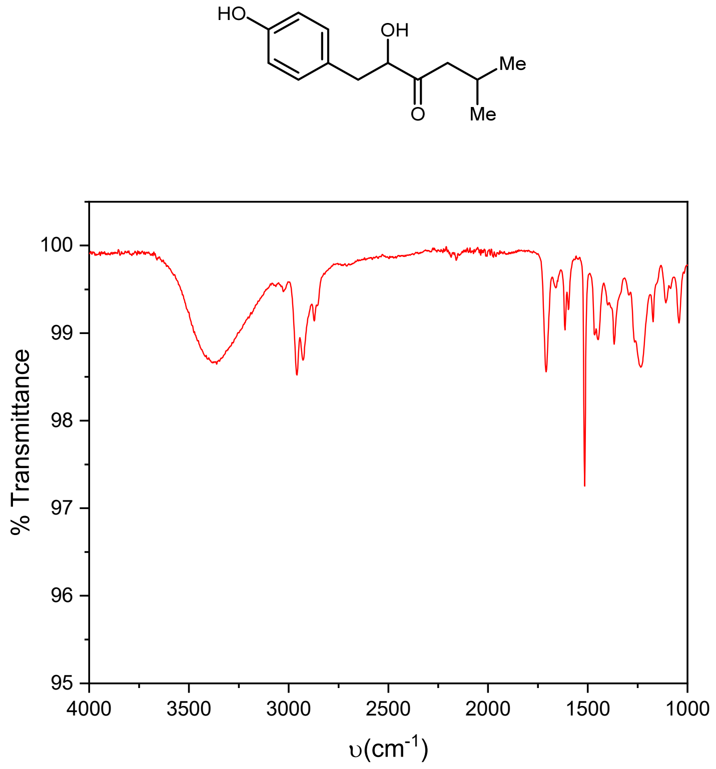

Figure S119. FT-IR spectrum for **39**.

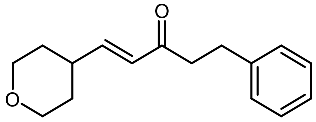

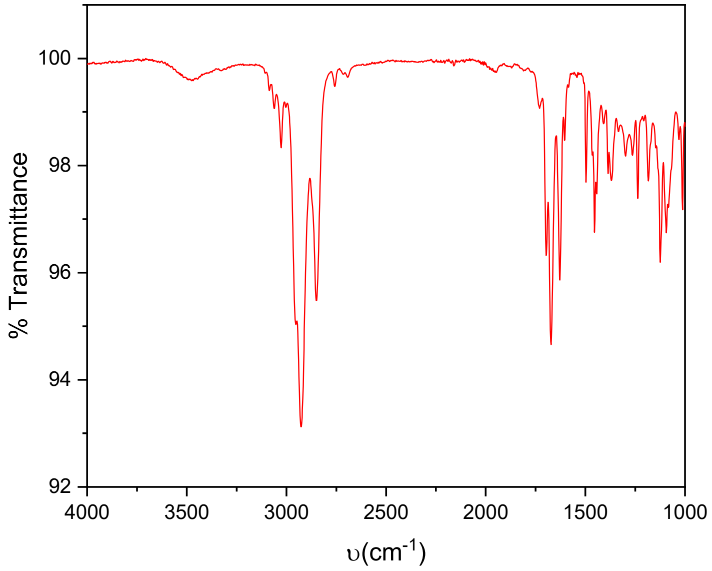

Figure S120. FT-IR spectrum for **42**.

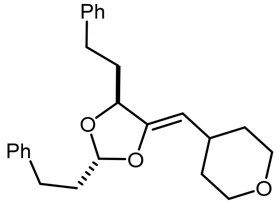

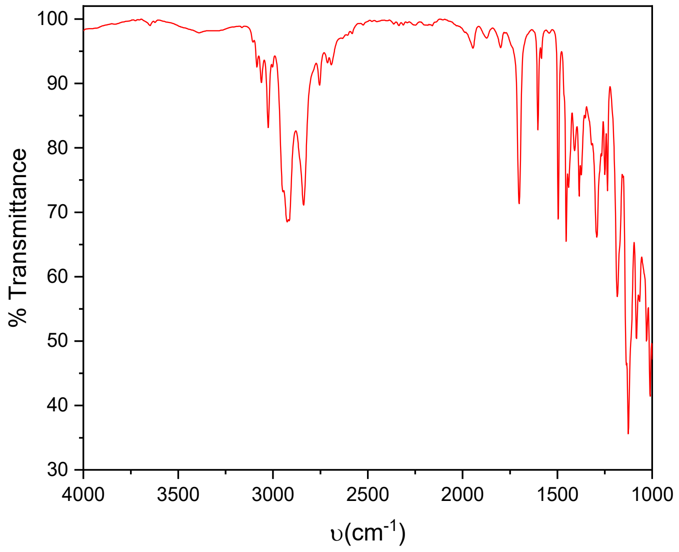

Figure S121. FT-IR spectrum for **43.**

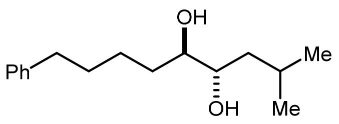

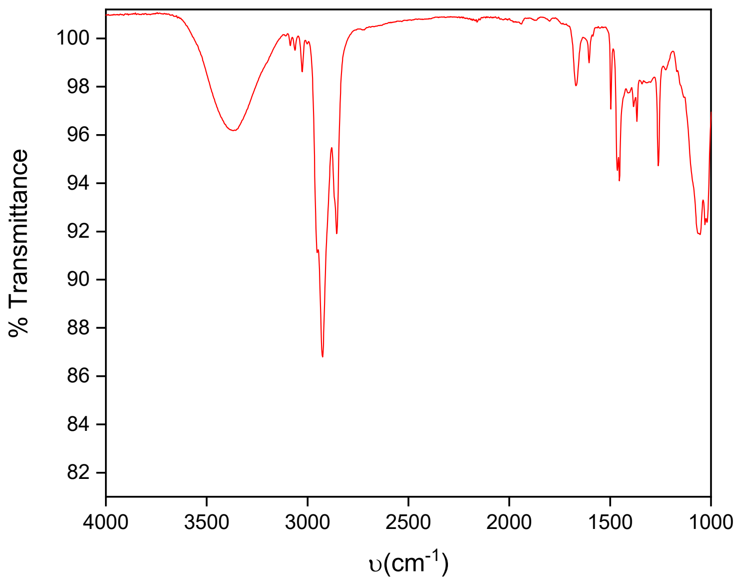

Figure S122. FT-IR spectrum for *anti*-**6**.

6. DFT Calculations

Computational Methods. Structure geometries were fully optimized with Gaussian16/A.03 software package[25] using the unrestricted BP86 functional and the 6-311G(d,p) basis set. Single points were calculated at this geometry with empirical dispersion and the SMD continuum solvent model for diethyl ether. Stationary points were confirmed by frequency analysis. Geometries are reported as Cartesian coordinates. All energies are reported in kcal/mol and are the sum of electronic energies from the single point calculations and thermal free energy corrections at 298 K from the fully optimized structures. All energy values are normalized to the energy of bound aldehyde complex **45a** in the triplet spin state. Single points were also calculated of the major pathway using the unrestricted M06L functional and def2-TZVP basis set with empirical dispersion and the SMD continuum solvent model for diethyl ether. All structures were optimized by truncating the isopropyl groups on the ligand to methyl groups. Select structures were also fully optimized using isopropyl groups (Figure X). All singlet spin structures of the major pathway were examined for open-shell singlet diradical character by reoptimization of the restricted, closed-shell structures. This was done using UBP86 with the guess=(mix,always) keyword and options. Only intermediate **48** was found to have open-shell character.

Reaction Mechanism. The first intermediate modeled in the reaction mechanism is aldehyde complex (**45**). Initial C–C bond formation occurs through migratory insertion (**46**), forming a metallacycle (**47**). At this point, there are two pathways to form the dioxolane and enone products.

A second aldehyde can bind to the metallacycle to form another bound aldehyde complex (**48**). Migratory insertion occurs (**49**); subsequently, a larger metallacycle (**50**) is formed. Upon rearrangement of the metallacycle (**51**), the final C–O bond is formed upon reductive elimination (**52**), forming a bound dioxolane complex (**53**).

Alternatively, the other isomer of **47** can undergo β-hydride elimination (**54**), forming nickel–hydride complex (**55**). Upon reductive elimination (**56**), the bound product is formed (**57**).

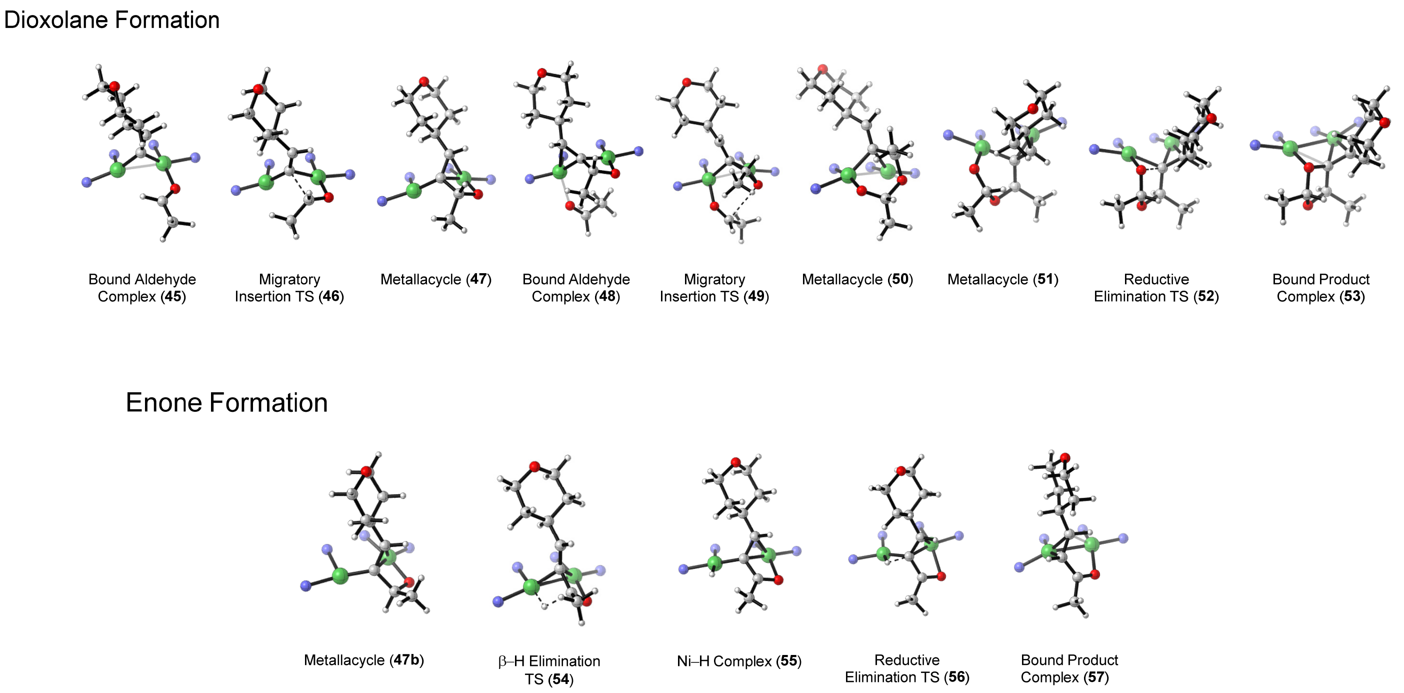

Figure S123. Optimized structures of intermediates and transition states.

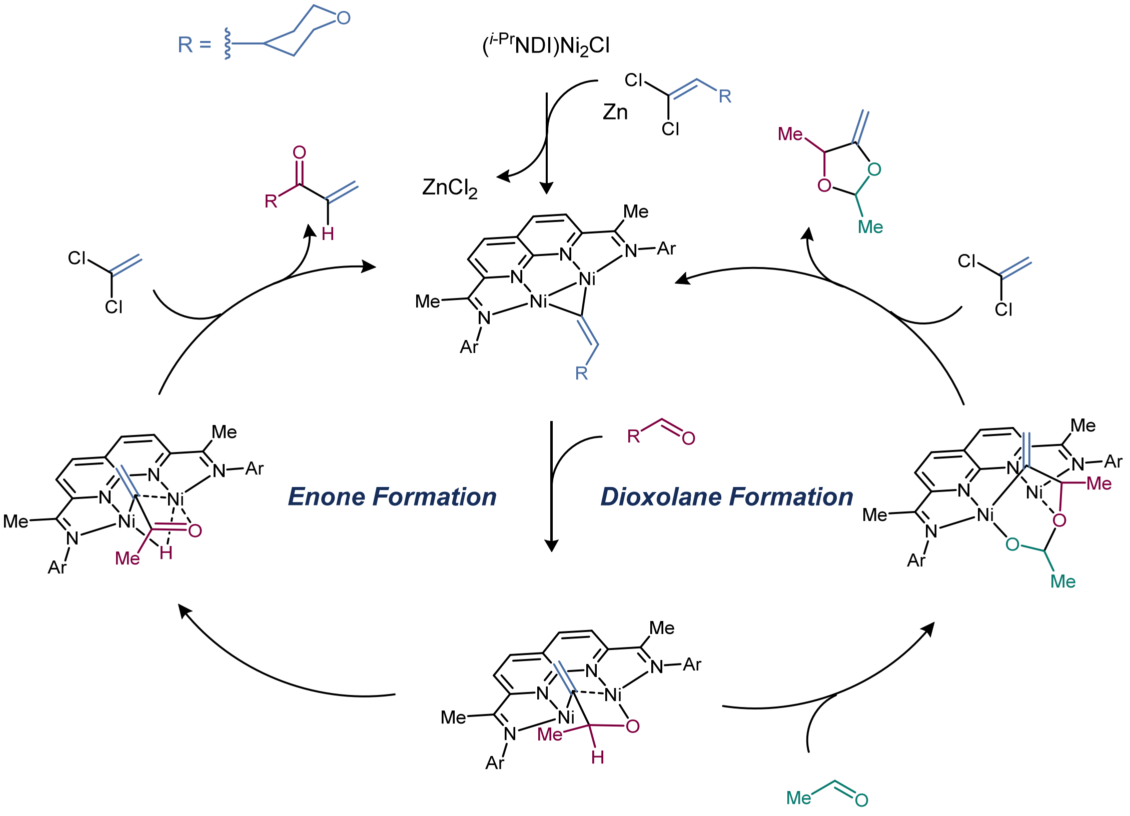

Figure S124. An overview of the proposed catalytic cycle.

[2 + 2 + 1] Reaction Selectivity: *E/Z* Selectivity Determining Step. There are two possible orientations of the vinylidene, as well as two orientations of each aldehyde, leading to eight total pathways to four possible products. The first step of the reaction is selectivity-determining and irreversible. Therefore, only the first migratory insertion transition state was calculated for the *(E)-anti* and *(E)-syn* products, which are not experimentally observed.

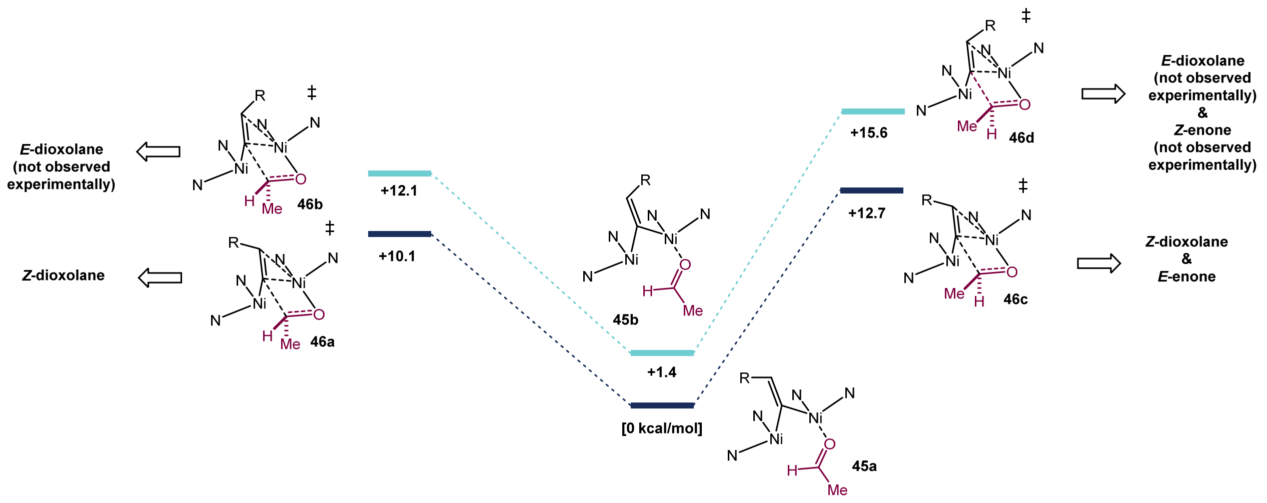

Figure S125. Energy diagram for E/Z Selectivity Determining Step

Anti:Syn Selectivity. Due to the dependence of the anti/syn selectivity on reductant and solvent, we did not attempt to explain the experimentally observed diasteroselectivity computationally. By varying the orientation of each aldehyde, there are four potential pathways, with two pathways leading to the (*Z*)-anti and the other two leading to the (*Z*)-syn products. Only the lowest energy pathway leading to the (*Z*)-anti and (*Z*)-syn dioxolane are presented here.

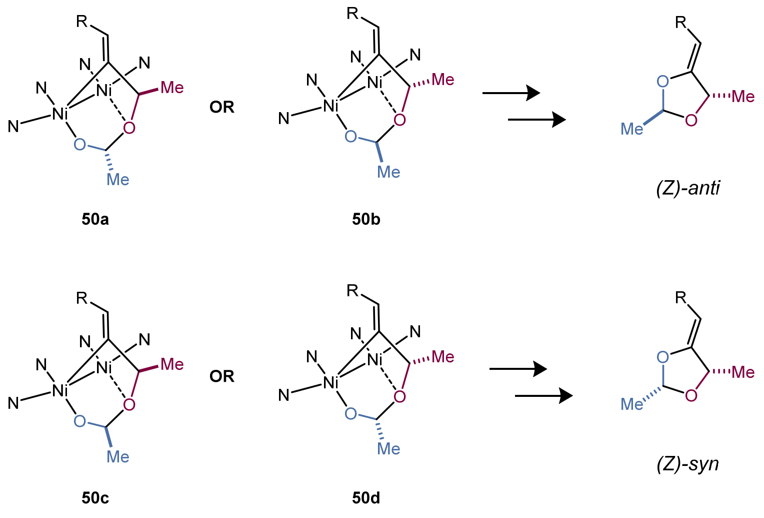

Figure S126. Metallacycles leading to (*Z*)-dioxolanes.

Catalyst Oxidation State. The first step of the reaction can occur with either one or no chlorines bound to the catalyst. However, complex **50** is not viable without reduction of a chlorine from the catalyst. Previous studies have demonstrated the competency of zinc to reduce metalacyclic intermediates such as **47**. Therefore, reduction could potentially happen before or after formation of metallacycle **47**.

C–O Reductive Elimination to Form a Methylene Epoxide. The reductive elimination step to form the methylene epoxide was performed and found to be prohibitively high in energy.

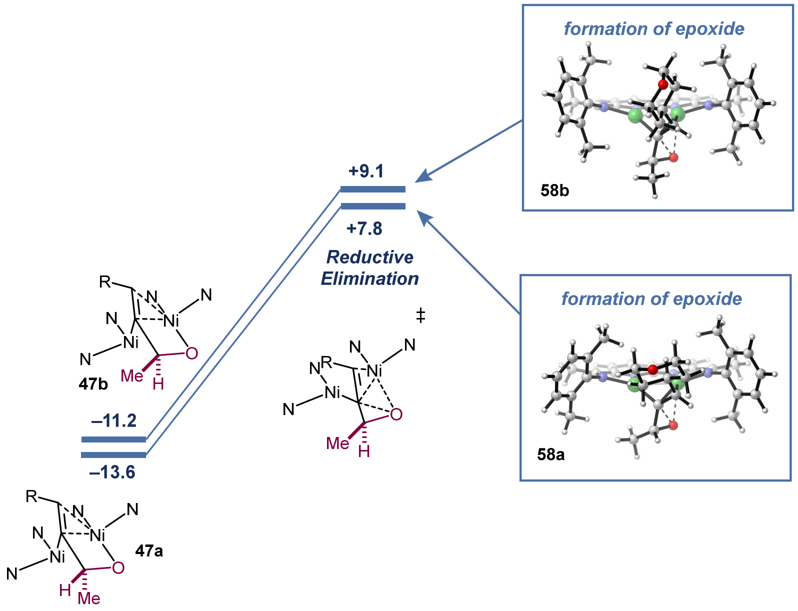

Figure S127. Reductive elimination transition states leading to epoxide products.

Calculated Reaction Coordinate Diagrams. The reaction coordinate diagrams are shown below. All energies are normalized to the energy of the triplet (*Z*) bound aldehyde complex (**45a**). There are four possible reaction coordinate diagrams leading to the *Z* isomers of the dioxolane that are shown below.

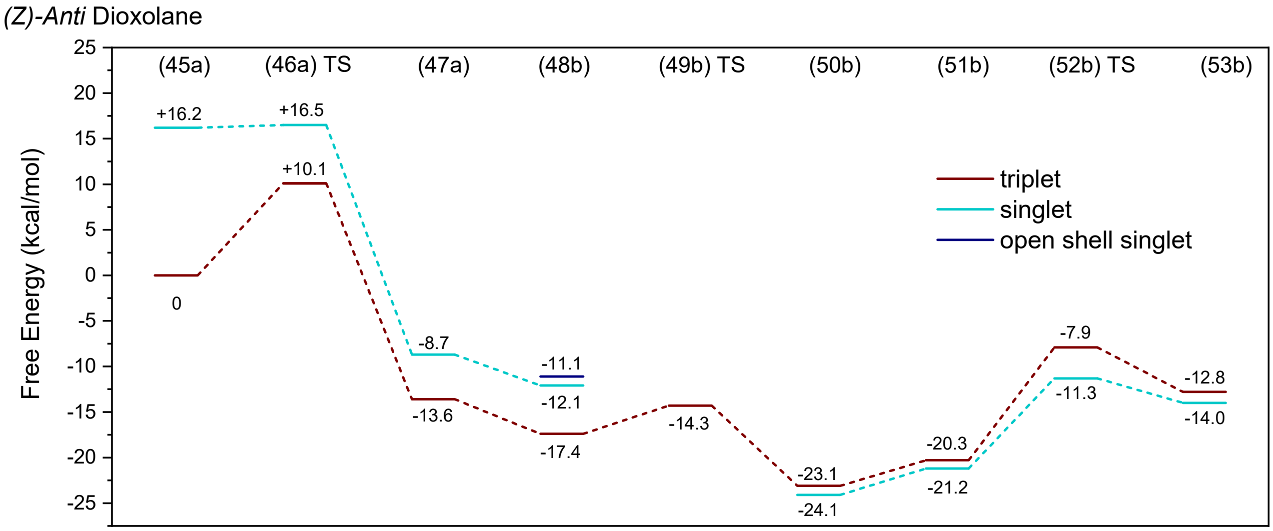

Figure S128. Energy diagram for the lowest energy formation of (*Z*)-*anti* dioxolane.

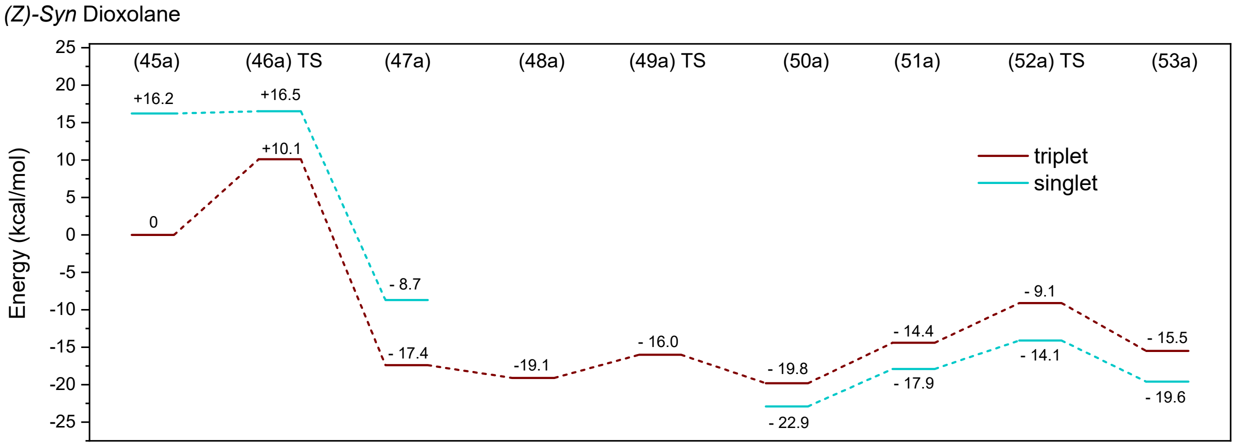

Figure S129. Energy diagram for the lowest energy formation of (*Z*)-*syn* dioxolane.

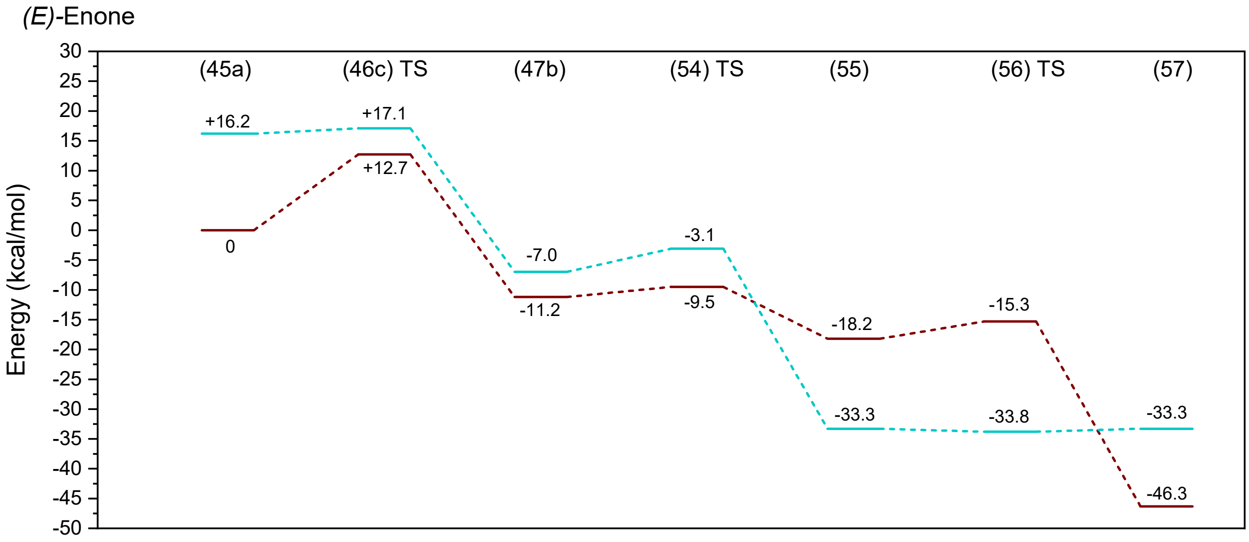

Figure S130. Energy diagram for (*E*)-enone

Table S4: Structure energies, relative to the triplet bound aldehyde complex **45a** (kcal/mol). Absolute energies in parentheses (Hartree). All energies were fully optimized using (U)BP86/6-311g(d,p) level of DFT. Single point calculations were performed on this geometry with empirical dispersion and the smd solvent continuum model for ether. Thermal corrections were applied to single point calculations from the fully optimized structures.

**Structure** UBP86/6-311g(d,p)	EE + Thermal Free Energy Correction	Charge	Number of Imaginary Frequencies
**45a (S=0)**	16.2 (-3025705.371)	0	0
**45a (S=1)**	0 (-3025721.552)	0	0
**45b (S=1)**	-1.0 (-3025722.509)	0	0
**46a (S=0)**	16.5 (-3025705.027)	0	1
**46a (S=1)**	10.1 (-3025711.413)	0	1
**46b (S=0)**	19.4 (-3025702.14)	0	1
**46b (S=1)**	12.1 (-3025709.408)	0	1
**46c (S=0)**	17.1 (-3025704.448)	0	1
**46c (S=1)**	12.7 (-3025708.858)	0	1
**46d (S=0)**	21.3 (-3025700.232)	0	1

**46d (S=1)**	15.6 (-3025705.987)	0	1
**47a (S=0)**	-8.7 (-3025730.22)	0	0
**47a (S=1)**	-13.6 (-3025735.196)	0	0
**47b (S=0)**	-7.0 (-3025728.505)	0	0
**47b (S=1)**	-11.2 (-3025732.755)	0	0
**48a (S=0)**	-12.1 (-3122278.159)	0	0
**48a (S=1)**	-17.4 (-3122283.53)	0	0
**48a (S=0) open shell**	-11.1 (-3122277.182)	0	0
**48b (S=1)**	-19.1 (-3122285.181)	0	0
**49a (S=1)**	-14.3 (-3122280.382)	0	1
**49b (S=1**	-16.0 (-3122282.095)	0	1
**50a (S=0)**	-24.1 (-3122290.162)	0	0
**50a (S=1)**	-23.1 (-3122289.189)	0	0
**50b (S=0)**	-22.9 (-3122288.967)	0	0
**50b (S=1)**	-19.8 (-3122285.888)	0	0
**51a (S=0)**	-21.2 (-3122287.275)	0	0
**51a (S=1)**	-20.3 (-3122286.426)	0	0
**51b (S=0)**	-17.9 (-3122283.945)	0	0
**51b (S=1)**	-14.4 (-3122280.446)	0	0
**52a (S=0)**	-11.3 (-3122277.407)	0	1
**52a (S=1)**	-7.9 (-3122273.938)	0	1
**52b (S=0)**	-14.1 (-3122280.171)	0	1
**52b (S=1)**	-9.1 (-3122275.222)	0	1
**53a (S=0)**	-14.0 (-3122280.055)	0	0
**53a (S=1)**	-12.8 (-3122278.871)	0	0
**53b (S=0)**	-19.6 (-3122285.683)	0	0
**53b (S=1)**	-15.5	0	0

	(-3122281.606)		
**54 (S=0)**	-3.1 (-3025724.659)	0	1
**54 (S=1)**	-9.5 (-3025731.041)	0	1
**55 (S=0)**	-33.3 (-3025754.845)	0	0
**55 (S=1)**	-18.2 (-3025739.755)	0	0
**56 (S=0)**	-33.8 (-3025755.345)	0	1
**56 (S=1)**	-15.3 (-3025736.83)	0	1
**57 (S=0)**	-33.3 (-3025755.756)	0	0
**57 (S=1)**	-46.3 (-3025767.806)	0	0
**58a (S=1)**	7.8 (-3025713.771)	0	1
**58b (S=1)**	9.1 (-3025712.454)	0	1

Table S5: Structure energies, relative to the triplet bound aldehyde complex **45a** (kcal/mol). Absolute energies in parentheses (Hartree). Single point energies were calculated using (U)M06L/def2-TZVP level of DFT with empirical dispersion and the smd solvent continuum model for ether. Thermal corrections were applied to single point calculations from the (U)BP86/6-311g(d,p) fully optimized structures. No significant changes in the reaction mechanism were observed (i.e. the selectivity-determining step was found to be the same as the calculations performed using the BP86 functional).

**Structure** UM06L/def2TZVP	EE + Thermal Free Energy Correction
**45a (S=0)**	26.0 (-3025290.953)
**45a (S=1)**	0 (-3025315.548)
**46a (S=0)**	28.2 (-3025287.327)
**46a (S=1)**	14.0 (-3025301.594)
**47a (S=0)**	2.3 (-3025311.831)
**47a (S=1)**	-11.1 (-3025326.634)
**48a (S=1)**	-9.7 (-3121864.593)
**49a (S=1)**	-9.3 (-3121864.128)
**50a (S=0)**	-8.1 (-3121863.006)
**50a (S=1)**	-14.0 (-3121868.879)

**51a (S=0)**	-3.4 (-3121858.253)
**51a (S=1)**	-6.5 (-3121861.424)
**52a (S=0)**	5.7 (-3121849.162)
**52a (S=1)**	4.5 (-3121850.42)
**53a (S=0)**	-1.1 (-3121856.022)
**53a (S=1)**	-4.9 (-3121859.792)

Table S6: Structure energies with isopropyl groups present on the ligand, relative to the triplet bound aldehyde complex **45a** (kcal/mol). Absolute energies in parentheses (Hartree). All energies were fully optimized using (U)BP86/6-311g(d,p) level of DFT. Single point calculations were performed on this geometry with empirical dispersion and the smd solvent continuum model for ether. Thermal corrections were applied to single point calculations from the fully optimized structures.

**Structure** UBP86/6-311G(d,p)	EE + Thermal Free Energy Correction
**45a-iPr (S=1)**	0 (-3223019.477)
**46a-iPr (S=1)**	11.2 (-3223008.252)
**46b-iPr (S=1)**	14.3 (-3223005.172)
**46c-iPr (S=1)**	14.2 (-3223005.272)
**47a-iPr (S=1)**	-12.3 (-3223031.713)
**47b-iPr (S=1)**	-11.7 (-3223031.148)
**48a-iPr (S=1)**	-15.3 (-3319579.34)
**49a-iPr (S=1)**	-10.0 (-3319574.005)
**54-iPr (S=1)**	-8.8 (-3223028.233)

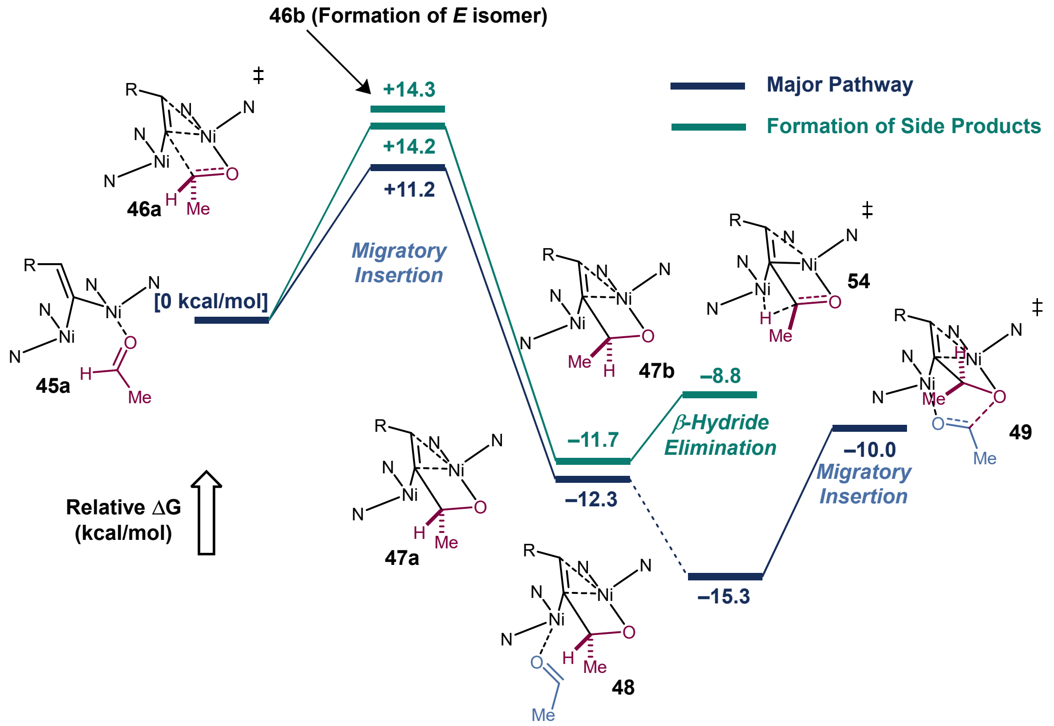

Figure S118. Energy diagram for select steps of the reaction mechanism with full isopropyl groups on the catalyst.

9. References

[1] Zhou, Y.-Y.; Hartline, D. R.; Steiman, T. J.; Fanwick, P. E., and Uyeda. C. “Dinuclear Nickel Complexes in Five States of Oxidation Using a Redox-Active Ligand” *Inorg. Chem.,* **2014,** *53* (21), 11770-11777

[2] Steiman, T. J.; Kalb, A. E.; Coombs, J. C.; Kirkland, J. K.; Torres, H.; Ess, D. H.; Uyeda, C. "Dinickel-Catalyzed Vinylidene–Alkene Cyclization Reactions" *ACS Catal.,* **2021**, 14408-14416.

[3] Zhou, Y.-Y., and Uyeda, C. “Catalytic Reductive [4+1]-Cycloadditions of Vinylidenes and Dienes.” *Science,* **2019**, 363(*6429*), 857-862.

[4] Zhou, Y.-Y.; Uyeda, C. "Reductive Cyclopropanations Catalyzed by Dinuclear Nickel Complexes." *Angew. Chem., Int. Ed.,* **2016**, *128* (9), 3223-3227.

[5] Pal, S., Zhou, Y.-Y., and Uyeda C. “Catalytic Reductive Vinylidene Transfer Reactions” *J. Am. Chem. Soc.,* **2017** 139 *(34*), 11686-11689.

[6] Watanabe, H.; Machida, K.; Itoh, D.; Nagatsuka, H.; Kitahara, T. “Novel asymmetric oxy-michael addition reaction of the chiral ketones to the achiral γ- or δ-hydroxy-α,β-unsaturated carbonyl compounds” *Chirality* **2001**, *13*, 379–385.

[7] Grisin, A.; Oliver, S.; Ganton, M. D.; Basca, J.; Evans, P. W. “Diastereoselective construction of *anti*-4,5-disubstituted-1,3-dioxolanes *via* a bismuth-mediated two-component hemiacetal oxa-conjugate addition of γ-hydroxy-α,β-unsaturated ketones with paraformaldehyde” *Chem. Commun.* **2015**, *51*, 15681–15684.

[8] Coombs, J. R.; Zhang, L.; Morken, J. P. “Enantiomerically Enriched Tris(boronates): Readily Accessible Conjunctive Reagents for Asymmetric Synthesis” *J. Am. Chem. Soc.* **2014**, *136*, 16140–16143.

[9] Mori, K.; Kiyota, H. “A New Synthesis of (3*S*,4*R*)-8-Nonene-3,4-diol, the Key Intermediate for the Synthesis of (+)-*endo*-Brevicomin” *Liebigs Ann. Chem.* **1992**, 989–992.

[10] Kroutil, W.; Mischitz, M.; Faber, K. “Deracemization of (±)-2,3-disubstituted oxiranes *via* biocatalytic hydrolysis using bacterial epoxide hydrolases: kinetics of an enantioconvergent process” *J. Chem. Soc. Perkin Trans. 1* **1997**, 3629–3636.

[11] Drop, A.; Wojtasek, H.; Frąckowiak-Wojtasek, B. “Synthesis of disparlure and monachalure enantiomers from 2,3-butanediacetals” *Beilstein J. Org. Chem.* **2020**, *16*, 616–620.

[12] Spallarossa, M., Wang, Q. Riva, R., and Zhu, J. “Synthesis of Vinyl Isocyanides and Development of a Convertible Isonitrile” *Org. Lett.,* **2016** 18 (*7*), 1622-1625

[13] Makings, L. R.; Garcia-Guzman Blanco, M.; Hurley, D. J.; Drutu, I.; Raffai, G.; Bergeron, D. M.; Nakatani, A.; Termin, A. P.; Silina, A. “Preparation of benzo-fused spiropiperidine compounds as modulators of muscarinic receptors” WO 2007076070, 2007

[14] Murphy S. K., and Dong V. M. “Enantioselective Ketone Hydroacylation Using Noyori’s Transfer Hydrogenation Catalyst” *J. Am. Chem. Soc.,* **2013,** 135(*15*), 5553-5556

[15] Tu, Y.; Zeng, X.; Wang, H.; Zhao, J. “A Robust One-Step Approach to Enamides” *Org. Lett.,* **2018**, 20(*1*), 280-283.

[16] Taylor, W. G. “Synthesis of 5,5-Dichloro-4-pentenoic Acid by the Wittig Reaction with Bromotrichloromethane and Triphenylphosphine” *J. Org. Chem.,* 1**981**, 46, 4292-4294.

[17] Friden-Saxin, M,; Pemberton, N.; Andersson, K. S.; Dyrager, C.; Friberg, A.; Grotli, M.; Luthman, K. "Synthesis of 2-Alkyl-Substituted Chromone Derivatives Using Microwave Irradiation" *J. Org. Chem.,* **2009**, 74(*7*), 2755-2759.

[18] Jui, N. T.; Lee, Esther C. Y.; MacMillan, D. W. C. "Enantioselective Organo-SOMO Cascade Cycloadditions: A Rapid Approach to Molecular Complexity from Simple Aldehydes and Olefins" *J. Am. Chem. Soc.,* **2010**, 132(*29*), 10015-10017.

[19] a) Alcohol synthesis: Vulovic, B.; Trmcic, M.; Matovic, R.; Saicic, R. N. "Cyclization Reactions of Oxyallyl Cation. A Method for Cyclopentane Ring Formation" *Org. Lett.* **2019**, 21, (*23*), 9618-9621. b) Aldehyde synthesis: Dachavaram, S. S.; Kalyankar, K. B.; Das, S. "First stereoselective total synthesis of Neocosmosin A: a facile approach" *Tet. Lett.* **2014**, 55(*41*), 5629-5631.

[20] Fraser, A. L.; Menzies, S. K.; King, E. F. B.; Tulloch, L. B.; Gould, E. R.; Zacharova, M. K.; Smith, T. K.; Florence, G. J. "Design and Synthesis of Broad Spectrum Trypanosomatid Selective Inhibitors" *ACS Infect. Dis.,* **2018**, 4(*4*), 560-567.

[21] Takeoka, G. R.; Buttery, R. G.; and Perrino, C. T. Jr. "Synthesis and Occurrence of Oxoaldehydes in Used Fying Oils" *J. Agric. Food Chem.,* **1995,** 43(*1*), 22-26.

[22] Matuszewska, I., Leniewski, A., Roszkowski P., Czarnocki, Z. "Synthesis of a Novel Class of Fatty Acids-Derived Isoquinolines" *Chem. Phys. Lipids,* **2005,** 135, 131–145.

[23] Buhler, H.; Effenberger, F.; Forster, S.; Roos, J.; Wajant, H. "Enzyme-catalyzed reactions, part 47. Substrate specificity of mutants of the hydroxynitrile lyase from Manihot esculenta" *ChemBioChem* **2003**, 4, (*2-3*), 211-216

[24] Davies, A. T.; Taylor, J. E.; Douglas, J. Collett, C. J.; Morrill, L. C.; Fallan, C.; Slawin, A. M. C.; Churchill, C. and Smith, A. D. "Stereospecific Asymmetric N-Heterocyclic Carbene (NHC)-Catalyzed Redox Synthesis of Trifluoromethyl Dihydropyranones and Mechanistic Insights" *J. Org. Chem.* **2013**, 78, *18*, 9243–9257.

[25] Gaussian 16, Revision A.03, Frisch, M. J.; Trucks, G. W.; Schlegel, H. B.; Scuseria, G. E.; Robb, M. A.; Cheeseman, J. R.; Scalmani, G.; Barone, V.; Petersson, G. A.; Nakatsuji, H.; Li, X.; Caricato, M.; Marenich, A. V.; Bloino, J.; Janesko, B. G.; Gomperts, R.; Mennucci, B.; Hratchian, H. P.; Ortiz, J. V.; Izmaylov, A. F.; Sonnenberg, J. L.; Williams-Young, D.; Ding, F.; Lipparini, F.; Egidi, F.; Goings, J.; Peng, B.; Petrone, A.; Henderson, T.; Ranasinghe, D.; Zakrzewski, V. G.; Gao, J.; Rega, N.; Zheng, G.; Liang, W.; Hada, M.; Ehara, M.; Toyota, K.; Fukuda, R.; Hasegawa, J.; Ishida, M.; Nakajima, T.; Honda, Y.; Kitao, O.; Nakai, H.; Vreven, T.; Throssell, K.; Montgomery, J. A., Jr.; Peralta, J. E.; Ogliaro, F.; Bearpark, M. J.; Heyd, J. J.; Brothers, E. N.; Kudin, K. N.; Staroverov, V. N.; Keith, T. A.; Kobayashi, R.; Normand, J.; Raghavachari, K.; Rendell, A. P.; Burant, J. C.; Iyengar, S. S.; Tomasi, J.; Cossi, M.; Millam, J. M.; Klene, M.; Adamo, C.; Cammi, R.; Ochterski, J. W.; Martin, R. L.; Morokuma, K.; Farkas, O.; Foresman, J. B.; Fox, D. J. Gaussian, Inc., Wallingford CT, 2016.
